# Zwitterionic Bergman cyclization triggered polymerization gives access to metal-graphene nanoribbons using a boron metal couple

**DOI:** 10.1038/s42004-023-00866-w

**Published:** 2023-04-07

**Authors:** Dinesh V. Vidhani, Rosemary Ubeda, Thalia Sautie, Diana Vidhani, Manoharan Mariappan

**Affiliations:** 1 Department of Math & Natural Science, Miami Dade College, Miami Dade College, 627 SW 27th Ave, Miami, FL 33135 USA; 2 Miami Dade Virtual School, 560 NW 151st, Miami, FL 33169 USA; 3 Department of Natural Science North Florida College, 325 Turner Davis Dr, Madison, FL 32340 USA

**Keywords:** Reaction mechanisms, Reaction mechanisms, Electronic properties and devices, Density functional theory, Structure prediction

## Abstract

With the rapid growth in artificial intelligence, designing high-speed and low-power semiconducting materials is of utmost importance. This investigation provides a theoretical basis to access covalently bonded transition metal-graphene nanoribbon (TM-GNR) hybrid semiconductors whose DFT-computed bandgaps were much narrower than the commonly used pentacene. Systematic optimization of substrates containing remotely placed boryl groups and the transition metals produced the zwitterions *via* ionic Bergman cyclization (*i-BC*) and unlocked the polymerization of metal-substituted polyenynes. Aside from *i-BC*, the subsequent steps were barrierless, which involved structureless transition regions. Multivariate analysis revealed the strong dependence of activation energy and the cyclization mode on the electronic nature of boron and Au(I). Consequently, three regions corresponding to radical Bergman (*r-BC*), ionic Bergman (*i-BC*), and ionic Schreiner-Pascal (*i-SP*) cyclizations were identified. The boundaries between these regions corresponded to the mechanistic shift induced by the three-center-three-electron (3c-3e) hydrogen bond, three-center-four-electron (3c-4e) hydrogen bond, and vacant p-orbital on boron. The ideal combination for cascade polymerization was observed near the boundary between *i-BC* and *i-SP*.

## Abstract (web summary)

Transition-metal-graphene nanoribbons (TM-GNR) exhibit unusual optoelectronic properties, however, synthetic access to such materials remains highly challenging. Here, the authors report a quantum chemical substrate design via DFT to access TM-GNR hybrid semiconductors using zwitterionic Bergman cyclization-triggered cascade polymerization.

## Introduction

**Fig. 1:**
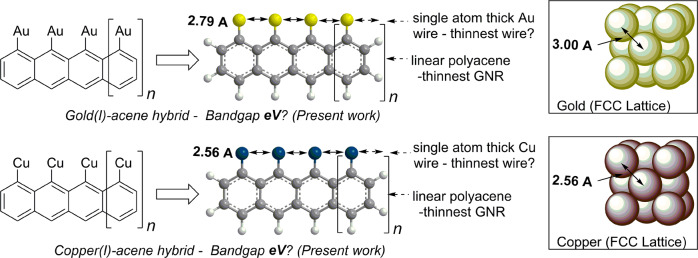
This figure illustrates a proposed metal-polyacene hybrid semiconductor created through ionic BC-triggered cascade polymerization. It displays the thinnest linear polyacene covalently bonded to a single-atom-thick transition metal wire, with the distance between metal atoms similar to that of the face-centered cubic lattice of gold and copper crystals.

As we reach the quantum limit of silicon-based semiconductors, graphene-based materials have become the focus of intense research due to their applications in solar cells^1,2^, OLEDs^3^, bioengineering^4,5^, drug and gene delivery systems^6,7^, composite materials^8^, and energy storage devices^9,10^. Despite their extraordinary mechanical and optoelectronic properties, vanishing bandgaps in graphene limits its ability to replace silicon-based semiconductors. Graphene nanoribbons (GNRs), which are quasi-one-dimensional polyacene sheets with a width of less than 100 nm and a bandgap between 1–1.6 eV, offer a better solution to the shortcomings of silicon and graphene-based materials (Fig. S1 in SI)^11^. The structure and material properties of graphene nanoribbons (GNRs) depend heavily on the method of synthesis. For example, the electronic properties of GNRs with jagged edges created using electron-beam lithography differ from the more precise armchair or zigzag GNRs made using a bottom-up technique utilizing polycyclic molecules^12^. In this context, linearly fused polyaromatic hydrocarbons (PAH) with a general formula C_4n+2_H_2n+4_ are essential precursors for synthesizing GNRs and serve as molecular models to understand their material properties (Fig. S1 in SI)^13^. However, the linear structure of acenes contains a single Clar or aromatic sextet (six π-electrons), making them susceptible to oxidation or polymerization and challenging to synthesize, especially when the number of rings is high^14–19^. Hence, producing these molecules using traditional synthetic organic methods has been difficult^20^. Making metal-hybrid polyacene materials is even more complex and requires special techniques (Fig. 1). These materials are predicted to have a combination of GNRs and pure metals properties, especially if the distance between the covalently bonded metal atoms closely matches the metallic bond distance in the pure metal.

**Fig. 2:**
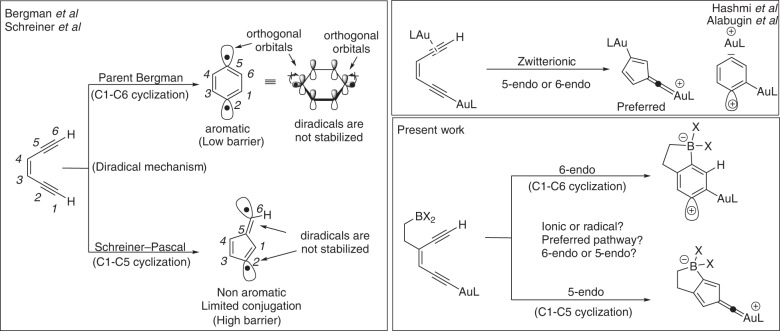
Possible outcomes in the cyclization of enediynes. The left box shows the conventional C_1_–C_6_ Bergman (*BC*) and C_1_–C_5_ Schreiner–Pascal (*SP*) cyclizations giving diradicals. The top right depicts the dual Au(I)-catalyzed zwitterionic version of *BC*, and the bottom right box shows the present work on the boron-Au(I) triggered zwitterionic *BC* and *SP* cyclizations.

Among various methods, Bergman cyclization (*BC*) provides a convenient strategy for synthesizing a wide range of fused polyaromatic compounds, such as substituted rylenes. Mechanistically, *BC* can be seen as a two-step, high activation energy pericyclic rearrangement, with an unstable diradical intermediate between the two transition states for ring formation and opening (Fig. 2). Roth et al. experimentally corroborated Bergman’s prediction of high activation energy, with a measured ΔH^#^ of 28.7 ± 0.5 kcal/mol at 200 °C^21–23^. This is because, unlike a concerted pericyclic process, the number of bonds broken and formed in the cycloaromatization step are not conserved, leading to the formation of a six-membered aromatic diradical that is orthogonal to the aromatic π-system and cannot take advantage of adjacent conjugated systems (Fig. 2, top left). As a result, this diradical is highly reactive and can produce a range of fused cycloaromatic compounds, yet it often gives low yields, 5-endo products (Fig. 2, bottom left), or reduced products. To overcome these limitations, the exploration of ionic-*BC* is essential^24–29^. In a recent study, Hashmi *et al*. found that 5-endo cyclization of dual σ,π-Au(I) substituted enediynes led to a non-aromatic fulvene-type cation (Fig. 2, top right)^30,31^. This route is comparable to Schreiner–Pascal (*SP*) cyclization; however, the classic *SP* cyclization is not preferred due to its high activation energy^32^ and the formation of non-aromatic fulvene diradicals (Fig. 2, bottom left)^33^, which is not a feature of the dual σ,π-Au(I)-catalyzed protocol. Subsequently, Alabugin *et al*. explored the dual σ,π-Au(I)-catalyzed Bergman cyclization, providing valuable insights into the nature of 1,4-zwitterionic species^34–37^.

**Fig. 3:**
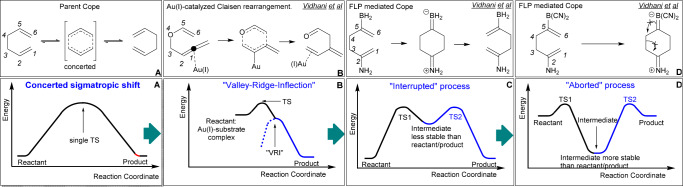
Evolution of concerted potential energy surface (PES) into interrupted and aborted pathways. **A** Parent Cope rearrangement, **B** Au(I)-catalyzed Claisen rearrangement displaying inflection along the PES, **C** FLP-mediated interrupted Cope rearrangement, and **D** FLP-mediated aborted Cope rearrangement.

From this viewpoint, we envisioned polyenynes containing tunable σ-Au(I)-acetylide and boryl groups to undergo the desired cascade polymerization with high 6-endo selectivity (Fig. 2, bottom right). Previous studies have shown that the potential energy surface (PES) of concerted pericyclic rearrangements can be altered by the influence of transition metals or frustrated Lewis pairs (FLPs), leading to an interrupted or aborted process (Fig. 3)^38^. Notably, Au(I)-catalyzed Claisen rearrangements of allenyl vinyl ethers and propargyl vinyl ethers appear to be switching from a concerted to a stepwise pathway (Fig. 3B). The near-perfect alignment of σ*(C3-O) and σ(C2-Au) at the second transition state (TS2), which manifests as an inflection on the PES, corroborates this phenomenon^39–43^. Alternatively, the Cope rearrangement mediated by the FLPs can be interrupted by a zwitterionic intermediate in which the boryl group holds onto the electron density it acquired, thus slowing down or aborting σ(C3–C4) bond scission (Fig. 3C, D). The present work aimed to explore the capability of zwitterions in (*BC*), and the stereoelectronic effects it can have on the cascade cyclization of polyenynes leading to metal-polyacene semiconducting materials. An extensive DFT analysis helped to design the polyenyne substrates that contained the ideal combination of a boryl group and Au(I)L and enabled the polyenynes to undergo a zwitterionic BC reaction without any side reactions. Further, the calculated HOMO-LUMO gaps and reorganization energies of the resulting transition metal-polyacene hybrid material were comparable to popular p-type semiconductors, such as pentacene.

## Results and discussion

**Fig. 4:**
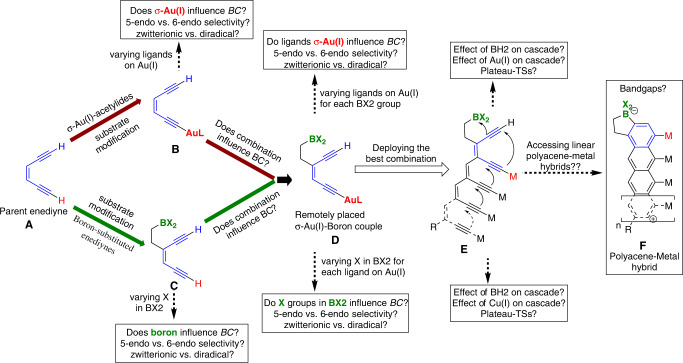
The flow chart provides a logical evolution of substrates from the parent enediyne into multifaceted polyenynes that can form polyacene-metal hybrids. Each modification in the substrate design was investigated separately using DFT level calculations [**A**_(Parent enediyne)_ → **D**_(Au/boron couple)_]. Structures **B** and **C** correspond to mono σ-Au(I) and mono boryl substituted enediynes. Structure **E** corresponds to the polyene containing the best combination of Au(I)/boryl couple. Structure **F** corresponds to the metal-substituted polyacenes product.

The polyenynes for the present investigation contain three major components (1) the boryl group, (2) a covalently bonded transition metal with the alkyne, and (3) the repeating polyenyne motifs. We hypothesized that the boryl groups play a critical role in controlling the barrier, deciding the 5-endo/6-endo selectivity, and selecting between radical and zwitterionic pathways. Based on the recent independent studies by the Alabugin and Hashmi groups, we further theorized that the electronic nature of Au(I)-acetylide would affect the stability of the developing *sp*^*2*^-hybridized β-carbocation at C2 of enediyne (Fig. 2). However, for the success of this strategy, it was essential that the effect of Au(I)-acetylide was synergic to the boryl group in the multistep cascade polymerization. Thus, before analyzing the cascade process, we investigated the role of ligand on Au(I) (Fig. 4B), the substituents on the boryl group (Fig. 4C), and the combined effect of Au(I) (Fig. 4D) and boryl groups on the overall nature of *BC* in substituted enediynes (Fig. 4E).

### Bergman cyclization of Au(I)-substituted enediynes

**Fig. 5:**
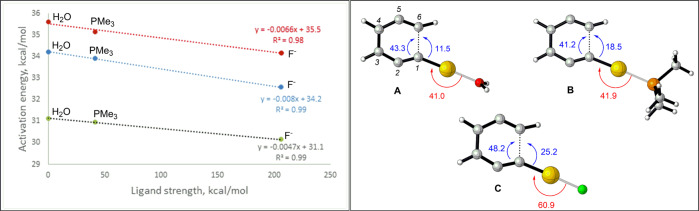
Plot on the left shows the dependence of activation energy on the strength of the ligand. Red, blue, and gray dotted lines in the plot correspond to the activation energies calculated at the B3LYP/6-311+G**/def2-TZVP, B3LYP/LANL2DZ, and PBE0/6-311+G**/def2-TZVP levels respectively. Structures **A**, **B** and **C** on the right correspond to the transition state geometries of substrates consisting of H_2_O, PMe_3_, and F^−^ attached to the Au(I)-acetylide, respectively. The values in red and blue correspond to interaction energies expressed in kcal/mol.

Previous studies of *BC* have shown a strong agreement between experimental kinetics and the data obtained using several theoretical methods, including CCSD(T), CASSCF, CASPT2, MBPT, and various DFT approaches^44–48^. In particular, the combination of experimental and computational studies demonstrated the capability of B3LYP and PBE0 methods to accurately predict the activation barriers of pericyclic rearrangements such as Bergman cyclizations, Cope rearrangement, Claisen rearrangement, and several transition metal-catalyzed reactions^33,37,38,40,45^. In the present study, the activation enthalpies for parent *BC* were found within 1 kcal/mol of experimentally determined barrier (32 kcal/mol, Table S1 in SI and Supplementary Data 1) when using B3LYP/LANL2DZ and PBE0/6-311+G**/def2-TZVP methods. Although both methods were dependable, the former was selected due to its reliability and affordability in predicting the properties of the compounds containing heavy atoms such as Au(I), Cu(I), and Br. From the mechanistic perspective, the parent enediynes and the systems containing σ-Au(I)-acetylides adopted a classical *BC*, generating 1,4-diradical *via* aromatic transition states [NICS(1): −11.0 ppm] and the activation energies ranging from 32–34 kcal/mol (Table S1 in SI). As for the ligands on Au(I), the fluoride ion showed the lowest barrier, followed by trimethyl phosphine and water (Table S1 in SI). The NBO analysis of the three transition states revealed that the radical stabilizing interaction from the remotely placed fluoride was far greater than trimethyl phosphine and water (Fig. 5). Furthermore, as evidenced by the interaction energies [48.2 kcal, Fig. 5], the fluoride ion enhances the orbital overlap between terminal carbon atoms and facilitates the formation of σ(C1–C6) bond. A similar radical stabilizing interaction is observed for water and trimethyl phosphine ligands, but the effect was not as pronounced (Fig. 5).

**Fig. 6:**
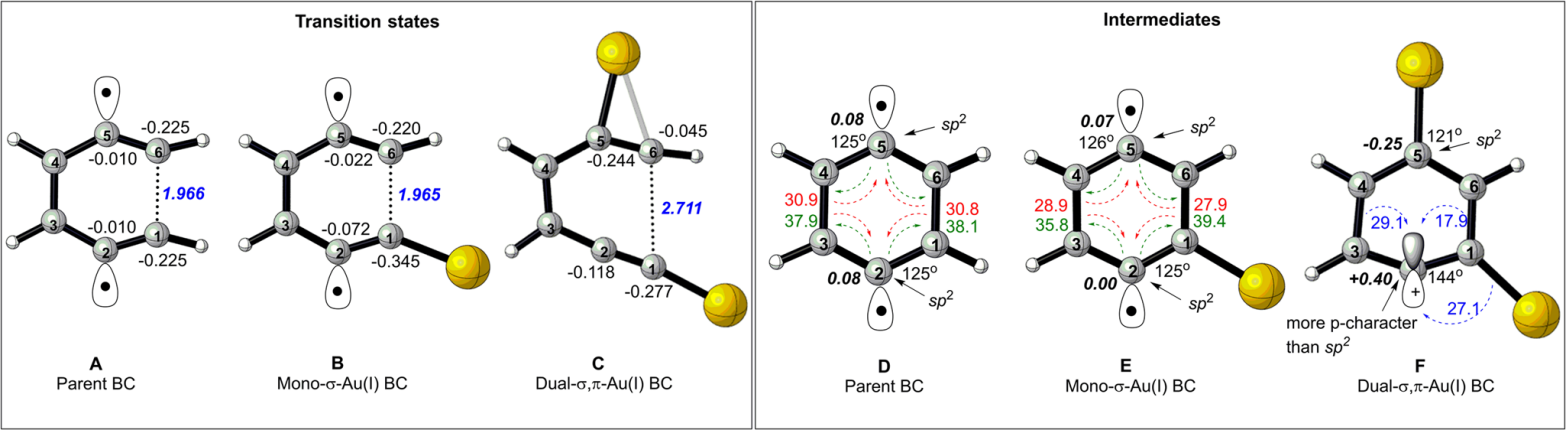
Bergman cyclization of parent enediyne, mono-Au(I)-acetylide, and dual Au(I)-catalyzed systems. Left: Transition state geometries of *BC* in parent enediyne (**A**), mono-Au(I)-acetylide system (**B**), and dual Au(I)-catalyzed system (**C**) obtained at the B3LYP/LANL2DZ level. Values in black and blue fonts reflect the NBO charges and the C1–C6 distance, respectively. Right: Geometries corresponding to the parent *BC* (**D**), *mono*-σ-Au(I) *BC* (**E**), and dual σ,π-Au(I) *BC* (**F**) intermediates. Values and arrows in red correspond to the interaction energies when σ(C1–C6) and σ(C3–C4) bonds are symmetrically delocalized into the antibonding orbital of closed shell C2–C5 diradical. Values and arrows in green reflect interaction energies when diradical is symmetrically delocalized into σ*(C_1_–C_6_) and σ*(C3–C4) bonds. Values and arrows in blue reflect interaction energies when σ(C1–C6), σ(C3–C4), σ(C1–Au) bonds are delocalized into the empty *p*-orbital of carbocation at C2.

The results to this point suggest that the ligands bound to σ-Au(I)L-acetylide do not alter the radical nature of the intermediate or the C1–C6 endo-selectivity of the enediyne substrates. Alabugin et al. demonstrated that this outcome might significantly change upon adding the second Au(I) that coordinates with the π(C5-C6) bond. The two Au(I) motifs in such dual σ,π-Au(I) activation produce stereoelectronically-stabilized zwitterion (Fig. 2)^33^. The energy of this trajectory was 16 kcal/mol lower than the 1,4-diradical pathway (Table S1 in SI). Conceptually, the TS of a pericyclic rearrangement can be visualized as the saddle point in a continuous spectrum spanned between the homolytic and the heterolytic bond-altering processes^49–51^. In the present study, the near-zero NBO charges at C2 and C5 of the parent enediyne and mono σ-Au(I) substrates clearly indicated the formation of aromatic 1,4-diradicals through homolytic bond-altering processes (Fig. 6A, B). The aromatic character of these radicals was confirmed by NICS (1) [Parent *BC*: −12.5 ppm; mono-Au(I) *BC*: −12 ppm]. However, the electrostatic repulsion between C1 and C6, as evidenced by their NBO charge distribution, contributed to the high activation energies in parent and mono-Au(I) systems (Fig. 6A, B).

**Fig. 7:**
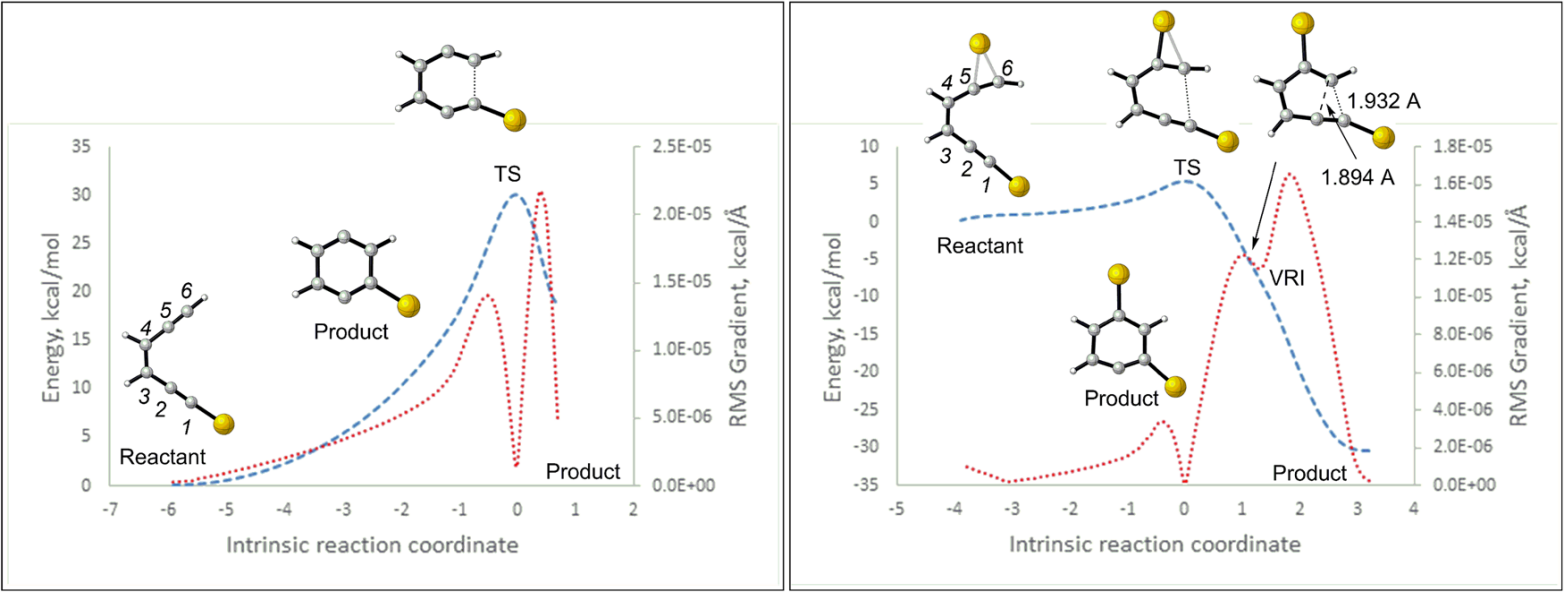
Comparison of the IRC paths for the mono-Au(I)-acetylide (left) and dual Au(I)-catalyzed system (right). Calculations were performed at the B3LYP/LANL2DZ level. The dashed blue line and dotted red line correspond to energy changes and RMS gradient along the intrinsic reaction coordinate. VRI corresponds to “Valley Ridge Inflection”^51–55^.

In contrast, the dual σ,π-Au(I) *BC* was characterized by an early non-aromatic-TS [NICS (1) = −5.2 ppm] where the NBO charges at C1 and C6 enabled the formation of σ-(C1–C6) bond (Fig. 6C). As previously demonstrated in Au(I)-catalyzed Claisen rearrangements (Fig. 3B), the potential energy surface (PES) of this reaction too exhibited a valley ridge inflection (VRI) along the IRC (Fig. 7, right)^52–56^. The C2–C6 and C1–C6 bond distances at the VRI further suggest that the 5-endo cyclization and 6-endo *BC* pathways bifurcate at the VRI and that they share a common non-aromatic-TS. Hence, several experimental studies have shown the formation of the fulvene-type intermediate from the Au(I)-catalyzed 5-endo cyclization of enediynes^30,31,33^.

We then compared the 6-endo cyclization (*BC*) products of mono-Au(I), dual σ,π-Au(I), and parent *BC* pathways. For parent *BC*, a slight increase in ∠C1C2C3 and ∠C4C5C6 from 120° to 125° suggested higher p-character at C2 and C5 of 1,4-diradical intermediate (Fig. 6D). These out-of-plane diradicals were stabilized by a strong and symmetrical two-way interaction with adjacent σ-bonds (Fig. 6D). Similarly, the NBO charges, hybridization, and geometry at C2 and C5 of the σ-Au(I)-acetylide system (Fig. 6E) suggested the presence of a 1,4-diradical intermediate, as seen in the analogous parent *BC* product (Fig. 6D). In contrast to the parent *BC* and the σ-Au(I)-acetylide system, the dual σ,π-Au(I)-catalyzed *BC* activates the π(C5-C6) bond, leading to heterolytic cleavage and formation of zwitterions that are stabilized by two Au(I) ions. There is a noteworthy increase in the bond angle of the *sp*^*2*^-hybridized C2 carbon atom, which allows electron density to flow from σ(C1–C6) and σ(C3–C4) to carbocation at C2, thus stabilizing it (Fig. 6F). Although DFT studies point to the formation of an aromatic zwitterion [NICS(1): −12.7 ppm], Hashmi et al. carried out experiments that show a preference for a non-aromatic fulvene-type zwitterion generated via 5-endo cyclization (Fig. 2)^30,31^. In this context, the first study predicts that the mono σ-Au(I)acetylides do not affect the nature of the intermediate if used without second π-(C5-C6)-coordinated Au(I) (Figs. 6, 7). We thus propose a novel strategy based on a boron-Au(I) couple, which would enable boryl groups to activate π-(C5-C6) towards 6-endo ionic *BC*, and σ(C1–Au(I)) would offer stability to the β-carbocation at C2 (Fig. S4 in SI and Fig. 2).

### Boron-mediated Bergman cyclization

Conceptually, Bergman cyclization is considered an interrupted version of Cope rearrangement where a high energy 1,4-diradical intermediate interrupts a concerted pericyclic process (Fig. 2, left)^49,50^. In the present context, we predicted the boryl groups would activate the π(C5-C6) bond in enediynes, resulting in a *BC* cyclization analogous to the π-Au(I)-catalyzed *BC*^33^. For the model substrates, B3LYP and PBE0-computed activation energies strongly correlated with the electrophilicity of the boryl groups (Table S2 in SI and Fig. S2 in SI). The NBO charge analysis and the high activation energy (32 kcal/mol) for the substrates containing electron-rich -B(NH_2_)_2_ group indicated a diradical pathway (Table S2 in SI). In contrast, the -B(CN)_2_ group showed a zwitterionic trajectory and a low activation energy of 14.6 kcal/mol, similar to mono π-Au(I)-catalyzed BC systems^33^.

**Fig. 8:**
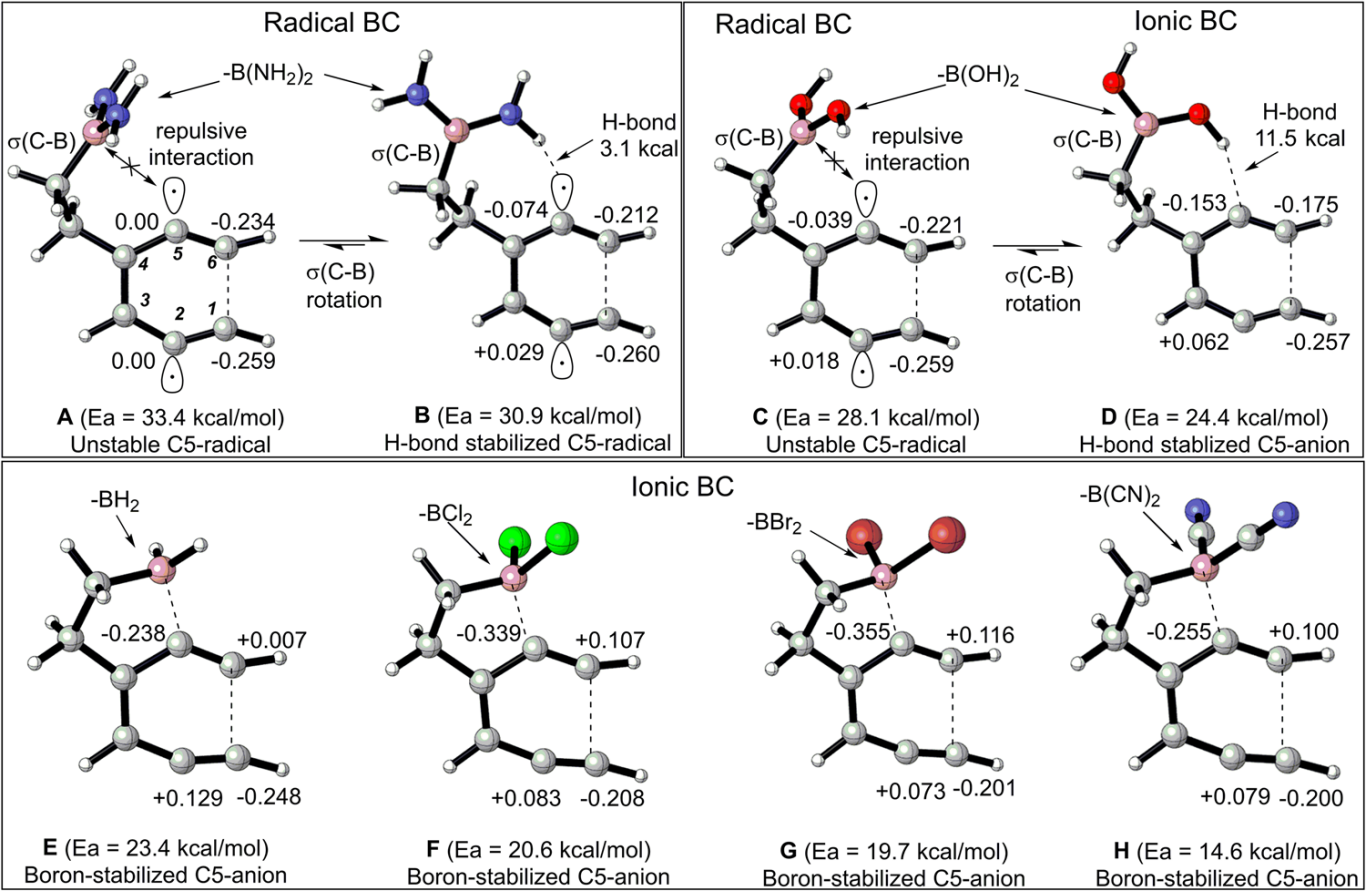
B3LYP/LANL2DZ-optimized transition state geometries of substrates containing electronically diverse boryl groups. Structures **A** and **B** correspond to non hydrogen bonding and hydrogen bonding transition states in -B(NH_2_)_2_ substituted substrates respectively, Structures **C** and **D** correspond to non hydrogen bond and hydrogen bonding transition states in -B(OH)_2_ substituted substrates respectively. Structures **E**, **F**, **G**, and **H** correspond to the transition states of substrates containing -BH_2_, -BCl_2_, -BBr_2_, and -B(CN)_2_ groups respectively. The values reflect the NBO charges at C1, C2, C5, and C6 carbons.

**Fig. 9:**
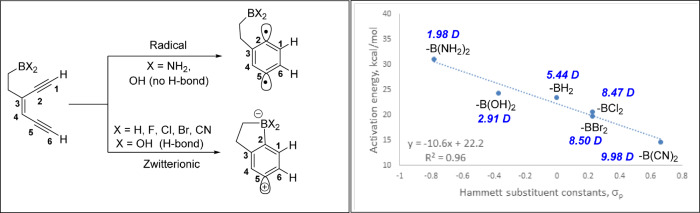
The scheme on the left show two possible outcomes when enediynes containing electronically diverse boryl groups undergo *BC*. The plot on the right shows the dependence of activation energy on the electrophilicity of boryl groups. Values in blue font indicate the net dipole moment of the TS.

A broader selection of substituents on the boryl groups showed a similar trend at the B3LYP/LANL2DZ level (Fig. 8). These substituents were selected based on Hammett’s constants, indicative of their ability to modify the electron density in the empty 2*p* orbital of the boron atom (See Fig. S3 in SI for the ESP maps). Based on the transition state geometries and NBO charges at C1, C2, C5, and C6, three main types of transition states were identified: (1) classic *BC*-type TS, (2) H bond stabilized TS, and (3) boron-stabilized TS. Substrates containing electron-rich B(NH_2_)_2_ and -B(OH)_2_ groups exhibited the classic *BC*-type TS with a high activation energy. The delocalization of non-bonding electrons on the amino and hydroxy groups partially filled the 2*p* orbital on boron and prevented it from interacting with the newly developed radical center at C5 (Fig. 8A, C). However, the rotation of the σ(C-B) bond enabled the amino and the hydroxy groups on boron to form a 3-center-3-electron (3c-3e) and 3-center-4-electron (3c-4e)-hydrogen bond with the intermediate at C5 respectively. The 3c–3e interaction of the C5 radical with the amino group is unique, as it forms a hydrogen bond instead of abstracting hydrogen atoms from the amino group. For a more efficient H bond donor, such as -OH on boron (Fig. 8C, D), we observed a mechanistic shift from a high-energy radical *BC*-type pathway (28.4 kcal/mol) to a lower-energy zwitterionic *BC* pathway (24.4 kcal/mol). As for the boron-stabilized TS, the NBO charges showed a developing zwitterion at C2 and C5, where the vacant 2*p* orbital on boron strongly interacted with the developing carbanionic center at C5 (Fig. 8E–H). The charges at C1 and C6 carbons further enabled the formation of a σ(C1–C6) bond and facilitated the flow of electron density from π(C1–C2) bond to vacant p(boron) via π*(C6–C5), making the TSs of these substrates more polar than their diradical counterparts (Fig. 8E–H). Additionally, Hammett’s constants of the substituents and dipole moment of the TS were negatively correlated with the activation barrier, indicating the role of electrophilic boron in shifting the reaction towards zwitterionic intermediates through a highly polar transition state (Fig. 9).

Overall, the boron-mediated *BC* approach is more reliable and efficient than the π-coordinated Au(I)-catalyzed method, as it did not show any undesired 5-endo cyclization in any of the three pathways discussed.

### Combining two pieces together: synergetic effect of the boryl group and σ-Au(I)-acetylide complex on the Bergman cyclization

**Fig. 10:**
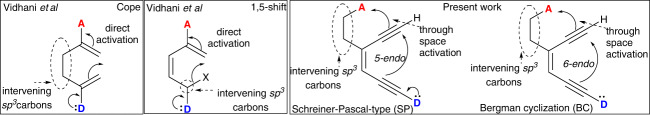
FLP-mediated Cope, 1,5-sigmatropic shift, and ionic-Bergman cyclization. Groups **A** and **D** correspond acceptor and donor, respectively.

The combination of electron-deficient boryl group and electron-rich σ-Au(I)-acetylide can form a frustrated Lewis pair (FLP) if placed remotely in the same enediyne. In such cases, the intervening *sp3*-hybridized methylene groups restrict the typical quenching of Lewis pairs, and Bergman cyclization provides an alternate route to share the electron density between them through σ-bonding, as illustrated in Fig. 10.

**Fig. 11:**
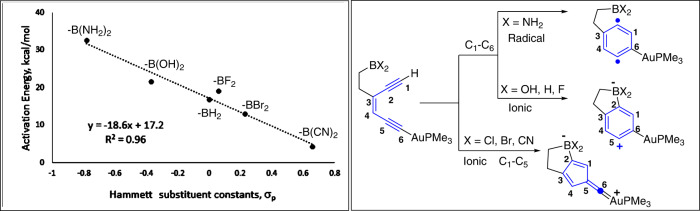
The scheme on the right show three possible outcomes when enediynes contain both electronically diverse boryl groups and Au(I)-acetylides. The plot on the left shows the dependence of activation energy on the electrophilicity of boryl groups when Au(I)-acetylide contains PMe_3_ ligand. All geometries were optimized at the B3LYP/LANL2DZ level.

This model is similar to the former study in which boron-mediated [3,3] and [1,5]-sigmatropic shifts helped to establish direct resonance-enabled communication between the remotely situated donor and acceptor groups (Fig. 10)^37,41^. Generally, enediynes prefer Bergman cyclization over the Schreiner–Pascal (*SP*) cyclization because the former produces a stable aromatic diradical, whereas the latter gives an unstable non-aromatic fulvene diradical^33,57^. The present study revealed an unexpected cyclization route in addition to the classic Bergman cyclization (*BC*) and Schreiner–Pascal (*SP*) pathways. This path has lower activation energy, applicable to boryl groups with Cl, Br, and CN substituents, and leads to non-aromatic fulvene zwitterions through ionic-*SP* [NICS(1): ~ −1.0 ppm]. In contrast, the presence of moderately electrophilic boryl groups [-BH_2_, -BF_2_, and -B(OH)_2_] at C5 led to 6-endo *BC* products through the ionic mechanism. The hydroxy group of -B(OH)_2_ was particularly noteworthy, as it formed a (3c-4e)-hydrogen bond with the anionic center at C5 and stabilized the transition state. Additionally, polar solvents stabilized the reactant more than the transition state, increasing activation energy (Fig. S9 in SI)^58–60^. Interestingly, the least electrophilic -B(NH_2_)_2_ generated 1,4-diradical through the classic *BC* (Fig. 11). This diradical had relatively low activation energies, the ability to form a (3c-3e)-hydrogen bond, and TS geometry comparable to the *BC* of the parent enediyne and its boron analogs (B and D, Fig. 8). Unlike -B(OH)_2_ system, the polar solvents only marginally increased the activation energy in the systems containing (3c-3e)-hydrogen bond. This was primarily due to greater stabilization of the reactant than the transition state, leading to a slight increase in activation energy when shifting from less polar to more polar solvents (Fig. S8 in SI).

**Fig. 12:**
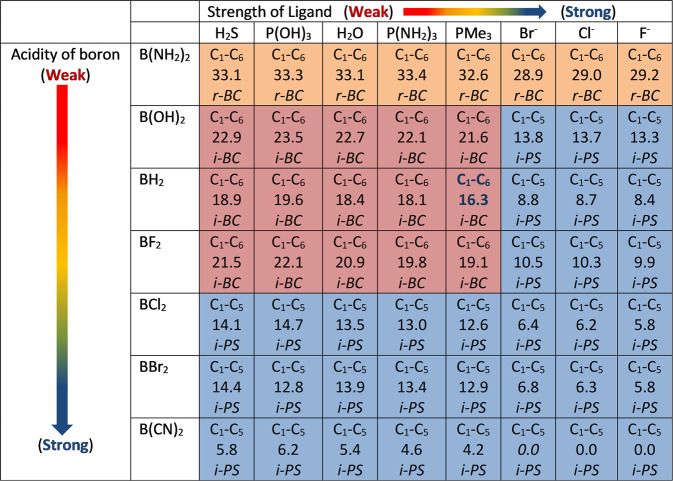
Comparative study of showing the mode of cyclization and the activation energy as a function of electrophilicity of boryl groups and the strength of ligand on Au(I). Calculations were performed at the B3LYP/LANL2DZ level, and the activation energy is expressed in kcal/mol. The terms i-*BC*, i-*PS*, and r-*BC* denote ionic-Bergman cyclization, ionic Schreiner–Pascal cyclization, and radical Bergman cyclization, respectively. The C_1_–C_6_ and C_1_–C_5_ represent 6-endo and 5-endo modes of cyclizations, respectively.

Bergman cyclization of a broader range of substrates showed a strong correlation between Hammett’s constants and the activation energies. Notably, the combination of -BX_2_ and Au(I)PMe_3_ revealed a steeper slope (−18.6, Fig. 11) compared to boryl substrates without the σ-Au(I)PMe_3_ (slope = −10.6, Fig. 9). This was unexpected, as the σ-Au(I)PMe_3_-acetylide had previously been inactive without boryl groups (Fig. 5). Consequently, the study of the combined effect of ligands on σ-Au(I) and substituents on the boryl groups revealed that, in general, ligands on Au(I) decreased the activation energy based on their strength, with anionic ligands having the most significant effect (7–10 kcal/mol, Fig. 12). The mechanism of cyclization was also affected, with -B(OH)_2_, -BH_2_, and -BF_2_ groups undergoing a change from ionic-Bergman cyclization (i-*BC*) to ionic-Schreiner–Pascal (i-*SP*) rearrangement when anions replaced the neutral ligands on Au(I) (Fig. 12). A similar mechanistic shift occurs when C5 contains highly electrophilic groups such as -BCl_2_, -BBr_2_, or -BCN_2_. The least electrophilic [-B(NH_2_)_2_] boryl group followed the r-*BC* pathway regardless of the ligand strength (Fig. 12).

**Fig. 13:**
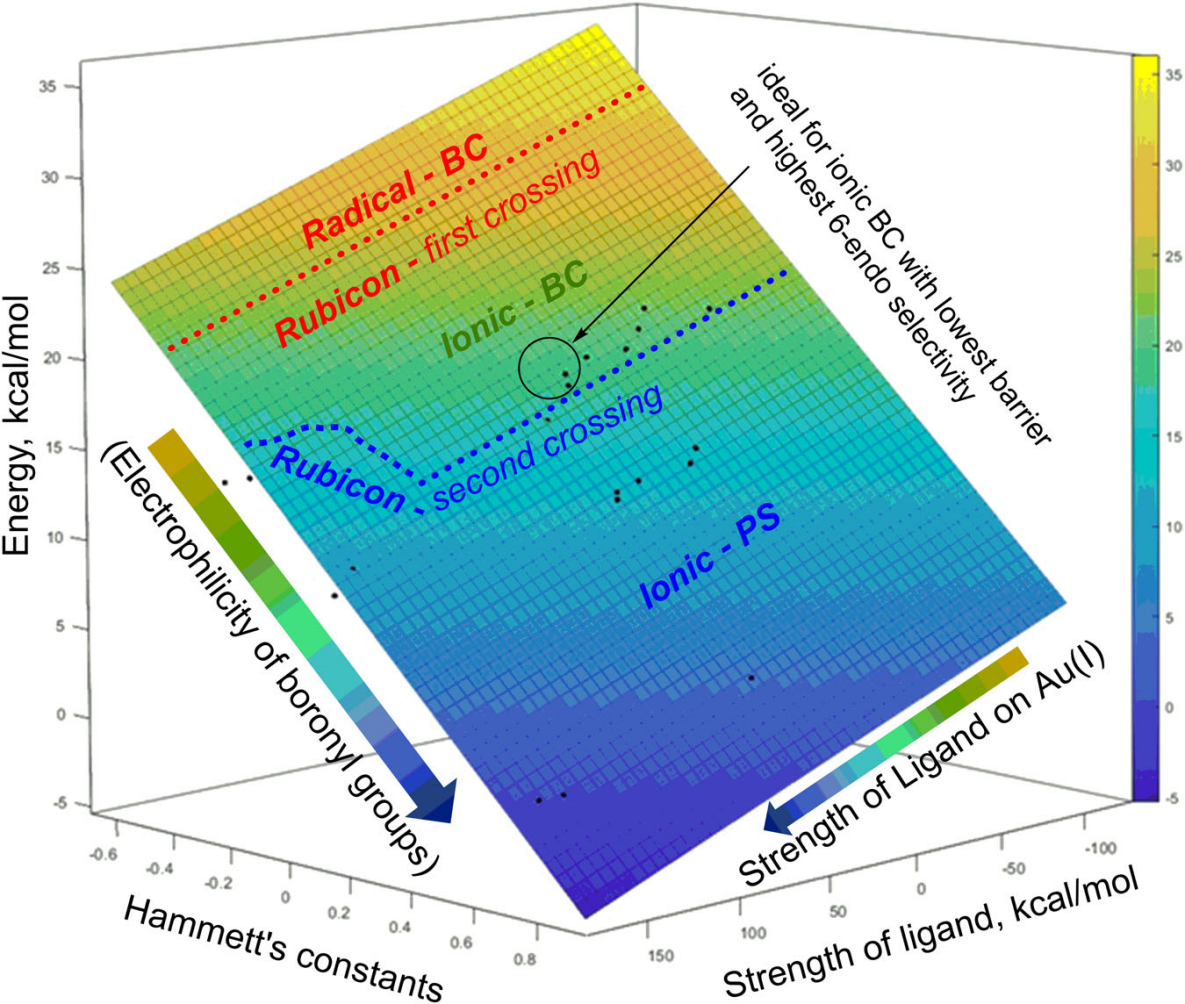
Multivariate plot with 55 data points showing the dependence of activation energy (Ea) on the strength of ligand on Au(I) and the electrophilicity of the boryl group. “Rubicon—first crossing” in red font denotes a mechanistic shift from *radical-BC*→*ionic-BC*. “Rubicon—second crossing” in blue font indicates a mechanistic shift from *ionic-BC*→*ionic-SP*.

The multivariate regression (*R*^2^ = 0.93) depicted in Fig. 13 shows a linear correlation between the activation energy (z-variable) and two variables—the electrophilicity of the boryl group (x-variable) and the strength of ligand on σ-Au(I)-acetylide (y-variable). The electrophilicity of the boryl group was assessed by analyzing Hammett’s constants of substituents attached to the boron atom, and the strength of the ligand was evaluated by comparing its binding affinity to Au(I) with that to water. The coefficients of the equation “*Ea* = 18.1 −18.9*x −* 0.042*y*”, obtained from fifty-five (55) substituted enediynes, showed the boryl groups had a larger impact on the activation energy than the ligands on Au(I). Furthermore, the multivariate plot revealed three distinct areas - r-*BC*, i-*BC*, and i-PS - delineated by the “Rubicon crossings,” corresponding to the significant mechanistic shifts. The first mechanistic shift, “Rubicon—first crossing” occurs when the least electrophilic -B(NH_2_)_2_ group is replaced by the slightly more electrophilic -B(OH)_2_, transforming r-*BC* into i-*BC*. The second shift, “Rubicon—second crossing,” highlights the influence of ligands on Au(I) and results in the transformation of i-*BC* into i-*SP*. All boryl groups, apart from -B(NH_2_)_2_, preferred the i-*SP* path when combined with anionic ligands (F, Cl, and Br) on Au(I). Moreover, highly electrophilic boryl groups also favor the i-*SP* pathway regardless of the ligand type present on Au(I). One of the goals of this study was to identify a suitable combination of the boryl group and the ligand on Au(I) that will: (1) initiate the i-*BC* pathway, (2) have low activation energy, and (3) avoid undesired i-*SP*. The combination of -BH_2_ and PMe_3_ groups, as depicted in Fig. 13, meets these criteria effectively and triggers the intended cascade.

### Cascade cyclizations: accessing TM-polyacene hybrid materials using *i-BC*

**Fig. 14:**
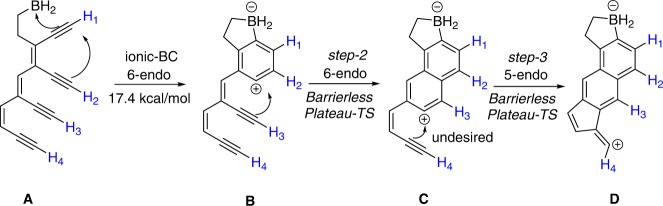
Boryl group-triggered Bergman cascade resulting into the terminal five-membered fulvene-type cation. Structures **A**, **B**, **C**, and **D** correspond to the polyenyne substrate, phenyl cation, naphthyl cation, and the final product of cascade cyclization respectively. Calculations were performed using the B3LYP/LANL2DZ level.

The Bergman cyclization of enediynes is a promising tool for generating carbon-rich materials such as carbon nanotubes and carbon-nano-onions^61,62^. However, its utility in polymer and material sciences is limited due to its inherent drawbacks, such as the self-quenching of diradicals, the high reactivity of diradicals, and the unwanted 5-endo cyclization leading to fulvene radicals causing large polydispersities^61^. In this regard, our investigation of *i-BC* triggered by the Au(I)PMe_3_/BH_2_ couple provides a viable alternative for initiating a defect-free polymerization (Figs. 12, 13). To evaluate its effectiveness, we chose polyenynes with the potential to give three consecutive aromatic rings. Initially, we studied the effect of -BH_2_ on the cascade reaction without Au(I)PMe_3_. In this situation, the -BH_2_ group initiated the i-*BC* reaction (17.4 kcal/mol, Fig. 14), creating two aromatic rings (B and C, Fig. 14). Surprisingly, the process ended with a 5-membered fulvene-type vinyl cation instead of a 6-membered aromatic ring (D, Fig. 14). In addition, the IRC analysis showed a notably exothermic cascade (−110 kcal/mol) in which steps 2 and 3 (B→C→D, Fig. 14) showed barrierless flat regions along the IRC. Mechanistically, these regions represent undefined intermediates B and C and their corresponding transition states (TS_B→C_ and TS_C→D_, Fig. S5 in SI).

**Fig. 15:**
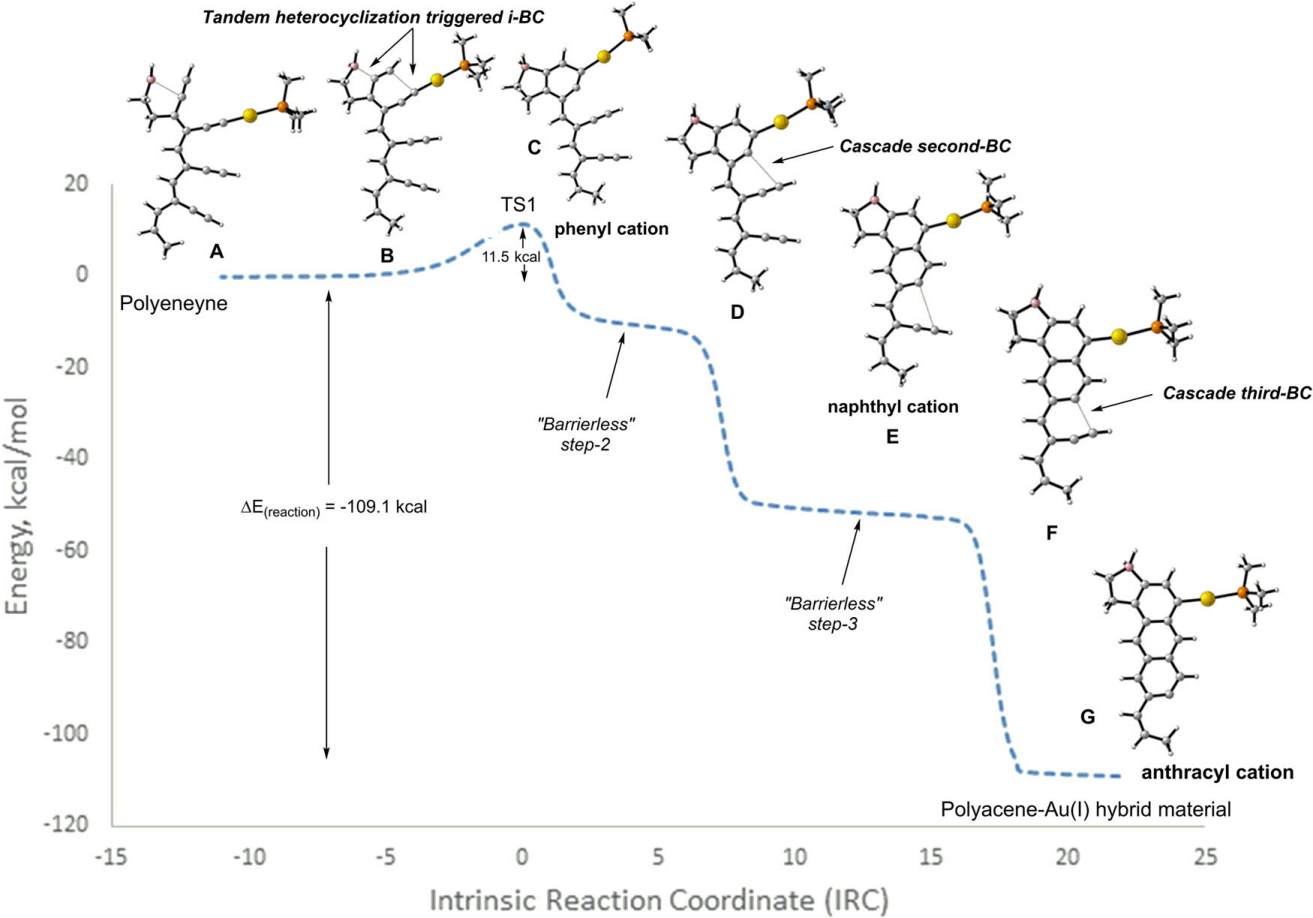
Au(I)/BH_2_ couple-triggered Bergman cascade resulting in an all-six-membered polyaromatic system. Structures **A**, **B** and **C** correspond to the acyclic polyenyne substrate, transition state and resulting phenyl cation from the ionic BC. Structures **D**, **E**, and **F** correspond to representative geometries in the barrierless regions of step-2 and step-3. Structure **G** corresponds to the anthracyl cation. Calculations were performed using the B3LYP/LANL2DZ level.

Remarkably, the utilization of BH_2_/Au(I)PMe_3_ couple in the polyenyne system yielded all six-membered rings with a low activation energy (Ea = 11.5 kcal/mol) and no undesired 5-endo cyclic unit (Fig. 15). The IRC analysis, once again, revealed an unusual trajectory along the PES, featuring barrierless steps for both the second and third cyclizations. The flat regions along the IRC indicate that the energies and geometries of the products of the first and second steps (C and E, Fig. 15) coalesce with that of the second and third transition states, respectively. The four-step cascade reaction generating an anthracene derivative is predicted to be highly exothermic (−109.1 kcal/mol), forming multiple aromatic rings contributing to the enormous reaction energy. This high reaction energy is consistent with the radical cascade process reported by ref. ^63^. Interestingly, polar solvents reduce the activation energy to 9.8 kcal/mol compared to the gas phase (Fig. S10 in SI). Additionally, the free energy surface in acetonitrile was also similar to that of the gas phase (Fig. S11 in SI).

**Fig. 16:**
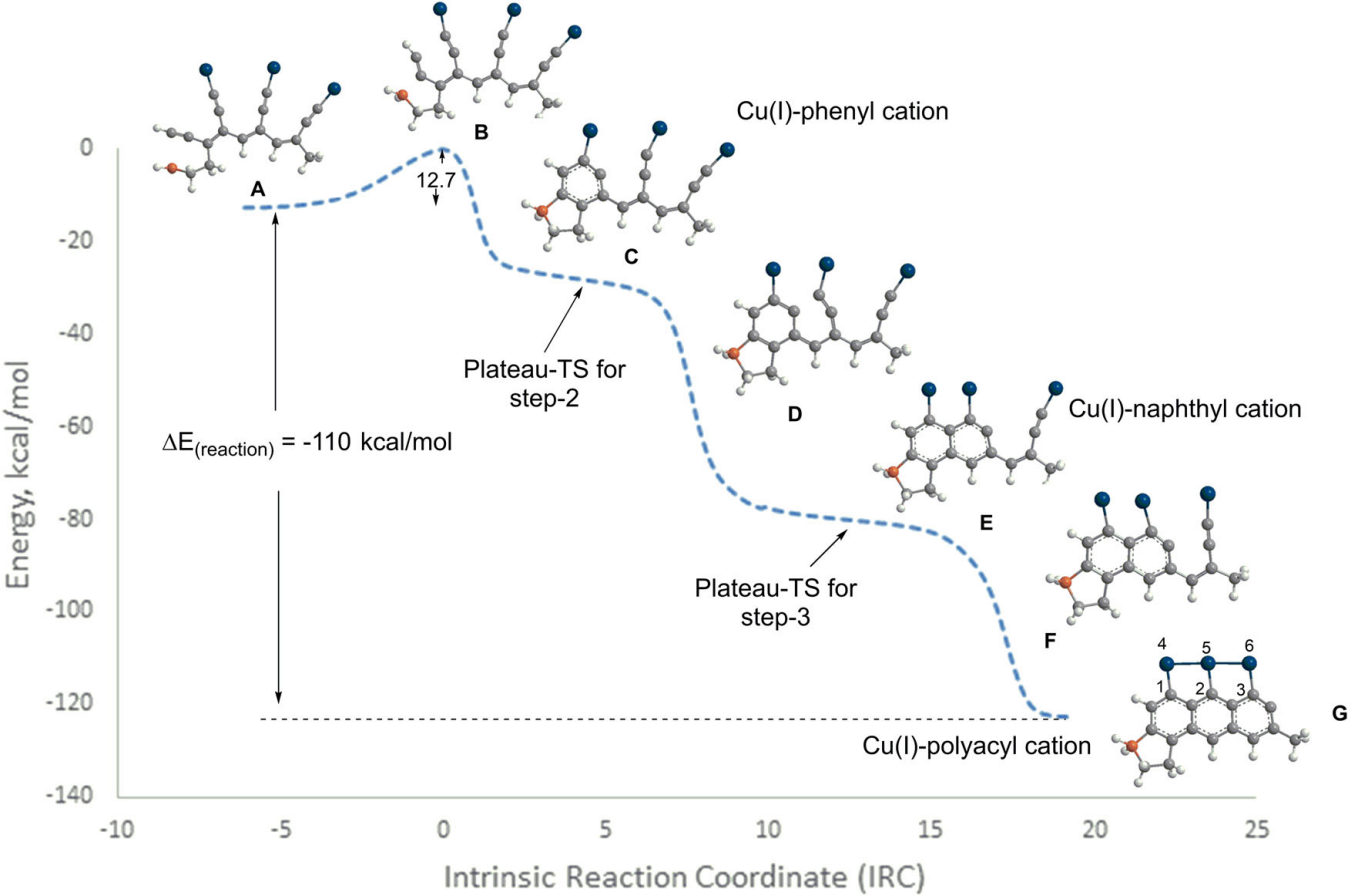
Cu(I)/BH_2_ couple-triggered Bergman cascade resulting in an all-six-membered polyaromatic system. Structures **A**, **B** and **C** correspond to the polyenyne acyclic substrate, transition state and resulting phenyl cation from the ionic BC. Structures **D**, **E**, and **F** correspond to representative geometries in the barrierless regions of step-2 and step-3. Structure **G** corresponds to the anthracyl cation. Calculations were performed using the B3LYP/LANL2DZ level.

Curiously, extending this approach to BH_2_/Cu(I) system produced two aromatic rings but terminated in an unwanted five-membered fulvene-type vinyl cation (Fig. S6 in SI). Replacing all hydrogens on alkynes by copper (I) produced the desired array of six-membered aromatic rings. The PES, low activation energy (12.7 kcal/mol), and a high exothermic effect (−110 kcal/mol) were also comparable with BH_2_/Au(I)PMe_3_ system (Fig. 16). The NBO interaction energies in poly-Cu(I) product G exhibited a significant degree of electron delocalization between the Cu(I) ions located in the peri-position and the polyacene framework, resulting in the Cu(I) ion distance being close to the FCC metallic bond distance of solid copper crystal (2.56 A) (Fig. 16). Since copper has excellent conducting properties, and the GNRs are exceptional semiconductors, we anticipate these novel hybrid materials to possess unique electrical properties^64^.

**Fig. 17:**
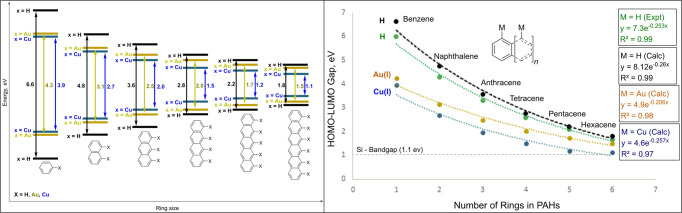
Comparing the bandgaps of unsubstituted and transition metal-substituted polyacenes. Left: Bandgaps as a function of the number of rings in unsubstituted, Au(I)-substituted, and Cu(I)-substituted polyacenes. Right: Exponentially declining bandgaps as a function of the number of delocalized p-bonds in unsubstituted, Au(I)-substituted, and Cu(I)-substituted polyacenes. The green and black plots on the right correspond to experimental and DFT-calculated bandgap, respectively. All calculations were performed using the B3LYP/LANL2DZ level.

Finally, we assessed the bandgaps and reorganization energies of the TM-polyacene hybrid material to evaluate its semiconducting characteristics. To validate the reliability of the B3LYP/LANL2DZ level, we compared the calculated HOMO-LUMO gap with the experimental data of the unsubstituted polyacenes (Fig. S7 in SI). The values for ≥4 rings agreed with experimental data, only differing by 0.1–0.2 eV (Fig. 17). When applied to molecules with a smaller dielectric constant, such as benzene and naphthalene, the B3LYP calculations with a 20% Hartree-Fock exchange differed from experimental values by 0.5 eV. Since we were interested in larger polyacenes, and the B3LYP-calculated trend agreed with the experimental results, we chose B3LYP to calculate the electronic properties of our Au(I) and Cu(I) hybrid systems^65^. The bandgap of unsubstituted polyenynes demonstrated an exponential decrease; however, the lowering is more substantial for metal-substituted polyacenes due to increased electron delocalization (Fig. 17). As reported by several groups, the transition metals, in these cases too, cause a more significant decrease in the energy of LUMO compared to the increase in the HOMO energy^66–70^. The comparison of the bandgaps in unsubstituted anthracene (3.6 eV), Au(I)-anthracene (2.6 eV), and Cu(I)-anthracene (2.0 eV) further highlights this effect (Fig. 17). Interestingly, the bandgaps in the latter two complexes were within the acceptable range of wide-bandgap semiconductors such as pentacene (2.5 eV) which is extensively used in UV/Visible LEDs, LASERS, and various electronic devices^71^. For the TM-polyacenes with four or more aromatic rings, the bandgap exponentially approaches the limit of silicon-based semiconductors (1.1 eV).

Alongside HOMO-LUMO energies and bandgaps, the reorganization energy associated with charge transport is an essential factor in evaluating the performance of semiconductors. According to the Marcus theory, the rate of charge transport is inversely proportional to the reorganization energy. For p-type semiconductors such as anthracene, tetracene, and pentacene, the four-point model equation (Eq. 1) predicted the hole transport reorganization energies $({\lambda }_{T}^{+})$ to decrease with the increasing number of aromatic rings (See Fig. S12 in SI)^72^. Additionally, the DFT-calculated values for the selected unsubstituted polyacenes were in excellent agreement with the experiments (Table 1)^73–75^. Interestingly, the low reorganization energies, ranging from 0.1 to 0.2 eV, were predicted for gold and copper derivatives (Table 1). This trend suggests that TM-polyacene hybrid materials with small bandgap and low reorganization energies could be promising candidates for further experimental study.

1 $${\lambda }_{T}^{\pm }={\lambda }_{1}^{\pm }+{\lambda }_{2}^{\pm }$$

$${\lambda }_{T}^{\pm }\,:{Hole}\,{or}\,{electron}\,{transport}\,{reorganization}\,{energies}$$

$${\lambda }_{1}^{\pm }:{E}_{({Neutral}\,{Geometry}\,{Vertical}\,{Ionization})}^{\pm }-{E}_{({Optimized}\,{Geometry}\,{of}\,{ion})}^{\pm }$$

$${\lambda }_{2}^{\pm }:{E}_{({Ion}\,{Geometry}\,{Vertical}\,{Neutralization})}^{0}-{E}_{({Neutral}\,{Optimized}\,{Geometry})}^{0}$$

**Table 1:** Hole transport reorganization energies (*λ*+) and bandgaps of selected polyacenes and their Au(I) and Cu(I) derivatives.

Compounds	Hole Transport λ_+_ (eV)	Bandgaps (eV)
Anthracene	0.135 (0.138)^73^	3.560
Tetracene	0.108 (0.111)^74^	2.762
Pentacene	0.045 (0.05)^75^	2.206
Anthracene-Cu	0.201	1.965
Tetracene-Cu	0.190	1.504
Pentacene-Cu	0.189	1.183
Anthracene-Au	0.169	2.473
Tetracene-Au	0.150	2.030
Pentacene-Au	0.139	1.724

Energies are expressed in electron volts (eV). Values in parenthesis correspond to experimentally determined reorganization energies. All calculations were performed at the B3LYP/LANL2DZ level.

## Concluding remarks

The present work provides a “bottom-up” substrate design to access TM-GNR hybrid semiconductors using zwitterionic Bergman cyclization-triggered cascade polymerization. These materials, exhibiting unusual optoelectronic properties, contain the thinnest semiconducting polyacene chain with a single-atom-thick metal wire. The first step towards accessing these materials was to design polyenynes that can undergo zwitterionic *BC* while avoiding unwanted side reactions. To this end, most boryl groups lowered the activation energy and changed the nature of the intermediate from the diradical to zwitterionic at the Bergman cyclization (*BC*) stage. Notably, electron-rich -B(NH_2_)_2_ adopted the classical *BC* pathway despite the unusual (3c-3e)-hydrogen bond stabilizing the transition state. For -B(OH)_2_, the hydroxy group forming a 3c-4e-hydrogen bond with C5 shifts the mechanism from a high-energy radical *BC* to a lower-energy zwitterionic pathway. Further, the remotely placed boryl groups caused an unexpected activation of σ-Au(I)-acetylides, emphasizing the role of ligands on the transition metals in regulating zwitterionic *BC*. Multivariate analysis revealed the right combination of boryl group and ligands on Au(I)-acetylide that generated a low-barrier (16.3 kcal/mol) zwitterionic-*BC* with high 6-endo selectivity near the “*Rubicon—Second Crossing*.” Thus, boron-Au(I)L catalysis had key benefits over σ,π-dual-Au(I) catalysis, namely the ability to transform radical-*BC* to zwitterionic-*BC*, provide a low activation energy pathway, and limit the undesired 5-endo *SP* rearrangement. This combination initiated the cascade polymerization *via* zwitterionic-*BC* followed by a series of unusual barrierless reactions where the formation of subsequent rings involved several “structureless transitions,” leading to the desired polyacenes. To the best of our knowledge, this would be the first set of examples where such consecutive multiple barrierless plateau reactions were captured in the single IRC calculations. Finally, the Au(I) and Cu(I)-substituted polyacenes show great potential for use as semiconducting materials due to their bandgaps and reorganization energies comparable to those of larger unsubstituted pentacene. These compounds would be one of the smallest single-molecule semiconductors and ideal candidates for further exploration.

## Methods

All geometries were optimized at the B3LYP/LANL2DZ levels, which frequently performs well for the transition metal compounds using Gaussian 03 program (see SI for reference)^76–78^. For comparison, B3LYP/6-311+G**/def2-TZVP and PBE0/6-311+G**/def2-TZVP calculations were performed for the *BC* of unsubstituted enediyne and σ-Au(I)-acetylide systems^33,37–40,45^. Furthermore, for comparison, B3LYP/6-311++G(d,p), PBE0/6-311++G(d,p), and PBE0/LANL2DZ calculations were performed for representative systems containing electronically diverse boryl groups. All IRC calculations were performed using B3LYP/LANL2DZ. Force Field calculation indicated that optimized structures were found to be true minima with no imaginary frequency. All energies have been expressed in hartrees and the frequencies in cm^−1^.

## Supplementary information

Supplementary Information

Description of Additional Supplementary File

Supplementary Data 1

## Data Availability

The authors declare that the data supporting the findings of this study are available within the paper [and its supplementary information files]. The cartesian coordinates of all species are deposited in Supplementary Data 1.
