# Supplementary material for: Zwitterionic Bergman cyclization triggered polymerization gives access to metal-graphene nanoribbons using a boron metal couple
Source: Commun Chem. 2023 Apr 7;6:66. doi: 10.1038/s42004-023-00866-w (PMC10082089; doi:10.1038/s42004-023-00866-w)
Supplement: Supplementary file 1 — Supplementary Information [file 42004_2023_866_MOESM1_ESM.pdf]

SUPPLEMENTARY INFORMATION

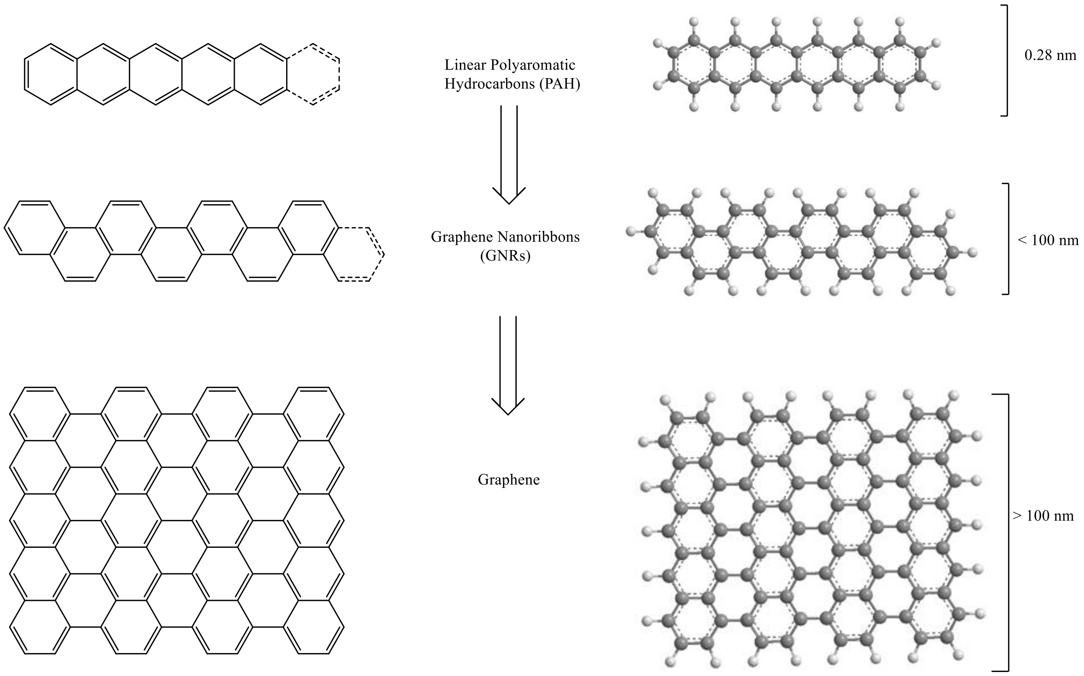

Figure S1. Broad classification of fused polyaromatic compounds based on the dimensions.

Gaussian 03, Revision E.01, M. J. Frisch, G. W. Trucks, H. B. Schlegel, G. E. Scuseria, M. A. Robb, J. R. Cheeseman, J. A. Montgomery, Jr., T. Vreven, K. N. Kudin, J. C. Burant, J. M. Millam, S. S. Iyengar, J. Tomasi, V. Barone, B. Mennucci, M. Cossi, G. Scalmani, N. Rega, G. A. Petersson, H. Nakatsuji, M. Hada, M. Ehara, K. Toyota, R. Fukuda, J. Hasegawa, M. Ishida, T. Nakajima, Y. Honda, O. Kitao, H. Nakai, M. Klene, X. Li, J. E. Knox, H. P. Hratchian, J. B. Cross, C. Adamo, J. Jaramillo, R. Gomperts, R. E. Stratmann, O. Yazyev, A. J. Austin, R. Cammi, C. Pomelli, J. W. Ochterski, P. Y. Ayala, K. Morokuma, G. A. Voth, P. Salvador, J. J. Dannenberg, V. G. Zakrzewski, S. Dapprich, A. D. Daniels, M. C. Strain, O. Farkas, D. K. Malick, A. D. Rabuck, K. Raghavachari, J. B. Foresman, J. V. Ortiz, Q. Cui, A. G. Baboul, S. Clifford, J. Cioslowski, B. B. Stefanov, G. Liu, A. Liashenko, P. Piskorz, I. Komaromi, R. L. Martin, D. J. Fox, T. Keith, M. A. Al-Laham, C. Y. Peng, A. Nanayakkara, M. Challacombe, P. M. W. Gill, B. Johnson, W. Chen, M. W. Wong, C. Gonzalez, and J. A. Pople, Gaussian, Inc., Wallingford, CT, 2003.

All geometries were optimized at the B3LYP/LANL2DZ levels which frequently performs well for the transition metal compounds (e.g., Soriano, E.; Marco-Contelles, J. *Acc. Chem. Res.* **2009**, *42*, 1026; Felix, R. J.; Xia, Y.; Dudnik, A. S.; Gevorgyan, V.; Li, Y. *J Am Chem Soc.* **2008**, *130*, 6940; and Weber, D.; Gutierrez, O.; Tantillo, D. J.; Gagné, M. R. *Nat. Chem*. **2012**, *4*, 405) using Gaussian 03 program (see reference). For comparison, B3LYP/6-311+G**/def2-TZVP and PBE0/6-311+G**/def2-TZVP calculations were performed for the *BC* of unsubstituted enediyne and σ-Au(I)-acetylide systems. Furthermore, for

comparison, B3LYP/6-311++G(d,p), PBE0/6-311++G(d,p), and PBE0/LANL2DZ calculations were performed for representative systems containing electronically diverse boronyl groups. All IRC calculations were performed using B3LYP/LANL2DZ. Force Field calculation indicated that optimized structures were found to be true minima with no imaginary frequency. All energies have been expressed in hartrees and the frequencies in cm^{-1}.

Table S1. Comparative study of activation energy in parent BC and Au(I)-acetylides using the two DFT-level methods. The values in parenthesis correspond to activation enthalpies.

Ligand	B3LYP/LANL2DZ (kcal/mol)	B3LYP/6-311+G**/def2-TZVP (kcal/mol)	PBE0/6-311+G**/def2-TZVP (kcal/mol)
Parent Cope	32.8 (31.4)	34.2 (32.7)	31.1 (29.6)
F^-	32.6	34.2	30.2
PMe_3	33.9	35.1	30.9
H_2O	34.2	35.6	31.1

Table S2. Comparative study of activation energy in parent BC and substrates containing boronyl groups using the three DFT-level methods. The values in parenthesis correspond to activation enthalpies.

Substituents on boron	B3LYP/ LANL2DZ (kcal/mol)	B3LYP/ 6-311++G** (kcal/mol)	PBE0/ 6-311++G** (kcal/mol)	PBE0/ LANL2DZ (kcal/mol)
Parent	32.8 (31.4)	34.2 (32.7)	31.1 (29.6)	30.9 (29.6)
$-B(NH_2)_2$	30.9	33.4	30.3	29.9
$-BH_2$	23.4	25.2	19.7	18.6
$-B(CN)_2$	14.6	16.1	16.0 (14.6)	10.3

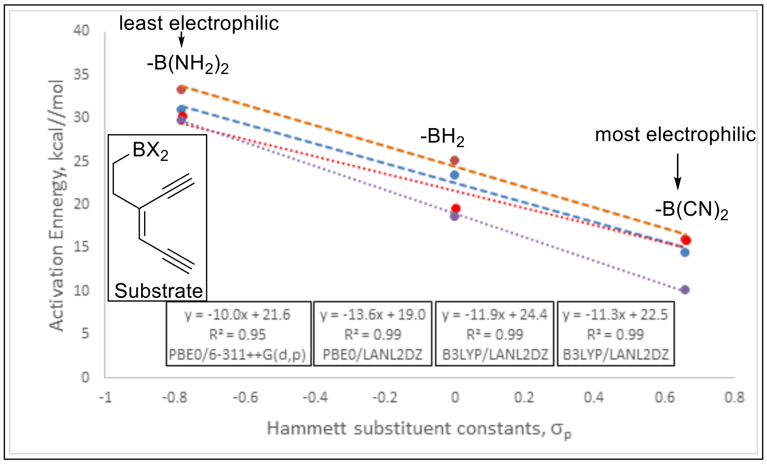

Figure S2. Comparing trends in activation energy for Bergman cyclization in boronyl-substrates using different DFT methods. Calculations were performed using B3LYP/LANL2DZ, B3LYP/6-311++G(d,p), PBE0/LANL2DZ, and PBE0/6-311++G(d,p) levels.

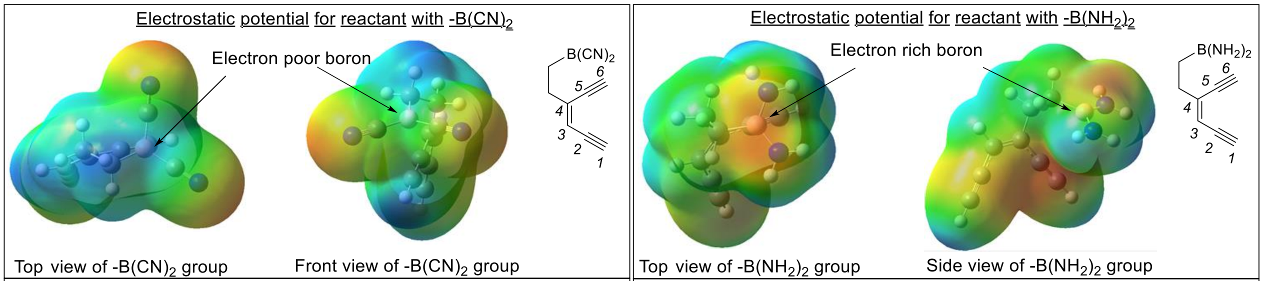

Figure S3. Electrostatic potential (ESP) diagram for two electronically opposite boronyl groups. Calculations were performed using B3LYP/LANL2DZ, B3LYP/6-311++G(d,p), PBE0/LANL2DZ, and PBE0/6-311++G(d,p) levels.

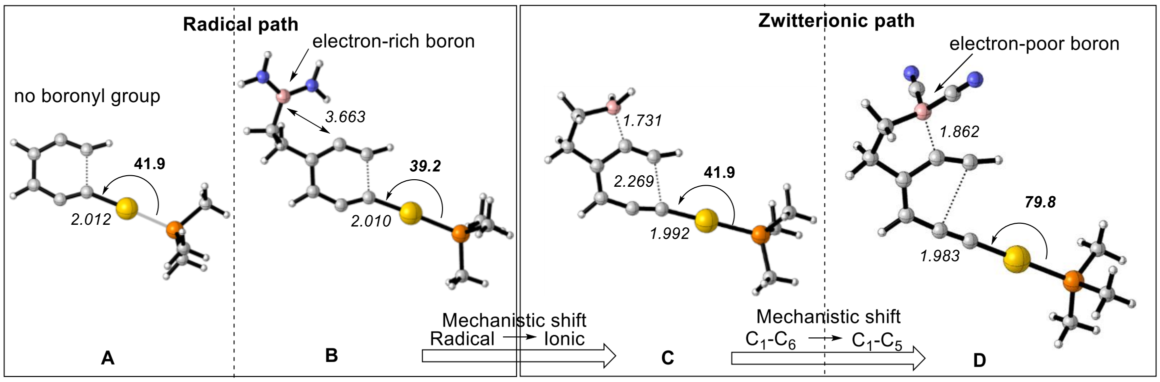

Figure S4. C5-Substituent-induced evolution of potential energy surface (PES) from radical-Bergman cyclization→H-bond-stabilized radical Bergman cyclization→ionic Bergman cyclization → ionic Schreiner-Pascal cyclization. Calculations were performed using B3LYP/LANL2DZ level.

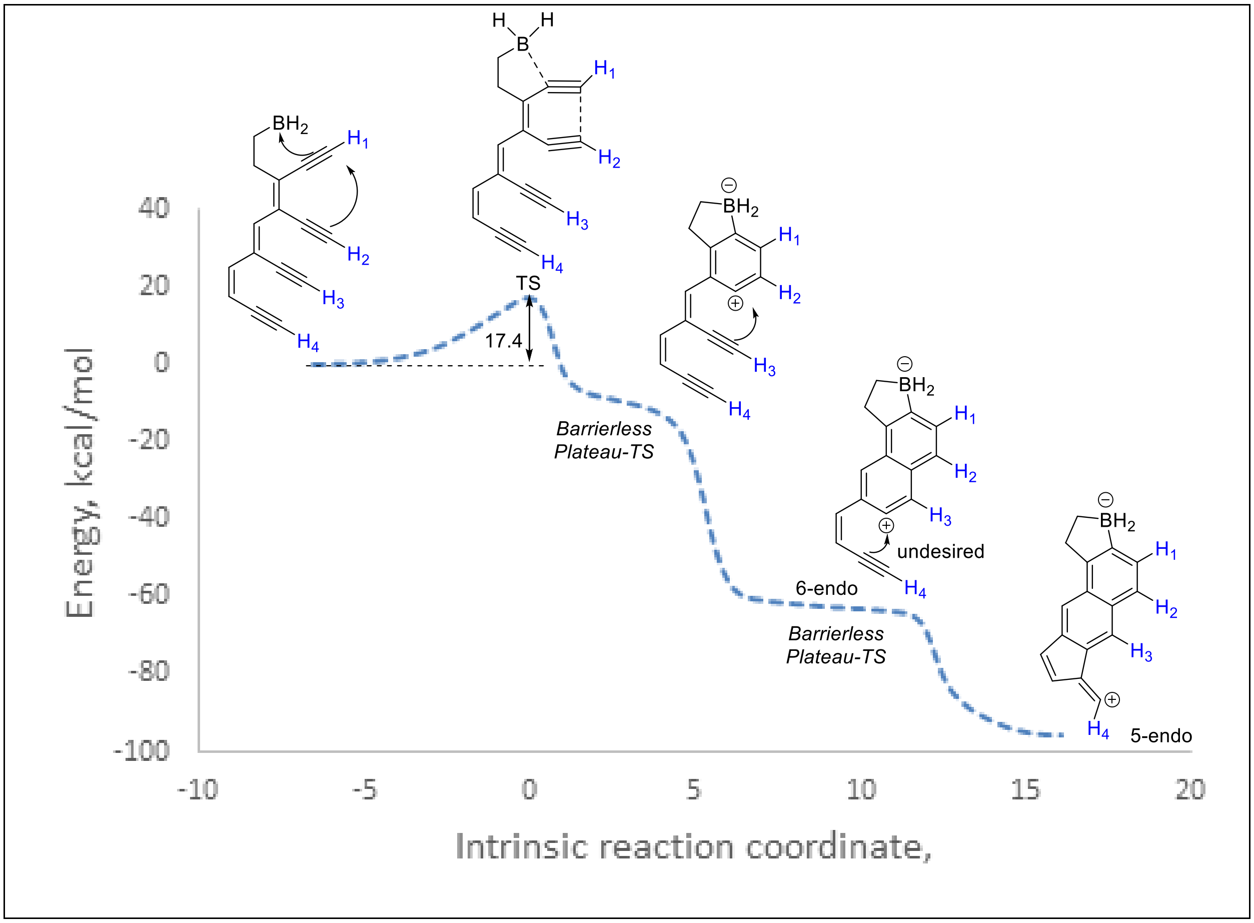

Figure S5. BH_2-triggered Bergman cascade resulting into a polyaromatic system with an undesired terminal 5-membered fulvene-type cation. Calculations were performed using the B3LYP/LANL2DZ level.

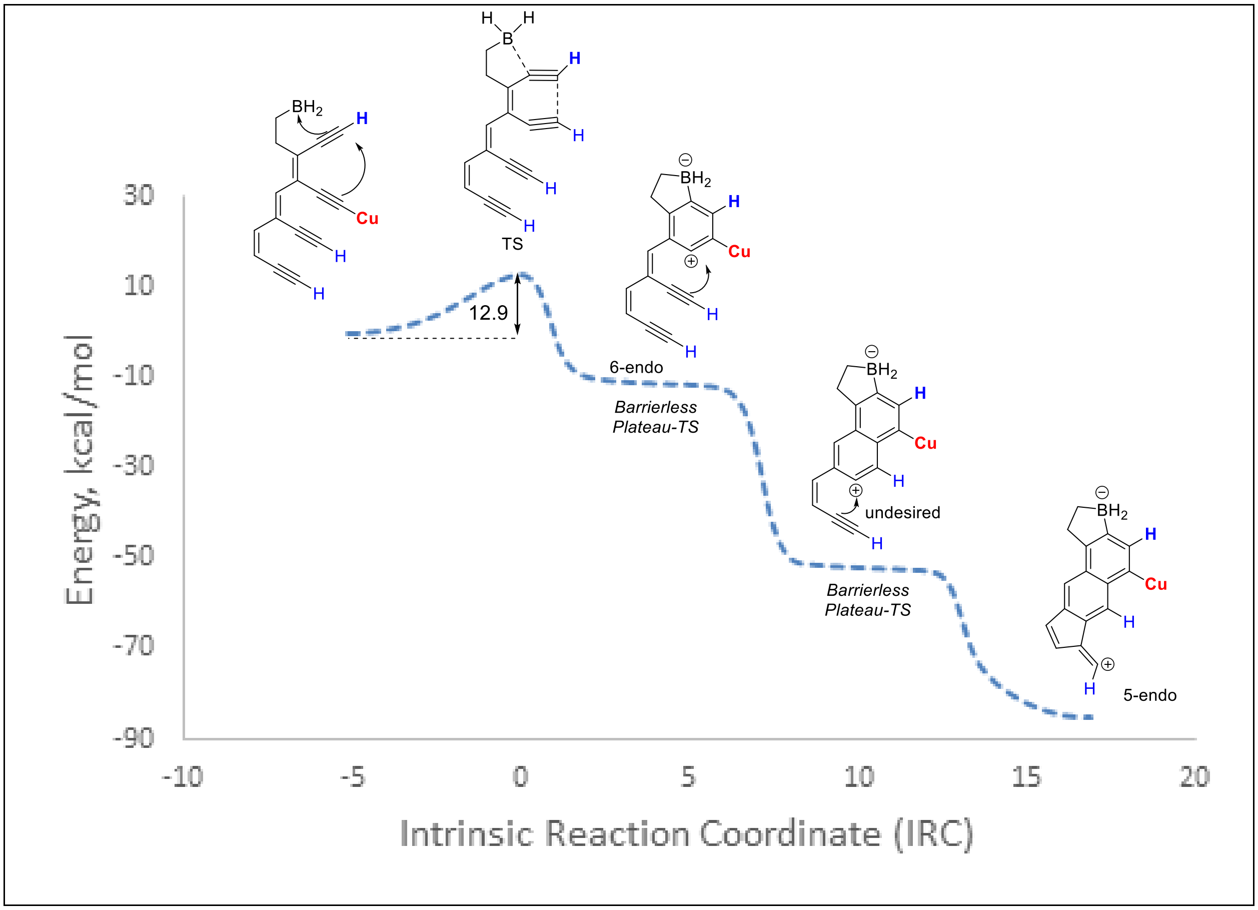

Figure S6. BH_2-Cu(I) couple-triggered Bergman cascade resulting into a polyaromatic system with an undesired terminal 5-membered fulvene-type cation. Calculations were performed using the B3LYP/LANL2DZ level.

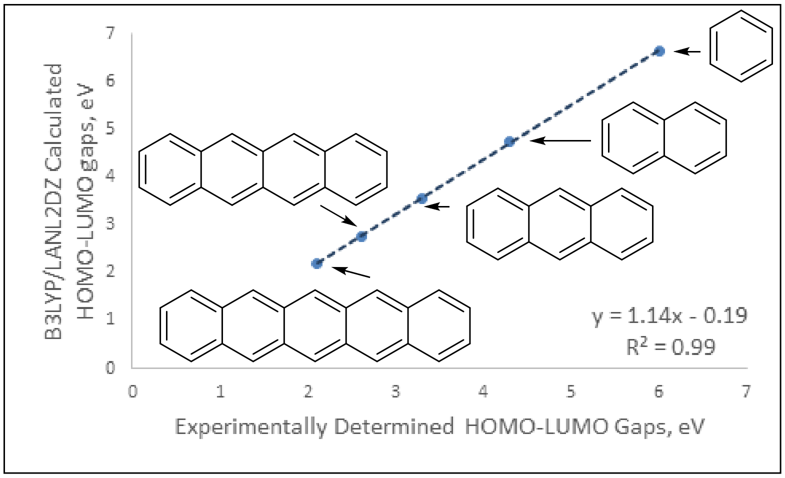

Figure S7. Correlation between experimentally determined bandgaps and DFT-calculated bandgaps in polyacenes.

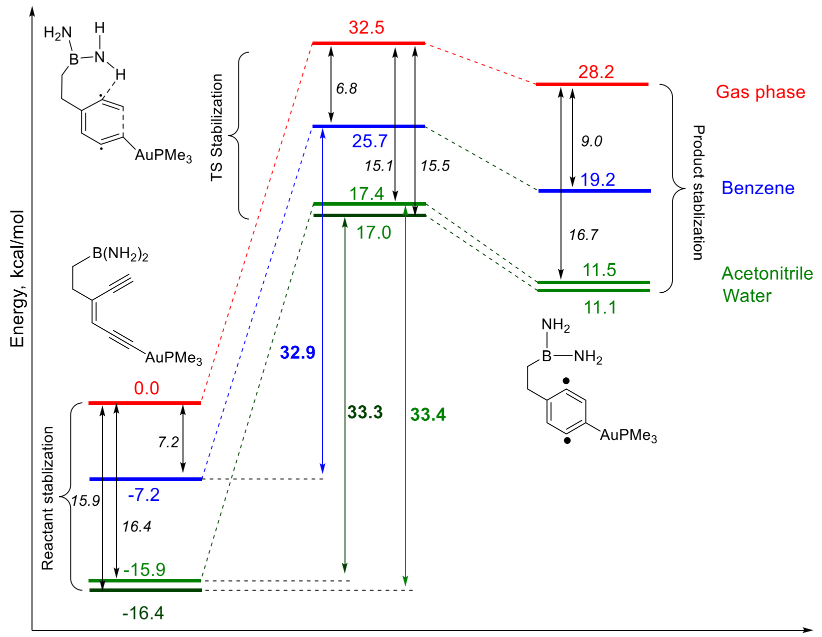

Figure S8. Effect of solvent polarity on the TS containing 3c-3e hydrogen bond. All calculations were performed using PCM-SCRF-B3LYP/LANL2DZ. Values in italics correspond to the extent of reactant, product, and TS stabilization in solvent vis-à-vis gas phase.

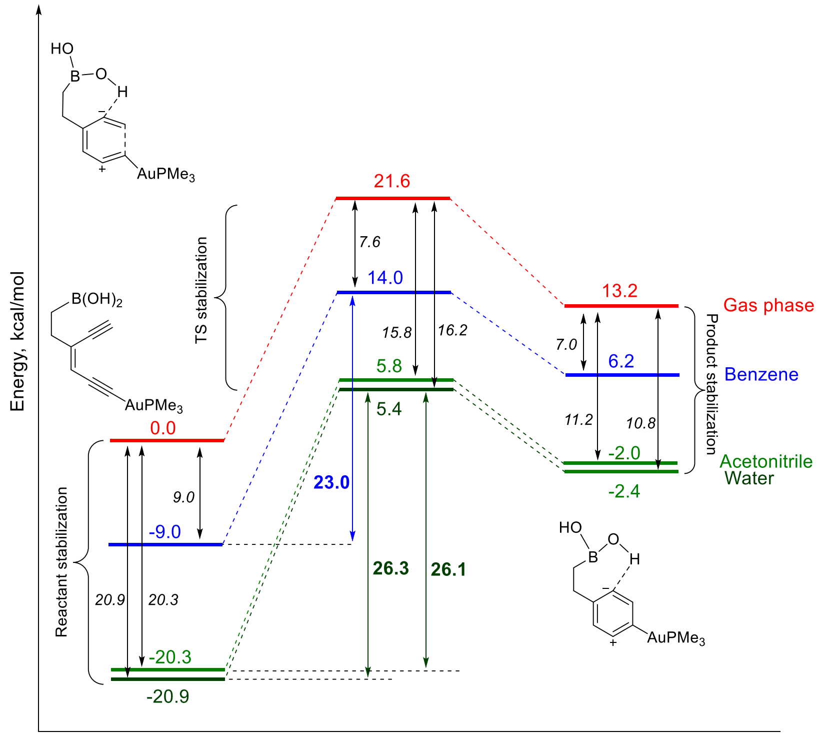

Figure S9. Effect of solvent polarity on the TS containing 3c-4e hydrogen bond. All calculations were performed using *PCM-SCRF-B3LYP/LANL2DZ*.

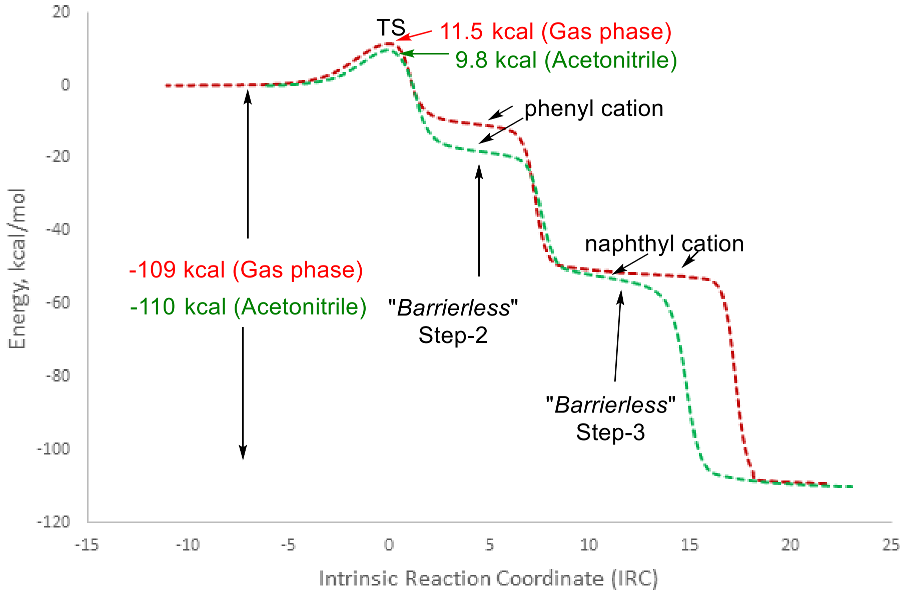

Figure S10. Au(I)/BH_2 couple-triggered Bergman cascade in gas phase and acetonitrile. Gas phase (red plot) and solvent (green plot) calculations were performed using the B3LYP/LANL2DZ and PCM-SCRF-B3LYP/LANL2DZ level respectively.

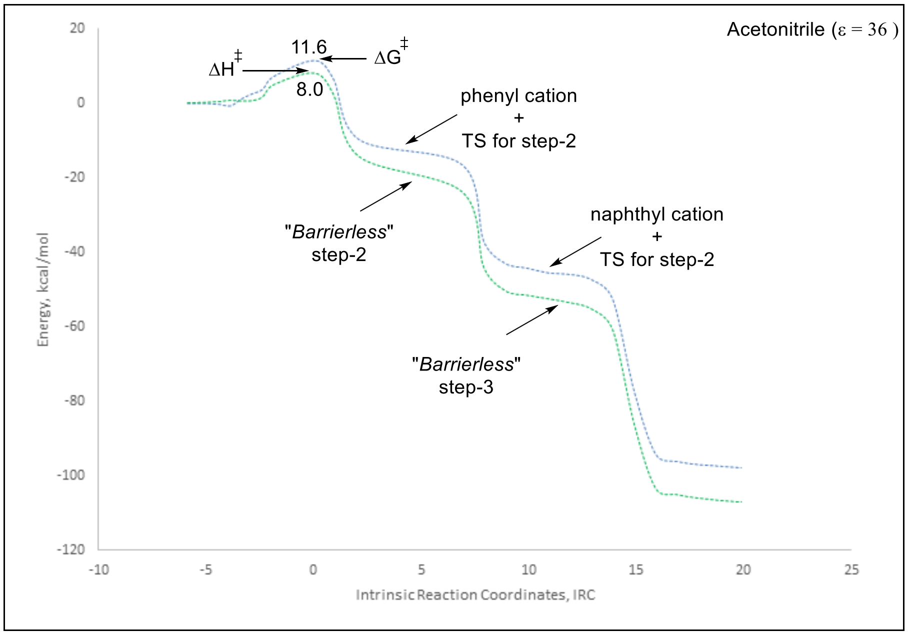

Figure S11. Plot in blue and green correspond to free energy and enthalpy change along the IRC for the Au(I)/BH_2 couple-triggered Bergman cascade in acetonitrile. Calculations were performed using the PCM-SCRF-B3LYP/LANL2DZ level.

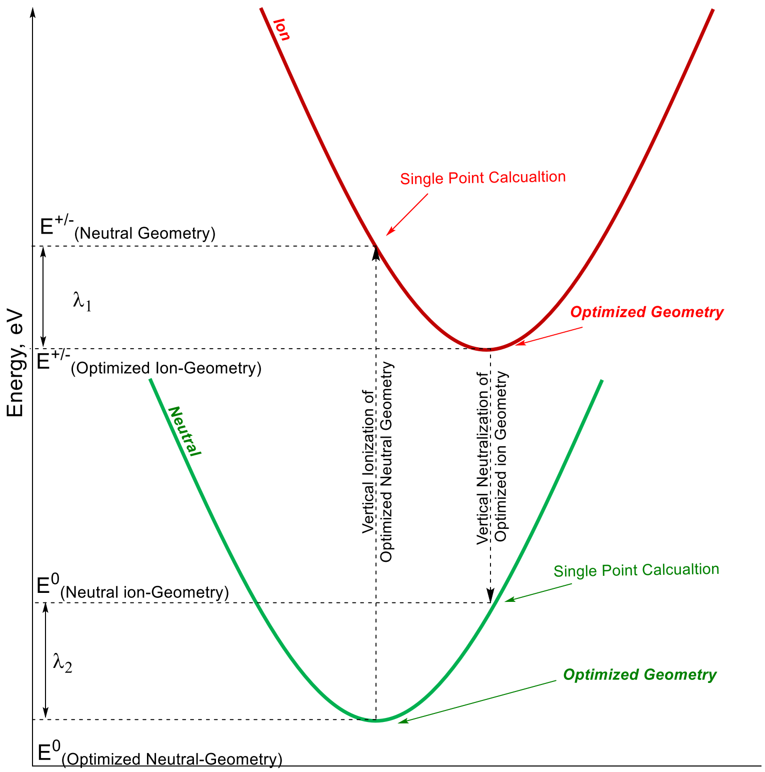

Figure S12. Schematic diagram of the four-point model depicting the above calculations.
