# Supplementary material for: Zwitterionic Bergman cyclization triggered polymerization gives access to metal-graphene nanoribbons using a boron metal couple
Source: Commun Chem. 2023 Apr 7;6:66. doi: 10.1038/s42004-023-00866-w (PMC10082089; doi:10.1038/s42004-023-00866-w)
Supplement: Supplementary file 2 — Description of Additional Supplementary File [file 42004_2023_866_MOESM2_ESM.pdf]

Description of Additional Supplementary File

File name: Supplementary Data 1

Description: Coordinates, total energies, enthalpies, free energies, and ZPE corrected energies of 4 geometries optimized using DFT level theory
