# Supplementary material for: Zwitterionic Bergman cyclization triggered polymerization gives access to metal-graphene nanoribbons using a boron metal couple
Source: Commun Chem. 2023 Apr 7;6:66. doi: 10.1038/s42004-023-00866-w (PMC10082089; doi:10.1038/s42004-023-00866-w)
Supplement: Supplementary file 3 — Supplementary Data 1 [file 42004_2023_866_MOESM3_ESM.docx]

Supplementary Data 1

*Table S3.*  Coordinates, total energies, enthalpies, free energies, and ZPE corrected energies of geometries optimized using DFT level theory.

|   B3LYP/LANL2DZ  B3LYP/ 6-311++g(d,p)  PBE0/ 6-311++g(d,p)  PBE0/ LANL2DZ | Total Energy: -445.7155713  Enthalpy: -445.523737  Free energy: -445.577877  ZPE Correction: -445.537443  C 1.95610400 0.95113900 0.07555000  C 0.82030100 0.37129700 0.57761600  C 0.69695000 -1.05149200 0.70795900  C 3.11734200 0.26042600 -0.38473000  C 0.57520200 -2.26144100 0.85785300  H 0.47104500 -3.31582700 0.97655600  C 4.13719300 -0.28842500 -0.78924800  H 5.01835100 -0.77900000 -1.13550700  C -0.36339200 1.23769700 0.99924200  H -0.82933100 0.80597900 1.89496500  H 0.01642400 2.23114700 1.27645000  C -1.44195800 1.40157800 -0.12164100  H -2.13666600 2.19275600 0.20636500  H -0.94471300 1.78781400 -1.02403800  B -2.30361800 0.09972700 -0.46861700  H 1.98979600 2.03997600 0.01896100  N -2.21435500 -0.48029600 -1.76908100  H -2.73338800 -1.29860700 -2.06280400  H -1.56683200 -0.12683000 -2.46051200  N -3.17873900 -0.44197800 0.52392300  H -3.26315500 -0.02996700 1.44350900  H -3.73823900 -1.27483400 0.38964500  Total Energy: -445.9072767  Enthalpy: -445.717801  Free energy: -445.772873  ZPE Correction: -445.732003  C 1.99176800 -0.93987600 -0.03619400  C 0.84653400 -0.40012500 -0.52249500  C 0.70235600 1.00490000 -0.68843000  C 3.13795700 -0.21980700 0.37415600  C 0.55939100 2.18905100 -0.86447300  H 0.43902300 3.23463800 -1.00842800  C 4.13758600 0.35242500 0.73371700  H 5.01065200 0.87091600 1.04589000  C -0.32481200 -1.28657200 -0.90359900  H -0.75226300 -0.91873300 -1.84183200  H 0.06652800 -2.28826200 -1.10504500  C -1.43094300 -1.37859300 0.18204200  H -2.10605200 -2.19150400 -0.11809100  H -0.96575800 -1.71323900 1.11637900  B -2.32328600 -0.07876100 0.43279900  H 2.04934300 -2.02186100 0.05108600  N -2.30945700 0.54972900 1.70529200  H -2.84156100 1.37381600 1.93612500  H -1.67411400 0.26845600 2.43276300  N -3.17350000 0.39950400 -0.60217600  H -3.21171800 -0.02555300 -1.51308000  H -3.73597900 1.23208800 -0.52992700  Total Energy: -445.3425323  Enthalpy: -445.151980  Free energy: -445.207120  ZPE Correction: -445.166213  C 2.09238400 -0.93009200 0.00292000  C 0.88513700 -0.46097700 -0.38777700  C 0.65884000 0.93161600 -0.54693000  C 3.22220000 -0.13522100 0.29475300  C 0.44568300 2.10794700 -0.70227100  H 0.26078300 3.14786400 -0.83118600  C 4.21058300 0.50753400 0.55125400  H 5.07433400 1.08766400 0.77428200  C -0.26182800 -1.40081000 -0.65745400  H -0.65479100 -1.19189200 -1.66022700  H 0.14270700 -2.41771100 -0.68649000  C -1.39232400 -1.31455700 0.38325200  H -2.02837300 -2.20108400 0.24901800  H -0.95418700 -1.41922200 1.38312200  B -2.36139000 -0.05323700 0.32115700  H 2.21819000 -2.00590400 0.10322100  N -2.64562800 0.66447300 1.50866800  H -3.27405800 1.44829000 1.56013700  H -2.16719700 0.47542300 2.37137600  N -3.00576700 0.28157000 -0.89758000  H -2.80677900 -0.19106000 -1.76122900  H -3.60795700 1.07887400 -1.01190800  Total Energy: -445.1667034  Enthalpy: -444.973104  Free energy: -445.027326  ZPE Correction: -444.986738  C 1.96051500 -0.95602600 -0.04145600  C 0.81074500 -0.41721300 -0.54347800  C 0.66316900 0.99385700 -0.72137700  C 3.11003600 -0.22410200 0.36873400  C 0.52417000 2.19427500 -0.90847200  H 0.40299600 3.24256800 -1.06131800  C 4.11888900 0.36816900 0.72966800  H 4.99258000 0.89595900 1.03858200  C -0.35975800 -1.30914700 -0.91165600  H -0.82171100 -0.93713400 -1.83519300  H 0.02482900 -2.31569700 -1.12366600  C -1.43251200 -1.39620500 0.20741100  H -2.13493500 -2.19919600 -0.06704800  H -0.94173500 -1.72375000 1.13499900  B -2.28017200 -0.06976100 0.46129000  H 2.01600800 -2.04013300 0.05627600  N -2.19404300 0.59036200 1.71913900  H -2.70597600 1.42840800 1.95484700  H -1.55187000 0.27667900 2.43095500  N -3.14043500 0.41069000 -0.57012200  H -3.22064400 -0.06316900 -1.45706100  H -3.68886400 1.25526100 -0.49717900 |
| --- | --- |
|   B3LYP/LANL2DZ  B3LYP/ 6-311++g(d,p) | Total Energy: -485.4630941  Enthalpy: -485.297990  Free energy: -485.350626  ZPE Correction: -485.311190  C 2.14654600 0.93039200 0.02028700  C 0.88888800 0.46496500 0.30051900  C 0.61797000 -0.94295100 0.37624300  C 3.30211800 0.12692700 -0.22030800  C 0.37844400 -2.14243000 0.45009600  H 0.19219700 -3.19049500 0.51022300  C 4.32116300 -0.52217500 -0.43197800  H 5.19998400 -1.09801300 -0.61427000  C -0.26573400 1.43228000 0.53742600  H -0.67750900 1.27185800 1.54293900  H 0.13030800 2.45547400 0.50115600  C -1.42197600 1.28213800 -0.49338500  H -2.04324800 2.19118000 -0.42846100  H -1.02937300 1.24592700 -1.51694300  B -2.38713800 0.06570900 -0.23904600  H 2.29410400 2.00972400 -0.02469100  O -2.86963700 -0.64043100 -1.33257100  H -3.48778500 -1.38794000 -1.25082700  O -2.76716800 -0.19379800 1.07218000  H -3.35306600 -0.92730500 1.32583200  Total Energy: -485.6762861  Enthalpy: -485.511771  Free energy: -485.564677  ZPE Correction: -485.525086  C 2.14470800 -0.91985600 -0.01981900  C 0.90586500 -0.45129700 -0.30965800  C 0.64839600 0.94647700 -0.38387500  C 3.29090300 -0.12952000 0.23120000  C 0.40671800 2.12586700 -0.45257100  H 0.21671700 3.16953100 -0.51044100  C 4.29444700 0.50315800 0.45077600  H 5.16948500 1.07411200 0.64292800  C -0.24883300 -1.40071100 -0.56111700  H -0.63916700 -1.22507900 -1.56907500  H 0.14874500 -2.41886500 -0.54309700  C -1.40376400 -1.26689500 0.46029300  H -2.01596600 -2.17710200 0.38542700  H -1.00996200 -1.25669500 1.48052400  B -2.39899300 -0.06419900 0.24015700  H 2.28635800 -1.99633200 0.02530800  O -2.87762400 0.60031600 1.34182100  H -3.52271700 1.29285800 1.16996600  O -2.80464100 0.20829300 -1.04441500  H -3.41103900 0.94636800 -1.15293800 |
|   B3LYP/LANL2DZ  B3LYP/ 6-311++g(d,p)  PBE0/ 6-311++g(d,p)  PBE0/ LANL2DZ | Total Energy: -334.8904651  Enthalpy: -334.740458  Free energy: -334.786738  ZPE Correction: -334.751482  C 1.36031500 -0.99253100 -0.06682400  C 0.11546300 -0.44211500 -0.19696200  C -0.10960800 0.97454200 -0.17133700  C 2.58025200 -0.26742100 0.08876100  C -0.40236700 2.16630500 -0.14361200  H -0.61992800 3.21052900 -0.12646600  C 3.65049900 0.31569100 0.22522400  H 4.57552600 0.83341000 0.34291700  C -1.13898000 -1.29560300 -0.37487300  H -1.44696000 -1.25475300 -1.43109600  H -0.90464500 -2.34362600 -0.14446500  C -2.30351900 -0.76553800 0.50284500  H -3.17753900 -1.42932400 0.34985000  H -2.04339600 -0.83829200 1.56685600  B -2.82311600 0.67287100 0.09642000  H -3.20068200 1.45384600 0.92091500  H -3.02270700 0.92341200 -1.05725000  H 1.44358600 -2.07953800 -0.08269500  Total Energy: -335.0396449  Enthalpy: -334.890807  Free energy: -334.937132  ZPE Correction: -334.901884  C 1.35947300 -0.98319300 -0.06002200  C 0.12298300 -0.44947300 -0.19311600  C -0.10995700 0.95349800 -0.17018300  C 2.56045300 -0.25037200 0.08617200  C -0.42543600 2.11937500 -0.13438900  H -0.65525300 3.15714500 -0.11437700  C 3.60627700 0.33701000 0.21392400  H 4.52078600 0.86652400 0.32480800  C -1.12519800 -1.29815100 -0.36008600  H -1.41998100 -1.27163300 -1.41669400  H -0.88618100 -2.33739300 -0.11715000  C -2.28035800 -0.74821800 0.49816300  H -3.15976300 -1.39977200 0.35664300  H -2.02395800 -0.81662100 1.56040800  B -2.78476700 0.68152100 0.07650300  H -3.17545600 1.46522900 0.88723900  H -2.97806200 0.91189500 -1.07948700  H 1.45227700 -2.06583500 -0.06667800  Total Energy: -334.600106  Enthalpy: -334.450161  Free energy: -334.495476  ZPE Correction: -334.460846  C 1.38422200 -1.00295800 -0.04820600  C 0.11622500 -0.54985000 -0.12503700  C -0.20754200 0.83337500 -0.08754000  C 2.53024400 -0.18427700 0.04332400  C -0.62181700 1.97499800 -0.03655000  H -0.83083300 3.01949800 -0.01171400  C 3.52758900 0.48951600 0.12396100  H 4.40302500 1.09096900 0.19441900  C -1.12073000 -1.40795600 -0.22938900  H -1.35507000 -1.53283300 -1.29527500  H -0.92743100 -2.40425600 0.18144000  C -2.25929100 -0.65230300 0.45447700  H -3.21321400 -1.16990600 0.28713600  H -2.09638000 -0.64380700 1.53878500  B -2.41763000 0.82605400 -0.13370200  H -2.92563600 1.65458000 0.57734200  H -2.60620400 0.93026100 -1.31780200  H 1.54649500 -2.07805500 -0.05606100  Total Energy: -334.4633161  Enthalpy: -334.311908  Free energy: -334.357292  ZPE Correction: -334.322601  C 1.39111600 -0.99422700 -0.05573000  C 0.12222900 -0.50985100 -0.14094800  C -0.18362400 0.88707600 -0.10768200  C 2.56909900 -0.20326100 0.05352600  C -0.58316500 2.04855000 -0.06885200  H -0.84160800 3.08376500 -0.04785800  C 3.60195400 0.44621200 0.14964000  H 4.49773000 1.01953500 0.23265300  C -1.11534500 -1.38212000 -0.26897200  H -1.37273500 -1.46643500 -1.33543600  H -0.90524400 -2.39493600 0.09736600  C -2.28009700 -0.70483800 0.47462200  H -3.20624900 -1.28150200 0.31045600  H -2.09477800 -0.71020800 1.55634600  B -2.58734600 0.76013600 -0.07361000  H -3.04792500 1.58876200 0.66718400  H -2.75376200 0.90980700 -1.25526600  H 1.52829900 -2.07471700 -0.07102700 |
|   B3LYP/LANL2DZ | Total Energy: -533.5368263  Enthalpy: -533.396282  Free energy: -533.447887  ZPE Correction: -533.408936  C 2.17979600 0.91516200 0.00439800  C 0.88554300 0.51947400 0.20406400  C 0.51030900 -0.86282900 0.28006400  C 3.30323100 0.04623300 -0.13707000  C 0.12637800 -2.02529500 0.34146500  H -0.19563300 -3.04101000 0.39695200  C 4.29509800 -0.66344500 -0.26386200  H 5.15053700 -1.29138700 -0.37187700  C -0.24046200 1.53819500 0.35589300  H -0.59992800 1.52821300 1.39502700  H 0.15333800 2.54238400 0.15815800  C -1.44286800 1.25094700 -0.58813900  H -2.11888100 2.12249600 -0.54349800  H -1.11376800 1.16107900 -1.63031600  B -2.30316900 0.01718300 -0.17736300  H 2.38738700 1.98416900 -0.05153300  F -2.84870900 -0.85420900 -1.08495600  F -2.69010900 -0.19051500 1.12418100 |
|   B3LYP/LANL2DZ | Total Energy: -363.6799068  Enthalpy: -363.541716  Free energy: -363.595890  ZPE Correction: -363.555029  C -2.79237400 -0.76328100 -0.41150200  C -1.49010100 -0.57627500 -0.03386300  C -1.06792900 0.58659200 0.69111100  C -3.87398000 0.12727500 -0.13927000  C -0.66276500 1.56600700 1.30628500  H -0.31912400 2.42147600 1.84314800  C -4.83132600 0.86355800 0.07274000  H -5.65629600 1.51201700 0.26421700  C -0.41923100 -1.60825600 -0.37240700  H -0.04984100 -2.07264000 0.55238100  H -0.87103800 -2.40469000 -0.97632800  C 0.78642400 -0.99925900 -1.15055000  H 1.37661700 -1.84307400 -1.55362700  H 0.42992200 -0.43056300 -2.01787400  B 1.80895200 -0.15426100 -0.32014800  H -3.04176600 -1.66785500 -0.96691200  Cl 2.46071300 -0.76496300 1.26035800  Cl 2.55072600 1.35781400 -0.98503600 |
|   B3LYP/LANL2DZ | Total Energy: -360.1020655  Enthalpy: -359.964578  Free energy: -360.021359  ZPE Correction: -359.978339  C -3.56703800 0.29569200 0.77461500  C -2.26066900 0.43671800 0.39031100  C -1.79742000 0.01146500 -0.89811700  C -4.61403300 -0.26045700 -0.02007200  C -1.36324600 -0.34058400 -1.98864700  H -0.99335800 -0.64815300 -2.94078600  C -5.54370000 -0.73016700 -0.66716600  H -6.34418000 -1.14016200 -1.24059600  C -1.23275200 1.05834300 1.32961500  H -0.86557100 2.00228600 0.90399100  H -1.72334500 1.30078400 2.28042500  C -0.01956800 0.11918700 1.61032300  H 0.52304400 0.53493000 2.47990500  H -0.37754400 -0.87135700 1.91688300  B 1.07196400 -0.01076700 0.48855600  H -3.84879700 0.63612400 1.77142900  Br 1.81387100 1.59778800 -0.39858000  Br 1.91928500 -1.74926800 0.09003200 |
|   B3LYP/LANL2DZ  B3LYP/ 6-311++g(d,p)  PBE0/ 6-311++g(d,p)  PBE0/ LANL2DZ | Total Energy: -519.3726737  Enthalpy: -519.218703  Free energy: -519.274655  ZPE Correction: -519.233372  C -2.61926900 -0.66413900 -0.43856500  C -1.27696900 -0.57174600 -0.19873500  C -0.69707500 0.50083600 0.55443900  C -3.61148500 0.24983700 0.02550200  C -0.11253800 1.38609800 1.17413300  H 0.35730900 2.16604300 1.73184400  C -4.49515600 1.01058600 0.40403100  H -5.25715100 1.67805400 0.73892100  C -0.28442300 -1.60960600 -0.71803800  H 0.03165100 -2.25780900 0.11266700  H -0.77386800 -2.25097100 -1.46085400  C 0.96714900 -0.92572100 -1.33455500  H 1.62765900 -1.71791400 -1.73309800  H 0.68239700 -0.29798700 -2.18691900  B 1.85506200 -0.12291600 -0.30671900  H -2.97705100 -1.50670000 -1.03098600  C 2.54622600 1.19851600 -0.69865800  C 2.33109600 -0.79050700 1.00370800  N 3.08353900 2.20072700 -1.02647900  N 2.70908900 -1.32973600 1.98683000  Total Energy: -519.6118778  Enthalpy: -519.458765  Free energy: -519.514675  ZPE Correction: -519.473505  C -2.60797300 -0.64122900 -0.44161400  C -1.27532000 -0.57487000 -0.21966600  C -0.67262200 0.47020400 0.53044400  C -3.56682700 0.27293400 0.05127700  C -0.04269600 1.31870600 1.12069200  H 0.45600700 2.08083400 1.67141600  C -4.41368000 1.03172800 0.45259400  H -5.15219900 1.70667700 0.81162100  C -0.29320400 -1.59657700 -0.76417300  H 0.00237800 -2.27088000 0.04816300  H -0.78536200 -2.20516000 -1.52600600  C 0.95605300 -0.89467600 -1.33323000  H 1.64056300 -1.66025000 -1.73105900  H 0.68282300 -0.25812500 -2.17890500  B 1.80125600 -0.09633500 -0.27369700  H -2.98611300 -1.46362500 -1.04281300  C 2.51295300 1.21557300 -0.65814200  C 2.27784800 -0.79713400 1.01692600  N 3.05275700 2.18471000 -0.98374000  N 2.64559100 -1.36696000 1.95308600  Total Energy: -518.9613463  Enthalpy: -518.807235  Free energy: -518.861831  ZPE Correction: -518.821587  C -2.60737600 -0.63055500 -0.41051400  C -1.26368700 -0.61739000 -0.30634800  C -0.55895200 0.42581300 0.34740900  C -3.46435200 0.35256300 0.12636500  C 0.18831500 1.23810300 0.86495500  H 0.68609700 2.00970500 1.41053600  C -4.21847100 1.17940500 0.57518800  H -4.88061800 1.91215300 0.97387800  C -0.31375000 -1.64744900 -0.86440900  H -0.06715000 -2.36086000 -0.06755100  H -0.79086900 -2.20800500 -1.67245900  C 0.94630400 -0.90580700 -1.30706400  H 1.72817300 -1.61242100 -1.60941200  H 0.72759700 -0.28323100 -2.18071000  B 1.55553800 -0.00220600 -0.13409400  H -3.07240200 -1.45800000 -0.94089900  C 2.42761800 1.20305100 -0.59480200  C 2.08121000 -0.76705900 1.11926800  N 3.06475900 2.09233100 -0.96562100  N 2.44814500 -1.37409700 2.03087800  Total Energy: -518.7366456  Enthalpy: -518.581305  Free energy: -518.636386  ZPE Correction: -518.595742  C -2.62510600 -0.63692700 -0.41289900  C -1.27224000 -0.57853200 -0.28848500  C -0.59759000 0.49376600 0.37096100  C -3.53855200 0.32575500 0.09809100  C 0.11849800 1.34733000 0.89565400  H 0.64140900 2.12669000 1.40729700  C -4.35056800 1.13543100 0.52420200  H -5.05431300 1.84483300 0.89897000  C -0.31561500 -1.62648600 -0.83152900  H -0.05820400 -2.32830500 -0.02515500  H -0.79742800 -2.20619600 -1.62658700  C 0.96089200 -0.92506400 -1.32770200  H 1.69262000 -1.67923200 -1.65723500  H 0.74112100 -0.29649800 -2.19829400  B 1.68400000 -0.07388000 -0.19581000  H -3.05396700 -1.48673200 -0.94249000  C 2.52743500 1.15810300 -0.60834600  C 2.11422600 -0.79145700 1.11104100  N 3.17648600 2.08214200 -0.95148400  N 2.44358200 -1.37023800 2.08528500 |
|  |  |
|   B3LYP/LANL2DZ  H-bond-stabilized TS    B3LYP/LANL2DZ (H-bonded)  B3LYP/ 6-311++g(d,p)  PBE0/ 6-311++g(d,p)  PBE0/ LANL2DZ | Free Energy: -445.526219  Enthalpy: -445.475394  ZPE Corrected Energy: -445.487779  Total Energy: -445.6650982  Imaginary Frequency: -509.8435  C 2.04094700 1.21122200 -0.20154000  C 0.79336900 0.66939600 0.20834000  C 0.77016700 -0.72323600 0.44570600  C 3.15190400 0.36095300 -0.34158800  C 1.62818400 -1.67089700 0.40151100  H 1.83133300 -2.71973900 0.52921000  C 3.35312700 -0.89464400 -0.17345500  H 4.03952700 -1.72307300 -0.21380500  C -0.41578500 1.57491400 0.37837700  H -0.73345200 1.54709400 1.43201400  H -0.11431600 2.61021400 0.16890600  C -1.61639000 1.18055500 -0.53168200  H -2.35504400 1.99832300 -0.47864900  H -1.27136800 1.15234200 -1.57624000  B -2.37754100 -0.17514900 -0.15825200  H 2.08988600 2.28138300 -0.38694600  N -2.69928900 -1.10200200 -1.19572400  H -3.21571900 -1.96065200 -1.04984700  H -2.38008900 -0.96255200 -2.14488800  N -2.78699900 -0.40983800 1.19059700  H -2.56307200 0.23205200 1.93877300  H -3.26909800 -1.24634300 1.49460800  Free Energy: -445.526526  Enthalpy: -445.476460  ZPE Corrected Energy: -445.488708  Total Energy: -445.6662087  Frequency: -504.1133  C -2.00343200 -1.31839200 0.00507000  C -0.85399800 -0.54764500 0.32600900  C -1.01894100 0.85901900 0.28047800  C -3.19700900 -0.66032200 -0.32759000  C -2.00064200 1.64030400 0.01891900  H -2.35540900 2.65187600 -0.07884900  C -3.59415900 0.55122900 -0.42954000  C 0.44447200 -1.24988100 0.69944000  H 0.76985500 -0.89583700 1.68761000  H 0.22819200 -2.32178300 0.79935200  C 1.61425800 -1.05826600 -0.32143200  H 2.24302700 -1.96161200 -0.26379700  H 1.20014200 -1.05584000 -1.34225800  B 2.57382700 0.21044500 -0.13303200  H -1.91462700 -2.40106100 0.03823500  N 2.07848300 1.45909600 0.33801100  H 2.66161100 2.27515600 0.47889600  H 1.08755300 1.61211400 0.49769100  N 3.96053200 0.04709900 -0.45641300  H 4.33700000 -0.84190600 -0.75735100  H 4.63930200 0.79780200 -0.42186900  H -4.38217700 1.24920900 -0.65181400  Free Energy: -445.719129  Enthalpy: -445.666963  ZPE Corrected Energy: -445.679971  Total Energy: -445.8540775  Frequency: -519.9162 cm^-1  C -1.97666300 1.18868600 0.26174700  C -0.79128300 0.64702000 -0.27511900  C -0.82697600 -0.71314900 -0.58482100  C -3.08271100 0.37313100 0.45107500  C -1.70293100 -1.61902200 -0.50607800  H -1.94225400 -2.65196900 -0.66162000  C -3.31158200 -0.85104700 0.23398000  H -4.00703600 -1.66407600 0.29073300  C 0.41706800 1.53296100 -0.47967200  H 0.73377700 1.44965200 -1.52545800  H 0.10981900 2.57203400 -0.33087300  C 1.60259500 1.19715700 0.45845700  H 2.33911700 2.00620400 0.35470700  H 1.25047500 1.25264400 1.49501300  B 2.36674400 -0.17963700 0.19938300  H -1.97315900 2.24664200 0.49723100  N 2.60195700 -1.06221400 1.28650300  H 3.10116400 -1.93433500 1.21152900  H 2.21736700 -0.89887200 2.20149200  N 2.87354200 -0.47828500 -1.09532200  H 2.70906500 0.11161200 -1.89323800  H 3.33435500 -1.34228200 -1.33211100  Free Energy: -445.157967  Enthalpy: -445.105811  ZPE Corrected Energy: -445.118850  Total Energy: -445.2941761  Frequency: -513.6005  C -1.97626300 1.18939700 0.23794100  C -0.77466200 0.66581000 -0.25728300  C -0.77500600 -0.69918900 -0.54372800  C -3.06875000 0.35397500 0.41040800  C -1.61689100 -1.62971700 -0.47968000  H -1.85258100 -2.66661200 -0.62104600  C -3.28701400 -0.86966300 0.21745400  H -3.95560800 -1.70714300 0.25946500  C 0.41919200 1.56106900 -0.44141300  H 0.73407000 1.50350900 -1.49082700  H 0.10489400 2.59542500 -0.26935900  C 1.59450700 1.20061000 0.48102300  H 2.34728200 1.99625300 0.38853500  H 1.24831600 1.24114600 1.52082400  B 2.32600100 -0.18254300 0.18978700  H -1.99823200 2.25107700 0.46143700  N 2.57010900 -1.07878200 1.25896100  H 3.04500800 -1.96029900 1.16167000  H 2.20123200 -0.92010600 2.17963700  N 2.79666700 -0.47018600 -1.11725200  H 2.61349000 0.13083700 -1.90091500  H 3.22400900 -1.34234800 -1.37864400  Free Energy: -444.978289  Enthalpy: -444.927613  ZPE Corrected Energy: -444.939957  Total Energy: -445.1191263  Frequency: -508.9094  C -2.02431500 1.21027600 0.19442500  C -0.77394600 0.68659700 -0.19992600  C -0.73285000 -0.70233100 -0.43643100  C -3.12208300 0.34667800 0.31967300  C -1.56342000 -1.66535900 -0.40705900  H -1.76702200 -2.71363200 -0.53388800  C -3.32246600 -0.90183200 0.15430800  H -3.98796700 -1.74679700 0.18047800  C 0.42377100 1.59533300 -0.35284900  H 0.74194700 1.58410200 -1.40577300  H 0.11891700 2.62570400 -0.12871900  C 1.61145400 1.18386400 0.54774700  H 2.36785500 1.98348200 0.49657700  H 1.27072300 1.15436000 1.59248200  B 2.33431200 -0.18333200 0.16038500  H -2.08825600 2.27855200 0.38217200  N 2.60736200 -1.13998400 1.17964600  H 3.09342800 -2.01104200 1.02171600  H 2.27336200 -1.00993100 2.12245700  N 2.75679900 -0.39903400 -1.18432400  H 2.57079800 0.27036600 -1.91545700  H 3.20866300 -1.24473300 -1.50054200 |
|   B3LYP/LANL2DZ  H-bond-stabilized TS    B3LYP/LANL2DZ (H-bond)  B3LYP/ 6-311++g(d,p) | Total Energy: -485.4183446  Enthalpy: -485.254819  Free energy: -485.304331  ZPE Correction: -485.266633  Frequency: -507.2290  C 2.06766300 1.23193400 -0.07703000  C 0.76988100 0.68969700 0.11348600  C 0.68876600 -0.71868000 0.22388100  C 3.16987300 0.36321000 -0.13084200  C 1.54138800 -1.67405600 0.20660200  H 1.72036600 -2.73241400 0.28574700  C 3.34277100 -0.90374100 -0.05399800  H 4.02122700 -1.73923600 -0.06043500  C -0.44473800 1.59916400 0.20171300  H -0.79230700 1.63034000 1.24383300  H -0.15538300 2.61974600 -0.07988400  C -1.62806800 1.10664000 -0.67715600  H -2.38932800 1.90354000 -0.69282000  H -1.29492800 0.96725000 -1.71575200  B -2.33278900 -0.18427100 -0.09964900  H 2.15912800 2.31064800 -0.17484300  O -2.69731900 -1.29462000 -0.85417200  H -2.40572400 -1.35698100 -1.77986500  O -2.69190700 -0.18811700 1.23486700  H -3.13051300 -0.98465500 1.58676600  H-Bonded TS  Total Energy: -485.4242645  Enthalpy: -485.260773  Free energy: -485.308913  ZPE Correction: -485.272267  Frequency: -489.9255  C 2.01220500 1.30822100 -0.02271900  C 0.80097300 0.62829900 0.25482800  C 0.85853300 -0.79356400 0.25554600  C 3.16364800 0.54777100 -0.26768100  C 1.81301500 -1.63162600 0.06593200  H 2.11780100 -2.66324400 0.01219600  C 3.51972000 -0.67410700 -0.32557000  C -0.46539400 1.41720500 0.55704600  H -0.76317800 1.21832100 1.59758700  H -0.22716200 2.48675300 0.49636700  C -1.67082900 1.11195100 -0.38348100  H -2.39274100 1.93605200 -0.30034800  H -1.32416900 1.12440500 -1.42922900  B -2.47579600 -0.22673900 -0.12123500  H 2.00766000 2.39455600 -0.02686800  O -1.92965700 -1.42306200 0.29641400  H -0.94541400 -1.48590400 0.39671200  O -3.84798100 -0.19086000 -0.32932400  H -4.33553800 -1.02567400 -0.19183900  H 4.27160800 -1.42509600 -0.48853300  Total Energy: -485.6277336  Enthalpy: -485.465176  Free energy: -485.515475  ZPE Correction: -485.477225  Frequency: -518.9510  C 2.03630800 1.20690900 -0.13832800  C 0.78437800 0.65604000 0.20018800  C 0.74829800 -0.72945900 0.37565100  C 3.13646500 0.37326200 -0.26521500  C 1.61414000 -1.64715200 0.30449500  H 1.81984100 -2.69435300 0.40556600  C 3.31880400 -0.87155400 -0.15339200  H 4.00428800 -1.69391800 -0.19105500  C -0.42660000 1.54922000 0.34170100  H -0.76755600 1.51623800 1.38171200  H -0.12725700 2.57973300 0.13371900  C -1.59828300 1.13396600 -0.57880600  H -2.34054100 1.94399100 -0.56099800  H -1.24543200 1.07338800 -1.61477800  B -2.35710100 -0.18209700 -0.13489700  H 2.08287700 2.27885200 -0.28930100  O -2.73065500 -1.18689800 -0.99432200  H -2.40127100 -1.07628600 -1.88966600  O -2.73565300 -0.30204300 1.17323000  H -3.19003700 -1.13302800 1.35026100 |
|   B3LYP/LANL2DZ  B3LYP/ 6-311++g(d,p)  PBE0/ 6-311++g(d,p)  PBE0/ LANL2DZ | Total Energy: -334.853163  Enthalpy: -334.705391  Free energy: -334.746549  ZPE Correction: -334.714527  Frequency: -343.7623  C -0.97173100 -1.44769100 0.01776300  C 0.19237700 -0.69410700 0.10469200  C 0.07918900 0.76087900 0.09651800  C -2.17019500 -0.71311200 -0.05270600  C -1.00728100 1.46496600 0.00465800  H -1.50310600 2.41970200 -0.00269000  C -2.83932000 0.34401300 -0.10583300  H -3.67283000 1.01408100 -0.14952400  C 1.62936300 -1.15901500 0.16781500  H 1.89945000 -1.31193400 1.22539700  H 1.78144100 -2.11587900 -0.35053600  C 2.45009300 0.03865300 -0.39731500  H 3.50108800 -0.00558500 -0.07755500  H 2.43830500 0.00941800 -1.49699000  B 1.72345700 1.39552400 0.18272100  H 1.85289900 2.42024400 -0.44055100  H 1.85975300 1.55716900 1.38090600  H -0.94925400 -2.53235100 -0.01561300  Total Energy: -334.9995423  Enthalpy: -334.853172  Free energy: -334.894618  ZPE Correction: -334.862486  Frequency: -350.8169  C -0.95973100 -1.42966300 0.02330700  C 0.19376500 -0.68883700 0.11608500  C 0.07912900 0.75430800 0.10992200  C -2.13337800 -0.68714900 -0.04858300  C -1.00726000 1.42609600 -0.00059600  H -1.51650400 2.36858100 0.00033500  C -2.82332400 0.32904100 -0.11622200  H -3.65029800 0.99918700 -0.16133300  C 1.62274500 -1.14622000 0.16127800  H 1.89325600 -1.30218900 1.21483500  H 1.76397700 -2.10294800 -0.35186500  C 2.42862300 0.04700900 -0.40479400  H 3.48320500 -0.01184800 -0.11591400  H 2.39542300 0.01393100 -1.50059200  B 1.70178700 1.39376800 0.18780200  H 1.82396500 2.42164700 -0.42672100  H 1.85061400 1.52797200 1.38672800  H -0.95598500 -2.51068000 -0.02686500  Total Energy: -334.5686666  Enthalpy: -334.421385  Free energy: -334.462883  ZPE Correction: -334.430712  Frequency: -302.6393  C -0.95172400 -1.41188100 0.02573100  C 0.20493800 -0.69320100 0.12014500  C 0.09602800 0.75004100 0.11548200  C -2.11971700 -0.65577700 -0.04797700  C -0.96882000 1.43921900 0.00011400  H -1.52174700 2.35706600 -0.02017900  C -2.88694400 0.29481800 -0.11655800  H -3.71258600 0.96752200 -0.17069500  C 1.62781600 -1.14190300 0.16197700  H 1.90496700 -1.27638200 1.21753100  H 1.77035200 -2.10662700 -0.33648500  C 2.41375600 0.04242900 -0.42100300  H 3.47578000 -0.00958000 -0.15710900  H 2.35251900 0.01359000 -1.51612200  B 1.70411800 1.38001500 0.19418200  H 1.83578600 2.42572000 -0.39530500  H 1.85155500 1.48028600 1.40127800  H -0.96921500 -2.49413400 -0.02128300  Total Energy: -334.4336485  Enthalpy: -334.284598  Free energy: -334.325734  ZPE Correction: -334.293710  Frequency: -307.0145  C -0.96252200 -1.43339600 0.02155200  C 0.20152900 -0.69815800 0.10906000  C 0.09211800 0.75473700 0.10100200  C -2.15328700 -0.68859300 -0.05021800  C -0.97699500 1.46981500 0.00253900  H -1.50574300 2.40568000 -0.01540900  C -2.88272600 0.31709800 -0.10826200  H -3.71223400 0.99042300 -0.15721200  C 1.63267900 -1.15337100 0.16521000  H 1.91055200 -1.29654600 1.22117700  H 1.78650700 -2.11136400 -0.34778800  C 2.43467800 0.03931200 -0.40862300  H 3.49012600 0.00520800 -0.10719400  H 2.40368100 0.01633000 -1.50704100  B 1.71320500 1.38389600 0.18953900  H 1.85263900 2.42445900 -0.41001000  H 1.85217400 1.51919500 1.39466200  H -0.95656800 -2.51752300 -0.01244500 |
|   B3LYP/LANL2DZ | Total Energy: -533.4937377  Enthalpy: -533.355964  Free energy: -533.401744  ZPE Correction: -533.366803  Frequency: -341.7106  C 1.96840300 1.24646800 0.01127800  C 0.60821500 0.96326600 0.02380200  C 0.19826400 -0.43047700 -0.00095700  C 2.82859000 0.13282700 -0.00504900  C 0.94003500 -1.48706400 -0.02359900  H 1.04495300 -2.55773700 -0.03130000  C 3.06725800 -1.09638200 -0.02460500  H 3.60158000 -2.02431000 -0.02885300  C -0.55841000 1.92782500 0.04309500  H -0.78089600 2.18611100 1.08967900  H -0.33069700 2.86333200 -0.48304600  C -1.75583700 1.13840700 -0.55503900  H -2.71830900 1.56846900 -0.24857900  H -1.71282800 1.14342700 -1.65280600  B -1.62324800 -0.38209800 -0.01992400  H 2.33516100 2.26820600 0.00155900  F -2.13563100 -1.42132500 -0.83840100  F -1.98679500 -0.56825600 1.35389100 |
|   B3LYP/LANL2DZ | Total Energy: -363.6470742  Enthalpy: -363.511098  Free energy: -363.559243  ZPE Correction: -363.522697  Frequency: -221.2547  C 2.59571200 0.94851700 -0.21601600  C 1.21654200 0.95701100 -0.24659200  C 0.52183800 -0.31377000 -0.00701800  C 3.21272600 -0.29180500 0.06291900  C 1.03836400 -1.47184700 0.23214000  H 1.00949800 -2.52180200 0.45818200  C 3.37378400 -1.49771400 0.31620700  H 3.73253700 -2.48074600 0.53592600  C 0.25724500 2.09480500 -0.51624700  H 0.05769500 2.61958600 0.42991300  H 0.67072400 2.82740300 -1.22033600  C -1.04748700 1.40628300 -1.00806700  H -1.92980500 2.04321700 -0.87819000  H -0.96506200 1.15742300 -2.07498000  B -1.18429600 0.04245900 -0.14671300  H 3.17833100 1.84474000 -0.40688100  Cl -1.75018100 0.37209300 1.66701600  Cl -2.18186500 -1.35391600 -0.95019400 |
|   B3LYP/LANL2DZ | Total Energy: -360.0706795  Enthalpy: -359.935271  Free energy: -359.986104  ZPE Correction: -359.947402  Frequency: -199.3970  C -3.29435800 0.31115100 0.67328000  C -1.93066900 0.43058600 0.82981000  C -1.06923600 -0.31083500 -0.10384600  C -3.74363700 -0.50691400 -0.38926200  C -1.42774000 -1.08917100 -1.06884700  H -1.27638500 -1.73551400 -1.91333700  C -3.77774900 -1.27215200 -1.36593200  H -4.01007600 -1.90943600 -2.19224400  C -1.13313300 1.20598200 1.85442400  H -0.99826300 2.23603300 1.49296100  H -1.64549200 1.25889500 2.82292700  C 0.24678600 0.49141200 1.90610700  H 1.03914200 1.13713500 2.30046800  H 0.18565800 -0.39688200 2.55012100  B 0.56764100 0.02982900 0.38499900  H -3.99264500 0.81025800 1.33858500  Br 1.13367200 1.65443200 -0.82789400  Br 1.85599200 -1.57186000 0.18964000 |
|   B3LYP/LANL2DZ  B3LYP/ 6-311++g(d,p)  PBE0/ 6-311++g(d,p)  PBE0/ LANL2DZ | Total Energy: -519.3494371  Enthalpy: -519.197279  Free energy: -519.248010  ZPE Correction: -519.210201  Frequency: -214.2187  C -2.45031700 -0.91590000 -0.25696500  C -1.07220100 -0.93324300 -0.28545600  C -0.37492900 0.31741100 0.02920200  C -3.05869500 0.31236400 0.09072300  C -0.87559100 1.46364300 0.34132600  H -0.85033100 2.50126400 0.62003000  C -3.22949400 1.49946600 0.41330300  H -3.58823700 2.46870800 0.68817800  C -0.11194500 -2.05214900 -0.61782800  H 0.09534600 -2.62462700 0.29954700  H -0.52697600 -2.75251400 -1.35297700  C 1.18436700 -1.33406000 -1.08694600  H 2.06197500 -1.98778200 -1.01901900  H 1.08200900 -1.01798000 -2.13330500  B 1.35365500 -0.01586700 -0.12060200  H -3.04119800 -1.79406100 -0.49973400  C 2.13365700 1.20153700 -0.73378800  C 1.82551900 -0.36643200 1.34549500  N 2.71728900 2.11059800 -1.21640200  N 2.16512700 -0.66338300 2.44010100  Total Energy: -519.5861891  Enthalpy: -519.435034  Free energy: -519.486094  ZPE Correction: -519.448130  Frequency: -226.3278  C -2.42175700 -0.90716400 -0.26652200  C -1.05861000 -0.93247600 -0.28359600  C -0.37232500 0.30529000 0.05160400  C -3.01070500 0.30906200 0.09570600  C -0.87654700 1.42970200 0.36261200  H -0.86744100 2.46038600 0.64986800  C -3.20996700 1.46826200 0.42314700  H -3.56316000 2.43216300 0.70719600  C -0.10021000 -2.03098600 -0.64452300  H 0.10232400 -2.62283100 0.25737000  H -0.51996300 -2.71275700 -1.38872900  C 1.18510800 -1.30439000 -1.09722200  H 2.06059400 -1.95691600 -1.05305700  H 1.07670100 -0.97222400 -2.13414000  B 1.35020900 -0.00269600 -0.11308700  H -3.02551700 -1.76602800 -0.53208600  C 2.10989000 1.23039300 -0.71840400  C 1.82912800 -0.37954200 1.34068900  N 2.64724100 2.13677100 -1.19363600  N 2.14438600 -0.70494500 2.40478800  Total Energy: -518.7203045  Enthalpy: -518.566675  Free energy: -518.617512  ZPE Correction: -518.579616  Frequency: -169.4074  C -2.42187900 -0.91355600 -0.26938100  C -1.05287900 -0.93056600 -0.29649700  C -0.36696600 0.31874400 0.02805100  C -3.04875800 0.30272600 0.08586800  C -0.82723700 1.46724500 0.34591800  H -0.85673800 2.50288600 0.62918000  C -3.34005500 1.45726000 0.41226000  H -3.73368500 2.41143900 0.69017400  C -0.08445000 -2.02831000 -0.63923500  H 0.13909400 -2.60214700 0.27246000  H -0.49424200 -2.72947700 -1.37481000  C 1.18654600 -1.29191200 -1.11160800  H 2.07536500 -1.93154800 -1.07084000  H 1.06599400 -0.95005100 -2.14685700  B 1.36088500 -0.00459000 -0.11947600  H -3.00840200 -1.79152100 -0.52172000  C 2.14683300 1.21920400 -0.69459700  C 1.80501100 -0.39382900 1.33795200  N 2.73739300 2.13350100 -1.15091300  N 2.12420700 -0.72331000 2.42625700  Total Energy: -518.7203045  Enthalpy: -518.566675  Free energy: -518.617512  ZPE Correction: -518.579616  Frequency: -169.4074  C -2.42187900 -0.91355600 -0.26938100  C -1.05287900 -0.93056600 -0.29649700  C -0.36696600 0.31874400 0.02805100  C -3.04875800 0.30272600 0.08586800  C -0.82723700 1.46724500 0.34591800  H -0.85673800 2.50288600 0.62918000  C -3.34005500 1.45726000 0.41226000  H -3.73368500 2.41143900 0.69017400  C -0.08445000 -2.02831000 -0.63923500  H 0.13909400 -2.60214700 0.27246000  H -0.49424200 -2.72947700 -1.37481000  C 1.18654600 -1.29191200 -1.11160800  H 2.07536500 -1.93154800 -1.07084000  H 1.06599400 -0.95005100 -2.14685700  B 1.36088500 -0.00459000 -0.11947600  H -3.00840200 -1.79152100 -0.52172000  C 2.14683300 1.21920400 -0.69459700  C 1.80501100 -0.39382900 1.33795200  N 2.73739300 2.13350100 -1.15091300  N 2.12420700 -0.72331000 2.42625700 |
|  |  |
|   B3LYP/LANL2DZ | Total Energy: -680.5677011  Enthalpy: -680.381038  Free energy: -680.444309  ZPE Correction: -680.397634  C 1.91749500 -0.22771100 -0.62350100  C 2.78460400 -1.24020900 -0.27390300  C 2.31735400 -2.44658800 0.34407700  C 0.51404500 -0.18056600 -0.43023900  C 1.98032300 -3.50141500 0.87631300  H 1.62937800 -4.39626900 1.33529200  C -0.71622400 -0.04214300 -0.28269900  C 4.29572900 -1.20314600 -0.55036800  H 4.81332600 -1.58960800 0.34095800  H 4.50316000 -1.93493100 -1.35037800  C 4.91851500 0.16367500 -0.93844400  H 5.92367100 -0.03244900 -1.34992600  H 4.35049700 0.60565800 -1.77370900  B 5.06817300 1.25023000 0.22649500  H 2.35400900 0.65490400 -1.09507200  Au -2.63478500 0.20261700 -0.02624200  F -4.63546600 0.48313400 0.25508200  N 4.14612800 1.31079700 1.30787000  H 4.19546300 1.98843300 2.05847700  H 3.33051500 0.71030500 1.33380300  N 6.16773500 2.17427300 0.15073000  H 6.84304800 2.13110900 -0.60053400  H 6.31521900 2.92989400 0.80842000 |
|   B3LYP/LANL2DZ | Total Energy: -595.696135  Enthalpy: -595.509772  Free energy: -595.575059  ZPE Correction: -595.526835  C -2.18911800 0.22145100 -0.60925500  C -3.05849600 1.22826700 -0.25371600  C -2.59201700 2.43883900 0.35761500  C -0.78198500 0.18914900 -0.42936600  C -2.25388700 3.49559600 0.88444100  H -1.90307800 4.39310300 1.33850800  C 0.45060400 0.06848500 -0.29475400  C -4.57172200 1.18135500 -0.51430400  H -5.08294000 1.55070700 0.38784000  H -4.79226800 1.92347800 -1.30093400  C -5.18631400 -0.18465600 -0.91650600  H -6.19528000 0.00948200 -1.31914000  H -4.62049500 -0.61126200 -1.76119600  B -5.32267000 -1.28784500 0.23445000  H -2.62147600 -0.66613200 -1.07435400  N -4.40015000 -1.35165100 1.31541600  H -4.44344600 -2.03880600 2.05772100  H -3.59077400 -0.74348700 1.35035100  N -6.41118300 -2.22326400 0.14663200  H -7.08745100 -2.17810700 -0.60369000  H -6.54963300 -2.98932200 0.79414400  Au 2.38996400 -0.14578200 -0.06008200  Cl 4.79235100 -0.44118700 0.24269000 |
|   B3LYP/LANL2DZ | Total Energy: -593.9183624  Enthalpy: -593.732109  Free energy: -593.799047  ZPE Correction: -593.749432  C -2.68005600 0.20873100 -0.59295100  C -3.55526500 1.20984300 -0.23668300  C -3.09474400 2.42553400 0.36927300  C -1.27114700 0.19144200 -0.42176500  C -2.76133700 3.48583900 0.89171900  H -2.41456300 4.38717800 1.34133900  C -0.03563700 0.09035300 -0.29927800  C -5.06887700 1.15240900 -0.49194000  H -5.57995700 1.51242200 0.41405700  H -5.29708400 1.89790800 -1.27308100  C -5.67407900 -0.21558200 -0.90116900  H -6.68412600 -0.02629000 -1.30327100  H -5.10507100 -0.63416400 -1.74770300  B -5.80410000 -1.32495100 0.24447100  H -3.10670100 -0.68408400 -1.05296600  N -4.88296800 -1.38638800 1.32687300  H -4.92293200 -2.07659200 2.06653300  H -4.07850100 -0.77208400 1.36637900  N -6.88555900 -2.26772900 0.15077600  H -7.56086500 -2.22411000 -0.60051800  H -7.01983900 -3.03750500 0.79474000  Au 1.91739400 -0.07926600 -0.09472600  Br 4.47264400 -0.31909900 0.17167700 |
|   B3LYP/LANL2DZ | Total Energy: -657.0361407  Enthalpy: -656.824405  Free energy: -656.891632  ZPE Correction: -656.842743  C -1.97488900 0.15973000 -0.51646200  C -2.77345500 1.23604800 -0.22629000  C -2.22274500 2.46222600 0.27968600  C -0.55959700 0.09643600 -0.36369800  C -1.81382000 3.53414500 0.71315500  H -1.43297400 4.45660200 1.08745800  C 0.66677500 -0.02182100 -0.24625700  C -4.29039000 1.25301400 -0.44435100  H -4.76268800 1.60701800 0.48466700  H -4.49253100 2.03903400 -1.19034300  C -4.96212900 -0.06750700 -0.89172100  H -5.96095500 0.18811300 -1.28178600  H -4.42118500 -0.48573400 -1.75671500  B -5.16570800 -1.21076400 0.21034100  H -2.44934700 -0.74395600 -0.89493000  N -4.36132400 -1.26642200 1.38531500  H -4.47587000 -1.96309700 2.11112500  H -3.58923200 -0.63012600 1.53534300  N -6.19154900 -2.18554600 -0.01686500  H -6.79335100 -2.14442800 -0.82859300  H -6.37264400 -2.96888400 0.59868400  Au 2.57960000 -0.20926600 -0.03341600  O 4.71926800 -0.44244500 0.22397700  H 5.20406700 -1.22824200 -0.07974500  H 5.28430100 0.24922200 0.60769400 |
|   B3LYP/LANL2DZ | Total Energy: -591.9017147  Enthalpy: -591.696845  Free energy: -591.765617  ZPE Correction: -591.715315  C -2.24397700 0.13679500 -0.49091300  C -3.03347900 1.22316600 -0.21465300  C -2.47196100 2.45139900 0.27452300  C -0.82798600 0.07189400 -0.34163300  C -2.05372000 3.52532400 0.69349000  H -1.66645600 4.44978700 1.05626400  C 0.39994800 -0.04020300 -0.23053900  C -4.55003400 1.24995000 -0.43099800  H -5.01946400 1.60298600 0.49990500  H -4.74681300 2.04114100 -1.17280800  C -5.22958700 -0.06385400 -0.88502800  H -6.22523600 0.20047300 -1.27719900  H -4.68883700 -0.48255200 -1.74991800  B -5.44643500 -1.21047200 0.21111200  H -2.72413900 -0.76941600 -0.85528500  N -4.65837200 -1.26787900 1.39710100  H -4.78231500 -1.96677300 2.11924300  H -3.88971800 -0.63096400 1.56094600  N -6.46724800 -2.18576400 -0.03354200  H -7.05708900 -2.14344700 -0.85396600  H -6.65634300 -2.97102400 0.57710500  Au 2.33495100 -0.18825300 -0.04668200  S 4.85880200 -0.39495700 0.20422700  H 5.16111500 0.83696300 0.74083800  H 5.26962500 -0.07486200 -1.07085700 |
|   B3LYP/LANL2DZ | Total Energy: -706.8634287  Enthalpy: -706.554842  Free energy: -706.634840  ZPE Correction: -706.578327  C 2.94280500 -0.12458800 -0.49010900  C 3.74958300 -1.19608700 -0.20169600  C 3.20704200 -2.42712800 0.30117500  C 1.52623100 -0.07738700 -0.34648700  C 2.80634100 -3.50321100 0.73231000  H 2.43107500 -4.42863300 1.10477000  C 0.29270000 0.01324900 -0.24586100  C 5.26623000 -1.20571300 -0.42292300  H 5.74327800 -1.55446000 0.50568700  H 5.47007000 -1.99295200 -1.16723600  C 5.93030300 0.11661300 -0.87691600  H 6.92574800 -0.13602200 -1.27745900  H 5.37894300 0.53388000 -1.73571700  B 6.14106800 1.25850400 0.22473800  H 3.41220300 0.78327400 -0.86548900  N 5.33263100 1.32211800 1.39650000  H 5.44974700 2.01851800 2.12213400  H 4.55516800 0.69162000 1.54383900  N 7.17726300 2.22330000 0.00093000  H 7.78124500 2.17575100 -0.80881700  H 7.36345500 3.00550200 0.61638100  Au -1.67530500 0.10995900 -0.08975500  P -4.09769900 0.20839200 0.09109500  C -4.80080500 1.88903200 0.56234100  H -5.89455900 1.84956700 0.62319400  H -4.39226200 2.19427800 1.53047400  H -4.50367100 2.62958900 -0.18643800  C -4.84135400 -0.95456300 1.37022100  H -5.93454000 -0.87424200 1.38068200  H -4.55328000 -1.98358400 1.13477400  H -4.44757800 -0.70443500 2.36009200  C -5.02219200 -0.22764700 -1.48940400  H -6.10584400 -0.16068700 -1.33781400  H -4.72194000 0.45952300 -2.28629800  H -4.75859800 -1.24499600 -1.79415000 |
|   B3LYP/LANL2DZ | Total Energy: -814.379023  Enthalpy: -814.356493  Free energy: -814.436179  ZPE Correction: -814.5909679  C -2.92959900 0.12137900 -0.47830100  C -3.71739000 1.20665700 -0.19279800  C -3.15244500 2.43049100 0.30321800  C -1.51335200 0.06126400 -0.33394100  C -2.72926100 3.49975400 0.72859900  H -2.34007500 4.42044300 1.09912800  C -0.28321100 -0.03774700 -0.22606600  C -5.23396600 1.23541000 -0.40379900  H -5.70079300 1.57001900 0.53519400  H -5.43325900 2.04111200 -1.12902100  C -5.91242400 -0.07003100 -0.88173000  H -6.90896900 0.20078500 -1.26690200  H -5.37267300 -0.47105100 -1.75558600  B -6.12761400 -1.23857700 0.19172600  H -3.40908700 -0.78343600 -0.84629100  N -5.35108400 -1.30870200 1.38465800  H -5.47592800 -2.02117700 2.09327300  H -4.59171400 -0.66662900 1.56972500  N -7.13502900 -2.21963300 -0.08141600  H -7.71670500 -2.16869100 -0.90718200  H -7.32296900 -3.01719700 0.51341500  Au 1.66408600 -0.14928300 -0.05149000  P 4.08234600 -0.19115100 0.07867500  O 5.02931700 -1.36654900 -0.71858400  H 5.25206800 -1.22088200 -1.66130600  O 4.87636200 1.17183000 -0.61753400  H 4.42356700 2.03981100 -0.57924900  O 4.72994800 -0.36408500 1.62862200  H 5.68428400 -0.58385300 1.70990900 |
|  |  |
|   B3LYP/LANL2DZ | Total Energy: -720.3128121  Enthalpy: -720.153042  Free energy: -720.216891  ZPE Correction: -720.169452  C 1.53147300 1.49742700 -0.12080400  C 2.70255000 0.83091200 -0.41491600  C 2.70225700 -0.55974600 -0.75962300  C 0.21311900 0.98012100 -0.09839200  C 2.72174100 -1.74905200 -1.06902200  H 2.66406500 -2.77862200 -1.33579800  C -0.96939700 0.58692200 -0.05291400  C 4.04689300 1.55527800 -0.38244700  H 4.55522600 1.45368000 -1.35237000  H 3.85668100 2.62583200 -0.22203000  C 5.01951900 1.04400800 0.72697200  H 5.87672000 1.73758800 0.77050400  H 4.52205900 1.07229000 1.70431600  B 5.58047300 -0.39809000 0.45751000  H 1.63424500 2.55963800 0.12603100  O 5.52347200 -1.34924900 1.47296800  H 5.77455300 -2.27827500 1.32543600  O 6.19056900 -0.64573300 -0.77231800  H 6.44567000 -1.54161800 -1.05264300  Au -2.81146700 -0.04525200 0.04588000  F -4.73854600 -0.71561700 0.17179300 |
|   B3LYP/LANL2DZ | Total Energy: -635.298648  Enthalpy: -635.281903  Free energy: -635.346652  ZPE Correction: -635.4414179  C 1.81983700 1.49785500 -0.09873800  C 2.98007200 0.81209600 -0.38539300  C 2.95965400 -0.58413400 -0.70821600  C 0.49355400 0.99682100 -0.07160300  C 2.95863200 -1.77830500 -0.99711200  H 2.88578500 -2.81177500 -1.24468200  C -0.69534400 0.62594800 -0.03283800  C 4.33288700 1.52084900 -0.37306100  H 4.82348700 1.41390000 -1.35146100  H 4.15696100 2.59328400 -0.20976400  C 5.31696500 0.99718800 0.71910500  H 6.17659600 1.68811400 0.75740700  H 4.83393200 1.01945400 1.70379800  B 5.87481000 -0.44283100 0.43180000  H 1.93441900 2.56173000 0.13346800  O 5.85561900 -1.39308600 1.44932000  H 6.11599700 -2.31882900 1.29729500  O 6.44619900 -0.68730300 -0.81662800  H 6.70496300 -1.57988400 -1.10385200  Au -2.56948700 0.04132200 0.03194100  Cl -4.90757800 -0.67665400 0.11535500 |
|   B3LYP/LANL2DZ | Total Energy: -633.6636783  Enthalpy: -633.504259  Free energy: -633.570466  ZPE Correction: -633.521240  C -2.34630300 -1.51431000 -0.08075900  C -3.49005400 -0.80372700 -0.37171400  C -3.43699500 0.58862200 -0.70769200  C -1.00808900 -1.04443900 -0.06178000  C -3.40796700 1.77958800 -1.00771600  H -3.31133500 2.80874200 -1.26501900  C 0.18952300 -0.70212000 -0.03127800  C -4.85908600 -1.48031900 -0.35150700  H -5.34745300 -1.37127500 -1.33076500  H -4.70820300 -2.55491300 -0.17788900  C -5.83009600 -0.92302500 0.73567000  H -6.70481400 -1.59419800 0.78162400  H -5.34707500 -0.94569000 1.72034500  B -6.35613900 0.52578500 0.43294500  H -2.48534200 -2.57281200 0.16190800  O -6.31433100 1.48678300 1.43949600  H -6.55560000 2.41606500 1.27785100  O -6.92314900 0.76859000 -0.81775200  H -7.16387900 1.66306400 -1.11461400  Au 2.08362300 -0.16106700 0.01670500  Br 4.56664100 0.53719300 0.07853800 |
|   B3LYP/LANL2DZ | Total Energy: -696.7813784  Enthalpy: -696.596470  Free energy: -696.664105  ZPE Correction: -696.614571  C 1.55157100 1.55705600 -0.09457200  C 2.69541500 0.84878500 -0.36538800  C 2.64768200 -0.55159600 -0.67559800  C 0.22131400 1.04707800 -0.07401300  C 2.62210400 -1.74610100 -0.95221700  H 2.56959300 -2.78335800 -1.19100100  C -0.94956000 0.64724900 -0.03813200  C 4.06103000 1.52859400 -0.34057200  H 4.54721600 1.42150900 -1.32000000  H 3.90711000 2.60151700 -0.16425100  C 5.02188800 0.95976300 0.74515300  H 5.85397700 1.67481100 0.86038900  H 4.51578300 0.90423500 1.71677000  B 5.66771400 -0.43019600 0.39324500  H 1.66146100 2.61974000 0.13073800  O 5.77965000 -1.38901400 1.39327600  H 6.17387200 -2.26576200 1.23923600  O 6.16946900 -0.60607700 -0.89194600  H 6.55796000 -1.44179400 -1.20236300  Au -2.76042300 -0.02477100 0.04029700  O -4.78893900 -0.80268700 0.14910000  H -4.99053300 -1.75289600 0.11434900  H -5.57169600 -0.23287900 0.23505900 |
|   B3LYP/LANL2DZ | Total Energy: -631.6470033  Enthalpy: -631.468966  Free energy: -631.538057  ZPE Correction: -631.487158  C -1.84329100 -1.54529400 -0.08317900  C -2.97773700 -0.82139600 -0.35053200  C -2.91367700 0.58090500 -0.64895000  C -0.50597900 -1.05301600 -0.06074800  C -2.87462600 1.77748400 -0.91409700  H -2.81285700 2.81663400 -1.14249300  C 0.67444800 -0.68059100 -0.02738200  C -4.35017900 -1.48725400 -0.33648800  H -4.82705300 -1.37365100 -1.31972400  H -4.20800900 -2.56183000 -0.16105500  C -5.31372000 -0.91008800 0.74190600  H -6.14776500 -1.62278600 0.85753200  H -4.81286300 -0.85095600 1.71600900  B -5.95753500 0.47831200 0.38011800  H -1.96558800 -2.60808200 0.13451200  O -6.09018300 1.43489300 1.37941400  H -6.48995400 2.30855100 1.22227300  O -6.43632100 0.65391800 -0.91362200  H -6.82759300 1.48634800 -1.22928200  Au 2.52679300 -0.07744400 0.03319400  S 4.95128400 0.69201600 0.12318800  H 4.82440700 1.84477300 0.86613500  H 5.05838400 1.34026200 -1.08736100 |
|   B3LYP/LANL2DZ | Total Energy: -746.608827  Enthalpy: -746.327059  Free energy: -746.406560  ZPE Correction: -746.350199  C -2.59993400 -1.56627900 -0.05956500  C -3.71432200 -0.81302600 -0.33386000  C -3.61107600 0.58202900 -0.65336300  C -1.24740800 -1.11972100 -0.04941400  C -3.53976500 1.77330000 -0.93646500  H -3.44559400 2.80641400 -1.18019000  C -0.05227600 -0.78548200 -0.02808200  C -5.10565900 -1.43903000 -0.30742100  H -5.58195700 -1.32676700 -1.29124400  H -4.99346400 -2.51465000 -0.11653800  C -6.05159900 -0.82063300 0.76409100  H -6.91035100 -1.50286900 0.88303800  H -5.55008800 -0.77048000 1.73837900  B -6.64305500 0.58697700 0.38859200  H -2.75736800 -2.62153100 0.17416000  O -6.72990100 1.56254200 1.37511400  H -7.08946800 2.45105800 1.20453700  O -7.12755600 0.76419800 -0.90323800  H -7.48002100 1.61036300 -1.22802100  Au 1.84624000 -0.23773300 0.00882800  P 4.18344200 0.42990900 0.05939000  C 4.63215000 1.63787100 1.43075500  H 5.69732000 1.89369300 1.39177000  H 4.03398200 2.54776400 1.32255900  H 4.40061300 1.18677500 2.40044800  C 5.40227900 -0.98358400 0.30263600  H 5.28713900 -1.70557800 -0.51152800  H 6.43305300 -0.61087100 0.31762900  H 5.18410500 -1.49113900 1.24720100  C 4.80329400 1.28188600 -1.49984800  H 5.86224900 1.54803100 -1.40308900  H 4.67187100 0.61238300 -2.35535000  H 4.21424700 2.18691400 -1.67680600 |
|   B3LYP/LANL2DZ | Total Energy: -854.3362699  Enthalpy: -854.128606  Free energy: -854.207823  ZPE Correction: -854.150742  C -2.58569400 -1.58643700 -0.10844900  C -3.68450800 -0.80415900 -0.36004400  C -3.55357700 0.60003500 -0.62545400  C -1.22868900 -1.15362500 -0.07560000  C -3.45620500 1.79916800 -0.86170600  H -3.34753700 2.83963100 -1.06575200  C -0.03348100 -0.82916000 -0.03757300  C -5.08608500 -1.40549700 -0.36369800  H -5.55230700 -1.24919100 -1.34613000  H -4.99474200 -2.48878400 -0.21019300  C -6.02594100 -0.80513500 0.72266600  H -6.88973600 -1.48350500 0.82501200  H -5.52614700 -0.78423900 1.69886300  B -6.61100000 0.61482900 0.38351700  H -2.75510900 -2.64709200 0.08517300  O -6.71521700 1.55483800 1.40122200  H -7.08345800 2.44512900 1.26151000  O -7.06892500 0.83456100 -0.91072600  H -7.42965600 1.68561000 -1.21281000  Au 1.84946100 -0.29603500 0.02586000  P 4.14923700 0.46414000 0.06589900  O 5.11761300 -0.16694700 1.29559400  H 6.09080300 -0.05295000 1.22009400  O 5.22601800 0.19841700 -1.23153200  H 5.12785000 0.73669500 -2.04343000  O 4.35437700 2.17408000 0.13076000  H 3.68325100 2.69844800 0.61528000 |
|   B3LYP/LANL2DZ | Total Energy: -794.7358261  Enthalpy: -794.487688  Free energy: -794.565407  ZPE Correction: -794.510030  C -2.58652600 -1.57747900 -0.01909100  C -3.69834200 -0.82760000 -0.31129900  C -3.58997400 0.56000300 -0.66053200  C -1.23371400 -1.13096100 -0.01773900  C -3.51290400 1.74457600 -0.96859700  H -3.41538800 2.77205500 -1.23408300  C -0.03965200 -0.79502100 -0.00395500  C -5.09134200 -1.44883400 -0.27356600  H -5.56473800 -1.35740600 -1.26088800  H -4.98289700 -2.52009500 -0.05790300  C -6.03748500 -0.80244000 0.78091100  H -6.89696300 -1.48066000 0.91619000  H -5.53746500 -0.72829200 1.75442800  B -6.62826300 0.59550100 0.36957400  H -2.74488700 -2.62702200 0.23756300  O -6.72250500 1.59261600 1.33339300  H -7.08486700 2.47576200 1.14213400  O -7.10384700 0.74264500 -0.92914000  H -7.45789300 1.57970800 -1.27508100  Au 1.85157600 -0.23698800 0.01674000  P 4.18181000 0.44871200 0.04541200  N 4.48489200 1.59582800 1.30097200  H 3.70923400 2.08258700 1.73184800  H 5.41899800 1.92197300 1.52214400  N 5.51489400 -0.63084300 0.38162200  H 5.78674500 -1.29479400 -0.33478300  H 5.65635800 -0.93479200 1.33846800  N 4.70288000 0.98624900 -1.51150800  H 5.66977400 1.22021200 -1.70697000  H 4.01545900 1.23041000 -2.21296700 |
|  |  |
|   B3LYP/LANL2DZ | Free Energy: -569.657495  Enthalpy: -569.601048  ZPE Corrected Energy: -569.614965  Total Energy: -569.7459644  C -2.20891100 -1.28489400 -0.14595800  C -3.37709600 -0.56091100 -0.20685200  C -3.42234900 0.86636500 -0.23952800  C -0.87934000 -0.80143500 -0.12644300  C -3.63523200 2.08225300 -0.23913400  H -3.70459700 3.14483700 -0.27352100  C 0.32204300 -0.46718600 -0.08960000  C -4.76602400 -1.20578600 -0.22034900  H -5.15194200 -1.23936000 -1.25254200  H -4.69685600 -2.24482600 0.13365500  C -5.74049200 -0.36221300 0.64378500  H -6.75256100 -0.80834400 0.58710000  H -5.43380600 -0.40334700 1.69825500  B -5.89883100 1.13710800 0.12601500  H -6.10758900 2.02671300 0.90535800  H -6.19547800 1.32338500 -1.02208000  H -2.32370200 -2.37319700 -0.10501600  Au 2.20773300 0.01252500 0.01336000  F 4.18824100 0.47799500 0.15419700 |
|   B3LYP/LANL2DZ | Free Energy: -484.787719  Enthalpy: -484.729559  ZPE Corrected Energy: -484.743902  Total Energy: -484.8741744  C -2.50343500 -1.31535400 -0.11110300  C -3.64123200 -0.54751700 -0.18394100  C -3.62594700 0.88121000 -0.21733400  C -1.15783800 -0.87332900 -0.08355500  C -3.77741300 2.10557800 -0.21974500  H -3.79746400 3.17044000 -0.25061700  C 0.04751100 -0.55724700 -0.05349000  C -5.05395300 -1.13707600 -0.21898800  H -5.41826400 -1.16412900 -1.25910300  H -5.03307900 -2.17498700 0.14395500  C -6.01254300 -0.24852000 0.61687800  H -7.04010200 -0.65411200 0.53947100  H -5.73276300 -0.29374300 1.67857800  B -6.09977600 1.25149400 0.08548600  H -6.29028600 2.15500000 0.85302400  H -6.35498300 1.44139500 -1.07163500  H -2.65578100 -2.39859600 -0.07037000  Au 1.94738300 -0.06621300 0.00494300  Cl 4.31337800 0.53209700 0.08508500 |
|   B3LYP/LANL2DZ | Free Energy: -483.011399  Enthalpy: -482.951843  ZPE Corrected Energy: -482.966420  Total Energy: -483.096344  C -3.00867300 -1.30974400 -0.09922900  C -4.12790900 -0.51598700 -0.17585100  C -4.07909600 0.91200100 -0.21386200  C -1.65293100 -0.89829600 -0.07315500  C -4.20002700 2.13955900 -0.22031600  H -4.19499400 3.20453900 -0.25389500  C -0.44070700 -0.60949800 -0.04732800  C -5.55363300 -1.07313600 -0.21221500  H -5.91538800 -1.09531800 -1.25330700  H -5.55736000 -2.11002400 0.15409500  C -6.49428500 -0.16036700 0.61792500  H -7.53039300 -0.54316800 0.53843800  H -6.21901900 -0.20850600 1.68064900  B -6.54695600 1.33927500 0.08147500  H -6.71795700 2.24974200 0.84521200  H -6.79330900 1.53106400 -1.07718200  H -3.18554500 -2.38902700 -0.05523000  Au 1.47657300 -0.16073500 -0.00376600  Br 3.98694500 0.41300600 0.05301600 |
|   B3LYP/LANL2DZ | Free Energy: -546.101891  Enthalpy: -546.040467  ZPE Corrected Energy: -546.056322  Total Energy: -546.210329  C -2.26772200 -1.33289500 -0.14487500  C -3.38668700 -0.54729400 -0.22290500  C -3.33042800 0.88416300 -0.27163200  C -0.91611200 -0.88487000 -0.11617700  C -3.40297400 2.11085900 -0.29598800  H -3.41797700 3.17636600 -0.33163500  C 0.27423400 -0.54740300 -0.07851400  C -4.80022800 -1.12880800 -0.24904700  H -5.18727000 -1.09086900 -1.27942300  H -4.76810600 -2.18624000 0.04748100  C -5.74751400 -0.30794400 0.66600000  H -6.75598400 -0.76470600 0.62123300  H -5.41970700 -0.37819200 1.71160300  B -5.95941700 1.18953700 0.19377200  H -6.09113900 2.07758000 0.98654300  H -6.21831000 1.41231100 -0.95440900  H -2.41648300 -2.41349100 -0.09708400  Au 2.13062800 -0.01583000 0.01420700  O 4.22005500 0.57109000 0.14268300  H 4.51319100 1.49686700 0.09799300  H 4.94336200 -0.07033700 0.24380600 |
|   B3LYP/LANL2DZ | Free Energy: -480.975127  Enthalpy: -480.912727  ZPE Corrected Energy: -480.928652  Total Energy: -481.0756997  C -2.55715400 -1.33655300 -0.10759200  C -3.65519600 -0.52360700 -0.19803000  C -3.56068300 0.90539600 -0.26221100  C -1.19519600 -0.91879800 -0.08420700  C -3.59737700 2.13307400 -0.30064400  H -3.58344200 3.19820400 -0.34839200  C 0.00230200 -0.60477300 -0.05913400  C -5.08237900 -1.07007100 -0.22775300  H -5.45903000 -1.03457400 -1.26203600  H -5.07852800 -2.12452600 0.08058700  C -6.01758200 -0.21629300 0.66912600  H -7.03561500 -0.65108500 0.62159800  H -5.70041800 -0.28069400 1.71834900  B -6.19377900 1.27887100 0.17663700  H -6.31207000 2.17963200 0.95691400  H -6.43339200 1.49345300 -0.97714300  H -2.73163700 -2.41254000 -0.04847900  Au 1.88369600 -0.09603900 -0.00935300  S 4.34076400 0.55830400 0.04430100  H 4.75683100 -0.10926100 1.17475500  H 4.26158900 1.79096900 0.65337800 |
|   B3LYP/LANL2DZ | Total Energy: -596.0380174  Enthalpy: -595.771325  Free energy: -595.844816  ZPE Correction: -595.792222  C -3.28759800 -1.33197700 -0.10229000  C -4.35912700 -0.48327700 -0.19326200  C -4.21980000 0.94168700 -0.25728900  C -1.91242500 -0.96363400 -0.07962600  C -4.22393100 2.17045900 -0.29421200  H -4.17641500 3.23452500 -0.34068800  C -0.70176400 -0.69112700 -0.05503100  C -5.80458600 -0.98042900 -0.22344800  H -6.17779900 -0.94117200 -1.25899500  H -5.83881800 -2.03168400 0.09425700  C -6.71092500 -0.08538700 0.66277500  H -7.74553000 -0.47853400 0.61187300  H -6.40180400 -0.15591400 1.71409400  B -6.82016400 1.41366600 0.16039900  H -6.91069000 2.32231900 0.93567800  H -7.05144000 1.63053800 -0.99490200  H -3.50167400 -2.40122400 -0.04220300  Au 1.22203100 -0.23646800 -0.01003100  P 3.58639000 0.32638800 0.04729800  C 4.75387100 -1.13063200 -0.18646200  H 5.79855900 -0.80043800 -0.15350300  H 4.57865700 -1.86639300 0.60430800  H 4.55208100 -1.60618500 -1.15108400  C 4.17325200 1.10969300 1.65429200  H 3.98351900 0.42180700 2.48387500  H 5.24420400 1.33836300 1.60671200  H 3.61109300 2.03090600 1.83498200  C 4.13989900 1.55121600 -1.26950300  H 5.21258300 1.75868800 -1.18077100  H 3.92908600 1.13830400 -2.26067100  H 3.57774600 2.48333200 -1.15765500 |
|   B3LYP/LANL2DZ | Free Energy: -703.644084  Enthalpy: -703.572121  ZPE Corrected Energy: -703.592042  Total Energy: -703.764626  C 3.26772300 -1.34533600 0.09915700  C 4.33292500 -0.49151900 0.20641500  C 4.17992200 0.93247800 0.27134700  C 1.89344900 -0.97229800 0.05880100  C 4.15867900 2.16013400 0.31114200  H 4.09530700 3.22379600 0.35487200  C 0.68848900 -0.68709300 0.01791000  C 5.77857900 -0.98384100 0.25713100  H 6.13482800 -0.93972800 1.29830600  H 5.81978300 -2.03605500 -0.05590800  C 6.69716000 -0.09076400 -0.61814100  H 7.72906200 -0.48945500 -0.55593400  H 6.40100200 -0.15896400 -1.67316800  B 6.81256100 1.40571500 -0.11295100  H 6.91413100 2.31553800 -0.88461000  H 7.01684300 1.62070100 1.04749800  H 3.48046000 -2.41412800 0.03949200  Au -1.21108400 -0.21284600 -0.04827100  P -3.57254500 0.32343200 -0.02160400  O -3.92323200 1.93534300 -0.39692300  H -4.85299100 2.19003300 -0.58532000  O -4.69855700 -0.49317500 -1.00777700  H -5.29368800 -1.13457200 -0.56342300  O -4.41348800 -0.00283700 1.44938900  H -3.94305400 0.11236500 2.30036400 |
|   B3LYP/LANL2DZ | Free Energy: -644.003701  Enthalpy: -643.931693  ZPE Corrected Energy: -643.951792  Total Energy: -644.1647701  C -3.27811400 -1.31949100 -0.14165800  C -4.34764500 -0.46604600 -0.20310900  C -4.20400900 0.95997800 -0.22106500  C -1.90241700 -0.95266900 -0.10939300  C -4.20082300 2.18907800 -0.21860500  H -4.14880100 3.25395200 -0.23040600  C -0.69302400 -0.67845300 -0.07730600  C -5.79381600 -0.95932200 -0.24778900  H -6.16804600 -0.88265000 -1.28081900  H -5.82927100 -2.02114400 0.03234300  C -6.69773100 -0.09525700 0.67101000  H -7.73229400 -0.48688400 0.60882900  H -6.38656400 -0.20219800 1.71861700  B -6.80966700 1.41978700 0.22080300  H -6.89642500 2.30167800 1.02658500  H -7.04155100 1.67602400 -0.92620000  H -3.49267500 -2.38984600 -0.11583200  Au 1.22811800 -0.23222100 -0.02122100  P 3.59459800 0.31130100 0.05348400  N 4.23525300 1.79976200 0.70670000  H 4.23700700 1.92166900 1.71318900  H 4.13199700 2.64802500 0.16112800  N 4.45581500 -0.78435500 1.07357300  H 4.03313000 -1.66684200 1.33194400  H 5.43490800 -0.65370200 1.30211400  N 4.26489200 0.44210200 -1.53280300  H 5.21622000 0.75375000 -1.69331800  H 3.76416000 0.05568800 -2.32280200 |
|  |  |
|   B3LYP/LANL2DZ | Free Energy: -768.322015  Enthalpy: -768.258526  ZPE Corrected Energy: -768.274439  Total Energy: -768.3937235  C -1.52446200 -0.75874100 -0.52141900  C -2.56032000 0.15127100 -0.50810100  C -2.35267200 1.55342800 -0.30777100  C -0.14298800 -0.50895900 -0.34858500  C -2.24012300 2.76584800 -0.14158900  H -2.07478000 3.80878900 -0.00016900  C 1.08617700 -0.35877400 -0.19822300  C -4.00554900 -0.29710600 -0.70678000  H -4.43702100 0.19003800 -1.59403100  H -4.02477500 -1.37832100 -0.89920600  C -4.90474300 0.03123600 0.51828600  H -4.51379700 -0.44772500 1.42852600  H -4.82971800 1.11422000 0.72487500  B -6.40379100 -0.31235800 0.35319600  F -7.01597300 -0.52552500 -0.86024000  F -7.26737300 -0.42476700 1.42283300  H -1.80679200 -1.80440300 -0.68653000  Au 3.00153300 -0.10568100 0.05641700  F 5.00032700 0.16793400 0.33615100 |
|   B3LYP/LANL2DZ | Free Energy: -683.452147  Enthalpy: -683.386893  ZPE Corrected Energy: -683.403262  Total Energy: -683.5217866  C -1.81086900 -0.77884800 -0.50135000  C -2.84202700 0.13446300 -0.51294400  C -2.62613300 1.54218300 -0.36328900  C -0.42628800 -0.52527200 -0.34594700  C -2.50400600 2.75849300 -0.24097200  H -2.33214300 3.80488000 -0.13714900  C 0.80346900 -0.37484500 -0.20982900  C -4.29033900 -0.31415000 -0.68671300  H -4.72375600 0.14332500 -1.58860400  H -4.31595000 -1.40121400 -0.84086600  C -5.18009800 0.06156600 0.53043900  H -4.78539300 -0.38727300 1.45472500  H -5.10153500 1.15012700 0.70196000  B -6.68109400 -0.28575700 0.38882000  F -7.29075300 -0.59661300 -0.80416100  F -7.54675600 -0.30313100 1.46211100  H -2.09514100 -1.82892400 -0.62779300  Au 2.73751600 -0.12718700 0.02134200  Cl 5.13476000 0.18069500 0.32174700 |
|   B3LYP/LANL2DZ | Free Energy: -681.676018  Enthalpy: -681.609118  ZPE Corrected Energy: -681.625753  Total Energy: -681.7438958  C -2.33286200 -0.77831000 -0.48224600  C -3.36331100 0.13503600 -0.50345400  C -3.14651300 1.54491700 -0.37623200  C -0.94669300 -0.52392700 -0.33887300  C -3.02368300 2.76283000 -0.27293500  H -2.85197300 3.81079900 -0.18597400  C 0.28515500 -0.37872400 -0.21752800  C -4.81214700 -0.31595900 -0.66641200  H -5.24698200 0.12708800 -1.57478200  H -4.83825100 -1.40528500 -0.80332400  C -5.69952900 0.07915300 0.54600000  H -5.30312900 -0.35485700 1.47668200  H -5.62167200 1.17024700 0.70053500  B -7.20051200 -0.27207100 0.41267500  F -7.80983400 -0.61181400 -0.77244900  F -8.06580100 -0.26405500 1.48624700  H -2.61777200 -1.83010200 -0.59096000  Au 2.23184500 -0.15173400 -0.02240300  Br 4.77956500 0.13034700 0.23229100 |
|   B3LYP/LANL2DZ | Free Energy: -744.764581  Enthalpy: -744.697114  ZPE Corrected Energy: -744.714745  Total Energy: -744.8574116  C -1.57226600 -0.85802700 -0.45630400  C -2.57925900 0.07016000 -0.52732300  C -2.32629100 1.48066800 -0.46759600  C -0.18141300 -0.59071300 -0.30980800  C -2.16887800 2.69636800 -0.42714100  H -2.00033000 3.74858700 -0.39437600  C 1.03585600 -0.41050300 -0.17593400  C -4.03640100 -0.35585400 -0.67557900  H -4.45034600 0.05988100 -1.60526200  H -4.08799400 -1.44801800 -0.76920400  C -4.91673400 0.10999100 0.51315700  H -4.53336200 -0.29305900 1.46366500  H -4.83488000 1.20405900 0.62910300  B -6.42243100 -0.24290800 0.39075800  F -6.98878800 -0.77413100 -0.74011200  F -7.30726600 -0.04009800 1.42206700  H -1.85482300 -1.91126400 -0.51578600  Au 2.93053900 -0.10440900 0.05941000  O 5.04626500 0.24066900 0.34352400  H 5.70881600 -0.46962500 0.30597700  H 5.41917800 1.12242700 0.51211700 |
|   B3LYP/LANL2DZ | Free Energy: -679.638554  Enthalpy: -679.569170  ZPE Corrected Energy: -679.587000  Total Energy: -679.7225679  C -1.85900000 -0.88854600 -0.40805400  C -2.85639500 0.04634400 -0.51242500  C -2.58885300 1.45547100 -0.50085800  C -0.46533500 -0.62656100 -0.27311800  C -2.41841500 2.66993100 -0.50084900  H -2.24036000 3.72116800 -0.50173000  C 0.75429800 -0.45078300 -0.15225800  C -4.31717400 -0.37002800 -0.65049100  H -4.72128500 0.01334400 -1.59824800  H -4.37998500 -1.46430200 -0.70198500  C -5.19886900 0.15109400 0.51365700  H -4.82514200 -0.21857500 1.48150300  H -5.10809200 1.24787700 0.58820900  B -6.70700300 -0.19433700 0.39625000  F -7.26680300 -0.78421200 -0.70829500  F -7.59891400 0.07503200 1.40550500  H -2.15019300 -1.94067300 -0.42996300  Au 2.67003200 -0.14526900 0.04390700  S 5.16706100 0.24920600 0.32116700  H 5.61647500 -0.05159600 -0.94548800  H 5.22803900 1.61446600 0.15056600 |
|   B3LYP/LANL2DZ | Free Energy: -794.508296  Enthalpy: -794.427762  ZPE Corrected Energy: -794.450581  Total Energy: -794.6849018  C -2.60584100 -0.90692800 -0.39930400  C -3.59643100 0.03491500 -0.51625000  C -3.31709900 1.44124900 -0.54608900  C -1.20803100 -0.66115900 -0.28969500  C -3.13832700 2.65431800 -0.58048500  H -2.94955800 3.70306800 -0.61229900  C 0.01843800 -0.49896900 -0.19054800  C -5.06279600 -0.37162000 -0.62426300  H -5.47698600 -0.01246000 -1.57729000  H -5.13533900 -1.46644000 -0.64307100  C -5.92492300 0.19045300 0.53565200  H -5.53810900 -0.15076700 1.50888300  H -5.82564900 1.28844700 0.57436000  B -7.43640500 -0.14818300 0.45339200  F -8.02052800 -0.76643600 -0.62300700  F -8.31029000 0.15564100 1.46957600  H -2.91190000 -1.95556200 -0.38984500  Au 1.96775600 -0.20880400 -0.02862100  P 4.36268300 0.15636300 0.17335900  C 4.87989500 1.18040600 1.66492200  H 5.96844800 1.30652700 1.69090300  H 4.39968500 2.16234000 1.61515000  H 4.54885900 0.68077700 2.58044300  C 5.39501500 -1.40856700 0.33193700  H 5.23457500 -2.04040000 -0.54691400  H 6.46019100 -1.16402300 0.41609000  H 5.07719800 -1.96431100 1.21931100  C 5.14858400 1.05996100 -1.27815200  H 4.67661700 2.04035700 -1.39380500  H 6.22529700 1.18950500 -1.11847500  H 4.98221200 0.48676300 -2.19523100 |
|   B3LYP/LANL2DZ | Free Energy: -902.308131  Enthalpy: -902.228314  ZPE Corrected Energy: -902.250239  Total Energy: -902.4112725  C -2.58826300 -0.92571000 -0.38496100  C -3.57784900 0.01394100 -0.51758500  C -3.29386300 1.41856300 -0.57348200  C -1.19152700 -0.66607400 -0.28189600  C -3.10239900 2.62828000 -0.63015700  H -2.90496400 3.67502600 -0.68053000  C 0.02967200 -0.47996200 -0.18845900  C -5.04389400 -0.39343200 -0.61727100  H -5.45441200 -0.05496300 -1.57925200  H -5.11862200 -1.48804300 -0.61161100  C -5.90616400 0.19714900 0.52779000  H -5.51873500 -0.11846200 1.50950100  H -5.81281100 1.29587300 0.53958100  B -7.41612600 -0.15377700 0.45512300  F -7.98717900 -0.83507200 -0.58910400  F -8.29600300 0.20082600 1.44797500  H -2.88732000 -1.97519800 -0.35581100  Au 1.95553500 -0.15513500 -0.03114200  P 4.35185500 0.13766300 0.18226800  O 5.22381600 -1.24270400 0.73508100  H 4.78960100 -1.84309400 1.37581100  O 4.82594000 1.42064400 1.17208200  H 5.76244500 1.71617000 1.13754000  O 5.36604200 0.44948500 -1.15355300  H 5.72830000 -0.31544300 -1.64720200 |
|   B3LYP/LANL2DZ | Free Energy: -842.667316  Enthalpy: -842.588139  ZPE Corrected Energy: -842.610170  Total Energy: -842.8116479  C -2.59517300 -0.87841800 -0.45244300  C -3.58653000 0.06783600 -0.50765500  C -3.30760500 1.47340500 -0.44939800  C -1.19751100 -0.63619000 -0.33022200  C -3.12760500 2.68593800 -0.40821500  H -2.93843100 3.73461500 -0.37436400  C 0.02757900 -0.47532200 -0.22103100  C -5.05283200 -0.33176500 -0.63714900  H -5.47075800 0.09186000 -1.56150900  H -5.12528100 -1.42276300 -0.73054400  C -5.91014700 0.14920800 0.56225400  H -5.52297900 -0.26141100 1.50794200  H -5.80712000 1.24154000 0.67770900  B -7.42329500 -0.17625600 0.45719200  F -8.01258000 -0.69497400 -0.66783500  F -8.29233000 0.04038100 1.49912900  H -2.89859300 -1.92618200 -0.50815900  Au 1.97329100 -0.19834300 -0.04525600  P 4.36960600 0.13643900 0.17531500  N 5.13547700 0.81409800 1.59168400  H 5.20178200 0.23967400 2.42445600  H 5.05472500 1.81209100 1.75088700  N 4.95691200 1.27814900 -0.97960900  H 5.91573300 1.60770700 -0.97399600  H 4.39101800 1.51322200 -1.78503800  N 5.21602300 -1.36865500 0.14522600  H 4.75420500 -2.19636100 -0.20939800  H 6.21234200 -1.43215200 0.32196600 |
|  |  |
|   B3LYP/LANL2DZ | Free Energy: -598.468533  Enthalpy: -598.404250  ZPE Corrected Energy: -598.420656  Total Energy: -598.537125  C -0.83665400 1.66912900 -0.37042600  C -2.04955800 1.11324600 -0.02299900  C -2.17320300 -0.20568400 0.51332500  C 0.44877600 1.09362600 -0.24960800  C -2.36005400 -1.33527100 0.96142300  H -2.47198600 -2.31895500 1.35520800  C 1.60782500 0.64060300 -0.16116300  C -3.35531800 1.88131900 -0.20424800  H -3.78526100 2.15244000 0.77171700  H -3.14873400 2.81866900 -0.73771800  C -4.41769000 1.06442900 -1.00403600  H -5.24992100 1.74727000 -1.26109300  H -3.98562600 0.71957400 -1.95156000  B -5.08813200 -0.12701200 -0.24881600  H -0.87423400 2.68202200 -0.78607200  Au 3.40326800 -0.09716500 0.00047500  Cl -5.50634600 -1.68723900 -1.09658400  Cl -5.81869400 0.09831300 1.41074300  F 5.27102000 -0.88961800 0.18908800 |
|   B3LYP/LANL2DZ | Free Energy: -513.598777  Enthalpy: -513.532588  ZPE Corrected Energy: -513.549433  Total Energy: -513.6651829  C -1.13493400 1.64846200 -0.35483900  C -2.34339000 1.08752700 -0.00582900  C -2.45963600 -0.22898000 0.53966200  C 0.15675400 1.08377000 -0.23024400  C -2.63702100 -1.35607900 0.99654300  H -2.74338300 -2.33763200 1.39733200  C 1.32611400 0.65973800 -0.14842600  C -3.65186800 1.84899800 -0.19511900  H -4.08866700 2.11608600 0.77868800  H -3.44549400 2.78823400 -0.72505400  C -4.70559800 1.03026700 -1.00445400  H -5.53357900 1.71399300 -1.27250300  H -4.26383000 0.68307200 -1.94658800  B -5.38718200 -0.15750000 -0.25380200  H -1.17665400 2.65838800 -0.77642500  Au 3.17085300 0.00224900 -0.01736400  Cl 5.47037200 -0.80018300 0.14545400  Cl -5.78969400 -1.72229400 -1.09755100  Cl -6.12846600 0.07220900 1.39924900 |
|   B3LYP/LANL2DZ | Free Energy: -511.822401  Enthalpy: -511.754831  ZPE Corrected Energy: -511.771910  Total Energy: -511.8873174  C -1.67869200 1.68992300 -0.33151500  C -2.87172800 1.09594400 0.01463000  C -2.95363600 -0.22839100 0.54761400  C -0.37298400 1.15509700 -0.21642900  C -3.10065500 -1.36381600 0.99422200  H -3.18112600 -2.35159300 1.38571500  C 0.80657500 0.75851900 -0.14487700  C -4.19895900 1.82666200 -0.16533900  H -4.64263800 2.06920600 0.81168700  H -4.01563400 2.77780300 -0.68225000  C -5.23133900 0.99302200 -0.98683700  H -6.07440000 1.66049200 -1.24887900  H -4.77968600 0.66764300 -1.93204700  B -5.88741300 -0.21895300 -0.25234300  H -1.74575700 2.70278900 -0.74236700  Au 2.67400400 0.14300600 -0.03606200  Cl -6.24824800 -1.78283800 -1.11574100  Cl -6.63552200 -0.02776100 1.40196300  Br 5.12179000 -0.64316200 0.09669000 |
|   B3LYP/LANL2DZ | Free Energy: -574.910605  Enthalpy: -574.842487  ZPE Corrected Energy: -574.860553  Total Energy: -575.0005054  C -0.88896100 1.74566900 -0.34613100  C -2.06761600 1.14653300 0.01853100  C -2.11597600 -0.18560400 0.54442000  C 0.41122800 1.17470400 -0.24773300  C -2.21449100 -1.32387800 0.99051800  H -2.28549400 -2.31412300 1.37941500  C 1.55597800 0.71029000 -0.17076800  C -3.39983200 1.87653000 -0.11996300  H -3.82305200 2.07495200 0.87485800  H -3.22467500 2.84913100 -0.59664600  C -4.44396500 1.07915200 -0.96055100  H -5.27692700 1.76836400 -1.19404100  H -4.00663600 0.78246300 -1.92162000  B -5.11879300 -0.14695000 -0.26320900  H -0.94182900 2.76039300 -0.74602400  Au 3.31780600 -0.07006200 -0.01228300  Cl -5.45208700 -1.68821900 -1.16042700  Cl -5.82257500 -0.00631600 1.40730300  O 5.27471600 -0.97285900 0.19525000  H 5.42550100 -1.78113500 0.71381400  H 6.07369800 -0.62078300 -0.23213700 |
|   B3LYP/LANL2DZ | Free Energy: -509.784698  Enthalpy: -509.714562  ZPE Corrected Energy: -509.732828  Total Energy: -509.86568  C -1.19053800 1.77422700 -0.31095100  C -2.35548000 1.14734000 0.04959400  C -2.37572400 -0.19302000 0.55650200  C 0.12086000 1.22533800 -0.22763400  C -2.45001800 -1.33866700 0.98769500  H -2.50183300 -2.33521500 1.36348500  C 1.27706200 0.78652400 -0.16787700  C -3.70176000 1.85351900 -0.07388000  H -4.12782500 2.02211200 0.92511600  H -3.54612700 2.83924600 -0.52979000  C -4.73051800 1.05388000 -0.93111900  H -5.57536100 1.73281100 -1.15135200  H -4.28770400 0.78460200 -1.89771000  B -5.38411300 -0.19751100 -0.25865700  H -1.26321500 2.79340800 -0.69561100  Au 3.07953700 0.05285500 -0.04569600  Cl -5.68308100 -1.72835800 -1.18398400  Cl -6.09132500 -0.10142300 1.41315700  S 5.42326000 -0.91811500 0.12899200  H 5.13410500 -2.26451000 0.14895300  H 5.63455100 -0.81912600 1.48637200 |
|   B3LYP/LANL2DZ | Free Energy: -624.654702  Enthalpy: -624.573280  ZPE Corrected Energy: -624.596564  Total Energy: -624.828119  C 1.96534700 -1.82003800 -0.29003700  C 3.10920500 -1.15251800 0.06788500  C 3.08741300 0.19283200 0.56049300  C 0.63509800 -1.31965400 -0.21871000  C 3.12871500 1.34483500 0.97986400  H 3.14832000 2.34613800 1.34555600  C -0.54064100 -0.92534600 -0.16888300  C 4.47783600 -1.81645600 -0.04398000  H 4.90909400 -1.95813600 0.95705600  H 4.35541300 -2.81271500 -0.48733000  C 5.48129500 -0.99524000 -0.91127500  H 6.35003300 -1.64748100 -1.11918100  H 5.03115600 -0.75571300 -1.88233300  B 6.08836200 0.28796700 -0.25625200  H 2.07681700 -2.84042400 -0.66348900  Au -2.40172700 -0.26461200 -0.06478500  Cl 6.33379200 1.81683000 -1.20215300  Cl 6.80582600 0.23846200 1.41415600  P -4.68504000 0.55781200 0.07998700  C -5.99776100 -0.61818200 -0.57883600  H -5.95145600 -1.56052200 -0.02454900  H -6.99782500 -0.18181100 -0.47438000  H -5.80030500 -0.82831200 -1.63438800  C -5.00933100 2.15662700 -0.85754800  H -6.05312700 2.47034900 -0.74114700  H -4.34831800 2.94081100 -0.47613700  H -4.78955800 2.00649600 -1.91889400  C -5.27076800 0.94205400 1.82621000  H -6.30121900 1.31576600 1.81711200  H -5.21711300 0.03426000 2.43484200  H -4.61369000 1.69514600 2.27160900 |
|   B3LYP/LANL2DZ | Free Energy: -732.454166  Enthalpy: -732.373603  ZPE Corrected Energy: -732.395912  Total Energy: -732.5543026  C 1.94552200 -1.81153300 -0.30700500  C 3.09060500 -1.14941200 0.05366900  C 3.06855900 0.19329500 0.55396700  C 0.61965200 -1.29768900 -0.22944500  C 3.10028800 1.34272300 0.97953700  H 3.11380100 2.34275700 1.34989100  C -0.54688700 -0.88456900 -0.17351600  C 4.45632400 -1.81801500 -0.06193100  H 4.88165600 -1.97078600 0.93985500  H 4.33052200 -2.80949100 -0.51456600  C 5.46591000 -0.99277900 -0.91702900  H 6.32820900 -1.65011000 -1.13491400  H 5.01940700 -0.73534800 -1.88515100  B 6.08676800 0.27514000 -0.24375900  H 2.04659300 -2.83007900 -0.68623300  Au -2.38293800 -0.20700200 -0.07283900  Cl 6.35450100 1.80942700 -1.17083700  Cl 6.78277700 0.19897300 1.43327600  P -4.68941800 0.52375400 0.07567000  O -5.13756700 1.74011800 -1.00453700  H -5.98414500 2.21334400 -0.84606100  O -5.34390400 1.20474200 1.49592700  H -5.62651900 0.60489200 2.21666600  O -5.86974400 -0.72086800 -0.08715400  H -5.67379800 -1.47085300 -0.68625400 |
|   B3LYP/LANL2DZ | Free Energy: -672.813489  Enthalpy: -672.733641  ZPE Corrected Energy: -672.756124  Total Energy: -672.9548429  C 1.95215600 -1.80883700 -0.34653000  C 3.09726400 -1.15443400 0.03002900  C 3.07698400 0.17273000 0.57006500  C 0.62274600 -1.30906300 -0.25272800  C 3.11793500 1.30915900 1.02970300  H 3.13821500 2.29698400 1.43051100  C -0.55180600 -0.91576900 -0.18692500  C 4.46503400 -1.81434600 -0.11189600  H 4.89985300 -1.99300300 0.88159600  H 4.33977100 -2.79350700 -0.59091200  C 5.46498300 -0.96225300 -0.95273600  H 6.33012300 -1.60827900 -1.19266400  H 5.00939700 -0.68331100 -1.91062300  B 6.08119300 0.29229800 -0.25209800  H 2.06030800 -2.81539400 -0.75621000  Au -2.40744000 -0.25548700 -0.06496700  Cl 6.33035700 1.85380900 -1.14134100  Cl 6.80061100 0.17750600 1.41373300  P -4.68839600 0.56507400 0.09215500  N -5.01560000 1.74977500 -1.12111900  H -4.24728300 2.20211400 -1.60004200  H -5.94251800 2.13488600 -1.26429100  N -5.05053900 1.10244000 1.69291700  H -5.98301000 1.38978800 1.96843700  H -4.29626800 1.29556600 2.33952400  N -6.10435500 -0.42779500 -0.15348000  H -6.36213300 -1.08651900 0.57275000  H -6.34347600 -0.69989300 -1.10032400 |
|  |  |
|   B3LYP/LANL2DZ | Total Energy: -594.9609181  Enthalpy: -594.828373  Free energy: -594.897086  ZPE Correction: -594.845378  C -0.20988700 0.12971400 -0.72173600  C 0.78234300 -0.75392300 -0.34876900  C 0.51980200 -1.95006300 0.39100200  C -1.59825400 0.04411600 -0.47440800  C 0.35547900 -2.99094400 1.02259800  H 0.15539000 -3.88561500 1.56528500  C -2.83303700 0.06525900 -0.29546600  C 2.23893800 -0.47119000 -0.69081800  H 2.68960900 -1.33188700 -1.20385000  H 2.29154900 0.38963800 -1.37017200  C 3.09335700 -0.16884500 0.60956400  H 2.61929700 0.66421400 1.14237000  H 3.02829800 -1.06183400 1.24783500  B 4.57322400 0.14885000 0.26685500  Br 5.84420500 -1.29530900 -0.23725600  Br 5.26616200 2.00937900 0.22840300  H 0.12083500 1.01116500 -1.28225100  Au -4.75727700 0.15656100 -0.0088590  F -6.76683600 0.29828500 0.29159600 |
|   B3LYP/LANL2DZ | Free Energy: -510.024159  Enthalpy: -509.957665  ZPE Corrected Energy: -509.974225  Total Energy: -510.0885742  C 0.00746600 -0.54222300 -0.73684500  C 1.00563400 -0.71328500 0.19743900  C 0.74845100 -0.73312600 1.60580700  C -1.37809100 -0.36660600 -0.50474700  C 0.59198900 -0.75773100 2.82406600  H 0.39135600 -0.77761500 3.87030000  C -2.60657100 -0.20306900 -0.37121400  C 2.46129900 -0.88285900 -0.22680500  H 2.85659400 -1.84065300 0.13985900  H 2.51962300 -0.90957800 -1.32292000  C 3.36497400 0.26838100 0.31194900  H 2.96271300 1.23961700 -0.01188100  H 3.26716000 0.28328000 1.41568200  B 4.88834900 0.20304800 0.00753400  Br 5.85378100 -1.49874700 -0.32022100  Br 5.98602400 1.86266200 -0.01881400  H 0.32277400 -0.54296200 -1.78565800  Au -4.53790900 0.06775800 -0.14522000  Cl -6.93125100 0.41328400 0.14103500 |
|   B3LYP/LANL2DZ | Free Energy: -508.254296  Enthalpy: -508.178948  ZPE Corrected Energy: -508.196711  Total Energy: -508.310668  C 0.54957000 -0.65229100 -0.63787700  C 1.55110000 -0.68731400 0.30709000  C 1.29810100 -0.51654500 1.70615100  C -0.83730000 -0.45559100 -0.42837200  C 1.14521900 -0.37738400 2.91706900  H 0.94823700 -0.25719600 3.95728200  C -2.06779600 -0.28775200 -0.32264800  C 3.00641100 -0.90797000 -0.09544100  H 3.40506400 -1.80566000 0.39799800  H 3.06137400 -1.08615000 -1.17738300  C 3.90743100 0.30694000 0.27604000  H 3.50306000 1.22398100 -0.17930900  H 3.81176200 0.48182500 1.36591900  B 5.43075600 0.21074700 -0.02193700  Br 6.38389600 -1.51009300 -0.26911700  Br 6.53847600 1.86108200 -0.12789600  H 0.86142100 -0.79578900 -1.67775400  Au -4.01165900 -0.01735600 -0.15841300  Br -6.55494500 0.33540600 0.04288300 |
|   B3LYP/LANL2DZ | Free Energy: -571.337591  Enthalpy: -571.267851  ZPE Corrected Energy: -571.285724  Total Energy: -571.4241359  C -0.23281900 -0.78866900 -0.50597000  C 0.73990300 -0.45280100 0.40088400  C 0.44380000 0.25643900 1.61190800  C -1.62454300 -0.50861200 -0.39285800  C 0.24737700 0.85852300 2.66221900  H 0.04514300 1.37962900 3.57008200  C -2.84207500 -0.29624000 -0.32585400  C 2.19930400 -0.83406300 0.17117800  H 2.53854000 -1.48866900 0.98599300  H 2.28251300 -1.41361900 -0.75648200  C 3.13488400 0.40231000 0.10046300  H 2.80171000 1.08080800 -0.70195900  H 3.01449000 0.99986800 1.02247900  B 4.66626800 0.14987000 -0.07687000  Br 5.48479900 -1.64494100 0.02357600  Br 5.87635200 1.68604900 -0.39800500  H 0.07958000 -1.33098800 -1.40084100  Au -4.74004500 0.03363600 -0.15655500  O -6.86260200 0.39352600 0.07054300  H -7.27499400 1.25794300 -0.09591800  H -7.48926400 -0.29989700 0.33770600 |
|   B3LYP/LANL2DZ | Free Energy: -506.214216  Enthalpy: -506.139085  ZPE Corrected Energy: -506.158048  Total Energy: -506.2893811  C 0.05428100 -0.83757400 -0.43303300  C 1.02500400 -0.46768600 0.46204700  C 0.72582100 0.27445500 1.65242700  C -1.33953700 -0.56213500 -0.33039600  C 0.52781900 0.90464100 2.68569500  H 0.32711900 1.45000200 3.57959100  C -2.55933300 -0.35471800 -0.28453400  C 2.48716700 -0.84101700 0.23772000  H 2.83908300 -1.46024200 1.07446800  H 2.57120400 -1.45431700 -0.66776900  C 3.40699900 0.40271400 0.11349500  H 3.05657700 1.04850700 -0.70873300  H 3.29173400 1.03191600 1.01444200  B 4.93875800 0.16135200 -0.07956900  Br 5.77182300 -1.62633100 0.01727500  Br 6.13056800 1.70604100 -0.42418900  H 0.36892400 -1.40423300 -1.31170600  Au -4.47759900 -0.02207900 -0.17759900  S -6.98046500 0.40382100 -0.03756700  H -6.99396700 1.64675400 0.55556200  H -7.28965600 -0.28398900 1.11487100 |
|   B3LYP/LANL2DZ | Free Energy: -621.083652  Enthalpy: -620.997683  ZPE Corrected Energy: -621.021622  Total Energy: -621.2517066  C 0.81771 -0.87214 -0.41978  C 1.78904 -0.50315 0.47620  C 1.48858 0.21873 1.67836  C -0.57876 -0.61755 -0.31347  C 1.29119 0.83271 2.72179  H 1.08736 1.36318 3.62376  C -1.80427 -0.42697 -0.26618  C 3.25460 -0.85530 0.23847  H 3.62071 -1.48206 1.06356  H 3.34071 -1.45389 -0.67674  C 4.15805 0.40194 0.12668  H 3.79137 1.05673 -0.68128  H 4.04114 1.01442 1.03906  B 5.69046 0.18506 -0.08276  Br 6.55182 -1.59117 -0.01307  Br 6.85870 1.75031 -0.42222  H 1.13950 -1.42296 -1.30656  Au -3.75452 -0.11663 -0.16421  P -6.15192 0.26267 -0.02900  C -6.69423 2.02969 -0.38002  H -7.78333 2.12438 -0.30105  H -6.21635 2.70521 0.33593  H -6.37570 2.31340 -1.38766  C -6.91246 -0.10927 1.65166  H -7.99117 0.08552 1.64269  H -6.73333 -1.15828 1.90635  H -6.43523 0.51635 2.41204  C -7.18754 -0.76188 -1.21995  H -7.01319 -1.82541 -1.03072  H -8.25386 -0.53933 -1.09748  H -6.88461 -0.53992 -2.24773 |
|   B3LYP/LANL2DZ | Free Energy: -728.883740  Enthalpy: -728.798403  ZPE Corrected Energy: -728.821456  Total Energy: -728.9781954  C 0.79081 -0.50482 -0.91606  C 1.78475 -0.72450 0.00335  C 1.50613 -0.90663 1.39810  C -0.60147 -0.39184 -0.64174  C 1.30819 -1.07404 2.59703  H 1.09550 -1.22362 3.63129  C -1.82147 -0.28097 -0.45773  C 3.24956 -0.79841 -0.41912  H 3.64544 -1.79537 -0.18341  H 3.32076 -0.67564 -1.50762  C 4.12460 0.27682 0.28527  H 3.72595 1.28110 0.09465  H 4.02040 0.13483 1.38067  B 5.66449 0.24172 0.02335  Br 6.66206 -1.44658 -0.23131  Br 6.71503 1.91856 0.05475  H 1.08549 -0.37339 -1.95890  Au -3.74879 -0.05132 -0.18692  P -6.13405 0.27411 0.11877  O -6.69614 1.76047 0.75403  H -6.60634 2.57832 0.22398  O -6.86027 -0.73098 1.27667  H -7.61974 -0.36193 1.78002  O -7.13662 0.18746 -1.27273  H -7.52553 -0.65919 -1.57058 |
|   B3LYP/LANL2DZ | Free Energy: -669.239159  Enthalpy: -669.159007  ZPE Corrected Energy: -669.181222  Total Energy: -669.3784552  C 0.80978 -0.86733 -0.41904  C 1.77944 -0.48844 0.47421  C 1.47637 0.24813 1.66682  C -0.58670 -0.61007 -0.31544  C 1.27493 0.87480 2.70173  H 1.06831 1.41690 3.59620  C -1.81095 -0.41605 -0.26776  C 3.24497 -0.84486 0.24393  H 3.60813 -1.46125 1.07803  H 3.33241 -1.45555 -0.66311  C 4.15008 0.40966 0.11781  H 3.78668 1.05405 -0.69988  H 4.03232 1.03454 1.02157  B 5.68293 0.18722 -0.08490  Br 6.53995 -1.58973 0.01017  Br 6.85438 1.74566 -0.44119  H 1.13110 -1.42859 -1.29917  Au -3.75794 -0.11341 -0.16344  P -6.15655 0.24591 -0.02500  N -6.92241 1.81241 -0.13226  H -6.98240 2.25769 -1.04108  H -6.84910 2.43932 0.66110  N -6.76700 -0.20989 1.52494  H -7.72873 -0.04093 1.79788  H -6.21052 -0.80100 2.12921  N -6.98375 -0.51088 -1.33882  H -6.51506 -1.22458 -1.88209  H -7.97865 -0.38968 -1.49209 |
|  |  |
|   B3LYP/LANL2DZ | Total Energy: -754.2326793  Enthalpy: -754.083732  Free energy: -754.152121  ZPE Correction: -754.101705  C 1.18961900 0.43374700 -0.71452300  C 2.16641400 -0.48172500 -0.36218600  C 1.87200000 -1.67773600 0.36372200  C -0.19512600 0.33719000 -0.47550600  C 1.67396900 -2.71670700 0.98780300  H 1.44582600 -3.60987800 1.52234100  C -1.43087200 0.28358800 -0.29680300  C 3.62274500 -0.23228300 -0.69802400  H 4.05911100 -1.09373200 -1.22000600  H 3.70556100 0.64186400 -1.35615300  C 4.47988900 0.01152000 0.63301100  H 4.00910800 0.81709900 1.20606600  H 4.39267600 -0.92055100 1.21036300  B 5.96130300 0.32093700 0.28584500  H 1.53278300 1.32426200 -1.25139400  Au -3.34670500 0.14642400 -0.00713300  C 6.58413700 1.71736300 0.47058400  C 6.88394700 -0.75239200 -0.32966800  N 7.05363000 2.79362000 0.63456700  N 7.58555600 -1.58786900 -0.79275600  F -5.34628300 -0.03410000 0.29554700 |
|   B3LYP/LANL2DZ | Total Energy: -669.3599853  Enthalpy: -669.211349  Free energy: -669.281951  ZPE Correction: -669.229797  C 1.47537200 0.38294500 -0.73295700  C 2.45817300 -0.51408000 -0.35805100  C 2.17354300 -1.70085500 0.38737800  C 0.08842800 0.28036200 -0.49723100  C 1.98390400 -2.73126200 1.02761600  H 1.76389900 -3.61812400 1.57601200  C -1.14735000 0.23780300 -0.32462600  C 3.91439600 -0.25397200 -0.69120900  H 4.36111900 -1.11847400 -1.19946200  H 3.98943400 0.61049900 -1.36296100  C 4.76096500 0.01974200 0.63704700  H 4.27841700 0.82605800 1.19923700  H 4.68578900 -0.90514500 1.22834900  B 6.24133000 0.34237500 0.29591100  H 1.81074800 1.26660500 -1.28541000  Au -3.08405600 0.14687000 -0.04525100  C 6.85515600 1.74061600 0.49665600  C 7.17378000 -0.72001900 -0.32401100  N 7.31679000 2.81808800 0.67290400  N 7.88329000 -1.54623900 -0.79109500  Cl -5.48673100 0.01607800 0.29665700 |
|   B3LYP/LANL2DZ | Total Energy: -667.5819713  Enthalpy: -667.433445  Free energy: -667.505319  ZPE Correction: -667.452123  C 2.00470000 0.33477900 -0.74912900  C 2.99393600 -0.54667700 -0.35658600  C 2.71791200 -1.72855800 0.39992900  C 0.61649400 0.22382800 -0.52168900  C 2.53662400 -2.75438700 1.04978300  H 2.32415800 -3.63809500 1.60619900  C -0.62065600 0.18574500 -0.35897700  C 4.44971200 -0.27233000 -0.68158900  H 4.91110400 -1.13681900 -1.17666800  H 4.51846700 0.58499800 -1.36311700  C 5.28183900 0.02808500 0.64886200  H 4.78258400 0.83139400 1.20072600  H 5.21877700 -0.89194100 1.24938400  B 6.75944000 0.37211900 0.31601300  H 2.33479900 1.21521200 -1.30978200  Au -2.56782000 0.12760100 -0.09657800  Br -5.11195200 0.05896400 0.23835100  C 7.35130000 1.77945800 0.51878100  C 7.71176100 -0.67726000 -0.29574200  N 7.79562900 2.86389000 0.69654200  N 8.43684200 -1.49327100 -0.75672300 |
|   B3LYP/LANL2DZ | Total Energy: -730.6909594  Enthalpy: -730.517351  Free energy: -730.589442  ZPE Correction: -730.537061  C 1.17754500 -0.37297400 -0.90150800  C 2.18317800 -0.59320100 0.00493600  C 1.93609000 -0.73689400 1.40975000  C -0.21085300 -0.25213100 -0.61485700  C 1.78892200 -0.86992900 2.62019300  H 1.62644200 -0.98921000 3.66723100  C -1.42717500 -0.13021100 -0.41962300  C 3.63867300 -0.69083800 -0.43980400  H 4.04737200 -1.67350900 -0.16431700  H 3.68931500 -0.61695900 -1.53368100  C 4.52093100 0.41805800 0.20053900  H 4.11919600 1.41710700 -0.02159000  H 4.43452500 0.33267000 1.30478000  B 6.05209400 0.36343800 -0.08750500  H 1.46049600 -0.27852400 -1.95215100  Au -3.32133100 0.08891700 -0.09878900  C 6.92912900 1.62249200 0.03676000  C 6.77437300 -0.94262200 -0.46633300  N 7.58315500 2.60442700 0.13616700  N 7.31399600 -1.95454400 -0.76051700  O -5.43601900 0.35869000 0.25612100  H -6.06507200 0.58724200 -0.44891000  H -5.84439400 0.25037500 1.13169300 |
|   B3LYP/LANL2DZ | Total Energy: -665.5559303  Enthalpy: -665.389261  Free energy: -665.463137  ZPE Correction: -665.409187  C 1.46564900 -0.47895100 -0.86596500  C 2.46490800 -0.54926600 0.07048900  C 2.20691300 -0.46170700 1.47810600  C 0.07526000 -0.31711300 -0.60619200  C 2.04813900 -0.39472700 2.69234200  H 1.87598300 -0.33897700 3.74324000  C -1.14228500 -0.17634300 -0.43027000  C 3.92254100 -0.72651100 -0.34141200  H 4.31708900 -1.65902900 0.08699700  H 3.98208700 -0.82460000 -1.43287700  C 4.81297200 0.45807100 0.12664200  H 4.43227200 1.41412700 -0.26113600  H 4.70940100 0.55616000 1.22830400  B 6.34751500 0.33689200 -0.12233400  H 1.75305300 -0.55607200 -1.91660500  Au -3.05636500 0.04850600 -0.13212900  C 7.24668800 1.58568500 -0.15627700  C 7.04926100 -1.02144800 -0.30379400  N 7.91877800 2.56004600 -0.18184000  N 7.57194600 -2.07427300 -0.44541200  S -5.54831200 0.36123900 0.26143300  H -6.04272700 0.06194200 -0.98861800  H -5.87426300 -0.85638100 0.81637500 |
|   B3LYP/LANL2DZ | Total Energy: -780.5186037  Enthalpy: -780.248164  Free energy: -780.333254  ZPE Correction: -780.273089  C 2.23001800 -0.40821800 -0.92571600  C 3.22099800 -0.56588100 0.01034000  C 2.94957900 -0.63201600 1.41626900  C 0.83495700 -0.29292200 -0.67338300  C 2.78325300 -0.69616100 2.62999200  H 2.60202800 -0.75601900 3.67897300  C -0.39042300 -0.18784800 -0.50580000  C 4.68500400 -0.67787200 -0.40158500  H 5.09251200 -1.64373700 -0.07013500  H 4.75571400 -0.65784300 -1.49666500  C 5.55226800 0.46536400 0.19947300  H 5.15459400 1.45034200 -0.08287400  H 5.44064800 0.43349900 1.30444900  B 7.08919800 0.39430500 -0.04965600  H 2.53452800 -0.36893700 -1.97418600  Au -2.33895800 -0.01888500 -0.21028500  C 7.96196600 1.66198900 -0.01499300  C 7.82250600 -0.93704400 -0.29830400  N 8.61252900 2.65082200 0.01405200  N 8.37189500 -1.96790900 -0.49119000  P -4.73256200 0.18959400 0.15993300  C -5.24249100 1.67957400 1.18961000  H -4.90960700 2.59564400 0.69226100  H -6.33069200 1.70862900 1.31780200  H -4.76082100 1.62512100 2.17056700  C -5.51940500 -1.26321600 1.06029200  H -6.59549900 -1.10162500 1.19223700  H -5.35618700 -2.17884700 0.48390100  H -5.04609000 -1.38299600 2.03952300  C -5.76591400 0.35570200 -1.40326300  H -6.83041500 0.44244900 -1.15691800  H -5.44629700 1.24365600 -1.95701700  H -5.60918400 -0.52192300 -2.03768800 |
|   B3LYP/LANL2DZ | Total Energy: -888.2445022  Enthalpy: -888.048358  Free energy: -888.132731  ZPE Correction: -888.072363  C 2.21378400 -0.56372300 -0.85730400  C 3.20266000 -0.53702500 0.09219300  C 2.92405700 -0.33659000 1.48421200  C 0.81859600 -0.40754400 -0.62106800  C 2.74278800 -0.17184200 2.68571400  H 2.55126100 -0.03523900 3.72592700  C -0.40313600 -0.28258500 -0.45877300  C 4.66750900 -0.72610700 -0.28685300  H 5.06613200 -1.61698200 0.21925700  H 4.74286700 -0.90993800 -1.36612800  C 5.53758100 0.50235000 0.09723400  H 5.15223800 1.42008000 -0.37053500  H 5.41849700 0.68729000 1.18596200  B 7.07706400 0.38137600 -0.12246900  H 2.51217500 -0.72422700 -1.89510600  Au -2.33334500 -0.09303200 -0.17700800  C 7.96197500 1.63605700 -0.22950700  C 7.79524400 -0.97776200 -0.20132300  N 8.62255300 2.61502100 -0.31299500  N 8.33029600 -2.03205800 -0.26355000  P -4.71687900 0.22004300 0.12101000  O -5.36425800 1.77253000 -0.26072300  H -4.83495700 2.57365200 -0.06984000  O -5.25996900 -0.11940300 1.68737200  H -6.22411800 -0.20148000 1.85561800  O -5.84516000 -0.63263800 -0.82903800  H -6.36314400 -0.10717400 -1.47657100 |
|   B3LYP/LANL2DZ | Total Energy: -828.6452245  Enthalpy: -828.408411  Free energy: -828.491679  ZPE Correction: -828.432524  C 2.22017700 -0.29981000 -0.96271900  C 3.20978500 -0.55409200 -0.04720400  C 2.93582900 -0.76475700 1.34405200  C 0.82498500 -0.21101100 -0.69842800  C 2.76522500 -0.95417700 2.54386800  H 2.58142300 -1.12156500 3.58075000  C -0.39904300 -0.12378400 -0.51674100  C 4.67412100 -0.62574000 -0.46678900  H 5.07905900 -1.62064200 -0.23247800  H 4.74648400 -0.49734400 -1.55438600  C 5.54208700 0.45014400 0.24570200  H 5.14796600 1.45944100 0.06029800  H 5.42805700 0.31229300 1.34220400  B 7.07986100 0.40116400 -0.00540400  H 2.52392100 -0.15270600 -2.00154600  Au -2.34417100 0.00468500 -0.20703900  C 7.95702200 1.65247000 0.18015200  C 7.80834700 -0.89522700 -0.40562500  N 8.61147600 2.62823800 0.32640700  N 8.35303200 -1.89946200 -0.71633000  P -4.73905100 0.15837300 0.17266800  N -5.49976600 1.40656500 1.12794900  H -5.56893100 2.33892400 0.73597500  H -5.41799600 1.35778300 2.13724600  N -5.32087400 -1.20605900 1.05707700  H -6.27767700 -1.26873400 1.38668000  H -4.75511900 -2.04309600 1.11680000  N -5.59003200 0.43678900 -1.30392900  H -5.13293100 0.25398300 -2.18812700  H -6.58634900 0.62353100 -1.32616500 |
|  |  |
| H-bond-stabilized TS    B3LYP/LANL2DZ | Total Energy: -680.5211178  Enthalpy: -680.336822  Free energy: -680.396392  ZPE Correction: -680.352105  Frequency: -421.4114  C 1.46164100 2.01648800 -0.01846000  C 2.40512900 1.00559100 -0.35963200  C 1.88359700 -0.29901300 -0.58196600  C 0.11388400 1.66546700 0.07411000  C 0.64025700 -0.69302200 -0.52049000  H 0.02899500 -1.57173300 -0.63533800  C -0.65859700 0.63570600 -0.05515400  C 3.88006700 1.37369000 -0.48806000  H 4.22883100 1.11825100 -1.50011800  H 3.96377800 2.46655900 -0.39030000  C 4.83697700 0.70312900 0.55336200  H 5.70449500 1.37334000 0.69056100  H 4.32389600 0.67564600 1.52839900  B 5.39226600 -0.75899400 0.21159600  H 1.82341400 3.02911700 0.14928900  N 4.58020600 -1.72018100 -0.44280700  H 4.88268900 -2.65845200 -0.67558800  H 3.59748600 -1.50775300 -0.64780900  N 6.74830200 -1.06649700 0.59525700  H 7.34022400 -0.37919100 1.04196500  H 7.17379900 -1.97717600 0.47290400  Au -2.49542500 -0.10407400 0.05561900  F -4.39640800 -0.83258700 0.20274800 |
| H-bond-stabilized TS    B3LYP/LANL2DZ | Total Energy: -595.6498505  Enthalpy: -595.465779  Free energy: -595.526954  ZPE Correction: -595.481476  Frequency: -431.0162  C 1.75462800 2.01779900 -0.00727500  C 2.67705800 0.98813700 -0.34449300  C 2.13142000 -0.30817800 -0.56115000  C 0.39902700 1.69321800 0.08617400  C 0.88743700 -0.69148400 -0.50294800  H 0.25628900 -1.55590200 -0.61621100  C -0.39167700 0.68059700 -0.04403300  C 4.15822300 1.32829100 -0.47770000  H 4.49824900 1.06479600 -1.49059700  H 4.26177000 2.41949400 -0.38246200  C 5.10617200 0.64210800 0.56162000  H 5.98226300 1.30053600 0.69991900  H 4.59388600 0.61874700 1.53709500  B 5.64201100 -0.82568700 0.21376900  H 2.13639200 3.02367300 0.15670400  N 4.81306900 -1.77687700 -0.43462900  H 5.10323500 -2.71778000 -0.67219600  H 3.83253400 -1.55316000 -0.63361100  N 6.99754200 -1.14857500 0.58435100  H 7.60127400 -0.46828800 1.02597000  H 7.41257300 -2.06323700 0.45592200  Au -2.26686800 -0.00296200 0.03731600  Cl -4.57729000 -0.78418900 0.15255600 |
| H-bond-stabilized TS    B3LYP/LANL2DZ | Total Energy: -593.8722764  Enthalpy: -593.688295  Free energy: -593.750883  ZPE Correction: -593.704231  Frequency: -433.7056  C 2.27632100 2.03317300 0.01784100  C 3.16718000 0.98060100 -0.33155900  C 2.58265300 -0.29529400 -0.56848600  C 0.91046300 1.74943000 0.10291900  C 1.32932800 -0.64514500 -0.52064900  H 0.67131700 -1.48716700 -0.64813800  C 0.08990200 0.76415100 -0.04459700  C 4.65873600 1.27563100 -0.45621800  H 4.99362000 1.01231300 -1.47085000  H 4.79608100 2.36191600 -0.34878800  C 5.58121300 0.54886900 0.57838800  H 6.47744000 1.17764400 0.72532500  H 5.06615700 0.53228800 1.55256000  B 6.07153800 -0.93165000 0.21769800  H 2.68850200 3.02427200 0.19743900  N 5.21689800 -1.84854900 -0.44662600  H 5.47837100 -2.79573700 -0.69240700  H 4.24536200 -1.59180700 -0.64897500  N 7.41323800 -1.30223100 0.59356800  H 8.03580300 -0.64663300 1.04625400  H 7.79948100 -2.22838500 0.45781400  Au -1.81168700 0.13397400 0.01670700  Br -4.27063600 -0.61992700 0.10648500 |
| H-bond-stabilized TS    B3LYP/LANL2DZ  TS Without H-bond stabilization   | Total Energy: -656.9833761  Enthalpy: -656.774093  Free energy: -656.837899  ZPE Correction: -656.791189  Frequency: -466.2357  C -1.48011400 2.00076300 0.00875800  C -2.41746600 0.99654900 0.38441900  C -1.88449800 -0.29309400 0.62931600  C -0.12750300 1.66259900 -0.09432800  C -0.67557800 -0.75103100 0.58711300  H -0.08130300 -1.63717300 0.72290200  C 0.60501200 0.61077000 0.06741100  C -3.89207000 1.35877900 0.51045600  H -4.24373200 1.08456100 1.51509500  H -3.97645300 2.45128100 0.43006900  C -4.83050700 0.69847200 -0.55315100  H -5.68458100 1.38003800 -0.70214000  H -4.30382100 0.67135200 -1.52061900  B -5.41913600 -0.75547000 -0.22944100  H -1.85504000 3.00460300 -0.17594500  N -4.65913100 -1.72342200 0.48345700  H -5.00158300 -2.64801600 0.71500600  H -3.69103600 -1.54491200 0.74277000  N -6.74753400 -1.05241800 -0.68558900  H -7.31269600 -0.36321100 -1.16320700  H -7.19163300 -1.95589200 -0.57646200  Au 2.43858200 -0.08207500 -0.05480600  O 4.48341000 -0.81886800 -0.22255500  H 4.86329000 -1.12156600 -1.06491600  H 5.12204300 -0.78080500 0.50975100  Total Energy: -656.9802875  Enthalpy: -656.771125  Free energy: -656.836360  ZPE Correction: -656.788476  Frequency: -477.8843  C 1.50538300 2.07745000 0.23376500  C 2.46838300 1.12345700 -0.20577700  C 1.96722800 -0.14724200 -0.56261200  C 0.15305900 1.71696700 0.28602200  C 0.77746400 -0.64733900 -0.58941100  H 0.21708600 -1.53684200 -0.81531800  C -0.52524400 0.64673400 0.01177400  C 3.93952600 1.50310100 -0.27374100  H 4.28409700 1.41175700 -1.31554800  H 4.04446100 2.56235100 -0.00004800  C 4.84843700 0.63241400 0.64512100  H 5.84054800 1.11424300 0.68775500  H 4.44839900 0.67026200 1.66967700  B 5.07126200 -0.88297600 0.18846800  H 1.86367600 3.06674800 0.50995000  N 4.90342000 -1.93456700 1.14095900  H 5.04756300 -2.91523500 0.93448000  H 4.54007200 -1.75045600 2.06627500  N 5.51259900 -1.16320300 -1.14242400  H 5.61533500 -0.43335500 -1.83424200  H 5.65832400 -2.09801200 -1.50199200  Au -2.35013500 -0.08360600 0.01311800  O -4.39437200 -0.85782900 0.03095100  H -4.99904000 -0.75804500 -0.72409100  H -4.81874400 -1.19986800 0.83625700 |
| H-bond-stabilized TS    B3LYP/LANL2DZ  TS Without H-bond Stabilization    B3LYP/LANL2DZ | Total Energy: -591.8489387  Enthalpy: -591.646474  Free energy: -591.712012  ZPE Correction: -591.663745  Frequency: -466.6687  C -1.77573400 2.00213700 -0.00506300  C -2.69202200 0.98124100 0.37542300  C -2.13434200 -0.29722000 0.62730100  C -0.41674600 1.68784400 -0.10461000  C -0.92052000 -0.73855400 0.59124900  H -0.30736700 -1.61048800 0.73301200  C 0.33779300 0.65457700 0.06453500  C -4.17349500 1.31455700 0.49957300  H -4.52009600 1.03515300 1.50448700  H -4.27916200 2.40492800 0.41713600  C -5.09784300 0.63379800 -0.56347300  H -5.96306700 1.30003600 -0.71662200  H -4.56925600 0.61210700 -1.52999700  B -5.66247400 -0.82838900 -0.23436100  H -2.17000300 2.99727700 -0.19609800  N -4.88777400 -1.77963900 0.48561100  H -5.21558700 -2.70835000 0.72176900  H -3.92390100 -1.58380200 0.74695800  N -6.98421900 -1.14995100 -0.69199100  H -7.55995100 -0.47273900 -1.17415400  H -7.41370900 -2.05998300 -0.57892000  Au 2.21338000 0.02462000 -0.03958600  S 4.64926700 -0.71412500 -0.20054300  H 4.51510100 -1.88866000 -0.90695400  H 4.81544200 -1.32567000 1.02223300  Total Energy: -591.846054  Enthalpy: -591.643703  Free energy: -591.710374  ZPE Correction: -591.661188  Frequency: -477.9790  C -1.80252500 2.08009200 -0.22566900  C -2.74173900 1.10106200 0.20819800  C -2.21183800 -0.16051300 0.55821200  C -0.44234900 1.74888200 -0.27839500  C -1.01506800 -0.63991100 0.58603400  H -0.43293300 -1.51613000 0.80762200  C 0.26166700 0.69566800 -0.00980500  C -4.22127200 1.44595500 0.27812300  H -4.56236900 1.34379400 1.32002100  H -4.35063500 2.50323800 0.00781700  C -5.11015200 0.55716600 -0.64266100  H -6.11140300 1.01940300 -0.68740300  H -4.70904000 0.60319000 -1.66642200  B -5.30598700 -0.96244200 -0.18745400  H -2.18332700 3.06222300 -0.49705100  N -5.13397400 -2.00833600 -1.14542300  H -5.26635100 -2.99133500 -0.94220900  H -4.78796500 -1.81588000 -2.07568100  N -5.72800400 -1.25296200 1.14729300  H -5.83491700 -0.52694800 1.84249000  H -5.86080500 -2.19066300 1.50430000  Au 2.12971000 0.03103000 -0.01637700  S 4.56277900 -0.75141200 -0.04899600  H 4.43165900 -1.97145300 -0.67432200  H 4.67997400 -1.27731900 1.21851000 |
| H-bond-stabilized TS    B3LYP/LANL2DZ  TS without H-bond stabilization    B3LYP/LANL2DZ | Total Energy: -706.8115689  Enthalpy: -706.505287  Free energy: -706.582111  ZPE Correction: -706.527537  Frequency: -456.4523  C 2.52106200 -2.02836100 -0.03808400  C 3.40102800 -0.97651200 0.34504700  C 2.80003500 0.27953400 0.61688000  C 1.15159800 -1.75807100 -0.11853700  C 1.56357800 0.66243100 0.59257700  H 0.91821100 1.50822700 0.75054100  C 0.35231400 -0.76216100 0.06652700  C 4.89541100 -1.25528600 0.45323200  H 5.23747000 -0.98337100 1.46198700  H 5.04265400 -2.33910700 0.34792700  C 5.78839800 -0.51843000 -0.59939200  H 6.67775200 -1.14772100 -0.77123900  H 5.25350300 -0.49584700 -1.56247300  B 6.29715500 0.95708700 -0.24063200  H 2.94964300 -3.00597400 -0.24537500  N 5.47971900 1.86703200 0.48433300  H 5.76843200 2.80367000 0.73941300  H 4.52146900 1.62620400 0.73287100  N 7.61256600 1.33322600 -0.67725800  H 8.21734100 0.68486000 -1.16346100  H 8.00819400 2.25569500 -0.54323500  Au -1.57126300 -0.18207300 0.00067300  P -3.91778500 0.44824000 -0.09897000  C -4.29548300 1.96934200 -1.14022800  H -3.96388100 1.79694900 -2.16860800  H -5.37009800 2.18585200 -1.13414400  H -3.74845900 2.82869400 -0.74080800  C -4.70950100 0.83949700 1.56258400  H -5.76688900 1.09891200 1.43598400  H -4.62319400 -0.03138600 2.21929800  H -4.18207900 1.67704000 2.02915600  C -5.03838300 -0.87758700 -0.82379600  H -4.97343400 -1.78233700 -0.21178200  H -6.07811300 -0.53214600 -0.85527000  H -4.70410900 -1.12164600 -1.83666200  Total Energy: -706.8079852  Enthalpy: -706.501824  Free energy: -706.580326  ZPE Correction: -706.524331  Frequency: -469.3151  C 2.54407600 -2.09348100 -0.23779200  C 3.44409100 -1.07693300 0.19542600  C 2.86751300 0.16300200 0.55268900  C 1.17315400 -1.81098800 -0.28073800  C 1.64536000 0.58143000 0.58156500  H 1.02849600 1.43230700 0.80936500  C 0.42175900 -0.79170900 -0.00839600  C 4.93680000 -1.36304100 0.25634400  H 5.27922600 -1.25617500 1.29757000  H 5.10710400 -2.41230500 -0.02330700  C 5.78661100 -0.43293500 -0.66064500  H 6.80347200 -0.85857400 -0.71973900  H 5.37786500 -0.48044700 -1.68137600  B 5.93300700 1.08684800 -0.18858600  H 2.96287500 -3.05840300 -0.51547300  N 5.71838400 2.13804500 -1.13260300  H 5.81132500 3.12267500 -0.91591300  H 5.36284400 1.94465300 -2.05905400  N 6.35900300 1.37629400 1.14542300  H 6.48617200 0.64610500 1.83281300  H 6.45671600 2.31377600 1.51407600  Au -1.49805700 -0.19158100 0.00027900  P -3.84577900 0.45048400 -0.01866700  C -4.84295900 -0.32213900 -1.41466500  H -5.89134500 -0.00603100 -1.36574200  H -4.41474500 -0.02074800 -2.37546100  H -4.78680500 -1.41235000 -1.33962100  C -4.17927000 2.29367100 -0.20201600  H -5.25690200 2.49437600 -0.19970100  H -3.70724300 2.83276500 0.62499500  H -3.74406700 2.65019800 -1.14051400  C -4.80960300 -0.01991900 1.52781000  H -5.85762600 0.28987700 1.44165500  H -4.75958300 -1.10365700 1.67027100  H -4.35649900 0.46322300 2.39879900 |
| H-bond-stabilized TS    B3LYP/LANL2DZ  TS without H-Bond stabilization    B3LYP/LANL2DZ | Total Energy: -814.5379071  Enthalpy: -814.305856  Free energy: -814.381434  ZPE Correction: -814.327164  Frequency: -468.7799  C 2.48512500 -2.03578700 -0.05118900  C 3.37810500 -0.98827200 0.31674300  C 2.78574600 0.26239000 0.61929100  C 1.11444500 -1.76529800 -0.08950200  C 1.55855900 0.66510400 0.64266700  H 0.92680100 1.51610600 0.82731600  C 0.33693800 -0.75923600 0.13468700  C 4.87458900 -1.26691500 0.37279600  H 5.24473300 -1.02682200 1.37948700  H 5.02096600 -2.34570000 0.22717700  C 5.72880800 -0.48817900 -0.68314800  H 6.59464200 -1.12209800 -0.93534100  H 5.14694000 -0.40124300 -1.61458500  B 6.28082200 0.95620700 -0.26530400  H 2.90747000 -3.01116500 -0.27922300  N 5.48913800 1.85764700 0.49912500  H 5.80400000 2.77475400 0.79138100  H 4.52985300 1.63152100 0.75140300  N 7.60375500 1.31429800 -0.68945100  H 8.18921300 0.67230400 -1.20673500  H 8.02499900 2.21890800 -0.51697400  Au -1.56480900 -0.17422800 0.06370700  P -3.89853000 0.46410000 -0.15050200  O -4.84773500 -0.42039000 -1.28539900  H -4.41765900 -0.74097200 -2.10450400  O -4.10912300 2.09468300 -0.54211900  H -5.00309000 2.49217300 -0.45443000  O -5.02523200 0.30941000 1.12211500  H -5.64253400 -0.45234600 1.08770600  Total Energy: -814.535301  Enthalpy: -814.303273  Free energy: -814.379596  ZPE Correction: -814.324747  Frequency: -478.5177  C 2.51766100 -2.09220600 -0.22601500  C 3.42470700 -1.07933100 0.20209600  C 2.85419700 0.16329600 0.55271100  C 1.14821200 -1.80331200 -0.27042600  C 1.64471200 0.60788800 0.58825500  H 1.04011700 1.46788300 0.81534400  C 0.41653000 -0.76889600 0.00046400  C 4.91563900 -1.37175500 0.26276300  H 5.25829300 -1.25796500 1.30280600  H 5.08110100 -2.42331600 -0.00967600  C 5.76570900 -0.44920200 -0.66155400  H 6.78372100 -0.87253900 -0.70961500  H 5.36312000 -0.51073900 -1.68388000  B 5.90225700 1.07729700 -0.20622300  H 2.93118300 -3.06029200 -0.49930600  N 5.68068600 2.11524600 -1.16208100  H 5.77083100 3.10289300 -0.95812200  H 5.33850700 1.90954800 -2.09094300  N 6.31745400 1.38332800 1.12704400  H 6.46039800 0.66188000 1.82056900  H 6.41215900 2.32546700 1.48458700  Au -1.48163600 -0.16368000 -0.00031100  P -3.83701100 0.44427300 -0.01149500  O -4.64094100 1.03800000 -1.39588800  H -4.94017400 0.39751500 -2.07371300  O -4.92982000 -0.85532100 0.28329400  H -4.64197800 -1.57550700 0.88202500  O -4.26979700 1.67824900 1.05738800  H -5.15089800 2.09898300 0.94519400 |
| H-bond-stabilized TS    B3LYP/LANL2DZ  TS without H-Bond stabilization    B3LYP/LANL2DZ | Total Energy: -754.9382084  Enthalpy: -754.665568  Free energy: -754.740421  ZPE Correction: -754.687017  Frequency: -459.4335  C 2.51608600 -2.01976800 -0.03874300  C 3.38572500 -0.96949200 0.37233000  C 2.77621000 0.28184900 0.64339500  C 1.14784600 -1.75228800 -0.14235400  C 1.54169700 0.66717200 0.59745200  H 0.89381800 1.51132500 0.75446900  C 0.34685400 -0.75595100 0.03326800  C 4.87820900 -1.24577200 0.50900700  H 5.20461000 -0.95053000 1.51614800  H 5.02702200 -2.33156400 0.43039700  C 5.78726000 -0.53194600 -0.54521300  H 6.68630700 -1.15712800 -0.67695200  H 5.27541900 -0.54417100 -1.52094700  B 6.27629800 0.95828900 -0.22099800  H 2.95160000 -2.99432000 -0.24585400  N 5.45518600 1.86971500 0.49816000  H 5.73459400 2.81478900 0.73170700  H 4.50171900 1.62459000 0.75896400  N 7.57982400 1.34603700 -0.68167700  H 8.18951300 0.69782000 -1.16193400  H 7.96215500 2.27717800 -0.57102800  Au -1.57621700 -0.18845700 -0.02636000  P -3.92136600 0.45056200 -0.08654000  N -4.17155400 1.83253500 -1.10329800  H -3.45058600 2.12413100 -1.75721700  H -5.03672000 2.36607000 -1.06964000  N -4.83675300 1.01588600 1.29828300  H -4.70440000 1.95941000 1.64467500  H -5.12624000 0.32400800 1.98040300  N -4.89551500 -0.90098600 -0.49776000  H -4.44506000 -1.71529700 -0.89003600  H -5.90125800 -0.85322200 -0.60844700  Total Energy: -754.9348735  Enthalpy: -754.662364  Free energy: -754.738012  ZPE Correction: -754.684019  Frequency: -471.4517  C 2.53318100 -2.09947500 -0.17253300  C 3.43230900 -1.06567600 0.22016300  C 2.85405700 0.18612600 0.52851400  C 1.16197000 -1.82077500 -0.22552200  C 1.63324100 0.60738600 0.54294800  H 1.01744600 1.46751200 0.73653800  C 0.41209300 -0.79076500 0.00720700  C 4.92524100 -1.34760200 0.29204300  H 5.26523400 -1.20838300 1.33025600  H 5.09713600 -2.40477500 0.04553100  C 5.77510800 -0.44487100 -0.65136300  H 6.79246400 -0.87100400 -0.69566200  H 5.36849800 -0.52394400 -1.67096500  B 5.91995900 1.08906300 -0.22645600  H 2.95312600 -3.07374400 -0.41297100  N 5.72465000 2.10870700 -1.20836500  H 5.82097500 3.09961200 -1.02419800  H 5.38660400 1.88579500 -2.13475200  N 6.32516400 1.42090400 1.10397500  H 6.43882400 0.71368200 1.81726400  H 6.42321300 2.36974600 1.44221700  Au -1.50473500 -0.19626300 -0.00009200  P -3.84976700 0.45374300 -0.01464200  N -4.57327900 1.53969500 -1.17674600  H -4.72297400 1.20204300 -2.12093600  H -4.38623400 2.53225100 -1.08933700  N -4.85911800 -0.91609900 -0.30996200  H -5.86022200 -0.83151400 -0.44598600  H -4.48402700 -1.84908400 -0.19470000  N -4.27323500 1.32879500 1.41416200  H -5.18838800 1.74737900 1.53893000  H -3.67335300 1.27763600 2.22774600 |
|  |  |
| H-bond-stabilized TS    B3LYP/LANL2DZ | Free Energy: -720.191462  Enthalpy: -720.133902  ZPE Corrected Energy: -720.148349  Total Energy: -720.2915654  Frequency: -374.2429  C 1.49562200 2.07823400 -0.01595000  C 2.47359700 1.08303700 -0.24298000  C 2.00075600 -0.26166600 -0.39626300  C 0.15950800 1.67291000 0.03443600  C 0.75484800 -0.65738200 -0.35761300  H 0.13664800 -1.53587200 -0.42990000  C -0.65340400 0.68202900 -0.04090100  C 3.94587400 1.46254200 -0.35258800  H 4.27371200 1.32154000 -1.39544000  H 4.04431200 2.53650600 -0.13673200  C 4.91101700 0.66744100 0.58017500  H 5.87457100 1.19595700 0.63458000  H 4.49806400 0.67359100 1.60224400  B 5.21251500 -0.83110400 0.16003800  H 1.80111100 3.11499500 0.10349100  O 4.30165100 -1.69820600 -0.37982000  H 3.33680300 -1.34236700 -0.47634300  O 6.52093600 -1.30103200 0.36143800  H 6.64890500 -2.23757800 0.11203400  Au -2.46486600 -0.10151800 0.03543000  F -4.33998700 -0.88003200 0.14267900 |
| H-bond-stabilized TS  B3LYP/LANL2DZ | Free Energy: -635.321295  Enthalpy: -635.262034  ZPE Corrected Energy: -635.276910  Total Energy: -635.4195298  Frequency: -385.6767  C 1.79130600 2.07777000 -0.00580600  C 2.74525100 1.06086400 -0.23534400  C 2.24375100 -0.27395800 -0.38432200  C 0.44560000 1.70306400 0.04979200  C 0.99682900 -0.65716700 -0.34426600  H 0.35792300 -1.52056300 -0.41470500  C -0.38501100 0.72954700 -0.02728400  C 4.22425400 1.41043300 -0.35345800  H 4.54374800 1.25782600 -1.39717900  H 4.34381600 2.48328800 -0.14390400  C 5.17842200 0.60238500 0.57920900  H 6.15012500 1.11587700 0.62998700  H 4.76821000 0.61772200 1.60220800  B 5.45599300 -0.90103100 0.16156600  H 2.12212200 3.10716400 0.11051100  O 4.52949000 -1.75624100 -0.37272400  H 3.57321500 -1.39030100 -0.46762700  O 6.75692100 -1.39053500 0.35719500  H 6.87175100 -2.32912600 0.10937500  Au -2.23869400 0.00586400 0.02426200  Cl -4.52280800 -0.82608400 0.09999600 |
| H-bond-stabilized TS  B3LYP/LANL2DZ | Free Energy: -633.544961  Enthalpy: -633.484350  ZPE Corrected Energy: -633.499462  Total Energy: -633.6417692  Frequency: -388.6424  C 2.31412100 2.08984900 0.00755800  C 3.23406900 1.04373600 -0.22799200  C 2.68946200 -0.27303500 -0.38650400  C 0.95619500 1.75966900 0.05957000  C 1.43285100 -0.62019300 -0.35119600  H 0.76516200 -1.46082400 -0.42846000  C 0.09490100 0.81458500 -0.02568700  C 4.72399500 1.34489700 -0.34173000  H 5.04027100 1.18718800 -1.38565700  H 4.87839200 2.41206200 -0.12631200  C 5.64889100 0.50103800 0.58867300  H 6.63646800 0.98266900 0.64498400  H 5.23670700 0.52384300 1.61073200  B 5.87954700 -1.00816300 0.16359400  H 2.67913200 3.10679100 0.13128000  O 4.92826400 -1.83044200 -0.37939400  H 3.98527600 -1.43480500 -0.47540800  O 7.16281300 -1.54081400 0.36154600  H 7.24826000 -2.48138400 0.10943100  Au -1.78853900 0.14968900 0.01221900  Br -4.22480900 -0.64609600 0.06693600 |
| H-bond-stabilized TS  B3LYP/LANL2DZ | Free Energy: -696.623993  Enthalpy: -696.562174  ZPE Corrected Energy: -696.578442  Total Energy: -696.7451894  Frequency: -442.0654  C 1.50455000 2.07609100 0.00587800  C 2.47096900 1.07430600 -0.26544400  C 1.98188700 -0.25046300 -0.45268500  C 0.16070100 1.69771200 0.06443900  C 0.76867100 -0.70693200 -0.43004900  H 0.18035900 -1.60198800 -0.53247700  C -0.58743800 0.65919300 -0.05117200  C 3.94409900 1.44653600 -0.37389800  H 4.27123700 1.29069900 -1.41350700  H 4.04410800 2.52110700 -0.17092000  C 4.89788600 0.66032700 0.57688200  H 5.85220600 1.20166400 0.64214200  H 4.47590800 0.67010400 1.59488500  B 5.23929400 -0.83395600 0.17598600  H 1.83849400 3.10040900 0.14826100  O 4.36320800 -1.72250500 -0.40540100  H 3.41928800 -1.41622300 -0.54729400  O 6.53199100 -1.28260800 0.43623100  H 6.71606700 -2.21374700 0.20560300  Au -2.40311600 -0.07759600 0.03590500  O -4.41619900 -0.87997000 0.15946300  H -4.81195100 -1.18099600 0.99513700  H -5.01597500 -0.93117000 -0.60430400 |
| H-bond-stabilized TS   B3LYP/LANL2DZ  TS without H-Bond stabilization    B3LYP/LANL2DZ | Free Energy: -631.497830  Enthalpy: -631.434194  ZPE Corrected Energy: -631.450665  Total Energy: -631.6103766  Frequency: -443.0284  C 1.80332800 2.07575100 0.01415000  C 2.74641700 1.05345000 -0.26030400  C 2.22897000 -0.26100300 -0.44765900  C 0.45186900 1.72428700 0.07423600  C 1.01070500 -0.69935700 -0.42548600  H 0.40129400 -1.57977600 -0.52872200  C -0.32028300 0.70525300 -0.04302400  C 4.22677900 1.39431200 -0.37200400  H 4.54821400 1.22988600 -1.41204200  H 4.34933000 2.46683600 -0.17121900  C 5.16574200 0.59026800 0.57847500  H 6.13018700 1.11337500 0.64243000  H 4.74523400 0.60895800 1.59691600  B 5.47893400 -0.91027400 0.17820700  H 2.15953700 3.09237200 0.15770000  O 4.58570900 -1.78277100 -0.40235100  H 3.64856300 -1.46019000 -0.54454800  O 6.76273700 -1.38258300 0.43761500  H 6.93101700 -2.31686400 0.20785700  Au -2.18181300 0.03583200 0.02687600  S -4.59222400 -0.76607800 0.13439800  H -4.45702300 -1.87653500 0.93749900  H -4.68094900 -1.47512300 -1.04288600  Free Energy: -631.490330  Enthalpy: -631.424853  ZPE Corrected Energy: -631.441753  Total Energy: -631.6011343  Frequency: -471.6721  C -1.79311900 2.13583800 -0.06891100  C -2.76199600 1.10994200 0.11057400  C -2.26968600 -0.21049300 0.23433800  C -0.43757700 1.78708900 -0.09821800  C -1.07569200 -0.70148500 0.23051600  H -0.50905300 -1.61080300 0.32392400  C 0.25211400 0.69710000 -0.00591400  C -4.24324700 1.44839900 0.16904000  H -4.59469700 1.35787800 1.20685200  H -4.39022100 2.49311900 -0.13529700  C -5.10958600 0.50251200 -0.71076600  H -6.12919100 0.91858200 -0.75121500  H -4.72458400 0.49560600 -1.74073400  B -5.23348700 -0.95482600 -0.11434700  H -2.14582700 3.15872000 -0.17876100  O -4.95257800 -2.12977700 -0.80611600  H -4.47857800 -2.06891500 -1.65326400  O -5.74952300 -1.09338400 1.16273100  H -5.81205500 -1.99643400 1.52571800  Au 2.11100400 0.00825900 -0.00080700  S 4.52069200 -0.83223400 0.00541400  H 5.06240600 -0.02691100 0.98263200  H 5.03840900 -0.11157800 -1.04789000 |
| H-bond-stabilized TS    B3LYP/LANL2DZ | Free Energy: -746.369682  Enthalpy: -746.294378  ZPE Corrected Energy: -746.315824  Total Energy: -746.5743732  Frequency: -429.7370  C 2.55942800 -2.10107100 -0.04171500  C 3.45763100 -1.04146300 0.23910300  C 2.88524000 0.24851900 0.44967000  C 1.19446900 -1.80242300 -0.08441400  C 1.64256900 0.62023400 0.43972800  H 0.99161100 1.46835600 0.55927300  C 0.36608800 -0.83185000 0.04858600  C 4.95310600 -1.31612200 0.33305700  H 5.27641700 -1.15614900 1.37337700  H 5.12341600 -2.37820000 0.11124300  C 5.84660100 -0.45232100 -0.60898300  H 6.83487500 -0.92688400 -0.68880300  H 5.41903400 -0.47291400 -1.62453100  B 6.08792100 1.05470800 -0.18318100  H 2.95709900 -3.09941700 -0.20376600  O 5.15589300 1.87061200 0.41556600  H 4.23454200 1.49351900 0.55368700  O 7.34735400 1.59437900 -0.43946600  H 7.46634200 2.53191900 -0.19240400  Au -1.54488700 -0.21387300 0.00322800  P -3.87142400 0.48158700 -0.07016200  C -4.33511000 1.83348300 1.15264900  H -5.39706300 2.08883500 1.06162400  H -4.12898200 1.49004500 2.17083100  H -3.72930500 2.72373500 0.95850300  C -5.10024100 -0.89558000 0.29041000  H -6.12855500 -0.51991600 0.23941400  H -4.96973200 -1.69976600 -0.44000300  H -4.90967700 -1.30112700 1.28860000  C -4.43332200 1.16321600 -1.73077000  H -4.28277500 0.40426000 -2.50440900  H -5.49220800 1.44385900 -1.69567600  H -3.83192600 2.04080500 -1.98637000 |
| H-bond-stabilized TS    B3LYP/LANL2DZ  TS without H-Bond stabilization    B3LYP/LANL2DZ | Total Energy: -854.2988673  Enthalpy: -854.093129  Free energy: -854.166381  ZPE Correction: -854.113598  Frequency: -447.1133  C -2.53006100 -2.09516100 0.04530800  C -3.43271300 -1.03835400 -0.24122600  C -2.86414700 0.24809000 -0.46454400  C -1.16639300 -1.79547300 0.08059100  C -1.62988900 0.64002900 -0.46830300  H -0.99154300 1.49596900 -0.60296700  C -0.36011100 -0.80750900 -0.07140200  C -4.92714700 -1.31924000 -0.32610900  H -5.25703700 -1.15532400 -1.36345100  H -5.09093800 -2.38278100 -0.10817700  C -5.81632300 -0.46352400 0.62705900  H -6.80035000 -0.94530600 0.71333000  H -5.38130200 -0.48581800 1.63930900  B -6.07474700 1.04331500 0.21099600  H -2.92737700 -3.09213700 0.21509900  O -5.16072300 1.86835900 -0.40518700  H -4.24117100 1.50857400 -0.56391900  O -7.33003500 1.57351900 0.49489600  H -7.46504300 2.51083800 0.25577200  Au 1.53723300 -0.20816800 -0.04168800  P 3.85853100 0.48635700 0.08426300  O 4.06104800 2.14679500 -0.16950600  H 4.96177600 2.50944300 -0.31738300  O 5.04266300 -0.18130900 -0.93980900  H 5.73501800 -0.73408600 -0.51651000  O 4.71507700 0.13184400 1.53692000  H 4.24879400 0.17713500 2.39657400  Total Energy: -854.2897797  Enthalpy: -854.083949  Free energy: -854.159804  ZPE Correction: -854.104935  Frequency: -473.0950  C 2.52127000 -2.14880800 -0.05309900  C 3.44573600 -1.08058900 0.12228800  C 2.89970600 0.21922500 0.23330100  C 1.15488300 -1.85123500 -0.09065800  C 1.68747900 0.66321500 0.22121900  H 1.08812600 1.55248100 0.30626400  C 0.42351600 -0.78777600 -0.01171800  C 4.93937600 -1.35666200 0.18715400  H 5.28245600 -1.24231900 1.22535500  H 5.13101500 -2.39656800 -0.10827200  C 5.76659600 -0.38080100 -0.69666800  H 6.80393000 -0.75151800 -0.72985700  H 5.38726100 -0.39872000 -1.72857900  B 5.82215200 1.08554400 -0.11147700  H 2.91638500 -3.15673100 -0.15446800  O 5.53806500 2.24314300 -0.83032700  H 5.11541900 2.16022200 -1.70239700  O 6.27789500 1.25283600 1.18440300  H 6.30088600 2.16088800 1.53926900  Au -1.47292500 -0.17732200 -0.02679800  P -3.81972100 0.45256200 0.01294200  O -4.77014700 0.56625000 -1.39887500  H -5.38781100 -0.17331700 -1.58324100  O -4.88369800 -0.64192200 0.81528000  H -4.55710300 -1.13507000 1.59574900  O -4.10848100 1.97129100 0.69940900  H -4.99487000 2.38076000 0.59042900 |
| H-bond-stabilized TS    B3LYP/LANL2DZ | Total Energy: -794.7006061  Enthalpy: -794.454248  Free energy: -794.526955  ZPE Correction: -794.474873  Frequency: -433.9453  C 2.54743900 -2.10043900 -0.05015300  C 3.44324000 -1.04121800 0.24177300  C 2.86826500 0.24573100 0.45952600  C 1.18253000 -1.80356300 -0.09590800  C 1.62663600 0.61947800 0.44881400  H 0.97685300 1.46777600 0.57453300  C 0.35672900 -0.83150000 0.04330600  C 4.93855600 -1.31493900 0.33955300  H 5.25776600 -1.15950900 1.38175400  H 5.11058200 -2.37571400 0.11329800  C 5.83418000 -0.44575100 -0.59541300  H 6.82289200 -0.91945700 -0.67422500  H 5.41029600 -0.46187600 -1.61256200  B 6.07411300 1.05933500 -0.16202500  H 2.94760100 -3.09687600 -0.21731000  O 5.14122800 1.87207000 0.44028700  H 4.22035000 1.49602900 0.57611600  O 7.33296900 1.60077400 -0.41473400  H 7.45254600 2.53709800 -0.16348900  Au -1.55133900 -0.21893900 -0.00469700  P -3.87264500 0.49205500 -0.07659500  N -4.73900000 1.14658700 1.28805300  H -4.54418700 2.09914800 1.57467500  H -5.03287100 0.51450400 2.02402600  N -4.08082100 1.82354400 -1.15531800  H -3.37044000 2.02447700 -1.84782200  H -4.96451000 2.31312900 -1.24387200  N -4.91382500 -0.84357100 -0.40828100  H -4.53147100 -1.69547900 -0.79886900  H -5.92399100 -0.76005600 -0.38071500 |
|  |  |
|   B3LYP/LANL2DZ | Free Energy: -569.642575  Enthalpy: -569.589471  ZPE Corrected Energy: -569.602173  Total Energy: -569.7326076  Frequency: -142.4964  C 2.36974500 1.59291100 -0.04428000  C 3.38752200 0.66601600 -0.10108400  C 3.01605800 -0.74655800 -0.12938700  C 1.00418400 1.21366400 -0.03202600  C 1.92528200 -1.38859800 -0.10867600  H 1.11640000 -2.08998100 -0.10495400  C -0.15768300 0.74795300 -0.02284100  C 4.89098900 0.87562900 -0.10938500  H 5.24302900 0.99574600 -1.14756200  H 5.18151200 1.78351200 0.44043700  C 5.48048600 -0.43733600 0.47412400  H 6.54828000 -0.55313400 0.22400200  H 5.39644100 -0.42709900 1.57154700  B 4.62338800 -1.66945600 -0.17586900  H 4.55507800 -2.70683100 0.43878200  H 4.76503700 -1.82186500 -1.37083700  H 2.62840900 2.65215300 0.00533900  Au -1.96762600 0.02752000 0.00958000  F -3.84535300 -0.75570700 0.05634600 |
|   B3LYP/LANL2DZ | Free Energy: -484.771910  Enthalpy: -484.717507  ZPE Corrected Energy: -484.730546  Total Energy: -484.8603028  Frequency: -128.1208  C -2.66279300 -1.57005400 -0.03631600  C -3.65893500 -0.62191000 -0.09719200  C -3.25236700 0.78396800 -0.12378300  C -1.29144200 -1.20732200 -0.02082400  C -2.12452600 1.36471600 -0.09912100  H -1.28755100 2.03275800 -0.09452200  C -0.12121900 -0.76379100 -0.01206500  C -5.16642900 -0.79298800 -0.11366800  H -5.51311600 -0.90416300 -1.15452300  H -5.48322600 -1.69347600 0.43366100  C -5.72424800 0.53604300 0.46505900  H -6.78520700 0.68257400 0.20362000  H -5.65238400 0.52210300 1.56335500  B -4.81948600 1.74130600 -0.17516800  H -4.72721400 2.77570100 0.44178200  H -4.95567000 1.90355400 -1.37026200  H -2.93669500 -2.62486800 0.01164200  Au 1.73323500 -0.11919000 0.00661600  Cl 4.03086100 0.68490000 0.03211300 |
|   B3LYP/LANL2DZ | Free Energy: -482.995354  Enthalpy: -482.939621  ZPE Corrected Energy: -482.952877  Total Energy: -483.0822868  Frequency: -124.4478  C -3.16198400 -1.54799500 -0.03051000  C -4.12301100 -0.56469700 -0.09414200  C -3.66495500 0.82595800 -0.12209200  C -1.77902000 -1.23088700 -0.01400600  C -2.51165200 1.35626000 -0.09612900  H -1.64895800 1.99086600 -0.09229600  C -0.59495200 -0.82501000 -0.00824500  C -5.63569000 -0.67931400 -0.11282700  H -5.98390300 -0.77897700 -1.15429800  H -5.98684200 -1.56657800 0.43510700  C -6.14413300 0.67097900 0.46244100  H -7.19790800 0.85727700 0.19763300  H -6.07600400 0.65599800 1.56097700  B -5.19067200 1.83883500 -0.17765000  H -5.06128200 2.87075100 0.43688500  H -5.32022600 2.00429900 -1.37329500  H -3.47181200 -2.59259500 0.01864400  Au 1.28710000 -0.24178900 0.00378000  Br 3.73462100 0.52669500 0.01866600 |
|   B3LYP/LANL2DZ | Free Energy: -480.941344  Enthalpy: -480.884597  ZPE Corrected Energy: -480.898669  Total Energy: -481.0454531  Frequency: -243.6706  C -2.71392000 -1.70995100 -0.01777300  C -3.59632500 -0.64409300 -0.09042800  C -3.02945300 0.70366500 -0.12618200  C -1.34516200 -1.37245500 -0.00337100  C -1.75695100 0.98894400 -0.08650900  H -0.97562000 1.72496900 -0.10728100  C -0.30310600 -0.66512700 -0.00281800  C -5.10820100 -0.62747000 -0.10564100  H -5.44974200 -0.71365200 -1.15023700  H -5.54087700 -1.47164100 0.45021700  C -5.48501100 0.78523100 0.43561700  H -6.50167200 1.07517300 0.13312000  H -5.45714000 0.77688300 1.53615300  B -4.35734700 1.81510000 -0.18888900  H -4.14107000 2.84037900 0.41570800  H -4.49608300 2.01395900 -1.38383100  H -3.05197400 -2.74018800 0.03968300  Au 1.57667800 -0.09512000 0.00776100  S 3.99188600 0.65178600 0.02189600  H 4.50354400 -0.11330300 -1.00243300  H 4.49841900 -0.13464300 1.03248400 |
|   B3LYP/LANL2DZ | Total Energy: -596.0112124  Enthalpy: -595.746507  Free energy: -595.814769  ZPE Correction: -595.765640  Frequency: -192.0872  C 3.45211500 -1.65615800 0.01040700  C 4.28821300 -0.55699600 0.08472900  C 3.65559800 0.76368800 0.12698400  C 2.06644400 -1.38098600 -0.00010700  C 2.37063000 0.97737500 0.09213800  H 1.53071700 1.64425800 0.11267900  C 0.95384200 -0.79293700 0.00318900  C 5.79777900 -0.46529300 0.09878300  H 6.14623900 -0.54026800 1.14196500  H 6.27084800 -1.28360200 -0.46310800  C 6.10383100 0.96665600 -0.43605300  H 7.10515100 1.30561800 -0.13255400  H 6.07577700 0.96151600 -1.53675200  B 4.92603200 1.93772000 0.19126200  H 3.83213500 -2.67177300 -0.04929400  Au -0.97036000 -0.27596400 -0.00336600  P -3.31393500 0.35043300 -0.01175100  C -3.69999300 1.90722200 -0.99299800  H -4.77155100 2.13308700 -0.95017200  H -3.13480400 2.74831600 -0.58037600  H -3.39881500 1.76612700 -2.03529500  C -4.46051700 -0.94741800 -0.74390600  H -4.37979100 -1.87316400 -0.16624800  H -5.49892100 -0.59755900 -0.72739100  H -4.16310300 -1.15588300 -1.77596400  C -4.03944000 0.69260200 1.68864700  H -5.09756800 0.96603500 1.60743100  H -3.94034000 -0.19996800 2.31359300  H -3.48786100 1.50978400 2.16305000  H 4.65972800 2.95265800 -0.41249200  H 5.05848100 2.14431900 1.38589000 |
|   B3LYP/LANL2DZ | Free Energy: -703.610162  Enthalpy: -703.543313  ZPE Corrected Energy: -703.561374  Total Energy: -703.7336314  Frequency: -248.9831  C -3.43009000 -1.65632200 0.03677700  C -4.24730400 -0.54070000 -0.05883100  C -3.60141200 0.76834700 -0.14742500  C -2.04470800 -1.40066600 0.02167300  C -2.31498000 0.98199300 -0.13569600  H -1.49351600 1.66956600 -0.20388800  C -0.96256600 -0.75705200 -0.01240900  C -5.75517200 -0.43294100 -0.05304000  H -6.11717200 -0.52926000 -1.08982800  H -6.22918200 -1.23247600 0.53402500  C -6.03754500 1.01551700 0.44943400  H -7.03971600 1.35722300 0.15292700  H -5.99198600 1.03886600 1.54910500  B -4.86234200 1.95700800 -0.22415200  H -4.57389900 2.98457500 0.34527200  H -5.00780300 2.12735900 -1.42230700  H -3.82907600 -2.66149500 0.13291300  Au 0.95885800 -0.29695900 -0.02487600  P 3.29295400 0.33933700 0.02303400  O 3.97650700 1.19351000 -1.27626800  H 4.32276500 2.09304600 -1.08725600  O 3.80097500 1.46526900 1.22088800  H 3.41814800 1.42957000 2.12122500  O 4.32065300 -0.99269700 0.18799700  H 5.28667700 -0.89001300 0.04442400 |
|  |  |
| B3LYP/LANL2DZ | Free Energy: -768.302713  Enthalpy: -768.244876  ZPE Corrected Energy: -768.259360  Total Energy: -768.3778826  Frequency: -151.7795  C -1.66343100 1.97434400 0.01869400  C -2.80567600 1.20389000 0.02649600  C -2.64358400 -0.24373900 0.02770300  C -0.36238000 1.41978800 0.02969100  C -1.68789600 -1.06639000 0.03692900  H -1.01383000 -1.89783700 0.05366000  C 0.75328700 0.85243700 0.01768500  C -4.25497900 1.65740700 0.02629300  H -4.59675900 1.81913900 1.06078400  H -4.38234700 2.60695500 -0.51129100  C -5.06339900 0.49216000 -0.59677600  H -6.13292700 0.55410400 -0.34511500  H -4.96720500 0.49712900 -1.69148600  B -4.45626500 -0.88183200 -0.02724100  H -1.77378800 3.06053900 -0.00734300  F -4.54499100 -2.04223000 -0.83657500  F -4.75142100 -1.18226600 1.33500500  Au 2.48762300 -0.02982000 -0.00164600  F 4.29577700 -0.95489300 -0.03323900 |
| B3LYP/LANL2DZ | Free Energy: -683.431950  Enthalpy: -683.372594  ZPE Corrected Energy: -683.387437  Total Energy: -683.5052953  Frequency: -140.6115  C -1.97619100 2.00327900 0.00916600  C -3.08157500 1.18224400 0.02286900  C -2.85300000 -0.25827100 0.01526400  C -0.65431000 1.49600000 0.00622400  C -1.84509900 -1.01821000 0.01672400  H -1.12413500 -1.80984700 0.01557800  C 0.46617600 0.93932800 0.00678800  C -4.55080800 1.56470200 0.04089400  H -4.88672200 1.70282900 1.08060700  H -4.73054500 2.51031400 -0.48842200  C -5.30686100 0.36335900 -0.57931300  H -6.37460200 0.36862000 -0.31383900  H -5.22505200 0.37923800 -1.67511900  B -4.61936500 -0.98001700 -0.02449300  H -2.13053700 3.08371900 -0.00740200  F -4.66068400 -2.13941000 -0.83996700  F -4.88835400 -1.30185000 1.33937100  Au 2.24056500 0.10229000 0.00027000  Cl 4.43030200 -0.94582100 -0.01391900 |
| B3LYP/LANL2DZ | Free Energy: -681.655129  Enthalpy: -681.594651  ZPE Corrected Energy: -681.609699  Total Energy: -681.7272235  Frequency: -138.2960  C -2.50448000 2.00361200 0.00799000  C -3.57639400 1.13976200 0.02259000  C -3.29040800 -0.29097200 0.01526200  C -1.16430800 1.54569900 0.00477900  C -2.24776300 -1.00350400 0.01639400  H -1.49315600 -1.76325600 0.01492800  C -0.02353900 1.03149400 0.00503500  C -5.05978600 1.46233300 0.04078300  H -5.40035200 1.58663200 1.08071100  H -5.27786000 2.39997500 -0.48827500  C -5.76606600 0.23075000 -0.57894800  H -6.83270800 0.19150700 -0.31216500  H -5.68621100 0.25024800 -1.67484300  B -5.02168500 -1.08265700 -0.02451900  H -2.69955100 3.07728800 -0.00886600  F -5.01566900 -2.24294200 -0.84016800  F -5.27888300 -1.41548600 1.33944100  Au 1.79206700 0.26977500 0.00087300  Br 4.15349600 -0.72658600 -0.00727500 |
| B3LYP/LANL2DZ | Free Energy: -744.726052  Enthalpy: -744.666277  ZPE Corrected Energy: -744.681847  Total Energy: -744.8240431  Frequency: -193.2177  C -1.77987300 2.19861900 0.01087600  C -2.80838200 1.27300700 0.02336800  C -2.43146400 -0.13552800 0.00528000  C -0.47057700 1.67272500 0.00127800  C -1.21994700 -0.60065600 -0.00967900  H -0.53955900 -1.43074600 -0.01817500  C 0.49067600 0.86200100 -0.00621500  C -4.30881700 1.47824300 0.03873500  H -4.63870500 1.60298100 1.08163200  H -4.60755400 2.38275400 -0.50722100  C -4.90616400 0.16135600 -0.53552600  H -5.94014600 0.00307100 -0.20207300  H -4.89985900 0.18475700 -1.63458400  B -3.94430400 -1.04115000 -0.02134800  H -1.96481300 3.26864900 0.00117600  F -3.84435500 -2.19431900 -0.86167500  F -4.18259200 -1.43623700 1.34790700  Au 2.23548300 0.00516300 -0.00282300  O 4.16282500 -0.94889000 0.00606500  H 4.60832600 -1.23357200 0.82252300  H 4.64791100 -1.17250700 -0.80681900 |
| B3LYP/LANL2DZ | Free Energy: -679.599060  Enthalpy: -679.537538  ZPE Corrected Energy: -679.553372  Total Energy: -679.6883475  Frequency: -207.0995  C -2.08867600 2.19143200 0.01143800  C -3.08290800 1.22867600 0.02426900  C -2.65801700 -0.16578000 0.00561400  C -0.76067900 1.71486800 0.00117400  C -1.43739200 -0.60095900 -0.00957000  H -0.73101400 -1.40858600 -0.01798000  C 0.22258200 0.93065600 -0.00683700  C -4.58939500 1.38274700 0.04107200  H -4.92206400 1.49434900 1.08455200  H -4.91904000 2.27762100 -0.50277700  C -5.14321700 0.04834700 -0.53539100  H -6.17218100 -0.14288200 -0.20370400  H -5.13608100 0.07278600 -1.63433000  B -4.14699600 -1.12441200 -0.02096000  H -2.31500700 3.25359400 0.00171300  F -4.00577200 -2.27187300 -0.86109300  F -4.36748900 -1.52539600 1.34783000  Au 2.02059500 0.14149000 -0.00412000  S 4.34029600 -0.85934000 0.00354700  H 4.23386900 -1.74290400 1.05434000  H 4.21029700 -1.81472300 -0.97952000 |
| B3LYP/LANL2DZ | Total Energy: -794.6544733  Enthalpy: -794.400790  Free energy: -794.470199  ZPE Correction: -794.420790  Frequency: -152.2878  C 2.85627700 -2.16466200 -0.01405300  C 3.79815000 -1.15401900 -0.00183100  C 3.29447300 0.21657800 0.01144300  C 1.49946100 -1.76790300 0.00482600  C 2.05492300 0.58273500 0.02619700  H 1.27203200 1.31493400 0.04612700  C 0.43000900 -1.10808600 0.03108300  C 5.31152800 -1.22199900 -0.01121400  H 5.66943300 -1.33364400 1.02383100  H 5.68142200 -2.08634500 -0.57837300  C 5.77954500 0.15066300 -0.57214500  H 6.80227600 0.39416000 -0.25508600  H 5.75322700 0.14533400 -1.67114500  B 4.73027500 1.25783400 -0.02037000  H 3.14049900 -3.21285000 -0.04456800  F 4.51427200 2.41095700 -0.83711600  F 4.95104400 1.64755900 1.35143700  Au -1.42439700 -0.38197700 0.01855200  P -3.67178300 0.52912000 -0.02596000  C -4.67234400 0.08952500 -1.55528800  H -5.65537400 0.57252800 -1.52266500  H -4.13612600 0.41683600 -2.45086500  H -4.80158200 -0.99587100 -1.60609600  C -3.72361600 2.40788500 0.04242100  H -4.75878300 2.76723000 0.02213100  H -3.23943700 2.74997600 0.96218300  H -3.17685700 2.82147700 -0.81010600  C -4.76944500 0.00083200 1.40581800  H -4.32074400 0.33073700 2.34764200  H -5.76851500 0.43983400 1.30475100  H -4.85035300 -1.09007800 1.42542500 |
| B3LYP/LANL2DZ | Free Energy: -902.267466  Enthalpy: -902.195883  ZPE Corrected Energy: -902.215690  Total Energy: -902.3761246  Frequency: -220.5589  C 2.83336200 -2.17134700 0.04311800  C 3.76303500 -1.14487500 0.04834900  C 3.25144200 0.21865900 -0.00655800  C 1.47976000 -1.77943300 0.00611000  C 2.00791800 0.58068200 -0.04925900  H 1.25890800 1.34779700 -0.09423200  C 0.45576700 -1.04895800 -0.02749000  C 5.27555900 -1.20319700 0.09076900  H 5.59678700 -1.27308600 1.14145600  H 5.66949600 -2.08607900 -0.42925100  C 5.75432200 0.15196200 -0.50372400  H 6.76454000 0.41245200 -0.16184500  H 5.76489900 0.10733400 -1.60192700  B 4.68060400 1.26922800 -0.02425100  H 3.12827400 -3.21638100 0.06012800  F 4.48355900 2.39387200 -0.88162500  F 4.84925500 1.69834800 1.34324000  Au -1.40389200 -0.38100200 -0.03431300  P -3.65697200 0.50183600 0.01824500  O -4.00128400 1.80258600 1.08829600  H -3.59094400 1.83376100 1.97660900  O -4.30701300 1.26272100 -1.35266200  H -4.57341400 2.20370700 -1.26011000  O -4.79518900 -0.69512700 0.37718600  H -5.75397100 -0.51640300 0.26246800 |
| B3LYP/LANL2DZ | Free Energy: -842.629724  Enthalpy: -842.559046  ZPE Corrected Energy: -842.579051  Total Energy: -842.7800745  Frequency: -173.0439  C 2.84140800 -2.16734400 0.00109600  C 3.78212000 -1.15457600 0.02689900  C 3.27862600 0.21528200 0.01714200  C 1.48741300 -1.76513200 -0.01225600  C 2.03611100 0.57842300 -0.00009300  H 1.26700000 1.32602900 -0.00307000  C 0.43255100 -1.08089200 -0.01700200  C 5.29499800 -1.22244300 0.05224800  H 5.62824100 -1.32321400 1.09665700  H 5.67813500 -2.09263900 -0.49680400  C 5.77517900 0.14557700 -0.51053500  H 6.78979200 0.39276700 -0.17135800  H 5.77528400 0.12964800 -1.60973500  B 4.71086900 1.25593800 0.00650500  H 3.12456600 -3.21590300 -0.01482400  F 4.51235000 2.40081400 -0.82643300  F 4.90248200 1.65952400 1.37929000  Au -1.42411100 -0.36890300 -0.01887200  P -3.67797000 0.52394400 -0.00211100  N -4.84946400 -0.74144900 0.00572000  H -5.83988500 -0.55588000 0.12038900  H -4.58662400 -1.67732500 -0.27637800  N -3.89242300 1.67493000 -1.26749000  H -4.73213500 2.23801700 -1.34700900  H -3.24879700 1.69515100 -2.04849300  N -4.31974000 1.46945900 1.31082400  H -4.02151900 2.43322300 1.40933600  H -4.58664900 0.99759800 2.16723800 |
|  |  |
| B3LYP/LANL2DZ | Free Energy: -598.457072  Enthalpy: -598.396697  ZPE Corrected Energy: -598.412094  Total Energy: -598.5278427  Frequency: -150.7309  C -0.99270400 2.08963000 -0.23102600  C -2.22893900 1.48314000 -0.18223600  C -2.31317300 0.04126100 -0.08906100  C 0.25131100 1.42628100 -0.17829800  C -1.72984200 -1.06589800 -0.02062900  H -1.39400200 -2.07929700 0.04341900  C 1.34322500 0.82152400 -0.11629000  C -3.58937500 2.16159600 -0.21644700  H -3.90163700 2.43686400 0.80263000  H -3.56937300 3.08249100 -0.81427700  C -4.56963900 1.10397300 -0.77961000  H -5.62116600 1.36109700 -0.58686700  H -4.43899700 1.00880200 -1.86611100  B -4.23977300 -0.30626900 -0.09193700  H -0.98407500 3.17961300 -0.32511500  Au 3.05357000 -0.09533500 0.01129000  Cl -4.75074500 -1.85208100 -1.06055600  Cl -4.70494900 -0.43779400 1.74513500  F 4.84418800 -1.04088300 0.17309200 |
| B3LYP/LANL2DZ | Free Energy: -513.586567  Enthalpy: -513.524382  ZPE Corrected Energy: -513.540201  Total Energy: -513.6552361  Frequency: -150.2774  C -1.30649400 2.15661200 -0.19535200  C -2.51398600 1.49673900 -0.15392600  C -2.53200700 0.04944400 -0.08465600  C -0.03736100 1.53733300 -0.15671300  C -1.88530100 -1.02279500 -0.03573200  H -1.48987100 -2.01573500 0.01011400  C 1.06636400 0.95514600 -0.10984800  C -3.90356200 2.11306200 -0.17538900  H -4.22492100 2.35681000 0.84872500  H -3.92610300 3.04374700 -0.75756800  C -4.83577000 1.02153900 -0.75493300  H -5.89739900 1.22764300 -0.55661500  H -4.70340500 0.95062100 -1.84304500  B -4.44155400 -0.38381000 -0.09120400  H -1.34104600 3.24704500 -0.27116500  Au 2.80309200 0.05058200 -0.00938500  Cl -4.87950200 -1.93434700 -1.08414900  Cl -4.89248000 -0.56574000 1.74412400  Cl 4.95050900 -1.07224000 0.14980800 |
| B3LYP/LANL2DZ | Free Energy: -511.810099  Enthalpy: -511.746439  ZPE Corrected Energy: -511.762497  Total Energy: -511.8771764  Frequency: -150.5411  C -1.85203400 2.19231500 -0.18497100  C -3.03136500 1.48362100 -0.15043600  C -2.98956800 0.03695500 -0.06881200  C -0.55913700 1.62428100 -0.13184200  C -2.29361100 -1.00321600 -0.00339100  H -1.85242000 -1.97619600 0.05347200  C 0.56630500 1.08544300 -0.08346100  C -4.44506900 2.04078000 -0.19643300  H -4.79080900 2.28001300 0.82067900  H -4.49817400 2.96423600 -0.78800700  C -5.32201200 0.90558400 -0.77838800  H -6.39413600 1.06808700 -0.59695300  H -5.17117400 0.83056100 -1.86382800  B -4.87759900 -0.47548400 -0.09547200  H -1.92966900 3.27971200 -0.27041500  Au 2.34492500 0.24718300 -0.01439700  Cl -5.23634600 -2.05270800 -1.07727900  Cl -5.34406300 -0.65797300 1.73604700  Br 4.66289200 -0.84884000 0.07563200 |
| B3LYP/LANL2DZ | Free Energy: -574.885772  Enthalpy: -574.823009  ZPE Corrected Energy: -574.839630  Total Energy: -574.9789702  Frequency: -70.5372  C -1.13034300 2.30011500 -0.20148700  C -2.31254900 1.60538700 -0.16079100  C -2.23867200 0.13791900 -0.10358100  C 0.11453500 1.61781300 -0.16211000  C -1.26019700 -0.67275400 -0.07750100  H -0.62685200 -1.53584000 -0.03967300  C 1.14575900 0.92303900 -0.11040500  C -3.73964100 2.11390500 -0.17988400  H -4.05963700 2.33700000 0.84859500  H -3.83736300 3.03731300 -0.76450800  C -4.58008400 0.93504100 -0.73525300  H -5.64402700 1.01989300 -0.48129700  H -4.49641600 0.89201300 -1.82971100  B -3.96106200 -0.41521200 -0.10321500  H -1.14224200 3.38680100 -0.26853100  Au 2.81402400 -0.05068400 0.00671900  Cl -4.23384000 -2.00482000 -1.09548900  Cl -4.38092800 -0.65307600 1.75108200  O 4.68455400 -1.11098600 0.16786500  H 5.22028300 -1.36677000 -0.60247700  H 5.06548200 -1.38099300 1.02095500 |
| B3LYP/LANL2DZ | Free Energy: -509.758345  Enthalpy: -509.694169  ZPE Corrected Energy: -509.710960  Total Energy: -509.8431739  Frequency: -62.6159  C 1.45650300 2.35425500 0.17324900  C 2.60400000 1.60223200 0.14326800  C 2.45515500 0.13960800 0.09357000  C 0.18654200 1.71982400 0.13404800  C 1.42176700 -0.60397200 0.06826800  H 0.74689500 -1.43479000 0.03087000  C -0.86019700 1.04574100 0.08982100  C 4.05556800 2.03411800 0.17108400  H 4.39335900 2.23615100 -0.85607800  H 4.19865800 2.95295000 0.75348700  C 4.82755400 0.81239800 0.73530900  H 5.89523600 0.83700500 0.48497900  H 4.73785400 0.77964800 1.82970000  B 4.13230900 -0.50281000 0.10639900  H 1.51478600 3.43941800 0.23388500  Au -2.57527400 0.11262700 0.00710700  Cl 4.31134300 -2.10292100 1.10544800  Cl 4.55289400 -0.77176500 -1.74584000  S -4.81379800 -1.06386700 -0.11541500  H -4.61071100 -2.10467400 0.76305200  H -4.64359800 -1.82290900 -1.25172700 |
| B3LYP/LANL2DZ | Free Energy: -624.631088  Enthalpy: -624.555283  ZPE Corrected Energy: -624.577266  Total Energy: -624.8081018  Frequency: -106.0778  C 2.20804600 -2.34599700 -0.15075900  C 3.32382000 -1.54784400 -0.12394700  C 3.12824000 -0.09485800 -0.08671600  C 0.89810800 -1.79848100 -0.12209500  C 2.13169600 0.68454300 -0.06713200  H 1.45330500 1.51246900 -0.03951500  C -0.20517500 -1.21932200 -0.08939500  C 4.78807200 -1.94047600 -0.14257100  H 5.13243700 -2.11929900 0.88672000  H 4.95629200 -2.86277600 -0.71267500  C 5.53034800 -0.71151800 -0.72583600  H 6.60221200 -0.71643300 -0.48974100  H 5.42583700 -0.68827600 -1.81903100  B 4.83716500 0.60218700 -0.10085100  H 2.32973900 -3.42762500 -0.20270200  Au -1.99907300 -0.37837000 -0.02866000  P -4.19971500 0.64773400 0.06070500  C -4.36639700 2.02632600 1.32898100  H -5.38189000 2.43839100 1.32122300  H -4.14049400 1.63395800 2.32503000  H -3.65019800 2.82120300 1.10006000  C -5.59287100 -0.53818000 0.49587100  H -6.55272700 -0.01024400 0.52718700  H -5.63989200 -1.33461500 -0.25291300  H -5.39460700 -0.99145300 1.47174200  C -4.76807600 1.44064800 -1.54715700  H -4.78966100 0.68540200 -2.33854600  H -5.76745800 1.87512700 -1.43047700  H -4.06222700 2.22483900 -1.83696000  Cl 4.98323100 2.19314000 -1.11252800  Cl 5.23801000 0.88889500 1.74387600 |
| B3LYP/LANL2DZ | Free Energy: -732.425869  Enthalpy: -732.352497  ZPE Corrected Energy: -732.373198  Total Energy: -732.53093  Frequency: -58.0032  C 2.24107400 -2.40821000 -0.19849300  C 3.32467700 -1.56536200 -0.16651500  C 3.05377300 -0.12120200 -0.08125500  C 0.93357000 -1.86077200 -0.13190400  C 1.94169000 0.50543200 -0.02544300  H 1.20419300 1.27866700 0.05180900  C -0.14506500 -1.23715300 -0.06876100  C 4.80651800 -1.86944000 -0.22712900  H 5.17753800 -2.06334400 0.79014800  H 5.01857200 -2.75989000 -0.83219300  C 5.45863800 -0.57095300 -0.77350700  H 6.52692300 -0.50412300 -0.53496400  H 5.35265900 -0.52459100 -1.86603600  B 4.64898200 0.66003700 -0.10807800  H 2.37701500 -3.48415800 -0.28346300  Au -1.92963100 -0.41402200 0.00988200  P -4.10833700 0.64483600 0.04572300  Cl 4.66844800 2.29089700 -1.07708300  Cl 5.08410700 0.93121900 1.74387800  O -4.50409500 1.75195700 -1.21013700  H -4.21348600 1.57915400 -2.12882600  O -4.51534300 1.70556600 1.30716100  H -4.73984000 2.62972800 1.06116700  O -5.36550300 -0.48570700 0.06128900  H -6.28570500 -0.20099500 0.25223700 |
| B3LYP/LANL2DZ | Free Energy: -672.789040  Enthalpy: -672.714910  ZPE Corrected Energy: -672.736002  Total Energy: -672.9341049  Frequency: -91.9896  C 2.20578900 -2.35680700 -0.16481500  C 3.31610300 -1.55145400 -0.12995900  C 3.10751700 -0.09907800 -0.07700400  C 0.89656100 -1.80779400 -0.13031800  C 2.08495000 0.64943100 -0.04875100  H 1.38899900 1.46241200 -0.00851700  C -0.20126700 -1.21899600 -0.09155200  C 4.78389600 -1.92831800 -0.15478800  H 5.13052400 -2.11438500 0.87245000  H 4.96132000 -2.84268900 -0.73478600  C 5.51118400 -0.68361000 -0.72444900  H 6.58219400 -0.67602900 -0.48555700  H 5.40897800 -0.65127500 -1.81766600  B 4.79360500 0.61249100 -0.08737000  H 2.32921000 -3.43724400 -0.22791000  Au -1.99157000 -0.37900400 -0.03446800  P -4.18772100 0.65355200 0.05179800  Cl 4.91891400 2.21625800 -1.08326200  Cl 5.19777000 0.88680000 1.76111400  N -4.36785000 1.84302200 -1.18503900  H -3.74331100 1.84164500 -1.98156000  H -5.18091400 2.44689600 -1.23730600  N -4.75007800 1.60840100 1.39638700  H -4.39651300 2.55206700 1.50502400  H -5.02047300 1.13469400 2.25066800  N -5.43268100 -0.54082100 0.05957600  H -5.22765200 -1.48561200 -0.24006500  H -6.40830500 -0.30133000 0.19794100 |
|  |  |
| B3LYP/LANL2DZ | Free Energy: -594.883325  Enthalpy: -594.819920  ZPE Corrected Energy: -594.835889  Total Energy: -594.9505478  Frequency: -147.3216  C -0.01074000 1.98903400 -1.13384600  C -1.29114100 1.52192800 -0.93148000  C -1.49730000 0.25115900 -0.27370700  C 1.17956500 1.34191000 -0.74243900  C -1.04662600 -0.77963800 0.27772700  H -0.82624900 -1.70395400 0.76821300  C 2.23184700 0.76018000 -0.40208600  C -2.58970800 2.19887900 -1.34236100  H -2.92887600 2.88573000 -0.55207800  H -2.46675300 2.78796400 -2.26083800  C -3.62155200 1.05594400 -1.50307800  H -4.65798000 1.42149400 -1.52215200  H -3.43797600 0.51576000 -2.44199200  B -3.44646400 0.03510500 -0.27472600  Br -4.12290500 -1.88989900 -0.61400400  Br -4.06495300 0.76979700 1.56276700  H 0.08281700 2.94452900 -1.65894900  Au 3.88897000 -0.13105700 0.08839900  F 5.63196400 -1.05625500 0.57320100 |
| B3LYP/LANL2DZ | Free Energy: -510.012354  Enthalpy: -509.947695  ZPE Corrected Energy: -509.964034  Total Energy: -510.0780158  Frequency: -145.805  C -0.31657200 2.14864400 -1.01799300  C -1.57520000 1.62196000 -0.83492400  C -1.72306200 0.29696500 -0.27154400  C 0.89875900 1.50896200 -0.68941400  C -1.20996400 -0.74836600 0.19042100  H -0.93376400 -1.69541400 0.60371600  C 1.96233900 0.92207100 -0.40061400  C -2.90418600 2.27794000 -1.17538900  H -3.25294100 2.89185600 -0.33124300  H -2.81850500 2.93708700 -2.04927200  C -3.89689800 1.11241000 -1.40407500  H -4.94577100 1.44001100 -1.38001700  H -3.71185700 0.64937900 -2.38309200  B -3.66352500 0.01197600 -0.25662900  Br -4.27378900 -1.90627800 -0.72243700  Br -4.26578600 0.59142900 1.63967600  H -0.26072600 3.14358800 -1.46903300  Au 3.64679300 0.00976200 0.01665600  Cl 5.74225600 -1.11890700 0.49965300 |
| B3LYP/LANL2DZ | Free Energy: -508.235960  Enthalpy: -508.169699  ZPE Corrected Energy: -508.186292  Total Energy: -508.299908  Frequency: -147.1575  C -0.86259900 2.32092200 -0.86564500  C -2.09306600 1.72669600 -0.70057100  C -2.17017900 0.34835600 -0.26444700  C 0.38491200 1.70184800 -0.62851500  C -1.59891400 -0.71150700 0.08097700  H -1.26924300 -1.67956600 0.39454600  C 1.47195400 1.12749100 -0.41090900  C -3.45723400 2.35479600 -0.93784500  H -3.80473900 2.86315100 -0.02580200  H -3.42570800 3.10223900 -1.74137000  C -4.40791900 1.17558500 -1.25734500  H -5.46799600 1.45404000 -1.17554400  H -4.23201200 0.81916500 -2.28168600  B -4.09837000 -0.02055300 -0.22984100  Br -4.64128700 -1.90882400 -0.86670400  Br -4.66802700 0.34394900 1.72840400  H -0.85841300 3.35552400 -1.22020100  Au 3.18820300 0.22690400 -0.07317000  Br 5.42589000 -0.94947800 0.36359800 |
| B3LYP/LANL2DZ | Free Energy: -571.313009  Enthalpy: -571.246647  ZPE Corrected Energy: -571.263975  Total Energy: -571.4020247  Frequency: -88.7775  C -0.16237500 2.29847300 -1.03181200  C -1.40755400 1.75318700 -0.85431700  C -1.49253000 0.38618300 -0.32198000  C 1.01987100 1.58542700 -0.69831000  C -0.64565900 -0.48492400 0.03878700  H -0.12421300 -1.34841000 0.39923100  C 2.00307500 0.89364000 -0.38166100  C -2.76923200 2.34937400 -1.15003800  H -3.11380600 2.92600300 -0.27922800  H -2.73765700 3.03215600 -2.00837600  C -3.70406200 1.13211600 -1.36414400  H -4.76494700 1.38869700 -1.25423700  H -3.56082600 0.71890000 -2.37207500  B -3.28492300 0.01825700 -0.27177400  Br -3.75117000 -1.93106800 -0.75598600  Br -3.84914100 0.51141000 1.67430100  H -0.07358600 3.30137800 -1.44795700  Au 3.60435200 -0.09626400 0.06476500  O 5.41313300 -1.17657100 0.54122300  H 5.92248200 -1.67298600 -0.12194100  H 5.78997500 -1.21248800 1.43703200 |
| B3LYP/LANL2DZ | Free Energy: -506.185226  Enthalpy: -506.117956  ZPE Corrected Energy: -506.135393  Total Energy: -506.2663808  Frequency: -77.7120  C -0.47910000 2.45520900 -0.88651700  C -1.69817800 1.84709400 -0.73108800  C -1.71515100 0.44038700 -0.30360800  C 0.73075400 1.75833300 -0.62446200  C -0.81112200 -0.40609900 -0.02727700  H -0.24377100 -1.26851900 0.25813100  C 1.73038500 1.06280900 -0.36956600  C -3.08973300 2.40210800 -0.95702000  H -3.43994900 2.89449000 -0.03820100  H -3.10608500 3.14900300 -1.76052900  C -3.97510300 1.16301600 -1.24681300  H -5.04314100 1.36164000 -1.09525700  H -3.83823200 0.83638600 -2.28696800  B -3.47831500 -0.01141000 -0.25346900  Br -3.86341200 -1.93834400 -0.88040900  Br -4.02667300 0.29948300 1.73573400  H -0.43344800 3.48910400 -1.22578000  Au 3.37297900 0.07055200 -0.00461600  S 5.52900200 -1.18255300 0.44877500  H 5.26910800 -2.38034500 -0.17903900  H 5.29418100 -1.65450100 1.72098300 |
| B3LYP/LANL2DZ | Total Energy: -621.2310417  Enthalpy: -620.978774  Free energy: -621.057349  ZPE Correction: -621.001332  Frequency: -116.7710  C -1.22562000 2.55281700 -0.75153100  C -2.41204900 1.87903800 -0.61198200  C -2.36464500 0.45273100 -0.27871300  C 0.03331000 1.92297800 -0.56495900  C -1.48245200 -0.42538600 -0.06380700  H -0.91092600 -1.30397400 0.15503500  C 1.09311000 1.29481800 -0.38095500  C -3.82783100 2.39747100 -0.77382500  H -4.17919500 2.81396200 0.18156500  H -3.88601300 3.19577900 -1.52429100  C -4.67676100 1.15341300 -1.13641400  H -5.74968900 1.30996500 -0.96698600  H -4.53795600 0.89781400 -2.19588100  B -4.15148700 -0.07022700 -0.22499500  Br -4.49119800 -1.95965300 -0.97322100  Br -4.66377000 0.09485700 1.78394700  H -1.24972700 3.60812300 -1.02376900  Au 2.82319500 0.36996700 -0.09998300  P 4.95461000 -0.74591400 0.24377500  C 6.42720900 0.41940500 0.34724800  H 7.35520800 -0.14274000 0.50184500  H 6.50108700 0.99607100 -0.57966000  H 6.28140700 1.11648100 1.17791500  C 5.43351300 -1.96099400 -1.10956000  H 6.40744300 -2.41508500 -0.89431600  H 4.67415600 -2.74555200 -1.18011500  H 5.47995200 -1.43742800 -2.06916700  C 5.06831600 -1.75954800 1.82368600  H 4.30315900 -2.54161000 1.81127600  H 6.05818000 -2.22057600 1.91692700  H 4.88608700 -1.11137300 2.68610900 |
| B3LYP/LANL2DZ | Free Energy: -728.852881  Enthalpy: -728.776448  ZPE Corrected Energy: -728.797771  Total Energy: -728.9543029  Frequency: -55.8411  C -1.46009 2.88616 -0.57745  C -2.60111 2.11559 -0.48092  C -2.30046 0.68559 -0.30301  C -0.30814 2.05383 -0.45915  C -0.99451 0.36400 -0.26971  H -0.44876 -0.56031 -0.18573  C 0.80614 1.45708 -0.33635  C -4.08465 2.37408 -0.53517  H -4.42567 2.67058 0.46879  H -4.35209 3.18537 -1.22449  C -4.70729 0.98160 -0.90138  H -5.74097 0.88773 -0.55335  H -4.70787 0.85730 -1.99423  B -3.70460 -0.12049 -0.23940  Br -3.66753 -1.98764 -1.18267  Br -4.14635 -0.34298 1.85718  H -1.38864 3.95744 -0.71410  Au 2.51029 0.48597 -0.09985  P 4.55061 -0.77815 0.22974  O 5.50210 -1.17514 -1.11259  H 5.66511 -2.12727 -1.29204  O 4.43898 -2.37616 0.83470  H 3.79535 -2.61117 1.53435  O 5.60892 0.04345 1.25450  H 6.54061 -0.24960 1.35783 |
| B3LYP/LANL2DZ | Free Energy: -669.215660  Enthalpy: -669.138483  ZPE Corrected Energy: -669.160182  Total Energy: -669.3571308  Frequency: -106.3270  C -1.23023 2.57345 -0.74144  C -2.41022 1.88783 -0.60618  C -2.34786 0.45942 -0.27786  C 0.03083 1.94758 -0.55413  C -1.43970 -0.39542 -0.06872  H -0.84763 -1.26152 0.14619  C 1.08710 1.31428 -0.36974  C -3.83164 2.38946 -0.76852  H -4.18839 2.79889 0.18794  H -3.89866 3.18923 -1.51666  C -4.66432 1.13519 -1.13450  H -5.73881 1.27594 -0.96220  H -4.52412 0.88561 -2.19523  B -4.11656 -0.08337 -0.22810  Br -4.43085 -1.97545 -0.98141  Br -4.63403 0.06905 1.78235  H -1.26007 3.62938 -1.00941  Au 2.81185 0.38647 -0.09442  P 4.92785 -0.75699 0.24215  N 5.94851 -0.58872 -1.13863  H 5.75403 0.12829 -1.82594  H 6.83142 -1.08062 -1.22054  N 5.07173 -2.48106 0.45013  H 4.97670 -3.08034 -0.36158  H 4.80636 -2.88949 1.33883  N 5.67182 -0.26859 1.72039  H 5.36789 0.57910 2.18285  H 6.51298 -0.71186 2.07336 |
|  |  |
| B3LYP/LANL2DZ | Total Energy: -754.2349893  Enthalpy: -754.087688  Free energy: -754.150315  ZPE Correction: -754.104313  Frequency: -140.4791  C -1.23021800 2.06495400 -0.27020100  C -2.45650000 1.43807400 -0.20630700  C -2.50970500 0.00029800 -0.07970900  C 0.01942400 1.41725300 -0.20321300  C -1.92314200 -1.10476800 0.01886100  H -1.56920200 -2.11110600 0.10466600  C 1.11496600 0.81934300 -0.12802600  C -3.83541500 2.07585500 -0.25722600  H -4.15823800 2.36672000 0.75496500  H -3.84363900 2.98407700 -0.87438400  C -4.77804800 0.97330800 -0.80041100  H -5.83690400 1.20537100 -0.61373600  H -4.64461100 0.86353700 -1.88447300  B -4.39822400 -0.42531800 -0.06266900  H -1.23732000 3.15215800 -0.39006400  Au 2.82718800 -0.08763500 0.01509900  C -4.74529300 -1.74236500 -0.84154200  C -4.76793200 -0.50722900 1.46343200  N -5.05242300 -2.71485100 -1.44214200  N -5.08411200 -0.54266400 2.60373000  F 4.61778800 -1.02407900 0.19094400 |
| B3LYP/LANL2DZ | Total Energy: -669.3620307  Enthalpy: -669.215032  Free energy: -669.279492  ZPE Correction: -669.232089  Frequency: -140.5372  C -1.54686700 2.12087400 -0.23131700  C -2.74284800 1.44000100 -0.17421000  C -2.72945100 -0.00243000 -0.07373000  C -0.27171300 1.51872400 -0.18079000  C -2.08131200 -1.07399900 0.00331000  H -1.67082400 -2.06042700 0.06903200  C 0.83688000 0.94556000 -0.12262600  C -4.14961600 2.01407000 -0.21032500  H -4.48213300 2.26816200 0.80848100  H -4.20101600 2.93356100 -0.80816600  C -5.04116200 0.88008500 -0.77439800  H -6.10916300 1.05978100 -0.58261000  H -4.90458500 0.79741300 -1.86043300  B -4.59690000 -0.51322000 -0.06319200  H -1.59825200 3.20847400 -0.33123800  Au 2.57939100 0.05686600 -0.00819100  C -4.88175400 -1.83043200 -0.86563700  C -4.95723600 -0.63899800 1.46185600  N -5.14252500 -2.80518200 -1.48410700  N -5.26722000 -0.70846900 2.60225700  Cl 4.73160400 -1.04579100 0.16753900 |
| B3LYP/LANL2DZ | Total Energy: -667.5838739  Enthalpy: -667.437006  Free energy: -667.503016  ZPE Correction: -667.454313  Frequency: -140.0528  C 2.09191300 -2.14225000 -0.21996400  C 3.25927000 -1.41350200 -0.17116200  C 3.18727800 0.02678700 -0.05850400  C 0.79377700 -1.59175500 -0.15419800  C 2.49071000 1.06629300 0.03521500  H 2.03590200 2.03233900 0.11220000  C -0.33695300 -1.06373100 -0.09500100  C 4.68797400 -1.92800000 -0.23335200  H 5.04647100 -2.17693700 0.77781600  H 4.76820600 -2.83902200 -0.84083200  C 5.52222000 -0.75234900 -0.80001000  H 6.59969700 -0.88855500 -0.62657200  H 5.36504700 -0.66539800 -1.88293700  B 5.03029500 0.61383700 -0.06767500  H 2.18571700 -3.22604200 -0.32907200  Au -2.12383900 -0.24696200 -0.01472200  C 5.24885400 1.95058500 -0.85829500  C 5.40605100 0.73721700 1.45389600  N 5.45997000 2.94242700 -1.46844300  N 5.72858100 0.80578500 2.59086700  Br -4.45130500 0.81803500 0.08691700 |
| B3LYP/LANL2DZ | Total Energy: -730.6823642  Enthalpy: -730.511165  Free energy: -730.574120  ZPE Correction: -730.528262  Frequency: -93.0535  C -1.36211400 2.24436900 -0.24067200  C -2.53226500 1.53042900 -0.18216100  C -2.42721700 0.07128900 -0.09141400  C -0.10609300 1.58416600 -0.18972900  C -1.45704300 -0.74582100 -0.04084100  H -0.82016400 -1.60578100 0.01382200  C 0.93317000 0.90269100 -0.12397900  C -3.97125300 2.00286200 -0.20840200  H -4.29833100 2.23279600 0.81721500  H -4.09430200 2.91654700 -0.80324600  C -4.77959900 0.79626000 -0.75210000  H -5.84855000 0.87531900 -0.51500900  H -4.68149500 0.73693500 -1.84382400  B -4.14671300 -0.54837100 -0.06849900  H -1.39220500 3.32898300 -0.33205500  Au 2.61760200 -0.04172900 0.00928800  C -4.29210800 -1.88472000 -0.87794100  C -4.47450700 -0.72249900 1.46290200  N -4.41750200 -2.88038400 -1.50488700  N -4.73516400 -0.82490800 2.61316900  O 4.50300900 -1.06820900 0.18890600  H 4.58384500 -2.03719100 0.21113600  H 5.35299000 -0.60055700 0.25749600 |
| B3LYP/LANL2DZ | Total Energy: -665.5466722  Enthalpy: -665.381469  Free energy: -665.448526  ZPE Correction: -665.399643  Frequency: -94.1344  C 1.68670300 -2.27901400 -0.20750900  C 2.82239800 -1.51027600 -0.16036200  C 2.64887400 -0.05667300 -0.08220800  C 0.40362600 -1.67179400 -0.15988000  C 1.63889600 0.71143000 -0.03650800  H 0.96515300 1.54312900 0.01199900  C -0.65671000 -1.02136600 -0.10427700  C 4.28201000 -1.91315300 -0.19110200  H 4.62464800 -2.11822900 0.83469000  H 4.44556300 -2.82505800 -0.77874600  C 5.02873200 -0.67356500 -0.74920400  H 6.10141800 -0.69976900 -0.51764500  H 4.92236400 -0.62810900 -1.84081300  B 4.33575300 0.64461300 -0.07237700  H 1.76530200 -3.36181200 -0.28917300  Au -2.38749900 -0.11862100 -0.00718000  C 4.41069100 1.98074400 -0.89151500  C 4.66078200 0.84509100 1.45629900  N 4.48105700 2.97761000 -1.52506200  N 4.91953000 0.96783500 2.60499000  S -4.64419800 1.01928900 0.13560400  H -4.46419900 2.06828900 -0.73812500  H -4.47950900 1.77430200 1.27537700 |
| B3LYP/LANL2DZ | Total Energy: -780.5119398  Enthalpy: -780.243869  Free energy: -780.319468  ZPE Correction: -780.266305  Frequency: -124.5207  C 2.44759100 -2.27503600 -0.18252300  C 3.54580500 -1.45300000 -0.14060600  C 3.31357100 -0.01129900 -0.07624400  C 1.12686200 -1.75559300 -0.14479300  C 2.32740500 0.77801500 -0.03647100  H 1.64731600 1.60475600 0.00144400  C 0.01461600 -1.19454800 -0.10183900  C 5.02088800 -1.80154000 -0.16461100  H 5.37238300 -1.98247500 0.86271300  H 5.21845400 -2.71292400 -0.74263700  C 5.72389800 -0.54493100 -0.73870000  H 6.79917300 -0.53609700 -0.51532800  H 5.60828500 -0.51171200 -1.82972700  B 5.00811200 0.76038200 -0.06984700  H 2.59267800 -3.35264500 -0.25498400  Au -1.79094800 -0.37763200 -0.02919200  P -4.00422100 0.62081400 0.06827100  C -4.21454200 1.92338500 1.40823300  H -5.23613200 2.32006400 1.40358300  H -4.00102600 1.47937700 2.38513200  H -3.50720100 2.74058100 1.23768300  C -5.39244700 -0.60267000 0.40289700  H -6.36075200 -0.08995900 0.42497300  H -5.40311300 -1.36668400 -0.38024200  H -5.21993100 -1.09511000 1.36463300  C -4.54086800 1.49815300 -1.50602300  H -4.54137300 0.78720000 -2.33768000  H -5.54462700 1.92199500 -1.38829800  H -3.83296200 2.29988300 -1.73678300  C 5.04048900 2.09071100 -0.89903400  C 5.31499100 0.97956400 1.45792000  N 5.08433000 3.08451300 -1.53971000  N 5.56522500 1.11909900 2.60651400 |
| B3LYP/LANL2DZ | Total Energy: -888.234539  Enthalpy: -888.039891  Free energy: -888.116001  ZPE Correction: -888.061924  Frequency: -80.8362  C -2.46379200 -2.32600700 0.14730500  C -3.53627100 -1.46986100 0.10982400  C -3.24672200 -0.03113800 0.08356700  C -1.14556300 -1.79985800 0.13778200  C -2.15277600 0.62215600 0.08458200  H -1.40994000 1.39516200 0.08312000  C -0.05540100 -1.19530000 0.11707600  C -5.02301600 -1.75431600 0.10520900  H -5.35986600 -1.90558800 -0.93189200  H -5.27048100 -2.66493300 0.66496900  C -5.67733000 -0.47113800 0.68180900  H -6.74192800 -0.39987900 0.42521500  H -5.59501600 -0.46470100 1.77649500  B -4.85050300 0.79855000 0.05918400  H -2.61812100 -3.40225400 0.19042600  Au 1.73405500 -0.38037700 0.06704400  P 3.93601500 0.61942200 -0.07873500  O 3.87046700 2.29489700 0.13923100  H 4.69235100 2.81697500 0.26572300  O 5.16803600 0.11460500 0.97314600  H 5.99773200 -0.21695400 0.56445100  O 4.85997200 0.38408100 -1.51028700  H 4.42337500 0.39090200 -2.38642800  C -4.84215100 2.11563100 0.91272900  C -5.12955900 1.06529200 -1.47030800  N -4.85272000 3.09815900 1.57197500  N -5.35419700 1.23548400 -2.62020300 |
| B3LYP/LANL2DZ | Total Energy: -828.6378398  Enthalpy: -828.402427  Free energy: -828.478961  ZPE Correction: -828.424850  Frequency: -116.6846  C 2.44371600 -2.28191700 -0.19398700  C 3.53634000 -1.45322300 -0.14237000  C 3.29203500 -0.01264300 -0.06497800  C 1.12276500 -1.76264900 -0.15424000  C 2.28217000 0.74866300 -0.01953800  H 1.58531500 1.56101100 0.02959600  C 0.01499400 -1.19382000 -0.10705800  C 5.01412200 -1.78781600 -0.16722300  H 5.36485800 -1.97392000 0.85944100  H 5.22120700 -2.69261200 -0.75220200  C 5.70529900 -0.51884000 -0.72877400  H 6.77940400 -0.49968200 -0.50133600  H 5.59315700 -0.47833000 -1.81994500  B 4.96865600 0.77196300 -0.05149100  H 2.59131100 -3.35822000 -0.27513400  Au -1.78782300 -0.38012800 -0.03846400  P -3.99525200 0.62721700 0.06237500  C 4.98760900 2.10868600 -0.87123400  C 5.27541600 0.98375600 1.47788300  N 5.02043500 3.10699400 -1.50552300  N 5.52515900 1.11680700 2.62736300  N -4.20364500 1.79374800 -1.19131800  H -3.58888900 1.78659200 -1.99540700  H -5.02333100 2.38871800 -1.24279800  N -4.54745700 1.59739000 1.39929600  H -4.20557600 2.54753800 1.48649500  H -4.80286900 1.13577200 2.26470200  N -5.22595900 -0.58054100 0.10743200  H -5.01572000 -1.52731600 -0.18214400  H -6.20189900 -0.34949000 0.25756500 |
|  |  |
| B3LYP/LANL2DZ  B3LYP/6-311++G**  PBE0/6-311++G**  PBE0/LANL2DZ | Total Energy: -230.8467257  Enthalpy: -230.768371  Free energy: -230.805009  ZPE Correction: -230.775438  C 0.68351200 1.08742800 -0.00001700  C -0.68350900 1.08742700 -0.00001500  C -1.51664200 -0.07233900 0.00000200  C 1.51664600 -0.07233700 -0.00000100  C -2.26828900 -1.04141500 0.00000700  H -2.91200700 -1.89217200 -0.00001500  C 2.26828400 -1.04141900 0.00000500  H 2.91199100 -1.89218500 0.00000600  H 1.19461800 2.05014400 0.00006200  H -1.19461500 2.05014300 0.00006400  Total Energy: -230.9511976  Enthalpy: -230.873419  Free energy: -230.910120  ZPE Correction: -230.880559  C 0.67574800 1.07820600 -0.00000400  C -0.67574700 1.07820400 -0.00000400  C -1.49685000 -0.07378800 -0.00000100  C 1.49686000 -0.07378400 0.00000400  C -2.23161500 -1.03007500 0.00000200  H -2.86706500 -1.88173900 -0.00000200  C 2.23161900 -1.03007400 0.00000300  H 2.86698300 -1.88180300 -0.00002100  H 1.18807200 2.03570600 0.00001000  H -1.18807500 2.03570200 0.00000800  Total Energy: -230.6497254  Enthalpy: -230.571449  Free energy: -230.608118  ZPE Correction: -230.578564  C -0.67412600 1.08437700 0.00000600  C 0.67410300 1.08437400 0.00002500  C 1.48464500 -0.07189600 0.00001000  C -1.48467400 -0.07188900 -0.00003300  C 2.20845000 -1.03647600 -0.00000200  H 2.83616300 -1.89605900 0.00004600  C -2.20846100 -1.03648200 -0.00005300  H -2.83577700 -1.89635500 0.00014100  H -1.19112000 2.04018200 0.00003300  H 1.19110200 2.04017700 0.00006600  Total Energy: -230.5554078  Enthalpy: -230.476323  Free energy: -230.512873  ZPE Correction: -230.483339  C -0.68090300 1.09051000 0.00004600  C 0.68097200 1.09052100 0.00012500  C 1.50532800 -0.07020800 0.00004200  C -1.50523600 -0.07023500 -0.00013700  C 2.24521500 -1.04506600 -0.00001700  H 2.88018700 -1.90247700 0.00014900  C -2.24518000 -1.04505000 -0.00022900  H -2.88140900 -1.90152900 0.00050100  H -1.19443100 2.05057400 0.00011400  H 1.19448300 2.05059300 0.00025000 |
|  |  |
| B3LYP/LANL2DZ  B3LYP/6-311++G**  PBE0/6-311++G**  PBE0/ LANL2DZ | Total Energy: -230.7944767  Enthalpy: -230.718352  Free energy: -230.751802  ZPE Correction: -230.724119  Frequency: -506.2126  C -1.23880800 0.70996900 0.00011300  C -1.24145800 -0.70609600 -0.00010700  C -0.00069600 -1.37131500 -0.00012500  C 0.00406600 1.37073400 0.00009800  C 1.22453400 -0.99342300 0.00005600  H 2.26497000 -1.27100800 0.00106400  C 1.22831100 0.98984000 -0.00004900  H 2.26930300 1.26542500 -0.00102600  H -2.19273000 1.22916600 0.00022700  H -2.19724000 -1.22183800 -0.00018500  Total Energy: -230.8967515  Enthalpy: -230.821318  Free energy: -230.854931  ZPE Correction: -230.827214  Frequency: -515.2016  C -1.21990700 0.70094900 -0.00000600  C -1.21870700 -0.70272100 -0.00000900  C 0.00478600 -1.35982200 0.00005400  C 0.00259200 1.36010900 -0.00006400  C 1.20547500 -0.96573300 0.00001300  H 2.24205800 -1.23777100 -0.00031900  C 1.20375300 0.96737300 0.00001000  H 2.24010300 1.24026800 0.00026300  H -2.17609600 1.20835400 0.00013500  H -2.17402000 -1.21177900 -0.00006800  Total Energy: -230.6002234  Enthalpy: -230.524242  Free energy: -230.557901  ZPE Correction: -230.530161  Frequency: -508.6891  C -1.21971800 0.69766700 0.00005700  C -1.21955100 -0.69792700 -0.00002900  C 0.00337900 -1.35403000 -0.00006700  C 0.00305600 1.35407300 0.00006500  C 1.20465000 -0.98294900 -0.00006800  H 2.24702300 -1.23639200 -0.00013900  C 1.20438900 0.98318100 0.00004400  H 2.24672000 1.23680400 0.00009500  H -2.17563500 1.20806500 0.00008300  H -2.17534100 -1.20856200 -0.00005400  Total Energy: -230.506076  Enthalpy: -230.429185  Free energy: -230.462633  ZPE Correction: -230.434948  Frequency: -504.9737  C -1.23844700 0.70379600 0.00005500  C -1.23839000 -0.70388400 -0.00003300  C 0.00148200 -1.36222700 -0.00008400  C 0.00137300 1.36224200 0.00008300  C 1.22424200 -1.00203800 -0.00007400  H 2.26813100 -1.26339600 -0.00011300  C 1.22415400 1.00211700 0.00005300  H 2.26802700 1.26353700 0.00007200  H -2.19136800 1.22281900 0.00009400  H -2.19126800 -1.22298600 -0.00005600 |
|  |  |
| B3LYP/6-311++G**  PBE0/6-311++G** | Total Energy: -230.9107256  Enthalpy: -230.833898  Free energy: -230.867284  ZPE Correction: -230.839704  C 1.19360900 -0.74057500 0.00004600  C 1.19339400 0.74077900 -0.00002200  C -0.00014900 1.35400000 -0.00002500  C 0.00016600 -1.35398700 -0.00000500  C -1.19358500 0.74041700 0.00005000  H -2.17489000 1.19640300 0.00006700  C -1.19341800 -0.74065100 -0.00003700  H -2.17467000 -1.19676000 -0.00007400  H 2.17489300 -1.19660500 0.00002000  H 2.17457100 1.19705900 -0.00006000  Total Energy: -230.6105784  Enthalpy: -230.533085  Free energy: -230.566545  ZPE Correction: -230.538921  C -1.19062500 0.73601600 -0.00014800  C -1.18960700 -0.73720400 0.00002700  C 0.00095300 -1.35106600 0.00008800  C -0.00077700 1.35123200 -0.00006900  C 1.19068400 -0.73649600 0.00004500  H 2.17487900 -1.19029200 0.00010000  C 1.18929000 0.73759600 0.00004800  H 2.17288000 1.19302100 0.00011200  H -2.17272200 -1.19347100 0.00006900  H -2.17454000 1.19026800 -0.00022500 |
|  |  |
| B3LYP/LANL2DZ  B3LYP/6-311+G**/def2-TZVP  PBE0/6-311+G**/def2-TZVP | Total Energy: -465.7008288  Enthalpy: -465.627689  Free energy: -465.675373  ZPE Correction-465.637815  C -4.33111700 -0.69825900 0.00004400  C -3.14148300 -1.39380500 0.00010000  C -1.82119600 -0.88388500 -0.00002400  C -4.47207700 0.72186100 -0.00016000  C -0.62675600 -0.52416700 -0.00014100  C -4.64746200 1.93928300 -0.00024800  H -4.75076000 2.99966900 0.00040700  H -5.25740300 -1.27474400 0.00016100  H -3.23054100 -2.48540800 0.00025600  Au 1.24695900 0.01111900 0.00000200  F 3.21883100 0.54621000 0.00017500  Total Energy: -466.144399  Enthalpy: -466.072002  Free energy: -466.119342  ZPE Correction: -466.082204  C -4.28419000 -0.67810700 0.00000700  C -3.11294200 -1.37590400 0.00000700  C -1.80211200 -0.88204100 -0.00000400  C -4.40918100 0.72976600 0.00000500  C -0.62132000 -0.54107000 -0.00001300  C -4.56729000 1.92929100 -0.00000200  H -4.64832700 2.98901200 -0.00001800  H -5.21405000 -1.24268100 0.00000100  H -3.21585900 -2.46316200 -0.00000600  Au 1.23135800 0.00572700 -0.00000100  F 3.17591000 0.57475300 0.00001100  Total Energy: -465.6952151  Enthalpy: -465.622271  Free energy: -465.669472  ZPE Correction: -465.632399  C -4.26561200 -0.67547700 -0.00001700  C -3.09995400 -1.37557400 -0.00007100  C -1.79166700 -0.88178100 -0.00003900  C -4.37553300 0.73094800 0.00007900  C -0.61118500 -0.53830200 -0.00001200  C -4.51736200 1.93235300 0.00015300  H -4.58616800 2.99424400 0.00020100  H -5.20003000 -1.23293300 -0.00005000  H -3.20343600 -2.46309300 -0.00014100  Au 1.22299700 0.00528600 -0.00000700  F 3.14896800 0.57013000 -0.00000500 |
| B3LYP/LANL2DZ  B3LYP/6-311+G**/def2-TZVP  PBE0/6-311+G**/def2-TZVP | Total Energy: -491.9944586  Enthalpy: -491.800411  Free energy: -491.861297  ZPE Correction: -491.816425  C -5.26390100 -0.36669400 0.00014500  C -4.20672100 -1.23941000 0.00007400  C -2.82388100 -0.90067900 0.00005000  C -5.16916900 1.05825200 0.00022600  C -1.60774600 -0.65277100 0.00002400  C -5.13958100 2.28547900 0.00031400  H -5.09498300 3.35072200 0.00033000  H -6.27109000 -0.78382800 0.00015600  H -4.44248100 -2.30561700 0.00003100  Au 0.32372900 -0.23027200 -0.00003500  P 2.69908400 0.28738400 -0.00005900  C 3.29160800 1.28832700 1.47886600  H 4.36933800 1.47729100 1.41360500  H 2.75544800 2.24166900 1.51031800  H 3.07274400 0.73921400 2.39968100  C 3.29169100 1.28772700 -1.47935500  H 4.36933000 1.47712900 -1.41386700  H 3.07332300 0.73801100 -2.39993000  H 2.75518000 2.24084700 -1.51151400  C 3.83037200 -1.21622900 0.00028400  H 3.62603000 -1.82371800 -0.88664300  H 4.88358600 -0.91220100 -0.00026400  H 3.62670300 -1.82279100 0.88800300  Total Energy: -827.3524894  Enthalpy: -827.159596  Free energy: -827.222688  ZPE Correction: -827.176325  C 5.21195600 -0.36671600 -0.00226600  C 4.16593500 -1.22884400 -0.00204600  C 2.79654700 -0.89377600 -0.00037300  C 5.11745400 1.04481100 -0.00069600  C 1.59715300 -0.65772700 0.00089300  C 5.08723800 2.25185600 0.00061700  H 5.03531900 3.31407100 0.00183400  H 6.21693400 -0.78117800 -0.00373600  H 4.40326300 -2.29154900 -0.00340300  Au -0.35004000 -0.22837700 0.00138100  P -2.62362300 0.28474900 -0.00077600  C -3.15141800 1.43772200 -1.33259100  H -4.22430000 1.64095800 -1.28165000  H -2.60541600 2.37832600 -1.24232800  H -2.91613800 1.00586700 -2.30681400  C -3.23654300 1.08968200 1.53473500  H -4.30536100 1.30963400 1.47008400  H -3.05800500 0.43504400 2.38947300  H -2.69069500 2.01920100 1.70489900  C -3.74606100 -1.15687100 -0.20944400  H -3.58115400 -1.87095100 0.59917500  H -4.79490100 -0.84889700 -0.20594700  H -3.52560200 -1.66082100 -1.15198900  Total Energy: -826.7010661  Enthalpy: -826.507118  Free energy: -826.570352  ZPE Correction: -826.523758  C -5.18402300 -0.35891600 0.00011600  C -4.14808900 -1.22772800 -0.00010400  C -2.78039200 -0.89600800 -0.00022500  C -5.06740300 1.04827400 0.00025200  C -1.58098500 -0.65910000 -0.00021500  C -5.01436900 2.25441800 0.00020400  H -4.94347300 3.31684300 0.00028800  H -6.19454900 -0.76056200 0.00016400  H -4.39024100 -2.28969600 -0.00022100  Au 0.35079800 -0.22928600 -0.00003500  P 2.59386900 0.28340500 -0.00001000  C 3.30097900 0.63833900 1.64572700  H 4.36595000 0.87735900 1.57685100  H 2.77369200 1.48009600 2.09899800  H 3.16969900 -0.22838500 2.29655500  C 3.04237800 1.75452100 -0.98453200  H 4.11781700 1.94863500 -0.94138000  H 2.74701700 1.60490100 -2.02491500  H 2.50553000 2.62688300 -0.60660900  C 3.68556700 -1.02283300 -0.66096400  H 3.40443300 -1.25075400 -1.69105300  H 4.73447400 -0.71427000 -0.63555900  H 3.56659200 -1.93431300 -0.07179800 |
|  | Total Energy: -442.1668092  Enthalpy: -442.068657  Free energy: -442.120464  ZPE Correction: -442.080515  C 4.33271700 -0.60773100 -0.00009900  C 3.21203200 -1.39638500 0.00003200  C 1.86032600 -0.94824100 0.00007800  C 4.34721400 0.82031300 -0.00007200  C 0.67226600 -0.60098800 0.00008200  C 4.41014200 2.04614300 0.00003400  H 4.44714600 3.11175400 0.00008300  H 5.30467500 -1.10120600 -0.00011800  H 3.36177900 -2.47767400 0.00000600  Au -1.17259400 -0.02546300 0.00000300  O -3.24793200 0.62809100 0.00002100  H -3.50539300 1.56548900 0.00007100  H -3.99800400 0.00982400 -0.00074500  Total Energy: -442.6064256  Enthalpy: -442.508448  Free energy: -442.558999  ZPE Correction: -442.520044  C -4.29915300 -0.59645600 0.00978800  C -3.18918000 -1.37316600 0.00699300  C -1.85116000 -0.92679400 0.00211700  C -4.31537200 0.81820200 0.00904300  C -0.67830600 -0.59575600 -0.00278300  C -4.37877400 2.02377200 0.00875100  H -4.41133600 3.08684200 0.00776100  H -5.26828600 -1.08878000 0.01254100  H -3.33816400 -2.45142100 0.00796300  Au 1.16412800 -0.03061300 -0.00846500  O 3.26343000 0.61508400 -0.01963600  H 3.40961000 1.56349000 -0.11306000  H 3.80631600 0.28879600 0.70718700  Total Energy: -442.1726697  Enthalpy: -442.073822  Free energy: -442.124271  ZPE Correction: -442.085337  C 4.28050100 -0.59153100 0.01014400  C 3.17818100 -1.37325300 0.00743700  C 1.84251100 -0.92819500 0.00231800  C 4.27758800 0.82055800 0.00911800  C 0.67029500 -0.59388500 -0.00227200  C 4.32123900 2.02692100 0.00862200  H 4.33740800 3.09165000 0.00746300  H 5.25508300 -1.07375600 0.01289000  H 3.33020300 -2.45148900 0.00843100  Au -1.15679900 -0.03079100 -0.00866300  O -3.22760900 0.60878400 -0.01981600  H -3.36843900 1.55571100 -0.09940300  H -3.76813500 0.27641500 0.70135400 |
| B3LYP/LANL2DZ  B3LYP/6-311+G**/def2-TZVP  PBE0/6-311+G**/def2-TZVP |  |
| B3LYP/LANL2DZ  B3LYP/6-311+G**/def2-TZVP  PBE0/6-311+G**/def2-TZVP | Total Energy: -465.6489208  Enthalpy: -465.577989  Free energy: -465.621719  ZPE Correction: -465.586783  Frequency: -446.9719  C -4.02087000 0.13562000 0.00069900  C -3.37110000 -1.13195600 0.00041200  C -1.97326900 -1.15147700 0.00014500  C -3.22035900 1.29838000 0.00067100  C -1.00343200 -0.28584500 0.00006100  C -1.91888500 1.39417300 0.00042100  H -1.12147800 2.11770200 0.00035000  H -5.10887200 0.15583300 0.00091600  H -3.98333800 -2.03161500 0.00043200  Au 0.96548700 -0.04123500 -0.00017200  F 2.99863700 0.16247900 -0.00028500  Total Energy: -466.0899287  Enthalpy: -466.019605  Free energy: -466.063279  ZPE Correction: -466.028480  Frequency: -441.2668  C -3.98234100 0.11134800 0.00064000  C -3.32201200 -1.13568700 0.00040100  C -1.94311500 -1.12774700 0.00016800  C -3.21690700 1.27696300 0.00063200  C -1.00055700 -0.25973700 0.00010400  C -1.92863500 1.35686800 0.00042200  H -1.15464900 2.09896100 0.00037400  H -5.06808000 0.10467800 0.00081900  H -3.92092500 -2.04067900 0.00040500  Au 0.95810400 -0.03176400 -0.00015400  F 2.97943000 0.11270800 -0.00040500  Total Energy: -465.6471657  Enthalpy: -465.576241  Free energy: -465.619899  ZPE Correction: -465.585104  Frequency: -437.8628  C -3.97217400 0.12623800 0.00063000  C -3.32358200 -1.11744200 0.00039800  C -1.94608800 -1.12037900 0.00017600  C -3.19682900 1.28475600 0.00062500  C -0.97383300 -0.29466200 0.00010800  C -1.91493500 1.35646500 0.00042600  H -1.11187300 2.06839200 0.00037200  H -5.05875100 0.12871600 0.00080500  H -3.92655500 -2.02059400 0.00040100  Au 0.95556700 -0.03708400 -0.00015200  F 2.95244500 0.14924900 -0.00041900 |
| B3LYP/LANL2DZ  B3LYP/6-311+G**/def2-TZVP  PBE0/6-311+G**/def2-TZVP | Total Energy: -491.9404287  Enthalpy: -491.747697  Free energy: -491.808300  ZPE Correction: -491.763355  Frequency: -468.7140  C 4.88404400 0.18215600 0.00126000  C 4.25840400 -1.09320500 0.00153900  C 2.85671300 -1.13138500 0.00073300  C 4.06654100 1.32946200 0.00020200  C 1.89891000 -0.26097700 -0.00026900  C 2.78044300 1.47311500 -0.00062600  H 1.98661600 2.19923700 -0.00146100  H 5.97017500 0.22236700 0.00188200  H 4.88643200 -1.98035200 0.00237900  Au -0.10568800 -0.08779200 -0.00104300  P -2.53447500 0.05504600 -0.00001100  C -3.37856800 -1.07680400 1.24313300  H -3.04376000 -0.82012000 2.25276300  H -4.46831200 -0.97638400 1.18300400  H -3.09616100 -2.11416800 1.03977800  C -3.34917400 -0.39002500 -1.63645300  H -4.43994500 -0.31097800 -1.56286600  H -2.98599700 0.28480300 -2.41747000  H -3.07557800 -1.41336900 -1.91072700  C -3.23156800 1.75631700 0.39980000  H -4.32747400 1.73810200 0.39315700  H -2.87857100 2.06981800 1.38688400  H -2.87544100 2.47897600 -0.34070800  Total Energy: -827.2964524  Enthalpy: -827.105719  Free energy: -827.165378  ZPE Correction: -827.121190  Frequency: -467.8353  C -4.83716700 0.13940200 0.00059600  C -4.19447100 -1.11314400 0.00032700  C -2.81092700 -1.11796900 -0.00017900  C -4.06167100 1.29441300 0.00028100  C -1.88854100 -0.23715400 -0.00051700  C -2.79050300 1.43098700 -0.00015600  H -2.01998600 2.17570100 -0.00034400  H -5.92158200 0.14770900 0.00091300  H -4.80713100 -2.00758200 0.00051300  Au 0.13536900 -0.06614800 0.00015900  P 2.46311700 0.04140600 0.00066700  C 3.28840300 -1.54036800 -0.44151300  H 2.97378200 -1.85310900 -1.43852500  H 4.37667200 -1.43904500 -0.42569200  H 2.99228000 -2.31905700 0.26339400  C 3.22098200 0.49225600 1.61368700  H 4.31182800 0.51623700 1.54758700  H 2.86131000 1.47349500 1.92785700  H 2.92519500 -0.23263900 2.37389400  C 3.18823000 1.25500000 -1.17475900  H 4.28055600 1.24341000 -1.13424600  H 2.86643500 1.02027600 -2.19073600  H 2.83376100 2.25867200 -0.93378200  Total Energy: -826.6509788  Enthalpy: -826.459216  Free energy: -826.519347  ZPE Correction: -826.474697  Frequency: -466.4242  C -4.81448100 0.17125200 0.00032500  C -4.19494700 -1.08405500 0.00025000  C -2.81316800 -1.11025300 0.00001100  C -4.01999200 1.31212100 0.00016000  C -1.85998800 -0.27157200 -0.00016900  C -2.75501600 1.44103600 -0.00006500  H -1.95474800 2.15484800 -0.00020600  H -5.89939200 0.19830000 0.00049700  H -4.82045600 -1.97046400 0.00037000  Au 0.14097400 -0.08167700 -0.00017500  P 2.43692300 0.05418900 0.00014100  C 3.16058800 0.91349500 1.43921300  H 2.85946400 0.40706300 2.35835500  H 4.25254000 0.93353300 1.38199800  H 2.78619100 1.93816300 1.48349600  C 3.16070000 0.92297900 -1.43316800  H 4.25262600 0.94312100 -1.37548700  H 2.86007400 0.42234900 -2.35564800  H 2.78590800 1.94776100 -1.47099500  C 3.27920700 -1.56498100 -0.00517800  H 4.36619400 -1.44550700 -0.00460800  H 2.98155700 -2.13784300 0.87528900  H 2.98181900 -2.13183300 -0.88961300 |
| B3LYP/LANL2DZ  B3LYP/6-311+G**/def2-TZVP  PBE0/6-311+G**/def2-TZVP | Total Energy: -442.1122888  Enthalpy: -442.016563  Free energy: -442.064722  ZPE Correction: -442.027165  Frequency: -476.1244  C 4.03084100 0.12140800 -0.00016800  C 3.37754500 -1.13999000 0.00009200  C 1.97567800 -1.15930600 0.00024800  C 3.23431300 1.28149200 -0.00027100  C 1.04115000 -0.26196300 0.00022500  C 1.95294700 1.45507200 -0.00016600  H 1.17510200 2.19829800 -0.00027700  H 5.11722300 0.13912300 -0.00023100  H 3.98838000 -2.03885700 0.00021700  Au -0.91214200 -0.04543700 0.00004000  O -3.08381100 0.15277800 -0.00030500  H -3.61318900 0.13932500 -0.81593000  H -3.61268600 0.14912100 0.81574800  Total Energy: -442.5496885  Enthalpy: -442.454082  Free energy: -442.501617  ZPE Correction: -442.464499  Frequency: -479.6112  C -3.99518000 0.08193700 -0.00001200  C -3.32554100 -1.15620500 0.00017600  C -1.94187200 -1.14874000 0.00023800  C -3.23758200 1.24596200 -0.00015000  C -1.04597300 -0.23754100 0.00013000  C -1.97333900 1.41983500 -0.00014600  H -1.21911200 2.18073600 -0.00028200  H -5.07902600 0.06944900 -0.00001400  H -3.92345800 -2.06053600 0.00030700  Au 0.90410300 -0.02623900 -0.00003400  O 3.10295000 0.13456100 -0.00033500  H 3.54521000 -0.20740600 0.78530300  H 3.54555800 -0.21735400 -0.78137100  Total Energy: -442.1216658  Enthalpy: -442.025102  Free energy: -442.072262  ZPE Correction: -442.035447  Frequency: -477.0125  C -3.98035200 0.10002600 0.00002100  C -3.32778600 -1.13838700 0.00005700  C -1.94564600 -1.14542300 0.00003800  C -3.20942500 1.25405100 -0.00001500  C -1.02423600 -0.26838300 0.00000300  C -1.95115600 1.42505400 -0.00002500  H -1.17206900 2.16172200 -0.00006100  H -5.06509800 0.10090000 0.00009600  H -3.93438200 -2.03786000 0.00013700  Au 0.90208700 -0.03228100 0.00000000  O 3.06492100 0.16453200 0.00007200  H 3.50952600 -0.17770800 0.77995200  H 3.50939500 -0.17470900 -0.78119100 |
|  |  |
| B3LYP/LANL2DZ | Total Energy: -465.6571672  Enthalpy: -465.584970  Free energy: -465.628761  ZPE Correction: -465.593783  C -1.08480500 -0.02279900 -0.00022800  C -1.85408800 1.29047500 -0.00007400  C -3.22796300 1.23780000 0.00021300  C -1.86059100 -1.12189800 -0.00008600  C -3.94793100 0.05964200 0.00014000  H -5.03431700 -0.00957900 0.00043000  C -3.22550200 -1.22649200 -0.00009800  H -3.81637000 -2.13899400 0.00010500  H -1.24826000 2.18855300 0.00003400  Au 0.94095800 -0.01151500 -0.00002900  F 2.99650500 -0.04785000 0.00027800 |
| B3LYP/LANL2DZ | Total Energy: -491.9519871  Enthalpy: -491.757956  Free energy: -491.819301  ZPE Correction: -491.773585  C 1.96854000 -0.01245400 0.00002700  C 2.75868400 -1.29749300 0.00003700  C 4.12263400 -1.16766700 0.00006700  C 2.67118200 1.13778000 0.00009800  C 4.80404400 0.02517800 0.00011500  H 5.88591800 0.13316900 0.00013900  C 4.03298300 1.28505600 0.00016300  H 4.59849300 2.21262600 0.00021600  H 2.19851700 -2.22444500 -0.00000300  Au -0.08085600 -0.02853100 -0.00005700  P -2.51956800 0.03633800 0.00001000  C -3.25003500 1.77040200 -0.00015400  H -4.34543400 1.73028900 -0.00015900  H -2.90471800 2.30986900 0.88698700  H -2.90470300 2.30970400 -0.88738900  C -3.33770700 -0.78965200 1.47977200  H -4.42909600 -0.71004800 1.41472000  H -3.05036000 -1.84498500 1.51234500  H -2.99132800 -0.30911200 2.39978700  C -3.33781600 -0.78997000 -1.47951500  H -4.42920700 -0.71052700 -1.41430100  H -2.99165800 -0.30949300 -2.39964600  H -3.05030900 -1.84526200 -1.51200000 |
| B3LYP/LANL2DZ | Total Energy: -442.1241692  Enthalpy: -442.027225  Free energy: -442.074973  ZPE Correction: -442.037751  C -1.11691300 -0.00109900 -0.00000100  C -1.88012700 1.29870800 -0.00021400  C -3.24528500 1.20648700 -0.00016200  C -1.85239800 -1.13851000 0.00018600  C -3.96276900 0.03932000 0.00002600  H -5.04598300 -0.04706900 0.00003900  C -3.21534900 -1.23929800 0.00021900  H -3.80851900 -2.14922700 0.00037700  H -1.29618800 2.21075100 -0.00037500  Au 0.88471800 -0.00127600 -0.00001700  O 3.08475200 -0.07488100 0.00002500  H 3.60860500 -0.14953000 0.81611300  H 3.60838400 -0.15874600 -0.81530900 |
|  |  |
| B3LYP/LANL2DZ | Total Energy: -485.4268199  Enthalpy: -485.263320  Free energy: -485.310129  ZPE Correction: -485.274824  C 2.10341800 1.17079200 0.01744100  C 0.66705200 0.89492600 0.01222100  C 0.22666000 -0.42367000 -0.02478100  C 2.88655300 0.05735800 0.01516700  C 1.09094500 -1.48994700 -0.03227300  H 0.79165200 -2.53441800 -0.02994200  C 2.55004000 -1.25545000 -0.02500400  H 3.20667200 -2.11828700 -0.02428600  C -0.41571400 1.95196800 0.04857700  H -0.61264800 2.21841100 1.09696000  H -0.14277500 2.87367100 -0.48287200  C -1.67052600 1.24053700 -0.54831800  H -2.60116600 1.74444900 -0.25491900  H -1.60986000 1.24698900 -1.64830100  B -1.63355200 -0.30476200 -0.00282100  H 2.43140700 2.20582600 0.01517000  O -2.20101000 -1.36669800 -0.80067700  H -2.13430600 -1.28161600 -1.76892000  O -1.97689500 -0.43530100 1.38741100  H -2.36855500 -1.29430300 1.63916000 |
| B3LYP/LANL2DZ | Total Energy: -334.88842  Enthalpy: -334.739299  Free energy: -334.779397  ZPE Correction: -334.747891  C 0.94901700 1.54697000 -0.11152600  C -0.17513800 0.65856000 0.00521300  C -0.03435700 -0.77628200 0.03707700  C 2.10971500 0.84398800 0.08004500  C 1.26384400 -1.34077900 0.04293200  H 1.41298800 -2.41672700 0.08954600  C 2.40436300 -0.49603100 -0.09110500  H 3.39805800 -0.89008500 -0.27974500  C -1.61104400 1.12079700 0.20051600  H -1.80575400 1.30060100 1.26983100  H -1.80489300 2.06353000 -0.33105900  C -2.44859200 -0.11473400 -0.27616500  H -3.43502600 -0.14481900 0.20064600  H -2.59945300 -0.03392300 -1.36493100  B -1.49552900 -1.40721500 0.06685000  H -1.69216400 -2.48265100 -0.45509900  H -1.56307300 -1.63913200 1.30122700  H 0.82011100 2.62434500 -0.08658100 |
| B3LYP/LANL2DZ | Total Energy: -533.5231696  Enthalpy: -533.384024  Free energy: -533.429731  ZPE Correction: -533.394762  C 2.06677900 1.23395900 -0.01985100  C 0.63396300 0.91908500 -0.02468100  C 0.18144100 -0.40941200 -0.02008900  C 2.76626700 0.08216900 0.00419500  C 1.07447100 -1.46708800 0.00759000  H 0.77291900 -2.51161400 -0.02686500  C 2.52853000 -1.24323300 -0.01297900  H 3.21621500 -2.07340500 -0.11057400  C -0.45869000 1.96983600 0.05842300  H -0.59350200 2.26516900 1.10923400  H -0.22361300 2.87601800 -0.51528000  C -1.74059000 1.20734900 -0.44466300  H -2.65019200 1.64565500 -0.01550000  H -1.80921600 1.27693800 -1.53974000  B -1.50537400 -0.35061400 -0.02086700  H 2.40929900 2.25833300 0.06388700  F -2.06474800 -1.32994300 -0.91712000  F -1.92503700 -0.64116800 1.34506500 |
| B3LYP/LANL2DZ | Total Energy: -363.6869794  Enthalpy: -363.548842  Free energy: -363.596394  ZPE Correction: -363.560093  C 2.72679000 0.81095400 -0.27681900  C 1.26732500 0.82933200 -0.28581100  C 0.49067500 -0.33148800 -0.03364400  C 3.09906200 -0.42405500 0.11774500  C 1.10673700 -1.54582500 0.28323900  H 0.53915400 -2.45457300 0.46514100  C 2.55073100 -1.65564200 0.27580600  H 3.05895000 -2.61103300 0.32912800  C 0.44689200 2.10452800 -0.43879200  H 0.34543700 2.59334500 0.54063500  H 0.91150100 2.81742500 -1.13154800  C -0.95356100 1.55706900 -0.89427400  H -1.75833200 2.24769700 -0.62085300  H -0.95940500 1.43659000 -1.98655600  B -1.06424200 0.10695300 -0.18341000  H 3.30583700 1.72195200 -0.36872000  Cl -2.16729900 -1.18261600 -1.05378600  Cl -1.62857300 0.33818100 1.71291000 |
| B3LYP/LANL2DZ | Total Energy: -360.1143301  Enthalpy: -359.976425  Free energy: -360.026625  ZPE Correction: -359.988139  C -3.35777600 -0.08753100 0.65487600  C -1.93225700 0.11309900 0.81815300  C -0.98616200 -0.38514800 -0.12376200  C -3.56107700 -0.63937300 -0.56710900  C -1.41401900 -1.05408900 -1.28112800  H -0.70931200 -1.43728500 -2.01432800  C -2.81389500 -1.30662000 -1.49470100  H -3.17616300 -1.94415500 -2.29284200  C -1.30980300 0.94188500 1.93654600  H -1.32297500 2.00394600 1.65363700  H -1.85622100 0.83474500 2.88205800  C 0.17055600 0.42640100 1.97566100  H 0.85613600 1.18959000 2.35689200  H 0.23296000 -0.44950400 2.63711600  B 0.47150300 -0.02982700 0.45374400  H -4.07758300 0.34217700 1.34221400  Br 1.93665900 -1.45057200 0.15335900  Br 0.88969100 1.78079700 -0.73463500 |
| B3LYP/LANL2DZ | Total Energy: -519.3870071  Enthalpy: -519.232834  Free energy: -519.282838  ZPE Correction: -519.245342  C 2.60125900 0.78555900 -0.28239100  C 1.13857600 0.79763300 -0.30729400  C 0.35686100 -0.34060500 0.01539700  C 2.94500000 -0.44088600 0.14604700  C 0.97321400 -1.53460500 0.40274400  H 0.41156500 -2.43887400 0.62600600  C 2.42199000 -1.65821500 0.40609100  H 2.92838800 -2.61037800 0.50581600  C 0.32699800 2.06504300 -0.54989000  H 0.22186100 2.62044000 0.39389200  H 0.80385200 2.72822700 -1.28254200  C -1.06427500 1.49081400 -0.98421500  H -1.86032000 2.21873900 -0.79339100  H -1.04590900 1.27423200 -2.06064700  B -1.22868200 0.08370300 -0.12902500  H 3.18565700 1.68966200 -0.39983000  C -1.75014600 0.36885700 1.35088700  C -2.09100100 -1.03571300 -0.83616400  N -2.70797600 -1.88318200 -1.38668800  N -2.09953100 0.61348900 2.45647900 |
|  |  |
| B3LYP/LANL2DZ | Total Energy: -680.5131999  Enthalpy: -680.329929  Free energy: -680.389014  ZPE Correction: -680.345563  C -1.38670200 2.22540900 -0.03061600  C -2.46723800 1.26607900 -0.05634000  C -2.25780500 -0.13122000 0.06967000  C -0.21358500 1.56205800 0.22652900  C -0.96971700 -0.64171600 0.26942000  H -0.76250900 -1.70706000 0.30949900  C 0.16242800 0.29333200 0.26121600  C -3.93998000 1.65192200 -0.12807900  H -4.31271800 1.82746000 0.89279300  H -4.10322800 2.57312400 -0.71090900  C -4.62761100 0.37023100 -0.69827600  H -5.70372800 0.34290100 -0.45892500  H -4.53304300 0.36280700 -1.79815900  B -3.78656600 -0.90150200 -0.05056800  H -1.57154300 3.29335900 -0.06438800  N -3.82489400 -2.14990700 -0.93168700  H -3.63665100 -3.05088300 -0.49690800  H -3.48422100 -2.09983300 -1.89057300  N -4.20742400 -1.15263400 1.40120400  H -5.19978600 -1.26536500 1.59670600  H -3.61905600 -1.71304200 2.01288800  Au 2.14042000 -0.07874400 0.00139000  F 4.13262600 -0.47710700 -0.22408300 |
| B3LYP/LANL2DZ | Total Energy: -595.6455013  Enthalpy: -595.462104  Free energy: -595.522057  ZPE Correction: -595.477844  C -1.64724700 2.22474500 -0.04330600  C -2.71684800 1.25632600 -0.08150500  C -2.49350500 -0.13864900 0.02916700  C -0.45472900 1.58938900 0.22132900  C -1.19976300 -0.64460000 0.21358600  H -0.99372900 -1.70925800 0.24849600  C -0.09875300 0.31302600 0.23026700  C -4.19287400 1.62405800 -0.15697500  H -4.56641400 1.85613900 0.85479900  H -4.36863000 2.52072400 -0.77370400  C -4.87855100 0.31961300 -0.68788600  H -5.95369600 0.29106800 -0.44102400  H -4.78894700 0.29235800 -1.78674200  B -4.00324100 -0.95892700 -0.05728000  H -1.84395100 3.29077700 -0.09215400  N -3.88133700 -2.24541900 -0.85092900  H -4.63003800 -2.92538700 -0.75899500  H -3.43865000 -2.24436500 -1.76489400  N -4.49410100 -1.28997400 1.34755900  H -4.90423900 -0.56783100 1.93275300  H -3.99830200 -2.00451700 1.87513900  Au 1.91275400 -0.00679700 0.02444300  Cl 4.30099700 -0.46963400 -0.16251400 |
| B3LYP/LANL2DZ | Total Energy: -593.868495  Enthalpy: -593.685200  Free energy: -593.746636  ZPE Correction: -593.701188  C -2.14227300 2.22590700 -0.05189200  C -3.18687100 1.23029200 -0.08489500  C -2.92594500 -0.15808200 0.01794800  C -0.92869500 1.62173900 0.19099300  C -1.61794700 -0.63168700 0.18709000  H -1.38393500 -1.69050900 0.22168000  C -0.54383200 0.35538100 0.20220400  C -4.67256000 1.55887600 -0.14972300  H -5.04358000 1.78172700 0.86508400  H -4.87738200 2.45030600 -0.76503200  C -5.32866500 0.23753000 -0.67573000  H -6.40034700 0.17992000 -0.41907600  H -5.24875800 0.21362400 -1.77535700  B -4.41557100 -1.01964200 -0.05599500  H -2.36630600 3.28661400 -0.09934100  N -4.26662500 -2.29819700 -0.85680300  H -4.98928000 -3.00403500 -0.75422200  H -3.83398500 -2.28299900 -1.77521500  N -4.88435500 -1.36952000 1.35104900  H -5.30564700 -0.66051800 1.94425200  H -4.36590100 -2.07410000 1.87020600  Au 1.48586700 0.08258400 0.03506200  Br 4.01848700 -0.35976200 -0.08795900 |
| B3LYP/LANL2DZ | Total Energy: -656.9651777  Enthalpy: -656.757066  Free energy: -656.819822  ZPE Correction: -656.774511  C -1.53403200 2.26256400 -0.01117300  C -2.56792700 1.28844500 -0.03117800  C -2.29161300 -0.11115700 0.06345800  C -0.32280700 1.63328700 0.18686400  C -0.95595000 -0.58317500 0.13759400  H -0.77254300 -1.65664800 0.16508300  C 0.12683900 0.35057600 0.12002000  C -4.07903300 1.60111300 -0.13215500  H -4.47578600 1.84295000 0.86396200  H -4.23250700 2.48131800 -0.77483700  C -4.69136100 0.30894200 -0.66343000  H -5.70985600 0.10472100 -0.31952300  H -4.63301400 0.25369100 -1.75862600  B -3.64537200 -0.98758300 0.01490000  H -1.71622900 3.33336200 -0.03777700  N -3.62620900 -2.18323400 -0.90475000  H -3.90690600 -3.09483400 -0.56026800  H -3.60555300 -2.09620900 -1.91531700  N -4.14122400 -1.31897500 1.39781700  H -5.09180100 -1.62911100 1.56986900  H -3.61263200 -1.09601700 2.23224400  Au 2.08535100 -0.05267900 0.00455600  O 4.25233600 -0.49297500 -0.13415900  H 4.82789700 -0.54823500 0.64934000  H 4.66169400 -0.83970600 -0.94679800 |
| B3LYP/LANL2DZ | Total Energy: -591.8821303  Enthalpy: -591.676876  Free energy: -591.740513  ZPE Correction: -591.693679  C -1.92552300 2.15498700 0.45940400  C -2.80423300 1.12158100 -0.19155000  C -2.48607100 -0.30199500 0.26340100  C -0.67300500 1.71159800 0.71520200  C -1.24042100 -0.58874700 0.78089700  H -0.99185700 -1.59672900 1.10741700  C -0.18467800 0.45331700 0.66679600  C -4.26078900 1.55487900 -0.49188600  H -5.05364100 0.84104600 -0.29785800  H -4.50686000 2.58743300 -0.25780900  C -3.33892000 1.28287900 -1.65885800  H -3.48928400 0.37240900 -2.23156600  H -2.93519800 2.11908000 -2.22024000  B -3.52819800 -1.48693000 0.09845900  H -2.36614500 3.05019000 0.89596700  N -3.21794900 -2.54929800 -0.80244000  H -3.79665000 -3.36860000 -0.94346400  H -2.35824600 -2.53989200 -1.33541900  N -4.74003100 -1.48369300 0.85523700  H -5.43344200 -2.22105700 0.81944100  H -4.91765500 -0.77260600 1.55228200  Au 1.69957700 0.00498600 0.10943600  S 4.20221900 -0.51196100 -0.53651000  H 4.84322000 0.33011500 0.34581500  H 4.33239100 0.33068500 -1.61811900 |
| B3LYP/LANL2DZ | Total Energy: -706.8184893  Enthalpy: -706.510930  Free energy: -706.589261  ZPE Correction: -706.533357  C 2.39503100 -2.00233900 -0.33708600  C 3.36902600 -1.03148500 0.21925700  C 2.85035400 0.15482900 0.68596400  C 1.07769500 -1.63096400 -0.33756000  C 1.53635000 0.52247400 0.69146100  H 1.12801000 1.45488300 1.06117700  C 0.55387400 -0.47462700 0.11968200  C 4.84180900 -1.43072100 0.21546900  H 5.22788100 -1.36785800 1.24333600  H 4.92594300 -2.48306600 -0.08942100  C 5.71599100 -0.54525300 -0.72255200  H 6.70645400 -1.02483800 -0.81047400  H 5.27655500 -0.56634900 -1.73132600  B 5.94293900 0.96193400 -0.24296100  H 2.80744700 -2.94102800 -0.70022100  N 5.66887700 2.03651600 -1.14362400  H 5.79941200 3.01456300 -0.91658300  H 5.22850800 1.86917100 -2.03816300  N 6.48931700 1.21323800 1.05480500  H 6.66338000 0.46450400 1.71148800  H 6.63485700 2.13997200 1.43478100  Au -1.45245500 -0.07757600 0.04834800  P -3.85937600 0.31288800 -0.07481200  C -4.38468000 1.70307500 -1.23019900  H -5.47486400 1.81829900 -1.23019000  H -3.91913000 2.63908700 -0.90685300  H -4.04119400 1.47984900 -2.24488200  C -4.85813200 -1.16411000 -0.67783100  H -4.70301600 -2.00762200 0.00170200  H -5.92625500 -0.92133000 -0.72125000  H -4.50918000 -1.45542400 -1.67316900  C -4.68178300 0.76577500 1.55671300  H -5.75982900 0.91096100 1.42108800  H -4.50976300 -0.03336300 2.28408800  H -4.23587500 1.68690100 1.94417100 |
|  | Total Energy: -754.945555  Enthalpy: -754.671601  Free energy: -754.747678  ZPE Correction: -754.693190  C 2.37504200 -2.02793700 -0.22877800  C 3.35772900 -1.01644900 0.23353600  C 2.84433900 0.20421300 0.60610600  C 1.05776600 -1.65763000 -0.24267200  C 1.53167600 0.57397000 0.59891800  H 1.12963600 1.53512100 0.89453000  C 0.54209400 -0.46482500 0.12362800  C 4.82942900 -1.41866800 0.25102900  H 5.20861700 -1.32321600 1.27925500  H 4.91262600 -2.48074700 -0.01775000  C 5.71120400 -0.56521700 -0.70834900  H 6.69933700 -1.05228200 -0.77873200  H 5.27579700 -0.61209900 -1.71789400  B 5.94535000 0.95368200 -0.27049400  H 2.78298000 -2.99347600 -0.51897500  N 5.70048600 2.00235300 -1.20913100  H 5.84084900 2.98535900 -1.01124400  H 5.27581200 1.81223200 -2.10670400  N 6.47004400 1.23946900 1.02901200  H 6.62762000 0.50997600 1.71099500  H 6.62222900 2.17601000 1.38134700  Au -1.46022300 -0.07354200 0.04337500  P -3.87139200 0.30573400 -0.06590900  N -4.71503700 -1.17702200 -0.34030900  H -4.24220800 -2.05787600 -0.18234400  H -5.71479400 -1.20902100 -0.50594700  N -4.67559600 1.26622000 -1.28567600  H -4.75551400 0.88387900 -2.22131600  H -4.59962800 2.27563800 -1.22878800  N -4.43697900 1.17380100 1.31832600  H -5.39677600 1.48997200 1.40265800  H -3.86091300 1.21747400 2.14931200 |
|  |  |
| BH2_AuF_Product | Total Energy: -569.7613376  Enthalpy: -569.616387  Free energy: -569.668484  ZPE Correction: -569.628345  C -2.09818200 -1.16175800 -0.06555100  C -3.37566400 -0.67553900 -0.11086000  C -3.39730400 0.82604300 -0.09835400  C -1.19227400 0.04202700 -0.03664200  C -2.09620200 1.26151600 -0.05223500  H -1.70668500 2.27039600 -0.02259200  C 0.12191800 0.02796700 -0.03070000  C -4.76868100 -1.25452700 -0.13171400  H -5.05116300 -1.45685500 -1.17890700  H -4.84788100 -2.20792800 0.41457000  C -5.67008400 -0.09268300 0.42048300  H -6.71252700 -0.20560100 0.08349700  H -5.67419600 -0.13932200 1.52323600  B -4.93651800 1.31942900 -0.10466700  H -1.73697300 -2.18225300 -0.05188600  Au 2.01886600 0.00140800 0.00696200  H -5.25332200 1.55697100 -1.27448700  H -5.17529200 2.31693600 0.56249100  F 4.02322600 -0.05544500 0.06120600 |
| BH2_AuCl_Product | Total Energy: -484.8849207  Enthalpy: -484.740454  Free energy: -484.793544  ZPE Correction: -484.752770  C -2.36782600 -1.17615600 -0.04181100  C -3.64039100 -0.67903100 -0.10411200  C -3.64943400 0.82301600 -0.09428600  C -1.45223900 0.01947800 -0.00669900  C -2.34666800 1.24952100 -0.03237000  H -1.94743100 2.25429600 -0.00163300  C -0.13883700 0.00692300 -0.00291300  C -5.03765200 -1.24580200 -0.14133400  H -5.30832100 -1.44712700 -1.19177900  H -5.13184100 -2.19763000 0.40501500  C -5.93572300 -0.07519500 0.39769600  H -6.97453100 -0.17904500 0.04733200  H -5.95449100 -0.11987500 1.50022800  B -5.18411500 1.32947500 -0.12075900  H -2.01473500 -2.19928300 -0.02343800  Au 1.77857200 0.00295700 0.00612600  H -5.48078000 1.56573700 -1.29593300  H -5.42293800 2.33113500 0.53904900  Cl 4.18006200 -0.02504300 0.01740900 |
| BH2_AuBr_Product | Total Energy: -483.1060553  Enthalpy: -482.961753  Free energy: -483.016158  ZPE Correction: -482.974300  C -2.82870800 -1.17755100 -0.03999100  C -4.10144400 -0.68101700 -0.10305700  C -4.11121800 0.82108500 -0.09322100  C -1.91365700 0.01853900 -0.00467000  C -2.80886900 1.24851400 -0.03057300  H -2.40994700 2.25337600 0.00018200  C -0.60005100 0.00785200 -0.00203500  C -5.49838700 -1.24840800 -0.14082000  H -5.76856300 -1.44970000 -1.19139000  H -5.59231900 -2.20033700 0.40535400  C -6.39714500 -0.07823900 0.39800600  H -7.43577400 -0.18248900 0.04729400  H -6.41624500 -0.12294000 1.50051000  B -5.64613500 1.32674300 -0.12029100  H -2.47502100 -2.20044900 -0.02154000  Au 1.32572400 0.00724100 0.00412400  Br 3.85660100 -0.01882100 0.01115300  H -5.94195500 1.56235200 -1.29580900  H -5.88582100 2.32850600 0.53888600 |
| BH2_AuH2O_Product | Total Energy: -546.2099932  Enthalpy: -546.041009  Free energy: -546.095206  ZPE Correction: -546.054477  C 2.14254900 1.79168000 0.06387300  C 3.04298100 0.66932400 0.01691100  C 2.61344500 -0.69528600 -0.17309500  C 0.90798500 1.33295200 -0.31619600  C 1.24581400 -0.96991800 -0.39042300  H 0.88584500 -1.98789200 -0.50368600  C 0.30689100 0.12370300 -0.30017800  C 4.55938400 0.80423900 0.03502300  H 4.93099500 0.96252400 -0.99038800  H 4.88234100 1.66409000 0.64086500  C 5.03161900 -0.60393000 0.53615600  H 6.06190400 -0.81210300 0.21976300  H 5.00802700 -0.61065000 1.63800700  B 3.91052500 -1.64662100 -0.08565300  H 2.49929000 2.81411800 0.11670100  H 3.76050600 -2.72413000 0.46069200  H 4.16538800 -1.88648000 -1.27793800  Au -1.66662000 -0.01941000 0.00018500  O -3.81439000 -0.22415900 0.31351000  H -4.48053100 0.12018800 -0.30650200  H -4.19229400 -0.69644600 1.07561700 |
| BH2_AuH2S_Product | Total Energy: -481.0752459  Enthalpy: -480.913163  Free energy: -480.969139  ZPE Correction: -480.926833  C 2.38654800 1.78869500 0.06924900  C 3.28745700 0.66591300 0.03653600  C 2.86059100 -0.69868100 -0.15514800  C 1.15584800 1.32844900 -0.32246700  C 1.49578000 -0.97604100 -0.38860900  H 1.13814800 -1.99424700 -0.50700600  C 0.55893200 0.12003500 -0.31371900  C 4.80344400 0.80119600 0.07706800  H 5.18949300 0.95864400 -0.94310700  H 5.11724300 1.66180500 0.68651200  C 5.26901900 -0.60620700 0.58681000  H 6.30353200 -0.81495900 0.28518200  H 5.23039500 -0.61096500 1.68824300  B 4.15703500 -1.64965000 -0.04807000  H 2.74283000 2.81109400 0.12573000  H 4.00168000 -2.72794600 0.49412700  H 4.42432700 -1.88736300 -1.23850100  Au -1.44011900 -0.01401900 -0.04528700  S -3.97484400 -0.22956300 0.26729700  H -4.38544100 1.00264400 -0.19052300  H -4.08620300 0.08986000 1.60232000 |
| BH2_AuPMe3_Product | Total Energy: -596.0399365  Enthalpy: -595.773749  Free energy: -595.841111  ZPE Correction: -595.792415  C 3.07941400 -1.77723500 -0.10866700  C 3.94431200 -0.62576300 -0.07294400  C 3.47979400 0.71565600 0.16692300  C 1.84120400 -1.35048500 0.29639900  C 2.11503900 0.94295000 0.43621200  H 1.72585000 1.94510800 0.58838700  C 1.19785700 -0.18308200 0.35026900  C 5.46296600 -0.70779300 -0.16489500  H 5.88325400 -0.87685400 0.84001000  H 5.78891900 -1.54109500 -0.80577700  C 5.86571200 0.72705000 -0.65091700  H 6.90635900 0.95630100 -0.38435600  H 5.78477400 0.76222800 -1.74981000  B 4.74860000 1.71945100 0.06037000  H 3.46117600 -2.78757000 -0.19823200  H 4.53254400 2.79306000 -0.47455000  H 5.05284200 1.94638400 1.23943400  Au -0.83794400 -0.06823800 0.10697000  P -3.25135100 0.12592100 -0.16100500  C -4.06494000 -1.35629400 -0.98420900  H -5.14683500 -1.20359900 -1.06996100  H -3.63789900 -1.49886400 -1.98135900  H -3.87064600 -2.25536800 -0.39175000  C -3.78980300 1.58884700 -1.21212000  H -4.88195600 1.62117900 -1.29717500  H -3.43309300 2.51689100 -0.75542400  H -3.34876900 1.50292900 -2.20963600  C -4.20414000 0.33942100 1.44570000  H -3.85439200 1.23943600 1.96013000  H -5.27836900 0.42746700 1.24735800  H -4.02339300 -0.52256100 2.09481800 |
| BH2_AuPOMe3_Product | Total Energy: -703.7626948  Enthalpy: -703.571141  Free energy: -703.637085  ZPE Correction: -703.588829  C 3.07608000 -1.76326700 -0.13989600  C 3.93550100 -0.60717400 -0.09010700  C 3.46094500 0.73053200 0.15712100  C 1.83784100 -1.35556200 0.27740200  C 2.09289800 0.94549800 0.42628800  H 1.70081200 1.94385500 0.59515400  C 1.18670100 -0.18102200 0.33192900  C 5.45403000 -0.68230300 -0.17207300  H 5.87010700 -0.85925300 0.83296900  H 5.78565300 -1.50814800 -0.81882300  C 5.85197000 0.75951500 -0.64239700  H 6.88559800 0.99693700 -0.35923900  H 5.78500500 0.80088900 -1.74172000  B 4.71884700 1.73693400 0.05779300  H 3.46650700 -2.76970500 -0.23754300  H 4.50704900 2.82375800 -0.44769800  H 5.00593800 1.94522200 1.24790600  Au -0.83233600 -0.08968600 0.12700600  P -3.25278500 0.11628700 -0.16088000  O -3.95699600 -1.31926300 -0.71530400  H -4.93357200 -1.42338300 -0.70913000  O -4.24974800 0.55046400 1.14589900  H -4.75227600 1.39053200 1.06416400  O -3.84175300 1.35176800 -1.20545600  H -3.35653700 1.57449000 -2.02600900 |
| BH2_AuPNH2_Product | Total Energy: -644.1656841  Enthalpy: -643.933199  Free energy: -643.998444  ZPE Correction: -643.951027  C 3.06784100 -1.77348400 -0.23799700  C 3.93515000 -0.62891900 -0.12259000  C 3.47144100 0.69474400 0.20281800  C 1.82962600 -1.37353300 0.19181300  C 2.10575700 0.90557800 0.48125500  H 1.71882700 1.89610600 0.70074600  C 1.18677400 -0.20916300 0.31908500  C 5.45381900 -0.70813300 -0.21229900  H 5.86886900 -0.94338000 0.78130500  H 5.78120100 -1.49818700 -0.90489800  C 5.86095700 0.75506800 -0.60094200  H 6.89989100 0.96522000 -0.31348900  H 5.78659200 0.86204700 -1.69555200  B 4.74091400 1.70009000 0.16656900  H 3.44826700 -2.77630800 -0.39352400  H 4.53055400 2.80900700 -0.29233300  H 5.03799100 1.84606300 1.36090000  Au -0.84483600 -0.07337200 0.09733400  P -3.26195800 0.14857000 -0.13742800  N -4.07980800 -0.46234200 1.25366700  H -3.57263000 -0.58995200 2.12039100  H -5.09069000 -0.53252100 1.29707500  N -4.19935700 -0.66865300 -1.35621700  H -4.40257400 -1.65452700 -1.23807700  H -4.15506300 -0.33401000 -2.31192700  N -3.68017100 1.77173500 -0.54287400  H -4.62873000 2.04356600 -0.77694600  H -3.00852800 2.51610400 -0.40337200 |
|  |  |
|  | Total Energy: -754.2890926  Enthalpy: -754.138839  Free energy: -754.198955  ZPE Correction: -754.154333  C 1.09756700 -1.69212700 -0.36745600  C 2.43126000 -1.40224200 -0.28468800  C 2.65157400 0.05723700 -0.06830400  C 0.37480200 -0.38736500 -0.18610100  C 1.44028200 0.68222100 -0.01447300  H 1.20692300 1.72907900 0.12280100  C -0.92392000 -0.19477800 -0.12836400  C 3.73575400 -2.15427600 -0.38220500  H 3.99149100 -2.55849500 0.61055600  H 3.69639800 -3.00405000 -1.07809000  C 4.77965200 -1.05354900 -0.77708800  H 5.80151000 -1.34501100 -0.50239900  H 4.75486000 -0.90518900 -1.86712300  B 4.25103800 0.34360800 -0.02958700  H 0.59788900 -2.63871500 -0.52374400  Au -2.78639000 0.08923600 0.02171000  C 4.70131900 1.67087000 -0.77933100  C 4.75934800 0.38244100 1.48863100  N 5.03959200 2.64579100 -1.36335800  N 5.14124900 0.36633400 2.61158200  F -4.74791100 0.37991600 0.21439700 |
|  | Total Energy: -669.4114838  Enthalpy: -669.261730  Free energy: -669.323813  ZPE Correction: -669.277693  C 1.37429600 -1.70151300 -0.35102500  C 2.70458100 -1.39629800 -0.27387900  C 2.90958800 0.06553400 -0.05328200  C 0.63743200 -0.40468100 -0.16354500  C 1.69265500 0.67747200 0.00906100  H 1.44688800 1.72087500 0.14978700  C -0.66255300 -0.22422400 -0.11319400  C 4.01685100 -2.13202300 -0.38513000  H 4.28671300 -2.53487400 0.60441300  H 3.98133000 -2.98067500 -1.08240600  C 5.04298300 -1.01717500 -0.78697600  H 6.07117500 -1.29744300 -0.52498700  H 5.00415900 -0.86431800 -1.87593300  B 4.50635400 0.36995500 -0.02655100  H 0.88378300 -2.65235500 -0.50966400  Au -2.55080400 0.04218900 0.00134000  C 4.93403100 1.70658400 -0.77191000  C 5.02631700 0.40378800 1.48739300  N 5.25521300 2.68889300 -1.35305400  N 5.41769100 0.38355400 2.60694100  Cl -4.90939400 0.35612900 0.17078700 |
|  | Total Energy: -667.632449  Enthalpy: -667.482866  Free energy: -667.546517  ZPE Correction: -667.499089  C 1.86147600 -1.69734500 -0.34115600  C 3.19005000 -1.38242400 -0.27854400  C 3.38666500 0.07699200 -0.03382600  C 1.11688900 -0.40896200 -0.12236800  C 2.16585900 0.67745500 0.05793000  H 1.91381100 1.71632500 0.21954800  C -0.18526100 -0.24285200 -0.07194500  C 4.50633400 -2.10420900 -0.42710400  H 4.79870400 -2.52165100 0.54986900  H 4.46518000 -2.94066300 -1.13864400  C 5.51431900 -0.97264100 -0.82843500  H 6.55010200 -1.24858600 -0.59309800  H 5.45155300 -0.79956100 -1.91324100  B 4.98112700 0.39457800 -0.03045800  H 1.37594700 -2.64862400 -0.51190200  Au -2.08869100 -0.01051600 -0.00176300  C 5.38339300 1.74935800 -0.75690000  C 5.53017800 0.40240900 1.47348000  N 5.68519000 2.74586100 -1.32415000  N 5.94417200 0.36327300 2.58438300  Br -4.59083800 0.25571400 0.08087500 |
|  | Total Energy: -730.6950291  Enthalpy: -730.520677  Free energy: -730.583907  ZPE Correction: -730.537683  C 1.23224700 -1.85874200 -0.35406100  C 2.54023000 -1.47216700 -0.23972400  C 2.65104400 0.01072900 -0.08982500  C 0.41519000 -0.61431600 -0.25215700  C 1.41746100 0.57233400 -0.10261900  H 1.09865000 1.60118200 -0.02620700  C -0.86510600 -0.38719300 -0.18537100  C 3.89402400 -2.12462900 -0.27027800  H 4.15385400 -2.44651200 0.75113200  H 3.93433600 -3.01615100 -0.90982900  C 4.85994500 -0.97038900 -0.70940600  H 5.89375800 -1.17134400 -0.40571900  H 4.84387800 -0.87561200 -1.80436400  B 4.23307100 0.42000900 -0.02881400  H 0.80411200 -2.84414800 -0.47565600  Au -2.70650700 0.06588200 0.00956900  O -4.76520900 0.53621200 0.26448800  H -5.36102700 0.75946700 -0.47295900  H -5.19070300 0.58911400 1.13902300  C 4.57468200 1.74891300 -0.81504900  C 4.65142400 0.53605600 1.50544400  N 4.81553000 2.73276700 -1.42947500  N 4.95113800 0.57665600 2.65162300 |
|  | Total Energy: -665.5561282  Enthalpy: -665.389048  Free energy: -665.455347  ZPE Correction: -665.406690  C 1.59234000 -1.99035100 -0.27369100  C 2.86805700 -1.49823600 -0.19027800  C 2.86175400 -0.01038300 -0.06785900  C 0.67967200 -0.82404700 -0.18706000  C 1.59285200 0.45942200 -0.06698000  H 1.19303700 1.46019900 -0.00683600  C -0.60200700 -0.61434000 -0.13778800  C 4.26942900 -2.03855400 -0.23335600  H 4.57136200 -2.31787800 0.78902900  H 4.37201700 -2.93620600 -0.85697800  C 5.13100000 -0.81886900 -0.71082000  H 6.18270400 -0.92867600 -0.42241200  H 5.08879100 -0.74641200 -1.80669300  B 4.40784900 0.53097800 -0.04380500  H 1.24490600 -3.00953000 -0.37200400  Au -2.45226400 -0.06360300 -0.00867800  C 4.61840800 1.86609200 -0.86392000  C 4.84138000 0.71583400 1.47826000  N 4.75827700 2.85432300 -1.50214100  N 5.15662500 0.80943500 2.61708100  S -4.86176500 0.66012600 0.17468700  H -4.90141900 1.66521900 -0.76523000  H -4.79514500 1.49531800 1.26705000 |
|  | Total Energy: -780.5266862  Enthalpy: -780.255577  Free energy: -780.333422  ZPE Correction: -780.278297  C 2.32674900 -1.99655300 -0.24240200  C 3.58836700 -1.46984300 -0.16102500  C 3.54221000 0.01865000 -0.06078600  C 1.38263900 -0.85050200 -0.17861800  C 2.25701400 0.44821200 -0.07386900  H 1.83383300 1.44053400 -0.03037600  C 0.09061700 -0.69128800 -0.14231100  C 5.00456000 -1.97459600 -0.18906200  H 5.30807900 -2.23288400 0.83833500  H 5.13392500 -2.87697900 -0.80105700  C 5.83746900 -0.73915900 -0.67764500  H 6.88983700 -0.81788000 -0.38095900  H 5.80135200 -0.68432500 -1.77500300  B 5.07055900 0.59891900 -0.03695100  H 2.00707700 -3.02604300 -0.32784900  Au -1.81626400 -0.22498900 -0.04367100  C 5.25372100 1.92680500 -0.87818700  C 5.49726700 0.82270800 1.48339200  N 5.37423000 2.90752300 -1.53202200  N 5.80962300 0.94428400 2.62046600  P -4.16525400 0.41340200 0.09277000  C -4.53898900 1.58822300 1.50933900  H -5.60386100 1.84532100 1.51965700  H -4.26996500 1.11584900 2.45859000  H -3.94722200 2.50123900 1.39571200  C -5.32961600 -1.03885900 0.34865800  H -6.36564400 -0.68780700 0.40852300  H -5.23243700 -1.73983200 -0.48552600  H -5.06935600 -1.55915800 1.27505200  C -4.80768400 1.28427100 -1.44193100  H -4.69849300 0.62769500 -2.31006100  H -5.86344400 1.54814600 -1.31584600  H -4.22569300 2.19355700 -1.61837400 |
|  | Total Energy: -888.2413544  Enthalpy: -888.045065  Free energy: -888.121645  ZPE Correction: -888.066820  C 2.34621200 -2.03554800 -0.21648800  C 3.59281900 -1.47314100 -0.12896500  C 3.50597600 0.01545400 -0.07090800  C 1.37219400 -0.92103300 -0.19753800  C 2.21670700 0.42091500 -0.11664200  H 1.76338700 1.40020200 -0.10948100  C 0.08503300 -0.75287600 -0.17052800  C 5.02054500 -1.93995500 -0.12046400  H 5.31316000 -2.16202800 0.91856400  H 5.18334200 -2.85520000 -0.70440600  C 5.82845500 -0.69699900 -0.62981400  H 6.87751500 -0.73896700 -0.31511700  H 5.80760900 -0.67022300 -1.72828400  B 5.02210600 0.63927600 -0.03421800  H 2.05547700 -3.07523600 -0.27660500  Au -1.80240000 -0.26110000 -0.06542700  C 5.18552500 1.95057300 -0.90079300  C 5.40478800 0.90437300 1.48936000  N 5.29333300 2.92032800 -1.57261200  N 5.68375100 1.05714100 2.63126700  P -4.13580700 0.43618400 0.10828900  O -4.91084100 0.99262400 -1.28616300  H -5.34261500 1.87494200 -1.25544400  O -4.55161000 1.77857100 1.08428100  H -4.14080000 1.92143900 1.96163300  O -5.07883800 -0.85319100 0.64939600  H -6.05971300 -0.82407100 0.61064100 |
|  | Total Energy: -828.6503271  Enthalpy: -828.413004  Free energy: -828.489379  ZPE Correction: -828.434937  C 2.32994100 -2.02183700 -0.24634600  C 3.58482300 -1.47888500 -0.16314600  C 3.51930600 0.00852000 -0.06148200  C 1.37120900 -0.89085600 -0.18241300  C 2.23059500 0.42470200 -0.07484600  H 1.79384700 1.41093300 -0.03090700  C 0.08067200 -0.73023900 -0.14663800  C 5.00716700 -1.96484200 -0.19016300  H 5.31292100 -2.21974700 0.83742800  H 5.14889300 -2.86515000 -0.80238000  C 5.82426500 -0.71844600 -0.67722900  H 6.87728700 -0.78338500 -0.37967000  H 5.78821900 -0.66283500 -1.77447200  B 5.04094000 0.60980800 -0.03561300  H 2.02397600 -3.05535600 -0.33278900  Au -1.81590800 -0.23235800 -0.04439100  C 5.20605200 1.94019800 -0.87566100  C 5.46122000 0.83744300 1.48533300  N 5.31305000 2.92303700 -1.52859200  N 5.76914300 0.96223000 2.62323800  P -4.15647600 0.44201900 0.09745800  N -4.45940400 1.21664600 1.60329300  H -5.39416500 1.48471300 1.89266800  H -3.69101500 1.57688900 2.15597500  N -5.45834600 -0.70177000 0.10680100  H -5.67001000 -1.20988400 0.95740600  H -5.75397200 -1.12893200 -0.76304100  N -4.60357000 1.35395700 -1.29121000  H -3.89254500 1.77475000 -1.87682400  H -5.56185600 1.64169300 -1.45948900 |
|  |  |
|  | Total Energy: -681.2114229  Enthalpy: -680.939565  Free energy: -681.005228  ZPE Correction: -680.958821  Frequency: -313.4360  C -1.14876600 0.20421300 -0.05178200  C -2.40359000 -0.43271200 -0.08405400  C -3.61026400 0.37117300 -0.08366800  C -1.21709000 1.62703300 -0.00763000  C -3.75442000 1.65242400 -0.02215000  H -4.33575200 2.55592700 -0.01747800  C -1.78994500 2.73089900 0.02955500  C -2.71544800 -1.91610800 -0.09993100  H -2.69130100 -2.27014200 -1.14415400  H -1.98253000 -2.51099600 0.46241000  C -4.16900400 -2.02432400 0.44631100  H -4.64308200 -2.96979800 0.14458700  H -4.15123700 -2.00028900 1.54591000  B -4.99087800 -0.74634400 -0.17320600  H -5.95375100 -0.34391800 0.43147000  H -5.17612500 -0.80200900 -1.37287700  C 0.08418900 -0.56136100 -0.05655000  H -0.05386800 -1.63870800 -0.09882400  C 1.39082300 -0.10655300 -0.02428700  C 1.69215900 1.29582600 0.02480600  C 1.93419900 2.49447900 0.06668800  C 2.45391100 -1.11885500 -0.04363200  H 2.07371900 -2.14024900 -0.08467000  C 3.81810400 -0.99918300 -0.01821900  C 4.56264200 0.22615800 0.03635700  C 5.33868200 1.17444100 0.08213200  C 4.69012600 -2.25091500 -0.04746900  H 5.35696900 -2.23750700 -0.91972900  H 5.32801700 -2.29874900 0.84501500  H 4.08097100 -3.16043100 -0.08900300  H 2.16361000 3.53539500 0.10417600  H -1.97380100 3.78368800 0.05809500  H 5.97469400 2.02970400 0.12224600 |
|  | Total Energy: -1019.75152  Enthalpy: -1019.326687  Free energy: -1019.426570  ZPE Correction: -1019.358487  Frequency: -158.0200  C 1.49945200 1.90301500 0.05461300  C 1.26415100 3.28804400 0.07821000  C -0.11163100 3.75326000 0.09252700  C 0.32704100 1.08559400 0.03493300  C -1.20755300 3.08098800 0.05502800  H -2.26355200 2.90197700 0.05517000  C -0.84489500 0.65238900 0.02200500  C 2.24608800 4.44438200 0.07707400  H 2.55903600 4.64843800 1.11495300  H 3.15655800 4.22929600 -0.49980800  C 1.43572800 5.65812200 -0.46268100  H 1.89652900 6.61289200 -0.16795600  H 1.41614300 5.62610000 -1.56243100  B -0.07386500 5.52697000 0.16887900  H -0.98533800 6.03745300 -0.43765800  H -0.13442200 5.72976700 1.36599400  Au -2.56360100 -0.34889700 0.00395200  P -4.65589800 -1.58049200 -0.01680300  C -5.76839300 -1.26483300 1.46628400  H -6.68775300 -1.85658600 1.39111100  H -5.23678700 -1.53222600 2.38442400  H -6.02284700 -0.20163200 1.51150200  C -5.76181000 -1.22618200 -1.49609200  H -5.22631500 -1.47017400 -2.41847900  H -6.68184900 -1.81902500 -1.44028800  H -6.01563300 -0.16203300 -1.51503600  C -4.45232700 -3.44986400 -0.04052900  H -5.43004500 -3.94478600 -0.04778400  H -3.88961500 -3.74359000 -0.93175300  H -3.89137100 -3.76632600 0.84399300  C 2.86279300 1.40566800 0.05021800  H 3.61301900 2.19308200 0.07436100  C 3.35831500 0.10847400 0.02791300  C 2.48684800 -1.03207600 0.00202100  C 1.72150300 -1.98514200 -0.01974000  H 1.05394400 -2.81570200 -0.03963000  C 4.81465900 -0.02490500 0.03274100  H 5.32064700 0.94158300 0.05776900  C 5.66468600 -1.11580200 0.01112400  C 5.20270500 -2.47649600 -0.02302500  C 4.83895800 -3.64506600 -0.05209100  H 4.49598600 -4.65420700 -0.07727700  C 7.11430200 -0.81034100 0.02493000  H 7.31118400 0.26360300 0.05360600  C 8.22569300 -1.59699000 0.00859100  H 9.17309100 -1.05223800 0.02638200  C 8.38843300 -3.09770900 -0.03098000  H 7.44034700 -3.63238800 -0.04373500  H 8.96502100 -3.38939400 -0.92196500  H 8.96783400 -3.43564300 0.84156400 |
|  | Total Energy: -876.7640718  Enthalpy: -876.500292  Free energy: -876.572140  ZPE Correction: -876.521366  Frequency: -214.8002  C 0.59975600 0.84549200 -0.05352500  C 1.58872700 1.84354300 -0.08392100  C 2.98369800 1.44279400 -0.10091500  C 1.10206900 -0.48991000 -0.02830700  C 3.50429700 0.26343300 -0.05803700  H 4.30986700 -0.44291900 -0.06204800  C 1.88638400 -1.46729800 -0.00868500  C 1.43901100 3.35281800 -0.08589900  H 1.29388800 3.69467800 -1.12450100  H 0.56946300 3.69475500 0.49282700  C 2.79951800 3.89001200 0.44642200  H 2.96384300 4.93602800 0.14793800  H 2.80228900 3.85611400 1.54625400  B 3.95967800 2.91718500 -0.18925600  H 5.00525000 2.82157600 0.40759000  H 4.11467400 3.04528600 -1.38842700  C -0.80735600 1.20698600 -0.04552800  H -0.98489000 2.27983700 -0.06842700  C -1.93707200 0.40774600 -0.02253100  C -1.86172300 -1.02531700 0.00058400  C -1.80736900 -2.24701900 0.02012300  C -3.23535000 1.09716000 -0.02422400  H -3.14080600 2.18374400 -0.04393900  C -4.51988300 0.62239600 -0.00537000  C -4.92124500 -0.75530800 0.02184700  C -5.43122700 -1.87047700 0.04537800  C -5.69056200 1.60230700 -0.01264600  H -6.33213300 1.43278800 -0.88761900  H -6.31712500 1.46393700 0.87854500  H -5.34094700 2.64036900 -0.03391800  H -1.77949100 -3.31248400 0.03762700  H -5.82636400 -2.86071600 0.06554500  Cu 2.57634700 -3.18396800 0.03276700 |
|  | Total Energy: -1267.8535066  Enthalpy: -1267.605915  Free energy: -1267.689931  ZPE Correction: -1267.630317  Frequency: -206.6338  C 2.40346800 0.99673300 -0.05532700  C 3.70265100 1.53201700 -0.07280500  C 4.83439200 0.62477600 -0.08268300  C 2.34188500 -0.42964400 -0.03443300  C 4.88603600 -0.66023200 -0.04374600  H 5.34403200 -1.62792400 -0.04931400  C 2.67237100 -1.63714100 -0.01711200  C 4.14607000 2.98323300 -0.06946700  H 4.14604100 3.36105900 -1.10576900  H 3.47287600 3.62983100 0.51082400  C 5.60500100 2.95649600 0.47019100  H 6.16053700 3.86040500 0.17750000  H 5.58835400 2.92103700 1.57001000  B 6.31037500 1.61613000 -0.16162500  H 7.23360800 1.12543500 0.44426600  H 6.51169600 1.67300600 -1.35917300  C 1.24646200 1.87413600 -0.05395600  H 1.49656000 2.93243600 -0.07658800  C -0.10644600 1.57089600 -0.03659300  C -0.61678500 0.23289600 -0.01593400  C -1.12789200 -0.89492800 0.00165500  C -1.02675500 2.71937000 -0.04172800  H -0.50558000 3.67812800 -0.05798200  C -2.39576600 2.80649400 -0.02914400  C -3.35177900 1.73734900 -0.00917400  C -4.33202700 0.97410100 0.00652600  Cu 2.63627300 -3.48268700 0.01021800  Cu -2.19558400 -2.38926900 0.02071300  Cu -5.83123400 -0.08372100 0.02406900  C -3.04778200 4.19062800 -0.03699800  H -3.69798900 4.30542800 -0.91474800  H -3.67901900 4.32409400 0.85191800  H -2.29580000 4.98795700 -0.05342000 |
|  |  |
|  | Total Energy: -232.2133325  Enthalpy: -232.106773  Free energy: -232.139531  ZPE Correction: -232.112061  C -1.40676300 -0.08562100 0.00000100  C -0.62912400 -1.26093100 -0.00000800  C 0.77748400 -1.17531400 0.00001400  C 1.40675900 0.08567200 0.00000000  C 0.62917000 1.26090700 -0.00001000  C -0.77752700 1.17528600 0.00000700  H -2.49246400 -0.15184100 -0.00000300  H -1.11475800 -2.23425300 -0.00002400  H 1.37759400 -2.08253200 -0.00000100  H 2.49246900 0.15174500 -0.00000700  H 1.11467800 2.23429200 -0.00000500  H -1.37751600 2.08258500 0.00001500 |
|  | Total Energy: -367.0653224  Enthalpy: -366.967956  Free energy: -367.007097  ZPE Correction: -366.974785  C -2.93457800 1.21767700 -0.00000100  C -3.64118200 0.00002500 0.00000900  C -2.93465300 -1.21763500 -0.00000800  C -1.52426000 -1.22553400 0.00000300  C -0.82478600 -0.00004600 0.00000700  C -1.52421800 1.22551200 -0.00000600  H -3.47373000 2.16244700 -0.00000100  H -4.72862500 0.00007600 -0.00000200  H -3.47380100 -2.16240700 -0.00001300  H -0.98975000 -2.17055200 0.00000000  H -0.98961400 2.17047400 -0.00000400  Au 1.18933700 0.00000000 0.00000000 |
|  | Total Energy: -427.7382508  Enthalpy: -427.641313  Free energy: -427.679256  ZPE Correction: -427.648191  C -2.05324800 -1.21704800 0.00000300  C -2.76151500 -0.00001400 -0.00000300  C -2.05327500 1.21703200 0.00000800  C -0.64206300 1.21855700 0.00000100  C 0.08338400 0.00001800 -0.00000200  C -0.64203900 -1.21854200 0.00000500  H -2.59364600 -2.16213700 0.00000400  H -3.84960800 -0.00002900 -0.00000100  H -2.59368800 2.16211300 0.00001100  H -0.11980700 2.17287000 0.00000300  H -0.11975800 -2.17284200 0.00000400  Cu 1.98927700 0.00000000 -0.00000300 |
|  | Total Energy: -385.8331595  Enthalpy: -385.676622  Free energy: -385.715410  ZPE Correction: -385.684257  C 2.45174700 -0.71439600 -0.00000100  C 1.25209000 -1.41404400 0.00000100  C 0.00000500 -0.72083900 0.00000000  C -0.00000100 0.72084200 0.00000100  C 1.25208800 1.41404300 0.00000200  C 2.45174400 0.71439600 -0.00000100  H -1.25071500 -2.50240700 0.00000400  H 3.39830900 -1.24953200 -0.00000200  H 1.25072400 -2.50240400 0.00000000  C -1.25209400 -1.41404500 0.00000000  C -1.25209500 1.41404800 0.00000000  H 1.25072900 2.50240500 -0.00000100  H 3.39830700 1.24953100 -0.00000300  C -2.45174200 0.71439800 0.00000000  C -2.45174200 -0.71440200 -0.00000100  H -1.25071800 2.50240700 0.00000200  H -3.39831600 1.24951600 -0.00000200  H -3.39831200 -1.24952300 -0.00000100 |
|  | Total Energy: -655.5333563  Enthalpy: -655.395518  Free energy: -655.446834  ZPE Correction: -655.406725  C 3.14792300 2.44885900 0.00001000  C 3.81092800 1.23484300 0.00000600  C 3.08977600 -0.00009700 0.00000100  C 1.62979600 -0.00004800 0.00000100  C 0.98834900 1.29234400 0.00000400  C 1.72271800 2.47569000 0.00000900  H 4.89806300 -1.19815100 -0.00000500  H 3.69915900 3.38560700 0.00001400  H 4.89814700 1.19783200 0.00000500  C 3.81084200 -1.23508300 -0.00000500  C 0.98825700 -1.29238800 -0.00000400  H 1.21492700 3.43438400 0.00001100  C 1.72254900 -2.47578900 -0.00001100  C 3.14775100 -2.44905500 -0.00001100  H 1.21468800 -3.43444600 -0.00001600  H 3.69892600 -3.38584000 -0.00001700  Au -1.03777900 1.49866700 -0.00000400  Au -1.03788300 -1.49860400 0.00000400 |
|  | Total Energy: -776.8921038  Enthalpy: -776.754996  Free energy: -776.804063  ZPE Correction: -776.766144  C 2.25946600 -2.45135300 -0.00001000  C 2.93882600 -1.24367300 -0.00000500  C 2.23044300 0.00000400 0.00000000  C 0.77472900 0.00000000 0.00000000  C 0.10336500 -1.27566700 -0.00000500  C 0.82957100 -2.46625800 -0.00001000  H 4.02721200 1.22178300 0.00000400  H 2.80433000 -3.39311500 -0.00001300  H 4.02721800 -1.22176600 -0.00000500  C 2.93882000 1.24368500 0.00000500  C 0.10335900 1.27566400 0.00000500  H 0.32061300 -3.42746500 -0.00001300  C 0.82955900 2.46626000 0.00000900  C 2.25945400 2.45136200 0.00000900  H 0.32059700 3.42746400 0.00001300  H 2.80431300 3.39312600 0.00001300  Cu -1.82603500 1.28679400 0.00000600  Cu -1.82602900 -1.28680000 -0.00000600 |
|  | Total Energy: -539.4466007  Enthalpy: -539.240275  Free energy: -539.285115  ZPE Correction: -539.250473  C 3.68673400 0.71873300 -0.00001500  C 2.49429200 1.41783400 0.00004700  C 1.23152400 0.72673100 0.00004700  C 1.23152400 -0.72673100 0.00001300  C 2.49429200 -1.41783400 -0.00003300  C 3.68673400 -0.71873200 -0.00006400  C 0.00000000 1.41441400 0.00003400  C 0.00000100 -1.41441500 0.00001700  C -1.23152300 -0.72673200 0.00002500  C -1.23152400 0.72673100 0.00000700  C -2.49429100 1.41783400 -0.00005200  H -2.49346800 2.50608300 -0.00010500  C -3.68673400 0.71873300 -0.00005700  C -3.68673400 -0.71873200 -0.00000200  C -2.49429200 -1.41783400 0.00004200  H -0.00000400 2.50340200 0.00005500  H 4.63514900 1.25047700 -0.00003200  H 2.49346700 2.50608300 0.00012100  H 2.49346500 -2.50608300 -0.00004100  H 4.63514500 -1.25048300 -0.00012200  H -0.00000500 -2.50340300 0.00003800  H -4.63514600 1.25048200 -0.00008300  H -4.63514600 -1.25048000 -0.00000800  H -2.49347000 -2.50608300 0.00010800 |
|  | Total Energy: -943.9946276  Enthalpy: -943.816609  Free energy: -943.880299  ZPE Correction: -943.832414  C 3.67760100 3.27740800 -0.00006700  C 2.45305900 3.90411000 0.00002100  C 1.23134300 3.14557900 0.00005300  C 1.25334700 1.67742500 0.00008900  C 2.58121300 1.07945300 -0.00001200  C 3.73740600 1.84483400 -0.00010300  C 0.00000000 3.81837800 0.00008600  C 0.00000000 1.00018300 0.00009900  C -1.25334600 1.67742400 0.00008300  C -1.23134300 3.14557900 0.00004700  C -2.45306000 3.90411000 -0.00000700  H -2.38234900 4.98951400 -0.00003400  C -3.67760100 3.27740900 -0.00007000  C -3.73740700 1.84483400 -0.00007200  C -2.58121400 1.07945300 0.00000200  H -0.00000100 4.90685900 0.00006100  H 4.60225600 3.84838000 -0.00014200  H 2.38234900 4.98951400 0.00002400  H 4.71046800 1.36487600 -0.00020900  H -4.60225600 3.84838100 -0.00014900  H -4.71046900 1.36487700 -0.00014100  Au 2.89615600 -0.93991400 -0.00003400  Au 0.00000100 -1.07421500 0.00006700  Au -2.89615700 -0.93991400 -0.00003600 |
|  | Total Energy: -1126.0399011  Enthalpy: -1125.862727  Free energy: -1125.923484  ZPE Correction: -1125.878375  C 3.68673200 -2.35366300 -0.00000200  C 2.48106100 -3.02357500 -0.00000500  C 1.23355200 -2.30560500 -0.00000500  C 1.24579300 -0.83921900 0.00000100  C 2.53780500 -0.17936700 0.00000200  C 3.71475800 -0.91469400 0.00000100  C 0.00000000 -2.98449500 -0.00000700  C 0.00000000 -0.16655600 0.00000000  C -1.24579400 -0.83921900 -0.00000200  C -1.23355200 -2.30560500 -0.00000700  C -2.48106100 -3.02357500 -0.00000900  H -2.45133300 -4.11160100 -0.00001200  C -3.68673200 -2.35366300 -0.00000900  C -3.71475800 -0.91469400 -0.00000600  C -2.53780500 -0.17936700 -0.00000300  H 0.00000000 -4.07383500 -0.00001000  H 4.62597700 -2.90280000 -0.00000200  H 2.45133300 -4.11160200 -0.00000800  H 4.68232400 -0.41809300 0.00000300  H -4.62597700 -2.90279900 -0.00001000  H -4.68232400 -0.41809200 -0.00000500  Cu -2.57263800 1.74910000 0.00000000  Cu 0.00000100 1.78588900 0.00000500  Cu 2.57263800 1.74910000 0.00000700 |
|  | Total Energy: -693.0576501  Enthalpy: -692.801623  Free energy: -692.852510  ZPE Correction: -692.814442  C 4.92334600 0.72077400 0.00000000  C 3.73389100 1.41985000 0.00000000  C 2.46638200 0.72991500 0.00000000  C 2.46638200 -0.72991500 0.00000000  C 3.73389100 -1.41985000 0.00000000  C 4.92334600 -0.72077400 0.00000000  C 1.24298900 1.41681400 0.00000000  C 1.24298900 -1.41681400 0.00000000  C 0.00007700 -0.73082700 0.00000000  C 0.00007700 0.73082700 0.00000000  C -1.24303000 1.41678100 0.00000000  H -1.24323100 2.50573400 0.00000000  C -2.46627300 0.73007100 0.00000000  C -2.46627300 -0.73007100 0.00000000  C -1.24303000 -1.41678100 0.00000000  H 1.24345100 2.50575500 0.00000000  H 5.87238000 1.25132900 0.00000000  H 3.73350300 2.50808600 0.00000000  H 3.73350300 -2.50808600 0.00000000  H 5.87238000 -1.25132900 0.00000000  H 1.24345100 -2.50575500 0.00000000  H -1.24323100 -2.50573400 0.00000000  C -3.73398200 -1.41990000 0.00000000  H -3.73376700 -2.50813700 0.00000000  C -4.92338700 -0.72081200 -0.00000100  H -5.87242000 -1.25137500 -0.00000100  C -3.73398200 1.41990000 0.00000000  H -3.73376700 2.50813700 0.00000000  C -4.92338700 0.72081200 0.00000000  H -5.87242000 1.25137500 0.00000000 |
|  | Total Energy: -1232.4531808  Enthalpy: -1232.235122  Free energy: -1232.311468  ZPE Correction: -1232.255585  C -5.00184600 1.96158800 0.00000000  C -3.87091100 1.16429700 0.00000000  C -2.51872900 1.72039800 0.00000000  C -2.46013300 3.19397500 0.00000000  C -3.66424800 3.98702500 0.00000000  C -4.90296200 3.39622500 0.00000000  C -1.29223600 1.00900600 0.00000000  C -1.21876200 3.82757400 0.00000000  C 0.00042300 3.11613100 0.00000000  C 0.00047800 1.64424100 0.00000000  C 1.29272400 1.00879900 0.00000000  C 2.51957700 1.71990300 0.00000000  C 2.46104300 3.19326400 0.00000000  C 1.21970600 3.82725500 0.00000000  H -5.98917200 1.51136300 0.00000000  H -3.56172200 5.06984800 0.00000000  H -5.81249600 3.99092900 0.00000000  H -1.18369300 4.91531600 0.00000000  H 1.18513400 4.91500600 0.00000000  C 3.66508300 3.98628800 0.00000000  H 3.56259800 5.06910900 0.00000100  C 4.90376200 3.39545300 -0.00000100  H 5.81332500 3.99011700 -0.00000100  C 3.87155100 1.16362700 0.00000000  C 5.00249900 1.96102700 -0.00000100  H 5.98980200 1.51076000 -0.00000100  Au -4.26613000 -0.84299600 0.00000000  Au -1.40685000 -1.07221100 0.00000000  Au 1.40465000 -1.07225600 0.00000000  Au 4.26775000 -0.84328300 0.00000000 |
|  | Total Energy: -1475.1852588  Enthalpy: -1474.969037  Free energy: -1475.037754  ZPE Correction: -1474.988323  C 4.96439600 0.97353800 0.00000000  C 3.79627200 0.22970400 0.00000000  C 2.49361900 0.87737100 0.00000000  C 2.46907200 2.35085700 0.00000000  C 3.71627600 3.07679000 0.00000000  C 4.92412300 2.41645100 0.00000000  C 1.26207300 0.19590200 0.00000000  C 1.23876900 3.02026200 0.00000000  C -0.00009000 2.33245300 0.00000000  C -0.00006900 0.85990300 0.00000000  C -1.26219300 0.19586000 0.00000000  C -2.49376300 0.87728900 0.00000000  C -2.46925300 2.35077800 0.00000000  C -1.23897100 3.02022100 0.00000000  H 5.93696000 0.48678600 0.00000000  H 3.67865000 4.16453500 0.00000000  H 5.85977500 2.97168200 0.00000000  H 1.23138700 4.10939900 0.00000000  H -1.23162200 4.10935900 0.00000000  C -3.71647400 3.07668000 0.00000000  H -3.67887700 4.16442600 0.00000000  C -4.92430300 2.41631000 0.00000000  H -5.85996900 2.97151700 0.00000000  C -3.79640600 0.22958300 0.00000000  C -4.96454100 0.97339500 0.00000000  H -5.93709300 0.48661900 0.00000000  Cu 3.85769500 -1.69866800 0.00000000  Cu 1.28645100 -1.75476900 0.00000000  Cu -1.28622500 -1.75481600 0.00000000  Cu -3.85759100 -1.69879600 0.00000000 |
|  | Total Energy: -846.6675657  Enthalpy: -846.361870  Free energy: -846.418803  ZPE Correction: -846.377336  C -6.16033300 -0.72197200 0.00013900  C -4.97234500 -1.42097000 0.00010400  C -3.70247700 -0.73148400 0.00008600  C -3.70249800 0.73146600 0.00010300  C -4.97244500 1.42080700 0.00013800  C -6.16036200 0.72173300 0.00015600  C -2.48297200 -1.41812500 0.00004900  C -2.48307000 1.41821100 0.00008300  C -1.23455400 0.73307900 0.00004700  C -1.23454100 -0.73279000 0.00003200  C 0.00000100 -1.41842300 -0.00000900  C 1.23453900 -0.73279100 -0.00002900  C 1.23455200 0.73308000 -0.00001400  C 0.00000100 1.41864000 0.00002300  H -7.10972300 -1.25189600 0.00015300  H -4.97216100 2.50904100 0.00015000  H -7.10985800 1.25146900 0.00018300  H -2.48360800 2.50712000 0.00009300  H -0.00000100 2.50748600 0.00003200  C 2.48307300 1.41821200 -0.00004300  H 2.48360600 2.50712000 -0.00003400  C 3.70249600 0.73146700 -0.00008600  C 2.48297400 -1.41812600 -0.00007600  C 3.70247600 -0.73148500 -0.00009100  C 4.97234700 -1.42097100 -0.00014400  C 6.16033200 -0.72197300 -0.00018000  H 7.10972400 -1.25189400 -0.00022200  C 4.97244700 1.42080800 -0.00011300  H 4.97215800 2.50904200 -0.00010600  C 6.16036100 0.72173500 -0.00015700  H 7.10985900 1.25146600 -0.00018700  H 4.97198500 -2.50921000 -0.00016400  H 2.48332100 -2.50703000 -0.00009500  H -0.00000100 -2.50725500 -0.00002600  H -2.48332500 -2.50702900 0.00003400  H -4.97198700 -2.50920900 0.00009000 |
|  | Total Energy: -1520.9102924  Enthalpy: -1520.652242  Free energy: -1520.741690  ZPE Correction: -1520.677392  C 6.26558500 2.08929400 -0.00034900  C 5.16018400 1.25905500 -0.00018700  C 3.78862000 1.77284000 -0.00004200  C 3.68858400 3.24762600 -0.00009600  C 4.87077600 4.07589700 -0.00027000  C 6.12496800 3.52241900 -0.00038800  C 2.58672400 1.02676900 0.00009600  C 2.43325100 3.84373500 -0.00001000  C 1.23050700 3.09737200 0.00014000  C 1.26767900 1.62172600 0.00020600  C 0.00045100 0.95082500 0.00028700  C -1.26642300 1.62185000 0.00034300  C -1.22933400 3.09748700 0.00027900  C 0.00053600 3.76997500 0.00017200  H 7.26625200 1.66941100 -0.00046100  H 4.73578700 5.15512800 -0.00031000  H 7.01714900 4.14275000 -0.00052500  H 2.36561300 4.92986200 -0.00007400  H 0.00066100 4.85807000 0.00008400  C -2.43222100 3.84379600 0.00022200  H -2.36454200 4.92991900 0.00011100  C -3.68742800 3.24763300 0.00022300  C -2.58559100 1.02667000 0.00033500  C -3.78738100 1.77271800 0.00035700  Au 5.62977800 -0.73323500 -0.00019700  Au 2.78710200 -1.04870400 0.00009800  Au 0.00010100 -1.14149400 0.00024600  Au -2.78762300 -1.04830700 0.00016000  C -5.15916400 1.25927000 0.00014100  C -6.26438300 2.08953200 -0.00014200  H -7.26516100 1.66994100 -0.00045400  C -4.86963200 4.07616200 0.00012300  H -4.73425300 5.15533800 0.00004900  C -6.12379300 3.52289400 0.00003000  H -7.01609400 4.14302000 -0.00017500  Au -5.63037700 -0.73290100 -0.00039600 |
|  | Total Energy: -1824.3294407  Enthalpy: -1824.073287  Free energy: -1824.153580  ZPE Correction: -1824.097162  C -6.21433000 1.02359000 0.00015300  C -5.05302100 0.27163200 0.00011900  C -3.74304100 0.90811700 0.00009100  C -3.70689800 2.38498500 0.00010000  C -4.95118200 3.11940900 0.00013700  C -6.16256500 2.46829000 0.00016200  C -2.52051300 0.21838200 0.00005900  C -2.47552300 3.04513200 0.00007400  C -1.23569400 2.35011800 0.00003900  C -1.24781400 0.87222700 0.00003100  C -0.00023000 0.19890700 -0.00000300  C 1.24739100 0.87218900 -0.00002800  C 1.23523600 2.35008000 -0.00002000  C -0.00022000 3.02866200 0.00001400  H -7.19108000 0.54531200 0.00017400  H -4.90565900 4.20684200 0.00014400  H -7.09430400 3.03006000 0.00019000  H -2.46086500 4.13415400 0.00008100  H -0.00020600 4.11762800 0.00002200  C 2.47503700 3.04509300 -0.00004400  H 2.46034600 4.13411400 -0.00003700  C 3.70642900 2.38500800 -0.00007500  C 2.52011800 0.21833200 -0.00005900  C 3.74273400 0.90810100 -0.00008700  C 5.05297000 0.27192700 -0.00011700  C 6.21410800 1.02415700 -0.00013600  H 7.19096600 0.54609900 -0.00015700  C 4.95056800 3.11966600 -0.00010000  H 4.90482600 4.20708800 -0.00009200  C 6.16207400 2.46882400 -0.00013000  H 7.09370000 3.03078200 -0.00014600  Cu 5.14143900 -1.65570200 -0.00013600  Cu 2.57263900 -1.73253000 -0.00004900  Cu -0.00000700 -1.74986000 -0.00002400  Cu -2.57278400 -1.73240400 0.00006300  Cu -5.14030600 -1.65602300 0.00010100 |
|  | Total Energy: -1000.2769073  Enthalpy: -999.921575  Free energy: -999.984552  ZPE Correction: -999.939700  C -4.93919700 0.73239900 -0.00057400  C -3.72162300 1.41889100 -0.00044500  C -2.47046200 0.73395400 -0.00028800  C -2.47052300 -0.73435900 -0.00026600  C -3.72184400 -1.41898800 -0.00039900  C -4.93928600 -0.73228500 -0.00055100  C -1.24004700 1.41940200 -0.00016000  C -1.24011400 -1.41980100 -0.00011500  C 0.00000000 -0.73497600 0.00001600  C 0.00000000 0.73444000 -0.00000500  C 1.24004700 1.41940200 0.00012400  C 2.47046200 0.73395400 0.00027500  C 2.47052300 -0.73435900 0.00029500  C 1.24011500 -1.41980100 0.00016600  H -3.72247800 -2.50789100 -0.00038300  H -1.24025000 -2.50861900 -0.00010000  H 1.24025000 -2.50861900 0.00018000  C 3.72184400 -1.41898800 0.00044500  H 3.72247800 -2.50789100 0.00046000  C 4.93928600 -0.73228500 0.00057100  C 3.72162300 1.41889100 0.00039900  C 4.93919700 0.73239900 0.00055500  C 6.21021800 1.42163800 0.00068800  C 7.39745400 0.72268700 0.00082500  H 8.34702500 1.25228300 0.00091900  C 6.21045500 -1.42126700 0.00072600  H 6.21026100 -2.50949400 0.00074000  C 7.39756100 -0.72213500 0.00084700  H 8.34731000 -1.25139700 0.00096000  C -6.21021800 1.42163800 -0.00073400  C -7.39745400 0.72268700 -0.00086100  H -8.34702500 1.25228300 -0.00098200  C -6.21045500 -1.42126700 -0.00068800  H -6.21026100 -2.50949400 -0.00067000  C -7.39756100 -0.72213500 -0.00083800  H -8.34731000 -1.25139700 -0.00094100  H 6.20992700 2.50988400 0.00066900  H 3.72199700 2.50780300 0.00038100  H 1.24012000 2.50822000 0.00010500  H -1.24012000 2.50822000 -0.00017900  H -3.72199700 2.50780300 -0.00046400  H -6.20992700 2.50988400 -0.00075300 |
|  | Total Energy: -1809.3666032  Enthalpy: -1809.068593  Free energy: -1809.171719  ZPE Correction: -1809.098442  C 5.05638000 1.84320100 -0.00037000  C 3.87931400 1.06155400 -0.00016100  C 2.53828700 1.61531100 -0.00017900  C 2.45920100 3.09179900 -0.00032600  C 3.64153100 3.87379800 -0.00055500  C 4.91215700 3.31640100 -0.00061500  C 1.29513500 0.90823000 -0.00006400  C 1.21464500 3.72725200 -0.00021500  C -0.00001100 3.01942800 -0.00006300  C -0.00001100 1.54013300 -0.00006900  C -1.29515700 0.90822600 -0.00004400  C -2.53831100 1.61530000 -0.00000400  C -2.45922500 3.09179000 0.00021100  C -1.21467000 3.72724800 0.00014000  H 3.54064000 4.95731200 -0.00069300  H 1.18195400 4.81477900 -0.00025000  H -1.18198400 4.81477400 0.00026000  C -3.64155400 3.87379100 0.00056700  H -3.54065900 4.95730400 0.00085900  C -4.91218100 3.31639600 0.00057000  C -3.87934100 1.06154700 -0.00004700  C -5.05640500 1.84319400 0.00010300  Au 4.16150200 -1.00502000 0.00025000  Au 1.38456100 -1.18190500 0.00016900  Au -1.38454300 -1.18191800 0.00006100  Au -4.16148600 -1.00503600 -0.00024700  C -6.44531000 1.37355500 0.00019300  C -7.52355300 2.23760200 0.00104600  H -8.53701300 1.84955100 0.00130400  C -6.06930600 4.18089600 0.00107800  H -5.90054800 5.25536300 0.00105700  C -7.33920000 3.66689500 0.00142200  H -8.21232000 4.31373700 0.00166900  Au -6.98812800 -0.60101300 -0.00033900  C 6.44528200 1.37355600 -0.00037000  C 7.52352500 2.23760000 -0.00064800  H 8.53698800 1.84955600 -0.00064100  C 6.06928200 4.18090000 -0.00089700  H 5.90052200 5.25536700 -0.00108200  C 7.33917200 3.66689600 -0.00093200  H 8.21229700 4.31373100 -0.00114800  Au 6.98812000 -0.60101200 0.00010800 |
|  | Total Energy: -2173.4730922  Enthalpy: -2173.177076  Free energy: -2173.269589  ZPE Correction: -2173.205587  C 4.99239500 0.93723400 -0.00052200  C 3.77643300 0.23939400 -0.00038000  C 2.49631400 0.88383500 -0.00024700  C 2.47297200 2.36426500 -0.00025000  C 3.71088000 3.06698400 -0.00038000  C 4.94505900 2.41579400 -0.00051000  C 1.25781700 0.20188600 -0.00013300  C 1.23680100 3.03404400 -0.00010300  C -0.00001200 2.34860900 0.00002300  C -0.00001000 0.86640300 0.00001500  C -1.25783800 0.20188700 0.00014400  C -2.49633700 0.88383200 0.00028000  C -2.47299600 2.36426200 0.00029000  C -1.23682600 3.03404200 0.00017200  H 3.68881600 4.15587100 -0.00038300  H 1.22984800 4.12295000 -0.00008500  H -1.22987400 4.12294700 0.00017900  C -3.71090700 3.06697700 0.00046200  H -3.68884700 4.15586400 0.00049500  C -4.94508500 2.41578400 0.00060200  C -3.77645400 0.23938800 0.00038400  C -4.99241800 0.93722500 0.00052600  C -6.30893900 0.31178500 0.00067300  C -7.46373700 1.07241500 0.00082600  H -8.44448700 0.60233700 0.00092500  C -6.18498100 3.15929600 0.00073500  H -6.13113800 4.24633600 0.00077100  C -7.40063400 2.51771700 0.00083200  H -8.32824500 3.08623900 0.00097200  C 6.30891500 0.31179500 -0.00067000  C 7.46371200 1.07242900 -0.00079900  H 8.44446200 0.60235400 -0.00091200  C 6.18495200 3.15930900 -0.00065400  H 6.13110700 4.24634900 -0.00065000  C 7.40060400 2.51773200 -0.00079500  H 8.32821400 3.08625700 -0.00090400  Cu -6.42081400 -1.61489100 0.00064700  Cu -3.85884300 -1.71037600 0.00027900  Cu -1.28825400 -1.74669900 0.00018800  Cu 1.28827900 -1.74670400 -0.00023900  Cu 3.85885300 -1.71036400 -0.00028700  Cu 6.42085000 -1.61487700 -0.00070900 |

**All calculations were performed at the B3LYP/LANL2DZ level except when different method specified in the table. All energies are expressed in hartrees (Conversion factors: 1 hartree = 627.5 kcal, 1 hartree = 27.2 eV). Frequencies are expressed in cm^-1^.*

Table 2. HOMO and LUMO of selected compounds obtained using the B3LYP/LANL2DZ level.

| Compounds | HOMO | LUMO |
| --- | --- | --- |
|  | 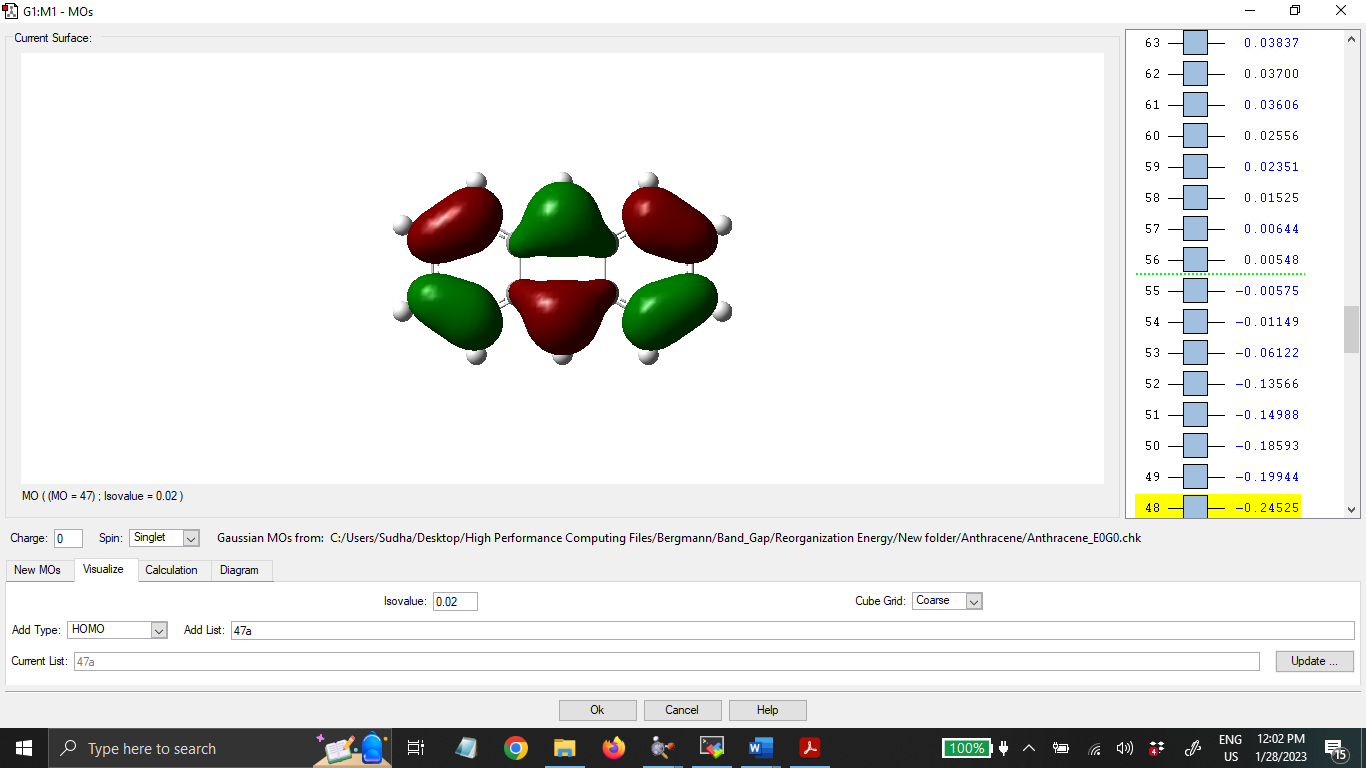 | 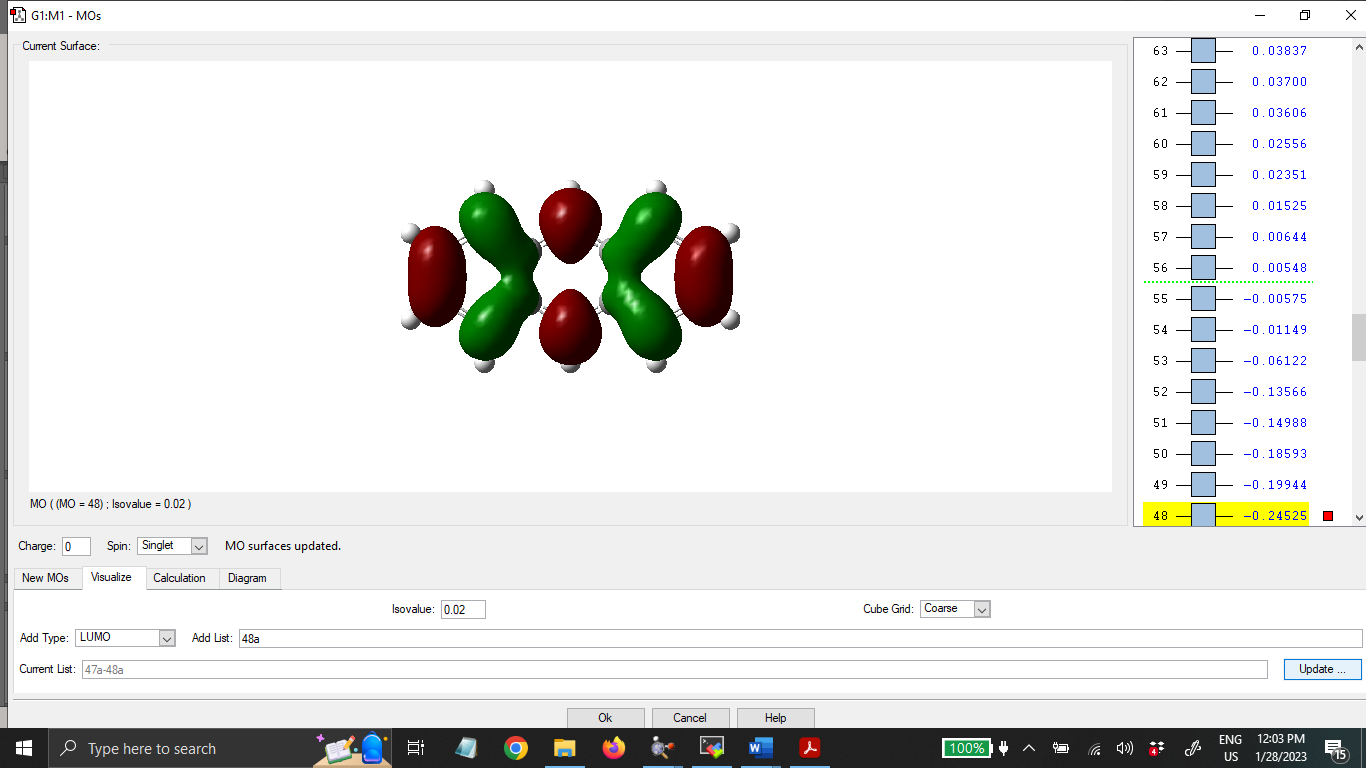 |
|  | 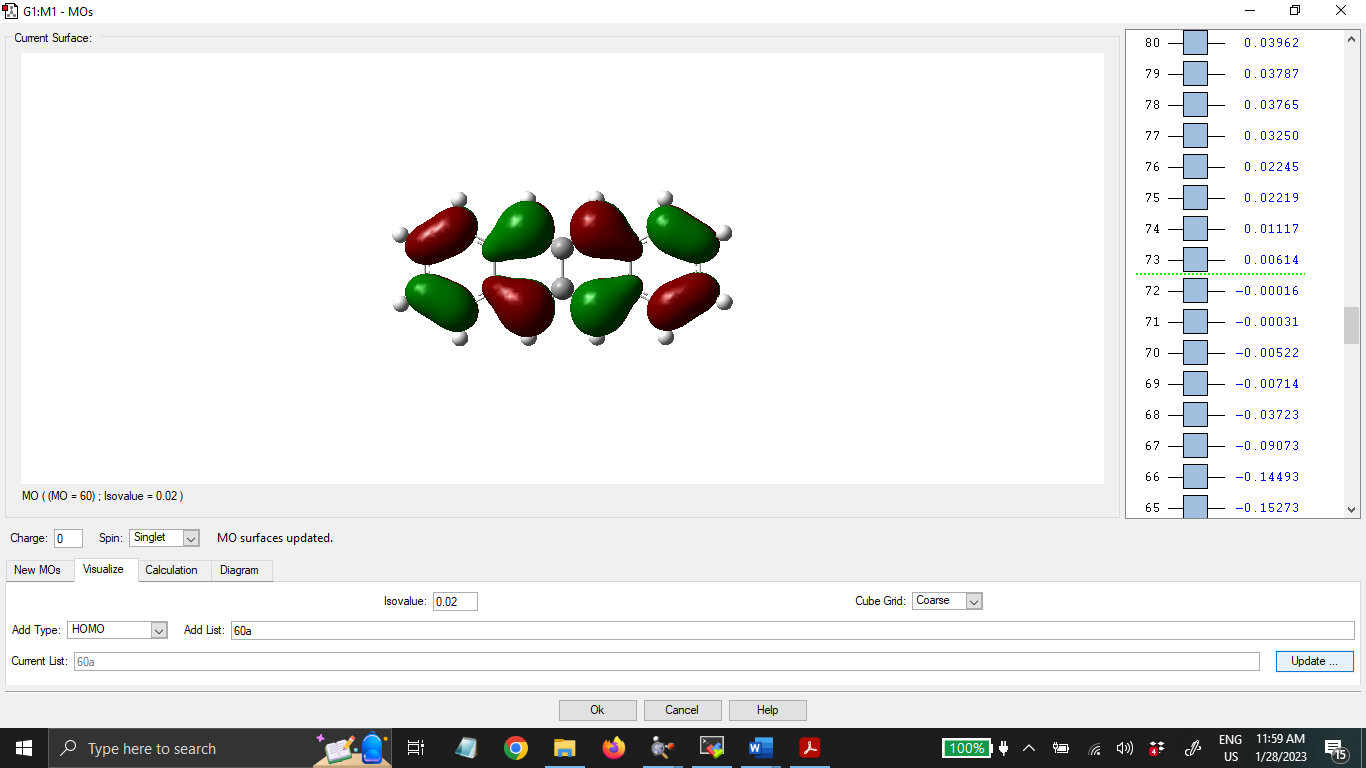 | 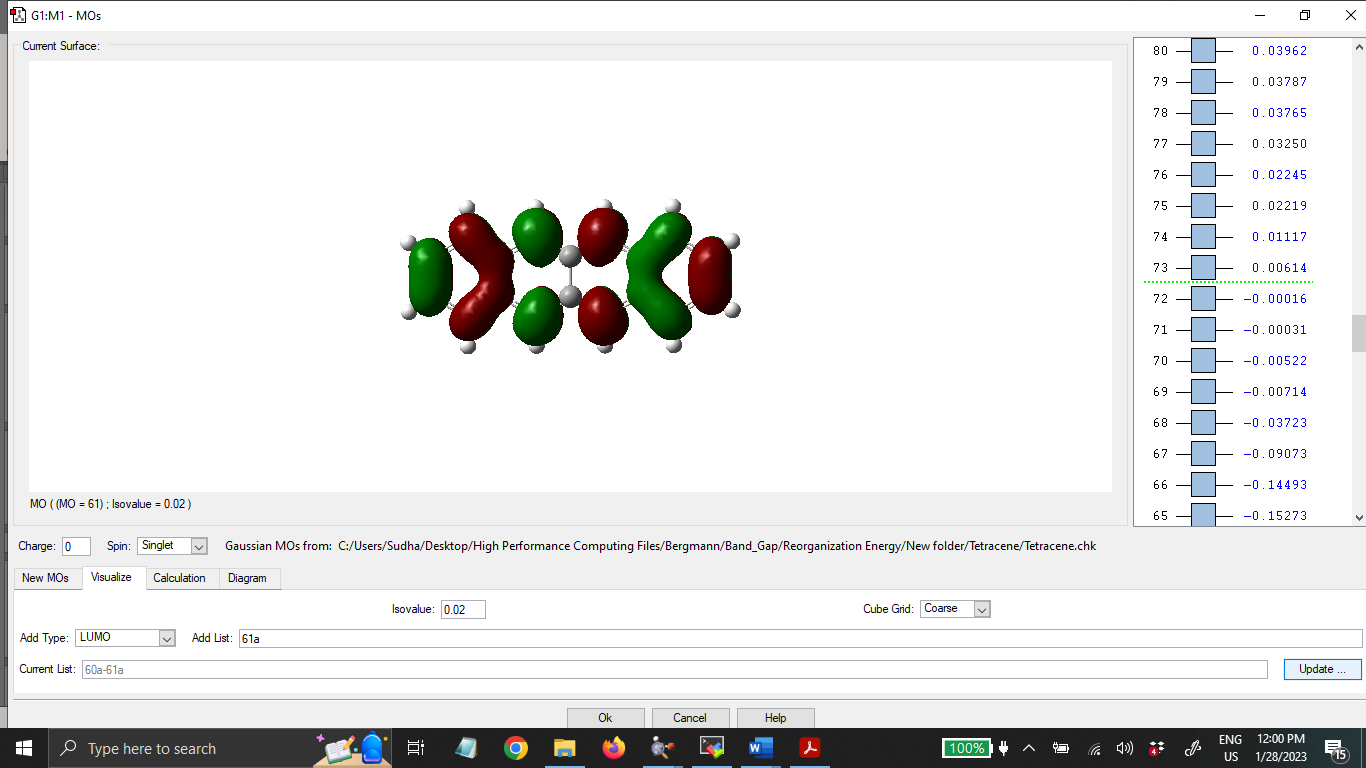 |
|  | 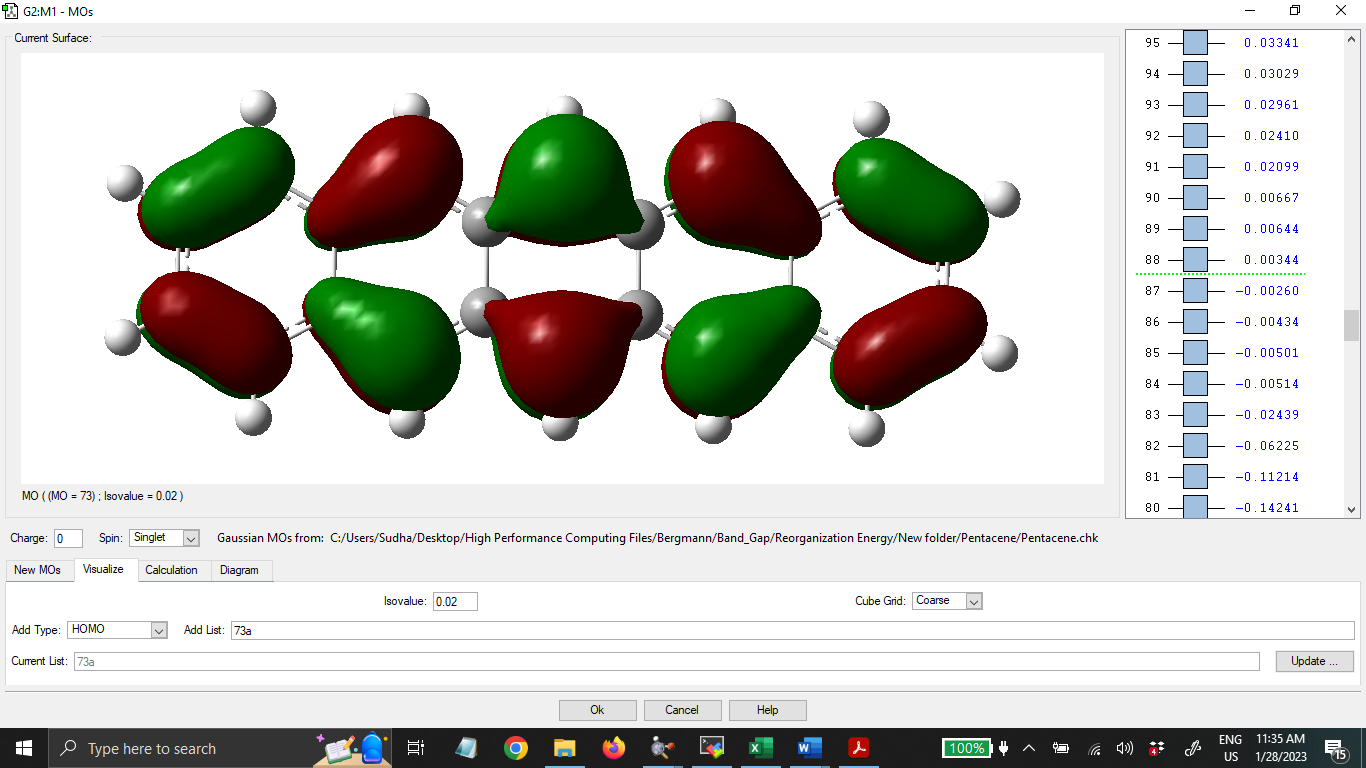 | 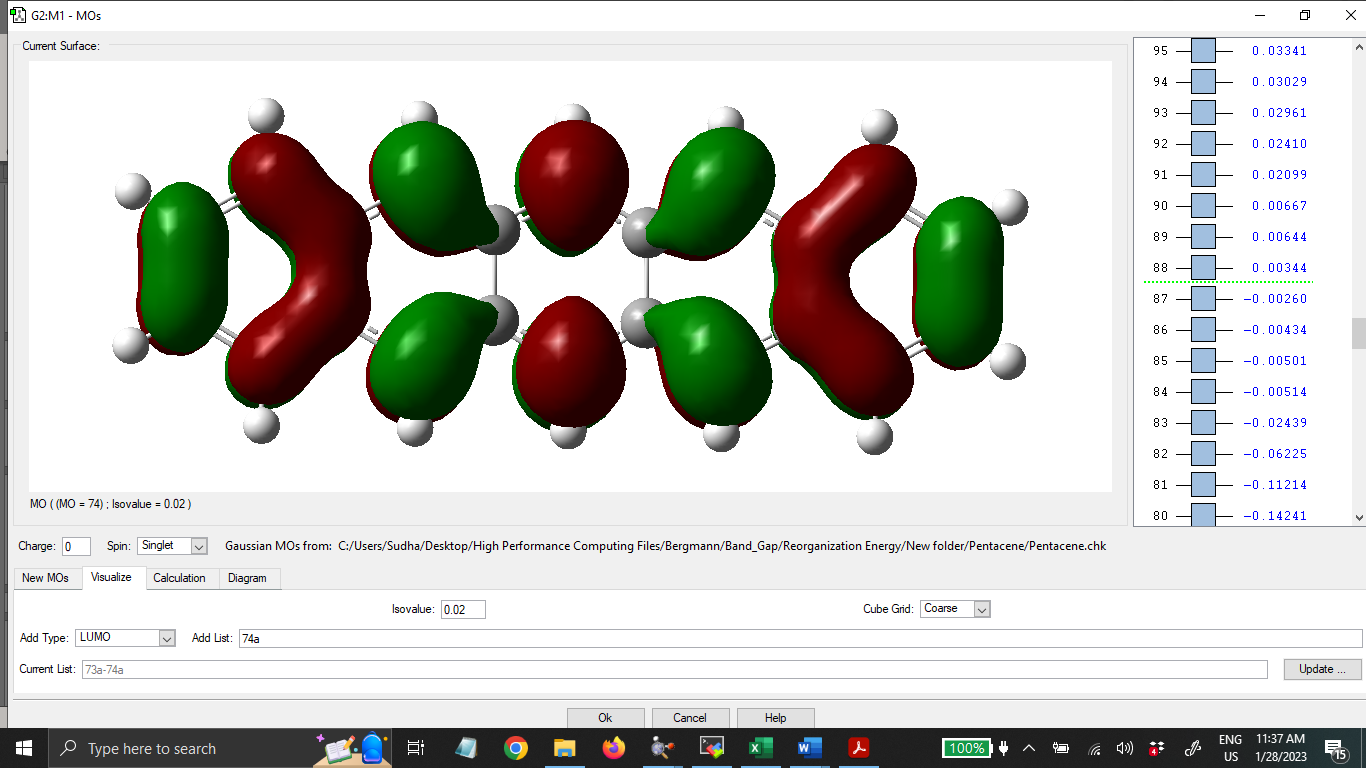 |
|  |  |  |
|  | 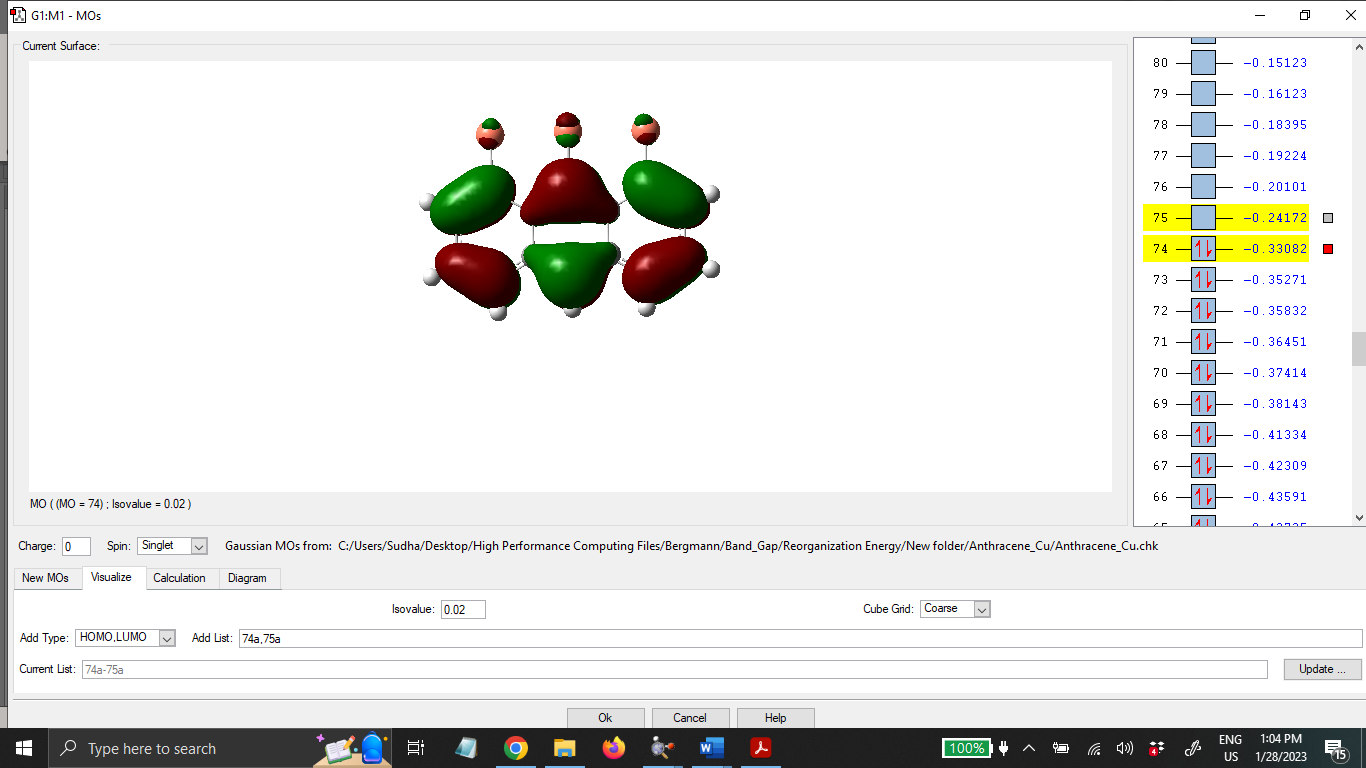 | 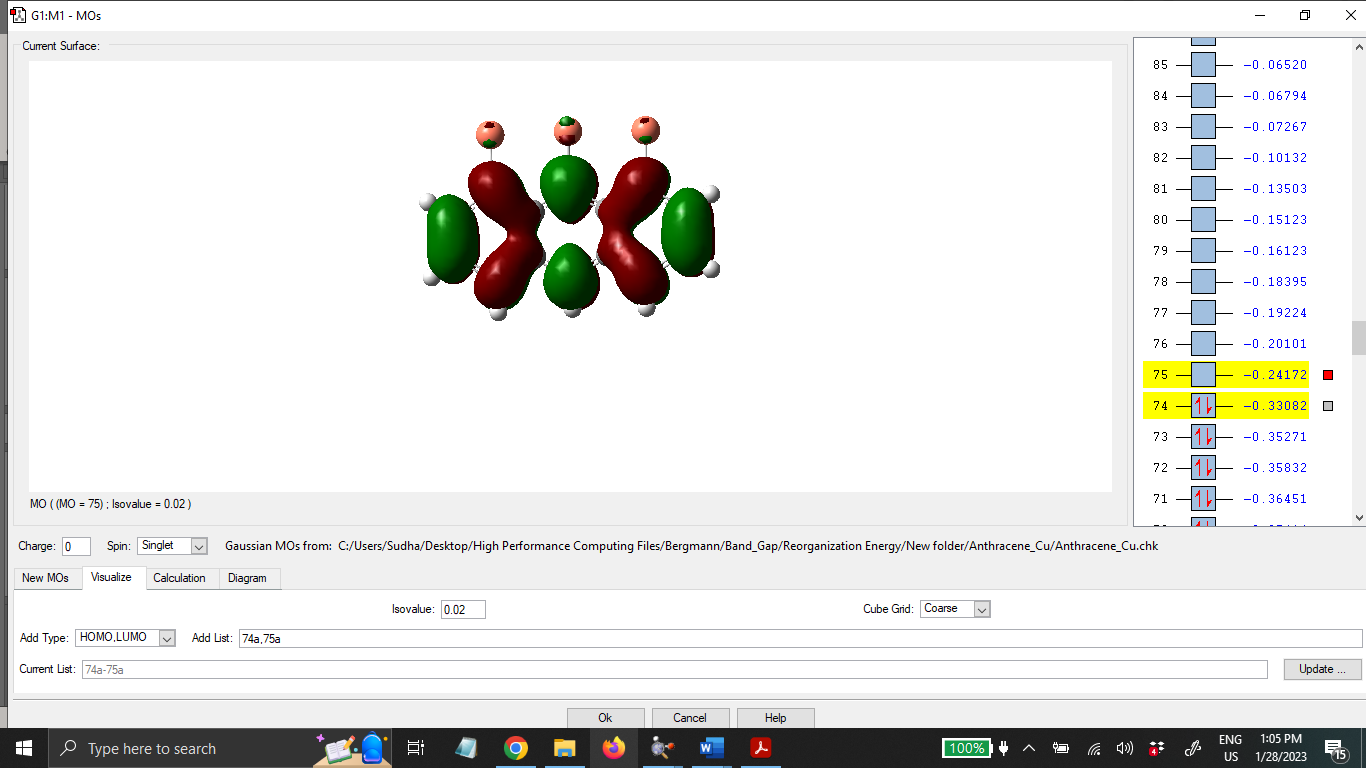 |
|  | 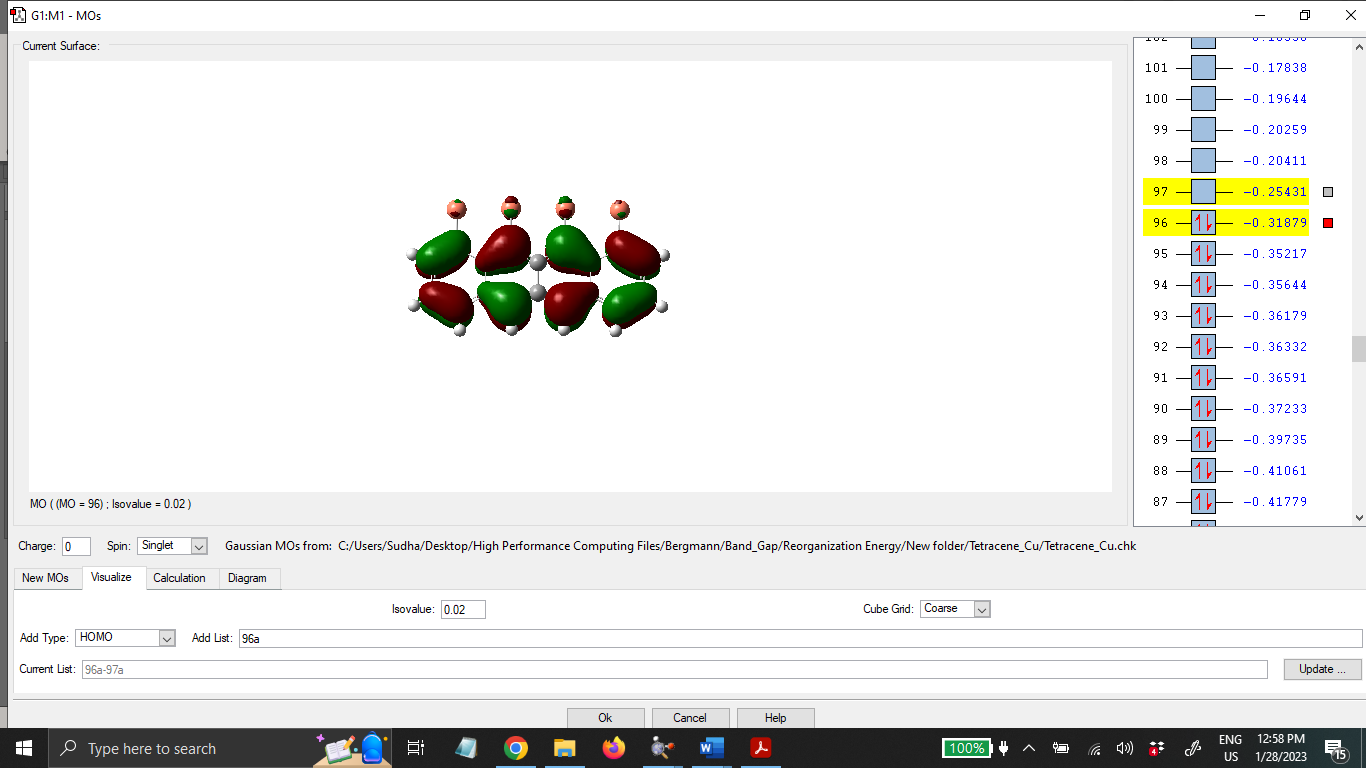 | 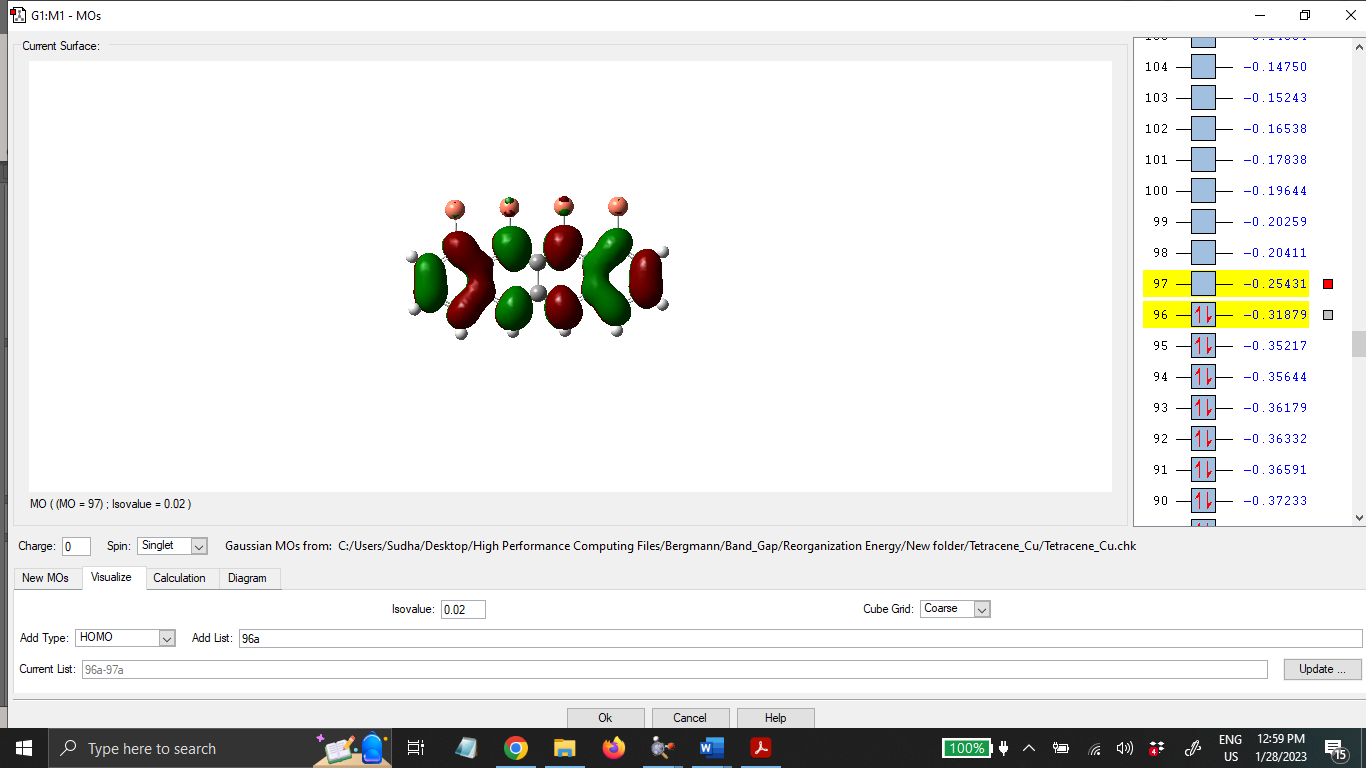 |
|  | 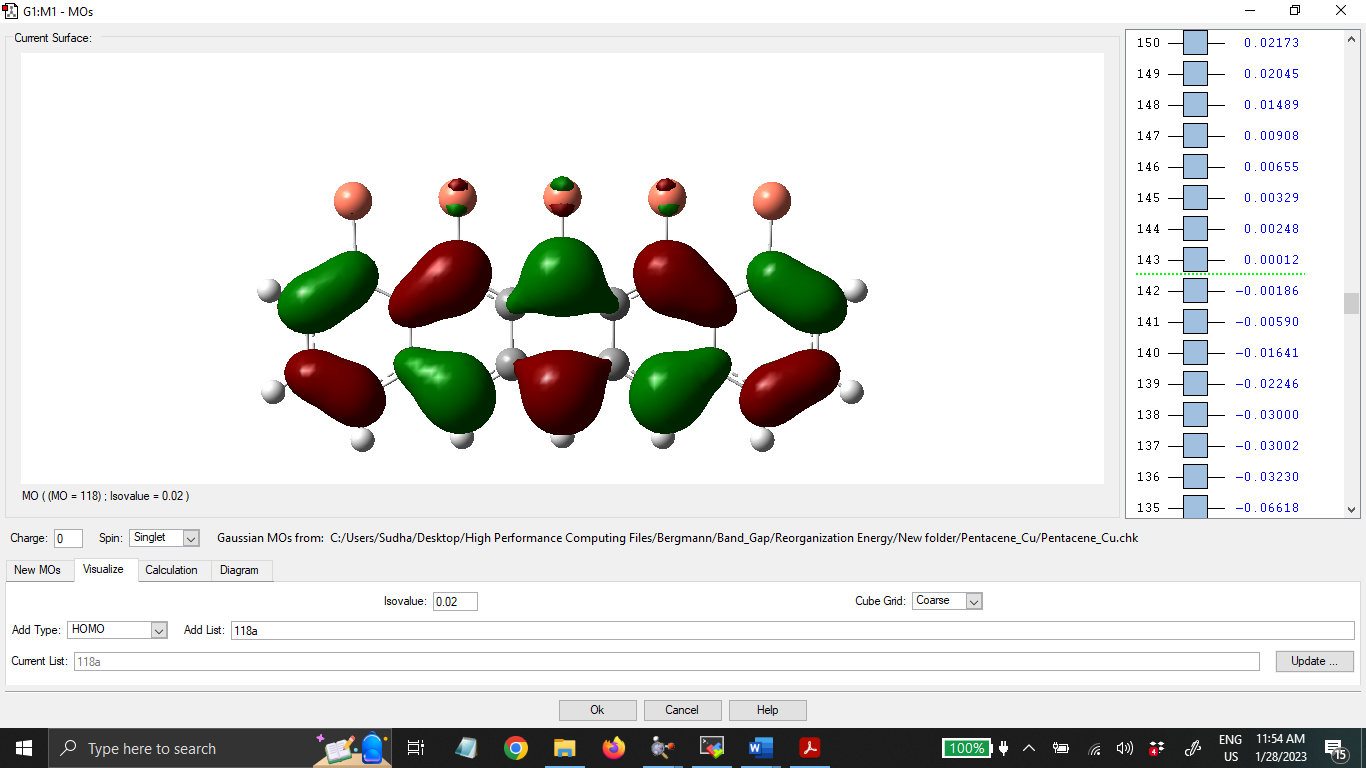 | 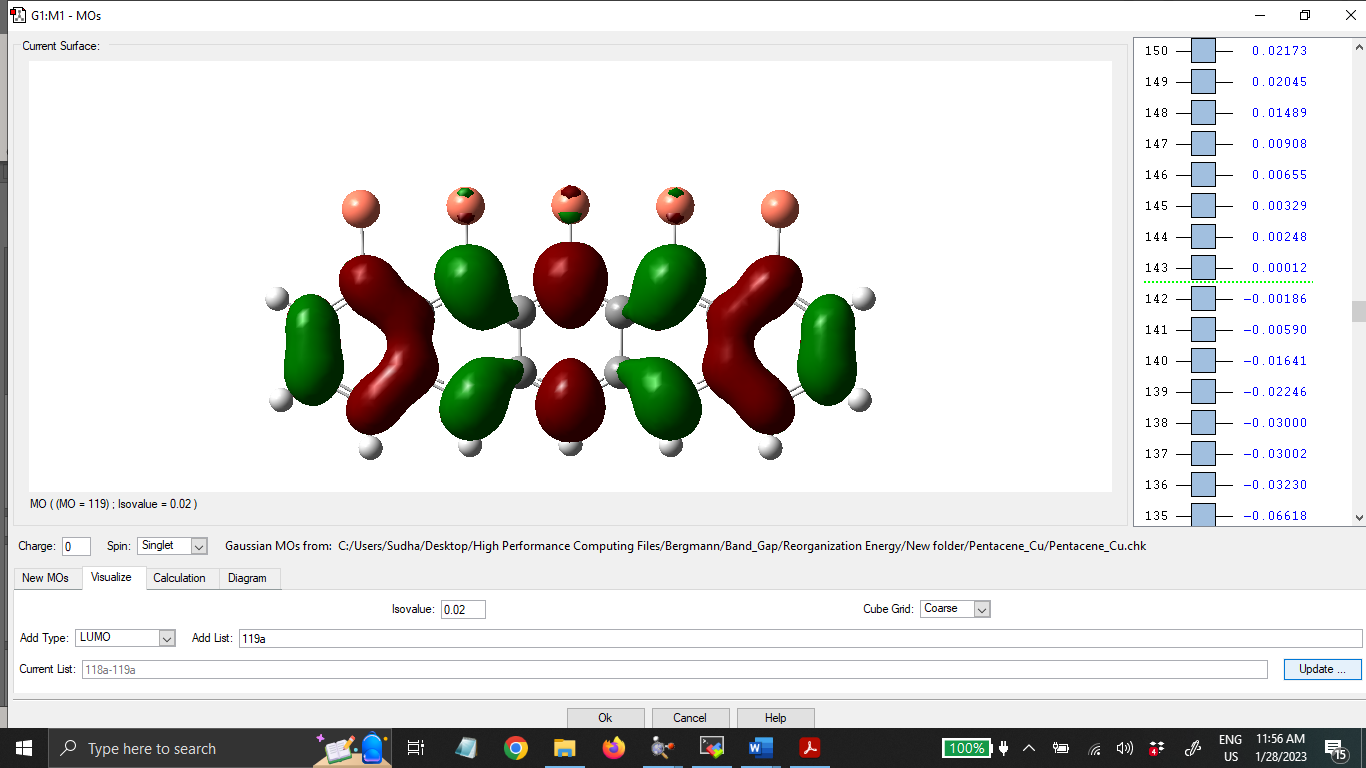 |
|  |  |  |
|  | 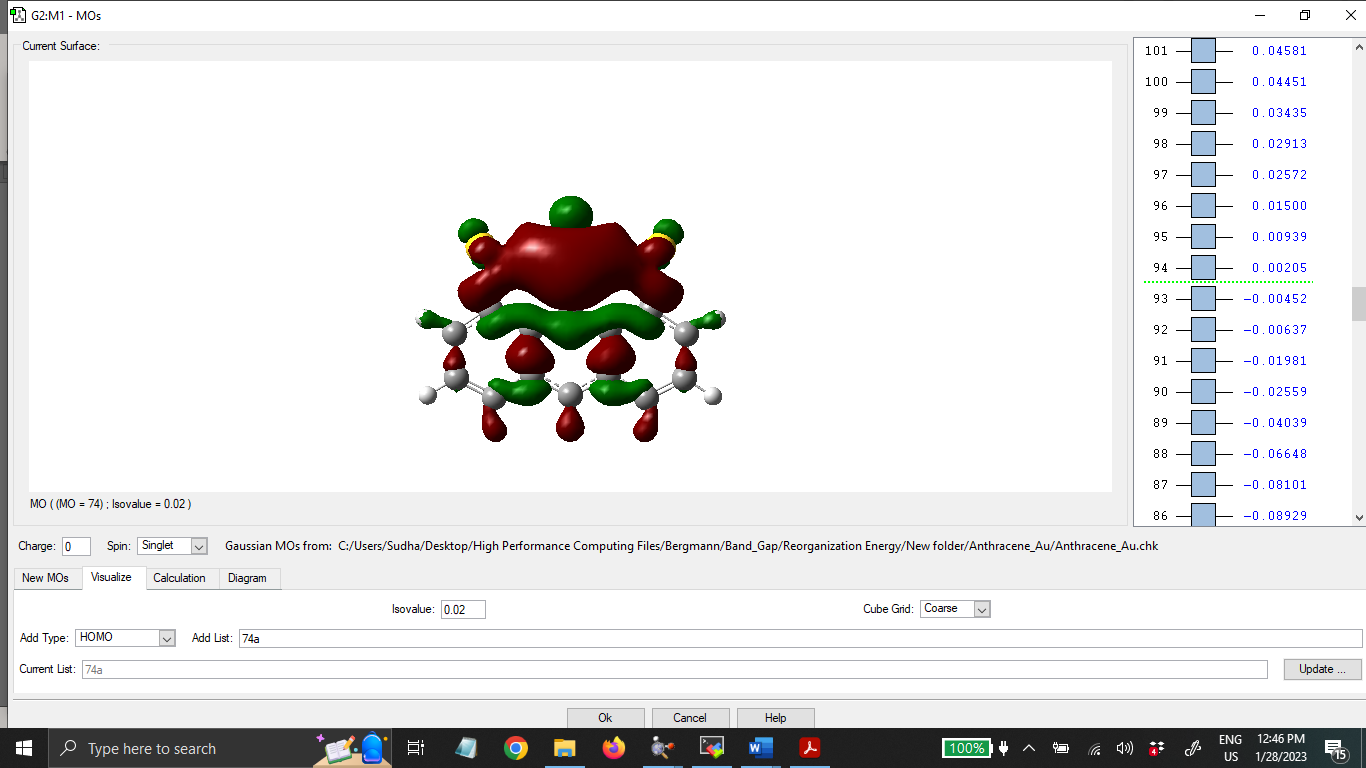 | 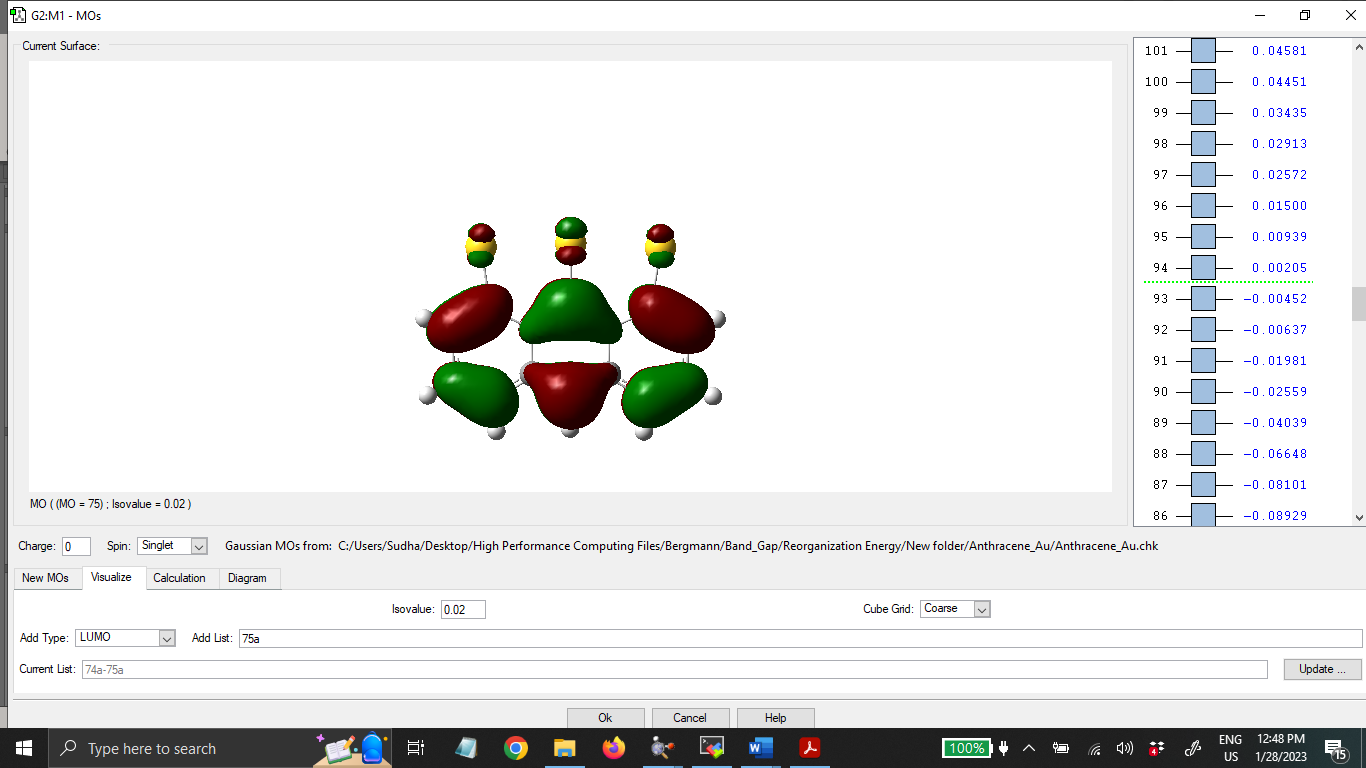 |
|  | 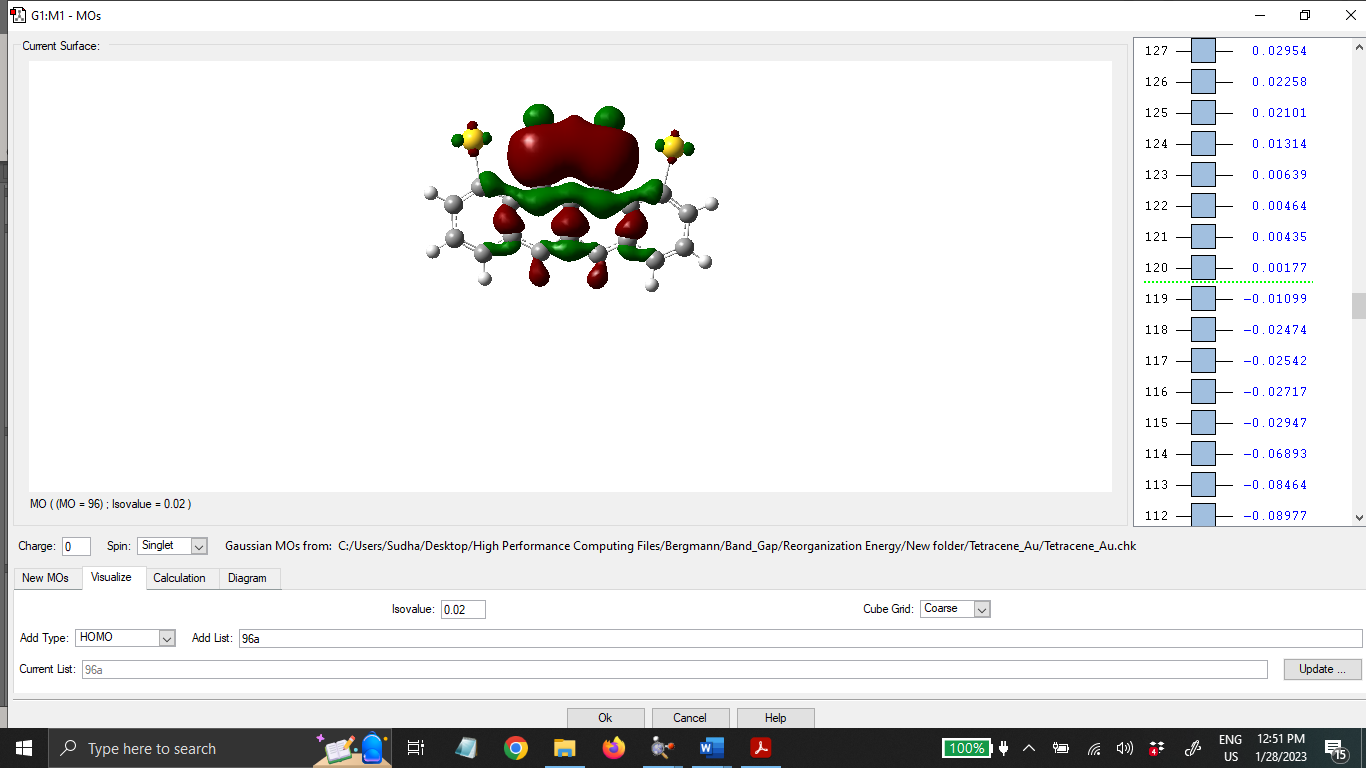 | 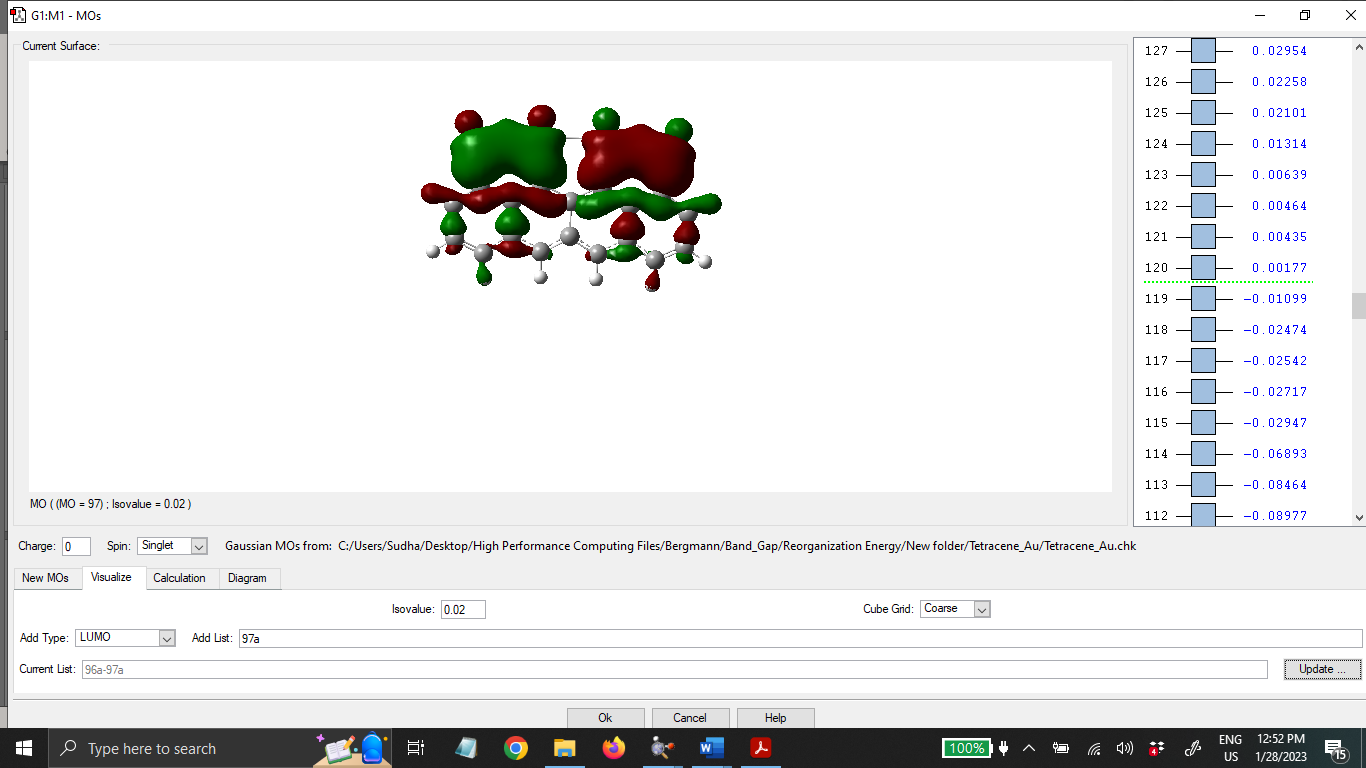 |
|  | 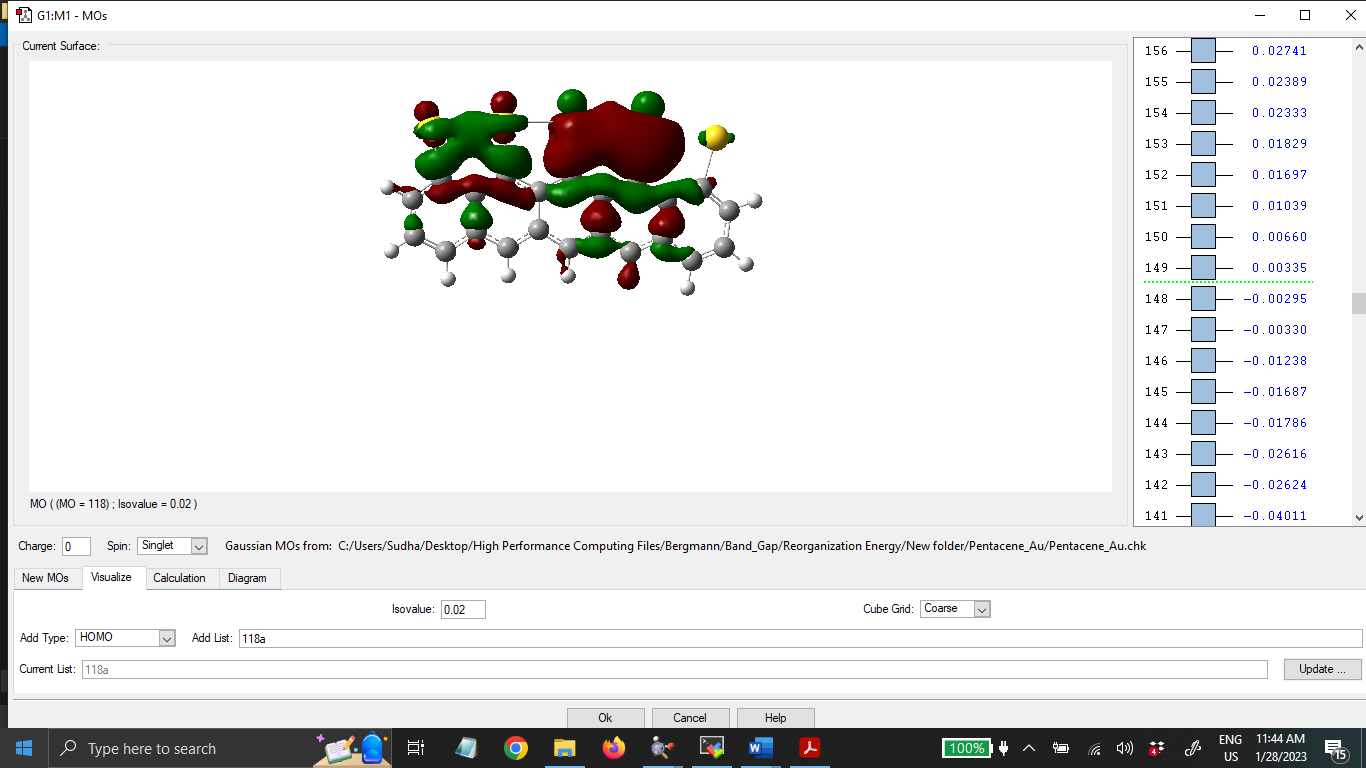 | 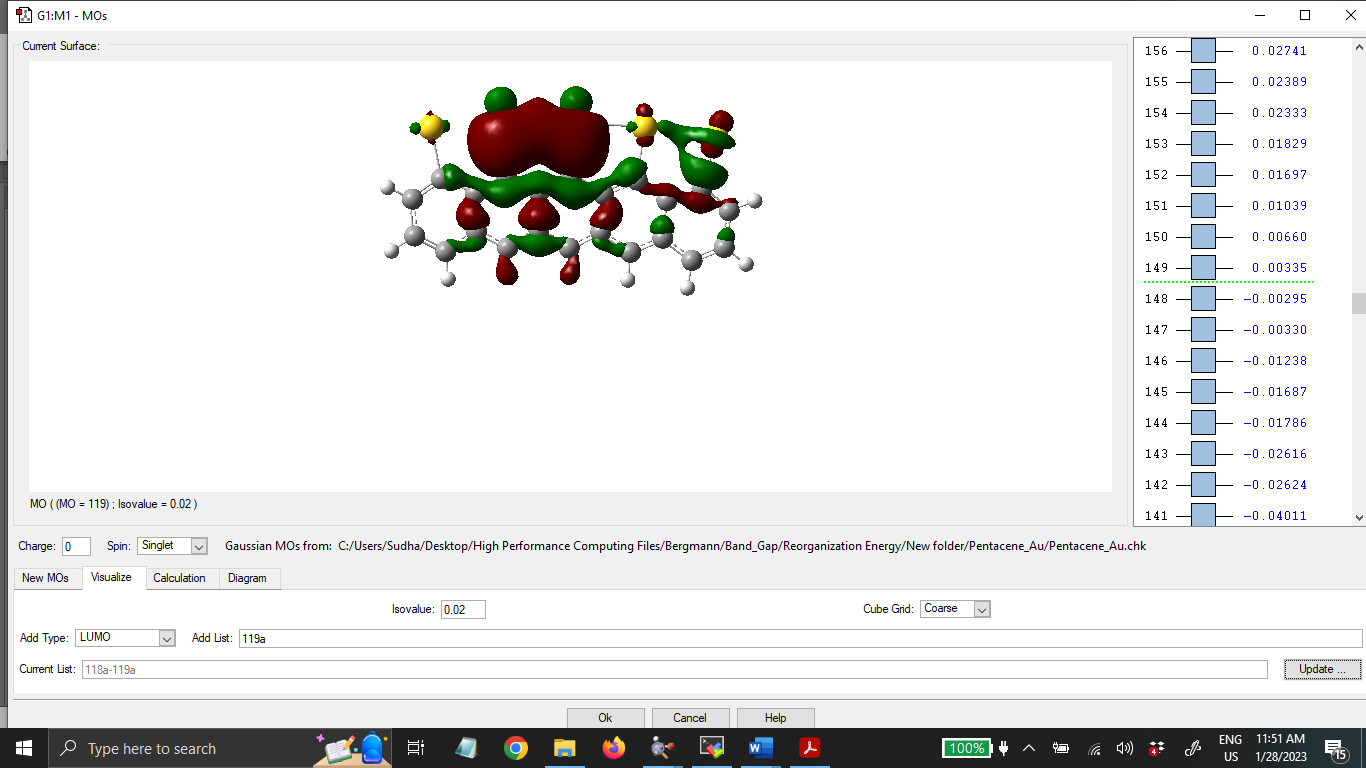 |
